# Supplementary material for: FUTURE-GB: functional and ultrasound-guided resection of glioblastoma – a two-stage randomised control trial
Source: BMJ Open. 2022 Nov 15;12(11):e064823. doi: 10.1136/bmjopen-2022-064823 (PMC9668053; doi:10.1136/bmjopen-2022-064823)
Supplement: Supplementary data [file bmjopen-2022-064823supp001.pdf]

BMJ Publishing Group Limited (BMJ) disclaims all liability and responsibility arising from any reliance placed on this supplemental material which has been supplied by the author(s)

FUTURE-GB STAGE 1 SITE DATA COMPLETION ASSESSMENT

**Reviewers:**	*Intra operative workflow & DTI:*	Prof Natalie Voets, Puneet Plaha, Miss Joy Roach, Amy Taylor
	Intra operative workflow & US:	Dipankar Nandi, Sophie Camp, Luke Dixon, Amy Taylor
	REDCap data entry & workflow:	Amy Taylor, Jack Morris, Puneet Plaha

**Site name:**		**Total Patients recruited:**		**Total Patients screened:**	

**Study ID**					
**Date of review**					
**Date of surgery**					
**Awake or GA surgery**					
**Pre -op tumour planning on MRI scans**					
**Pre-op tumour volume (cm^3)**					
**Post-op tumour volume (cm^3)**					
**Comments:**					
**MRI DTI and USG Acquisition**					
DTI – Site engaged with trials unit regarding DTI acquisition protocols ?	Yes/No	Yes/No	Yes/No	Yes/No	Yes/No
DTI scan acquired for surgery?	Yes/No	Yes/No	Yes/No	Yes/No	Yes/No
DTI Tracts reconstructed ?	Yes/No	Yes/No	Yes/No	Yes/No	Yes/No
USG used during surgery?	Yes/No	Yes/No	Yes/No	Yes/No	Yes/No
**Redcap data entry complete?**	Yes/No	Yes/No	Yes/No	Yes/No	Yes/No
Any difficulty entering data on REDCap ?	Yes/No	Yes/No	Yes/No	Yes/No	Yes/No
**Intraoperative workflow & Quentry imaging data transfer complete**	Yes/No	Yes/No	Yes/No	Yes/No	Yes/No
Data anonymised	Yes/No	Yes/No	Yes/No	Yes/No	Yes/No
Pre-op MRI scan performed	Yes/No	Yes/No	Yes/No	Yes/No	Yes/No
Pre -op MRI scan transferred					
Any Intra-op Screenshots acquired for awake surgery	Yes/No/ N/A	Yes/No/ N/A	Yes/No/ N/A	Yes/No/ N/A	Yes/No/ N/A
Intra-op Screenshots acquired for GA neurophysiology	Yes/No/ N/A	Yes/No/ N/A	Yes/No/ N/A	Yes/No/ N/A	Yes/No/ N/A
USG pre-resection – pictures/videos	Yes/No	Yes/No	Yes/No	Yes/No	Yes/No
USG post-resection- pictures/videos	Yes/No	Yes/No	Yes/No	Yes/No	Yes/No
Post-op MRI scan performed	Yes/No	Yes/No	Yes/No	Yes/No	Yes/No

FUTURE-GB_S1_Template_SiteDataCompletion_V3.0_18Oct2021.docx

FUTURE-GB STAGE 1 SITE DATA COMPLETION ASSESSMENT

**Post op MRI scan transferred**	Yes/No	Yes/No	Yes/No	Yes/No	Yes/No
**Difficulties in transferring Data to Quentry**	Yes/No	Yes/No	Yes/No	Yes/No	Yes/No
**Site PI - DTI comments**					
**Reviewer - DTI comments**					
**Reviewer - US comments**					
**Site PI - US comments**					
**Primary outcomes for Stage 1 complete**	Yes/No	Yes/No	Yes/No	Yes/No	Yes/No
Operation length					
Successful use of DTI neuronavigation and iUS to achieve complete tumour resection without major neurological deficit *(success defined here as appropriate and competent use of the imaging technologies to achieve the projected surgical outcome for each patient)*					
Extent of tumour resection assessed on postoperative MRI scan					
Surgical Complication and Serious Adverse Events (if applicable)	Yes/No	Yes/No	Yes/No	Yes/No	Yes/No
**Reviewer Comments:**					
**Analysis of imaging data to answer tertiary end point of the RCT study**					
Utility of imaging data to answer the DTI tertiary endpoint					
Utility of imaging data to answer the US tertiary endpoint					
**Reviewer Comments**					

**Overall reviewer comments**	

SITE FEEDBACK LOG

Date of meeting/discussion with site during Stage 1	Type of meeting	Feedback comments

FUTURE-GB_S1_Template_SiteDataCompletion_V3.0_18Oct2021.docx
