# Supplementary material for: FUTURE-GB: functional and ultrasound-guided resection of glioblastoma – a two-stage randomised control trial
Source: BMJ Open. 2022 Nov 15;12(11):e064823. doi: 10.1136/bmjopen-2022-064823 (PMC9668053; doi:10.1136/bmjopen-2022-064823)
Supplement: Supplementary data [file bmjopen-2022-064823supp002.pdf]

FUTURE-GB STAGE 2 PROGRESSION AGREEMENT

The Co-Chief investigators of the FUTURE-GB trial agree that the trial site:

<site name and NHS Trust>

has met the following provisions in Stage 1 of the FUTURE-GB trial and recruited <number> participants

Trial team review was conducted on DDMonYYYY and it was agreed on this date (DDMonYYYY) that this site can proceed to Stage 2.

Co-Chief investigators report:

This should include:

1. *Objective endpoints of Stage 1*
2. *Quality of DT and US imaging data*
3. *Any difficulties regarding data-workflow from site*
4. *Any suggestions for improvement*

Note: Completion of this agreement by all signatories will result in the Stage 1 Registration System and screening system being closed by the Trial Manager on or after this date, and requesting that the site is opened to recruitment in the Stage 2 Screening, Randomisation and Database System. The Stage 1 Database system will not be closed to the site until all outstanding data has been entered and cleaned/queried as required by the Trial Statistician.

FUTURE-GB STAGE 2 PROGRESSION AGREEMENT

Name	Signature	Date
Professor Puneet Plaha		
Professor Dipankar Nandi		
Miss Sophie Camp		

Plaha P, *et al. BMJ Open* 2022; 12:e064823. doi: 10.1136/bmjopen-2022-064823
