# Supplementary material for: FUTURE-GB: functional and ultrasound-guided resection of glioblastoma – a two-stage randomised control trial
Source: BMJ Open. 2022 Nov 15;12(11):e064823. doi: 10.1136/bmjopen-2022-064823 (PMC9668053; doi:10.1136/bmjopen-2022-064823)
Supplement: Supplementary data [file bmjopen-2022-064823supp004.pdf]

PARTICIPANT ID: FG-XX-XXXX

SITE ADMINISTERED QUESTIONNAIRE

Please tick which time point this questionnaire relates to:

Baseline	☐	12 months	☐
Hospital Discharge	☐	15 months	☐
6 weeks	☐	18 months	☐
3 months	☐	21 months	☐
6 months	☐	24 months	☐
9 months	☐		

Date of completion: DD/MM/YYYY

Completed by: (PRINT NAME)…………………………………………

This document contains the following validated questionnaires:

- Barthel Index
- MOCA Assessment
- MRC Muscle Power Scale
- WHO Performance Status

PARTICIPANT ID: FG-XX-XXXX

BARTHEL INDEX

Activity	Score
**FEEDING**<br>0 = unable<br>5 = needs help cutting, spreading butter, etc., or requires modified diet<br>10 = independent	_____
**BATHING**<br>0 = dependent<br>5 = independent (or in shower)	_____
**GROOMING**<br>0 = needs to help with personal care<br>5 = independent face/hair/teeth/shaving (implements provided)	_____
**DRESSING**<br>0 = dependent<br>5 = needs help but can do about half unaided<br>10 = independent (including buttons, zips, laces, etc.)	_____
**BOWELS**<br>0 = incontinent (or needs to be given enemas)<br>5 = occasional accident<br>10 = continent	_____
**BLADDER**<br>0 = incontinent, or catheterized and unable to manage alone<br>5 = occasional accident<br>10 = continent	_____
**TOILET USE**<br>0 = dependent<br>5 = needs some help, but can do something alone<br>10 = independent (on and off, dressing, wiping)	_____
**TRANSFERS (BED TO CHAIR AND BACK)**<br>0 = unable, no sitting balance<br>5 = major help (one or two people, physical), can sit<br>10 = minor help (verbal or physical)<br>15 = independent	_____
**MOBILITY (ON LEVEL SURFACES)**<br>0 = immobile or < 50 yards<br>5 = wheelchair independent, including corners, > 50 yards<br>10 = walks with help of one person (verbal or physical) > 50 yards<br>15 = independent (but may use any aid; for example, stick) > 50 yards	_____
**STAIRS**<br>0 = unable<br>5 = needs help (verbal, physical, carrying aid)<br>10 = independent	_____
**TOTAL (0–100):**	_____

Plaha P, *et al. BMJ Open* 2022; 12:e064823. doi: 10.1136/bmjopen-2022-064823

PARTICIPANT ID: FG-XX-XXXX

MOCA ASSESSMENT

VISUOSPATIAL / EXECUTIVE

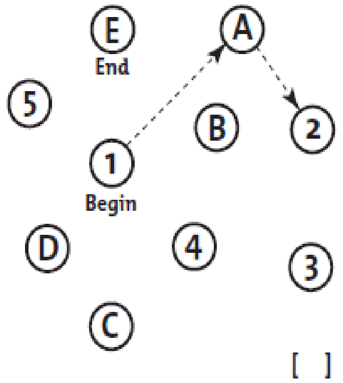

[ ]

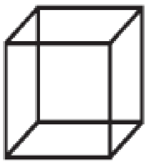

Copy cube

[ ]

Draw CLOCK (Ten past eleven) (3 points)

[ ] Contour [ ] Numbers [ ] Hands

POINTS: __/5

NAMING

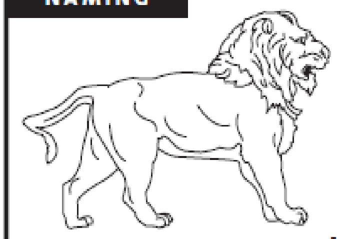
[ ]

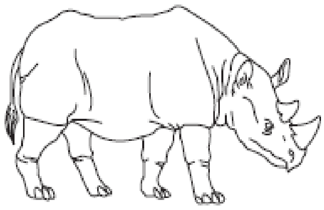
[ ]

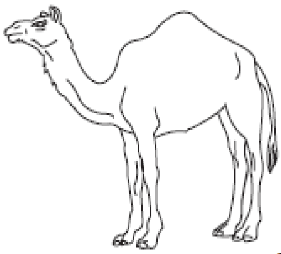
[ ]

__/3

MEMORY

Read list of words, subject must repeat them. Do 2 trials. Do a recall after 5 minutes.

	FACE	VELVET	CHURCH	DAISY	RED
1st trial					
2nd trial					

No points

ATTENTION

Read list of digits (1 digit/ sec.). Subject has to repeat them in the forward order [ ] 2 1 8 5 4
Subject has to repeat them in the backward order [ ] 7 4 2

__/2

Read list of letters. The subject must tap with his hand at each letter A. No points if ≥ 2 errors
[ ] F B A C M N A A J K L B A F A K D E A A A J A M O F A A B

__/1

Serial 7 subtraction starting at 100 [ ] 93 [ ] 86 [ ] 79 [ ] 72 [ ] 65
4 or 5 correct subtractions: **3 pts**, 2 or 3 correct: **2 pts**, 1 correct: **1 pt**, 0 correct: **0 pt**

__/3

LANGUAGE

Repeat : I only know that John is the one to help today. [ ]
The cat always hid under the couch when dogs were in the room. [ ]

__/2

Fluency / Name maximum number of words in one minute that begin with the letter F [ ] ____ (N ≥ 11 words)

__/1

ABSTRACTION

Similarity between e.g. banana - orange = fruit [ ] train – bicycle [ ] watch - ruler

__/2

DELAYED RECALL

		FACE	VELVET	CHURCH	DAISY	RED	
	Has to recall words WITH NO CUE	[ ]	[ ]	[ ]	[ ]	[ ]	Points for UNCUED recall only
Optional	Category cue						
	Multiple choice cue						

__/5

ORIENTATION

[ ] Date [ ] Month [ ] Year [ ] Day [ ] Place [ ] City

__/6

© Z.Nasreddine MD Version November 7, 2004
www.mocatest.org

Normal ≥ 26 / 30

TOTAL __/30
Add 1 point if ≤ 12 yr edu

PARTICIPANT ID: FG-XX-XXXX

MRC MUSCLE POWER SCALE

Score	Description
**0**	No contraction
**1**	Flicker or trace of contraction
**2**	Active movement, with gravity eliminated
**3**	Active movement against gravity
**4**	Active movement against gravity and resistance
**5**	Normal power

Please grade the 4 limbs of the patient

Left arm: ☐

Right arm: ☐

Left leg: ☐

Right leg: ☐

WHO PERFORMANCE STATUS

The WHO performance status classification categorises patients as:

- 0: able to carry out all normal activity without restriction
- 1: restricted in strenuous activity but ambulatory and able to carry out light work
- 2: ambulatory and capable of all self-care but unable to carry out any work activities; up and about more than 50% of waking hours
- 3: symptomatic and in a chair or in bed for greater than 50% of the day but not bedridden
- 4: completely disabled; cannot carry out any self-care; totally confined to bed or chair.

Please circle the WHO Performance status for the patient as at today:

0 1 2 3 4

Plaha P, *et al. BMJ Open* 2022; 12:e064823. doi: 10.1136/bmjopen-2022-064823
