# Supplementary material for: FUTURE-GB: functional and ultrasound-guided resection of glioblastoma – a two-stage randomised control trial
Source: BMJ Open. 2022 Nov 15;12(11):e064823. doi: 10.1136/bmjopen-2022-064823 (PMC9668053; doi:10.1136/bmjopen-2022-064823)
Supplement: Supplementary data [file bmjopen-2022-064823supp005.pdf]

PARTICIPANT ID: FG-XX-XXXX

PARTICIPANT QUESTIONNAIRE

Thank you for agreeing to participate in the FUTURE-GB study.

Please tick which time point this questionnaire relates to:

- Hospital Discharge ☐
- 6 weeks ☐
- 3 months ☐
- 6 months ☐
- 9 months ☐
- 12 months ☐
- 15 months ☐
- 18 months ☐
- 21 months ☐
- 24 months ☐

We would be grateful if you could complete this questionnaire on how you are feeling and if you are having any issues due to your glioblastoma.

If you have any questions about the study or this questionnaire, please do not hesitate to contact a member of the study team on future-gb@nds.ox.ac.uk or call 01865 xxxxxx (Monday to Friday, 9-4pm), there is an answering machine for messages outside of these times, or contact your local clinical team)

PARTICIPANT ID: FG-XX-XXXX

We are interested in some things about you and your health.

Please answer all of the questions yourself by circling the number that best applies to you. There are no "right" or "wrong" answers. The information that you provide will remain strictly confidential.

We appreciated that you may be very busy and this may be a distressing time, therefore if you are unable to complete this questionnaire either due to time or other reasons – if at all possible however we would be very grateful if could complete the date below and questions 29 and 30 on page 4.

What is today's date: DD/MM/YYYY

PARTICIPANT ID: FG-XX-XXXX

		Not at All	A Little	Quite a Bit	Very Much
1.	Does you have any trouble doing strenuous activities, like carrying a heavy shopping bag or a suitcase?	1	2	3	4
2.	Do you have any trouble taking a <u>long</u> walk?	1	2	3	4
3.	Do you have any trouble taking a <u>short</u> walk outside of the house?	1	2	3	4
4.	Do you need to stay in bed or a chair during the day?	1	2	3	4
5.	Do you need help with eating, dressing, washing yourself or using the toilet?	1	2	3	4

During the past week:

		Not at All	A Little	Quite a Bit	Very Much
6.	Were you limited in doing either your work or other daily activities?	1	2	3	4
7.	Were you limited in pursuing their hobbies or other leisure time activities?	1	2	3	4
8.	Were you short of breath?	1	2	3	4
9.	Have you had pain?	1	2	3	4
10.	Did you need to rest?	1	2	3	4
11.	Have you had trouble sleeping?	1	2	3	4
12.	Have you felt weak?	1	2	3	4
13.	Have you lacked their appetite?	1	2	3	4
14.	Have you felt nauseated?	1	2	3	4
15.	Have you vomited?	1	2	3	4
16.	Have you been constipated?	1	2	3	4
17.	Have you had diarrhoea?	1	2	3	4
18.	Were you tired?	1	2	3	4
19.	Did pain interfere with your daily activities?	1	2	3	4
20.	Have you had difficulty concentrating on things, like reading a newspaper or watching television?	1	2	3	4
21.	Did you feel tense?	1	2	3	4
22.	Did you worry?	1	2	3	4
23.	Did you feel irritable?	1	2	3	4
24.	Did you feel depressed?	1	2	3	4
25.	Have you had difficulty remembering things?	1	2	3	4
26.	Has your physical condition or medical treatment interfered with your <u>family</u> life?	1	2	3	4
27.	Has your physical condition or medical treatment interfered with your <u>social</u> activities?	1	2	3	4

Plaha P, *et al. BMJ Open* 2022; 12:e064823. doi: 10.1136/bmjopen-2022-064823

PARTICIPANT ID: FG-XX-XXXX

		Not at All	A Little	Quite a Bit	Very Much
28.	Has your physical condition or medical treatment caused you financial difficulties?	1	2	3	4

For the following questions please circle the number between 1 and 7 that best applies to you

29. How would you rate your overall <u>health</u> during the past week?

1 2 3 4 5 6 7

Very poor Excellent

30. How would you rate your overall <u>quality of life</u> during the past week?

1 2 3 4 5 6 7

Very poor Excellent

Patients sometimes report that they have the following symptoms. Please indicate the extent to which you have experienced the below symptoms or problems during the past week.

During the past week:

		**Not at All**	**A Little**	**Quite a Bit**	**Very Much**
31.	Did you feel uncertain about the future?	1	2	3	4
32.	Did you feel you had setbacks in your condition?	1	2	3	4
33.	Were you concerned about disruption of family life?	1	2	3	4
34.	Did you have headaches?	1	2	3	4
35.	Did your outlook on the future worsen?	1	2	3	4
36.	Did you have double vision?	1	2	3	4

Plaha P, *et al. BMJ Open* 2022; 12:e064823. doi: 10.1136/bmjopen-2022-064823

PARTICIPANT ID: FG-XX-XXXX

		**Not at All**	**A Little**	**Quite a Bit**	**Very Much**
37.	Was your vision blurred?	1	2	3	4
38.	Did you have difficulty reading because of your vision?	1	2	3	4
39.	Did you have seizures?	1	2	3	4
40.	Did you have weakness on one side of your body?	1	2	3	4
41.	Did you have trouble finding the right words to express yourself?	1	2	3	4
42.	Did you have difficulty speaking?	1	2	3	4
43.	Did you trouble communicating your thoughts?	1	2	3	4
44.	Did you feel drowsy during the daytime?	1	2	3	4
45.	Did you have trouble with your coordination?	1	2	3	4
46.	Did hair loss bother you?				
47.	Did itching of their skin bother you?	1	2	3	4
48.	Did you have weakness of both legs?	1	2	3	4
49.	Did you feel unsteady on your feet?	1	2	3	4
50.	Did you have trouble controlling your bladder?	1	2	3	4

Plaha P, *et al. BMJ Open* 2022; 12:e064823. doi: 10.1136/bmjopen-2022-064823
