# Supplementary material for: FUTURE-GB: functional and ultrasound-guided resection of glioblastoma – a two-stage randomised control trial
Source: BMJ Open. 2022 Nov 15;12(11):e064823. doi: 10.1136/bmjopen-2022-064823 (PMC9668053; doi:10.1136/bmjopen-2022-064823)
Supplement: Supplementary data [file bmjopen-2022-064823supp007.pdf]

PROXY ID: FG-XX-XXXX

PROXY QUESTIONNAIRE

Thank you for agreeing to participate in the FUTURE-GB study as a proxy.

This is the 3 months post operation questionnaire.

We would be grateful if you could complete the questionnaire on your opinion of how you friend/spouse/relative is coping in their glioblastoma journey. This is the same questionnaire as all the others you have completed to date.

If you have any questions about the study or this questionnaire, please do not hesitate to contact a member of the study team on future-gb@nds.ox.ac.uk or call 01865 xxxxxx (Monday to Friday, 9-4pm, there is an answering machine for messages outside of these times)

Plaha P, et al. BMJ Open 2022; 12:e064823. doi: 10.1136/bmjopen-2022-064823

PROXY ID: FG-XX-XXXX

We are interested in some things about the person and their health of the person you have agreed to act as a proxy for. The below questionnaire will use the term friend – we appreciate though that you may be answering about a relative/friend or spouse. We hope you will allow the use of this term for the purposes of this questionnaire.

Please answer all of the questions yourself by circling the number that best applies to them. There are no “right” or “wrong” answers. The information that you provide will remain strictly confidential.

We appreciated that you may be very busy and this may be a distressing time, therefore if you are unable to complete this questionnaire either due to time or other reasons – if at all possible however we would be very grateful if could complete the date below and questions 29 and 30 on page 4.

What is today’s date: DD/MM/YYYY

This document contains the following validated questionnaires:

- EORTC QLQ-C30
- EORTC QLQ - BN20

Plaha P, *et al. BMJ Open* 2022; 12:e064823. doi: 10.1136/bmjopen-2022-064823

PROXY ID: FG-XX-XXXX

		Not at All	A Little	Quite a Bit	Very Much
1.	Does your friend have any trouble doing strenuous activities, like carrying a heavy shopping bag or a suitcase?	1	2	3	4
2.	Does your friend have any trouble taking a long walk?	1	2	3	4
3.	Does your friend have any trouble taking a short walk outside of the house?	1	2	3	4
4.	Does your friend need to stay in bed or a chair during the day?	1	2	3	4
5.	Does your friend need help with eating, dressing, washing themselves or using the toilet?	1	2	3	4

During the past week:

		Not at All	A Little	Quite a Bit	Very Much
6.	Was your friend limited in doing either work or other daily activities?	1	2	3	4
7.	Was your friend limited in pursuing their hobbies or other leisure time activities?	1	2	3	4
8.	Were they short of breath?	1	2	3	4
9.	Have they had pain?	1	2	3	4
10.	Have they needed to rest?	1	2	3	4
11.	Have they had trouble sleeping?	1	2	3	4
12.	Have they felt weak?	1	2	3	4
13.	Have they lacked their appetite?	1	2	3	4
14.	Have they felt nauseated?	1	2	3	4
15.	Have they vomited?	1	2	3	4
16.	Have they been constipated?	1	2	3	4
17.	Have they had diarrhoea?	1	2	3	4
18.	Have they been tired?	1	2	3	4
19.	Has pain interfered with their daily activities?	1	2	3	4
20.	Have they have difficulty concentrating on things, like reading a newspaper or watching television?	1	2	3	4
21.	Have they felt tense?	1	2	3	4
22.	Have they worried?	1	2	3	4
23.	Have they felt irritable?	1	2	3	4
24.	Have they felt depressed?	1	2	3	4
25.	Have they had difficulty remembering things?	1	2	3	4
26.	Has their physical condition or medical treatment interfered with their family life?	1	2	3	4

PROXY ID: FG-XX-XXXX

		Not at All	A Little	Quite a Bit	Very Much
27.	Has their physical condition or medical treatment interfered with their social activities?	1	2	3	4
28.	Has their physical condition or medical treatment caused them financial difficulties?	1	2	3	4

For the following questions please circle the number between 1 and 7 that best applies to the person you are a proxy for in your opinion

29. How would you rate their overall health during the past week?

1 2 3 4 5 6 7

Very poor Excellent

30. How would you rate their overall quality of life during the past week?

1 2 3 4 5 6 7

Very poor Excellent

Patients sometimes report that they have the following symptoms. Please indicate the extent to which your relative/proxy/friend have experienced the below symptoms or problems during the past week.

During the past week:

		**Not at All**	**A Little**	**Quite a Bit**	**Very Much**
31.	Did they feel uncertain about the future?	1	2	3	4
32.	Did they feel that have had setbacks in their condition?	1	2	3	4
33.	Were they concerned about disruption of family life?	1	2	3	4
34.	Did they have headaches?	1	2	3	4

Plaha P, *et al. BMJ Open* 2022; 12:e064823. doi: 10.1136/bmjopen-2022-064823

PROXY ID: FG-XX-XXXX

		Not at All	A Little	Quite a Bit	Very Much
35.	Did their outlook on the future worsen?	1	2	3	4
36.	Did they have double vision?	1	2	3	4
37.	Has their vision blurred?	1	2	3	4
38.	Did they have difficulty reading because of their vision?	1	2	3	4
39.	Did they have seizures?	1	2	3	4
40.	Did they have weakness on one side of their body?	1	2	3	4
41.	Did they have trouble finding the right words to express themselves?	1	2	3	4
42.	Did they have difficulty speaking?	1	2	3	4
43.	Did they have trouble communicating their thoughts?	1	2	3	4
44.	Did they feel drowsy during the daytime?	1	2	3	4
45.	Did they trouble with their coordination?	1	2	3	4
46.	Did hair loss bother them?				
47.	Did itching of their skin bother them?	1	2	3	4
48.	Did they have weakness of both of their legs?	1	2	3	4
49.	Did they feel unsteady on their feet?	1	2	3	4
50.	Did they have trouble controlling their bladder?	1	2	3	4

Plaha P, *et al. BMJ Open* 2022; 12:e064823. doi: 10.1136/bmjopen-2022-064823
