# Supplementary material for: FUTURE-GB: functional and ultrasound-guided resection of glioblastoma – a two-stage randomised control trial
Source: BMJ Open. 2022 Nov 15;12(11):e064823. doi: 10.1136/bmjopen-2022-064823 (PMC9668053; doi:10.1136/bmjopen-2022-064823)
Supplement: Supplementary data [file bmjopen-2022-064823supp008.pdf]

NHS
Oxford University Hospitals
NHS Foundation Trust

Functional and Ultrasound guided Resection of Glioblastoma – the FUTURE-GB study –

Stage 1- IDEAL 2b Phase

FUTURE GB

Patient Information Leaflet

Invitation to join the FUTURE-GB study

We would like to invite you to take part in a research study (also called a clinical trial).

Before you decide whether to take part or not, it is important that you understand why we are doing this study and what it will involve.

Please take time to read the following information and talk to others about the study. If anything is unclear, or if you would like more information, please ask a member of the study team who will be happy to answer any questions.

What is the purpose of this study?

There are many different types of brain tumours. These can vary in how quickly they grow and what symptoms they cause. For a brain tumour that grows quickly it is important to remove as much tumour tissue as possible. To do this without causing damage to important functional parts of the brain involved in speaking, moving etc., we need accurate imaging during surgery. Several different types of imaging are used during operations, but at present we don't know how effective they are and whether they are actually better than standard care.

We have been funded by the National Institute of Health Research (NIHR) which receives its funding from the UK Government, to find out whether some of these available newer technologies improve quality of life, and life expectancy for people with brain tumours who undergo surgery. We also want to see if using these techniques during an operation means it takes more time for a tumour to come back, and if people have fewer complications from the surgery.

Stage 1

In the first part of the study (Stage 1) we are studying how to use these technologies in combination to achieve the best effect. This means that surgeons will be mentored in the best use of the imaging methods. The surgical teams too will be familiarised with a standard way of using them together. We will carefully monitor how the new methods are used during this phase and discuss them with the surgeons. The aim of this stage of the study is to standardise their use, as we want to have them used in the same way in all participating hospitals by the start of Stage 2. Everyone who agrees to take part in this stage may be helping the doctors treating you familiarise themselves with the new technologies. It is highly likely that your surgeon is already familiar with the use of these technologies.

Note: <u>You are only being asked to take part in Stage 1 and if you agree to take part you will only be part of Stage 1 of this study</u>

Plaha P, *et al. BMJ Open* 2022; 12:e064823. doi: 10.1136/bmjopen-2022-064823

NHS
Oxford University Hospitals
NHS Foundation Trust

For information – once approximately 5 people have agreed to take part in Stage 1 and their operations have taken place at this hospital, Stage 2 of the study will start at this hospital– the information below tells you more about Stage 2.

Stage 2

Stage 2 of the study will compare the operation using the new imaging techniques with the standard operation - so half of those that agree will have the additional, newer, technologies and half will have the standard operation. We hope this will allow us to find a definite answer about which technologies should be used during an operation. The aim of the surgery is to remove safely as much tumour as possible, whilst minimising the risks of damaging brain function and hence affecting quality of life.

The technologies that will be used in this study are all available and in use across NHS practice, and have been shown to be safe. No one knows whether using all of them together will have a definite positive effect on outcome, but it is logical to expect that it should.

The design of this study (FUTURE-GB) has involved patients, their families and healthcare professionals, including brain surgeons, using their knowledge and experience at every stage of project development.

Who is taking part and why have I been invited to take part?

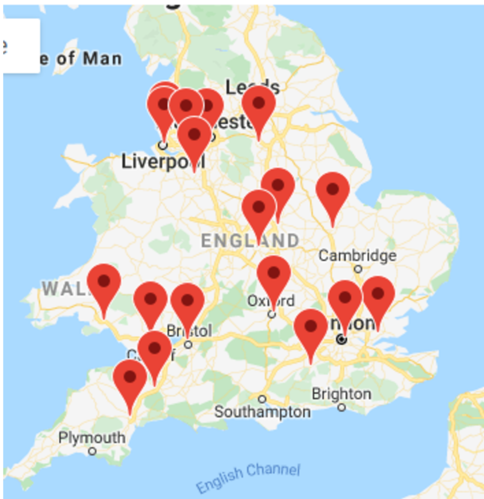

We are hoping to enrol 75 people aged 18 or over, from approximately15 neurosurgical centres in the UK. You will have surgery in your local hospital, which is participating in this trial.

You have been invited to take part because your brain scan suggests you have a brain tumour which comes from the brain itself, rather than from a cancer elsewhere in the body which has spread to the brain. Your scan also suggests the tumour is likely to be aggressive, called a glioblastoma or high-grade tumour.

Do I have to take part in this study?

No, you are under no obligation to take part in the study. Deciding not to will not affect the treatment/care you receive from your team. It is up to you to decide whether to take part or not.

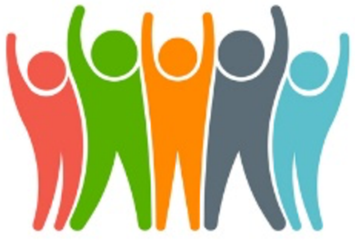

Please keep this leaflet and use it as it may to help you make your decision. If you decide to take part, you will be asked to sign another consent form, as well as that used for your NHS operation.

If you choose not to join the study, you will receive the routine NHS treatment, as agreed by your local treating team of healthcare professionals, in accordance with standard NHS practice as deemed appropriate by your treating team. A note will be made of your age and gender, so that we can find out who decides not to take part. You cannot be identified from this data. A researcher may ask you if you would be happy to give a reason for not wanting to take part in the study. Giving this information is entirely voluntary.

Should you decide to take part, you are still free to withdraw at any time and without giving a reason. This will not affect the standard of care you receive as either an inpatient or an outpatient.

FUNDED BY NIHR | National Institute for Health Research   OCTRU   SITU   UNIVERSITY OF OXFORD   Imperial College London

NHS
Oxford University Hospitals
NHS Foundation Trust

What will happen if I take part?

If you are happy to take part in this study, a researcher will ask you some simple questions and check your medical history to confirm that you are eligible.

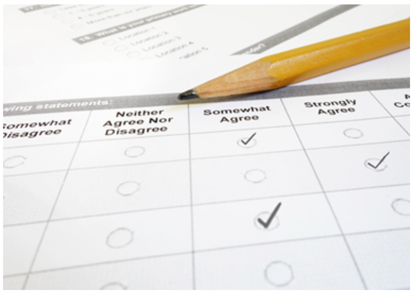

Initial assessment: If you are eligible and wish to participate, you will be asked to sign and date a consent form for the study. Researchers will then take from your medical notes, information about your brain tumour history including the symptoms you have had and where your tumour is.

The researchers would then record details about your operation and details of when you leave hospital after your operation. No questions will need to be asked of you as this will either be standard things already being recorded in your notes or when the technologies are being used, the settings being used in your operation.

Most significantly for those that agree to take part in FUTURE-GB the time taken for your preoperative scans (which you will have by being in the study or not) will perhaps take another 5 minutes. Your operation may also be slightly longer due to the technologies being used – this might perhaps extend it by 15 minutes. (Your doctors will talk to you about what happens in your preoperative scans – but we want you to know that some people find them quite claustrophobic – but the scans are needed for your surgery regardless of taking part in FUTURE-GB). Also, those who have metal in their body may potentially not be able to have type of scan called an MRI scan – talk to the doctors if you think you have metal in your body.

Please note: The design of this study has involved patients, their families and healthcare professionals, including brain surgeons, using their knowledge and experience at every stage of its development.

What technologies will be used in my operation?

Surgery will involve standard care and newer technologies

Standard care:

You will have an MRI scan before your operation. This can be used during the surgery to help your surgeon identify where your brain tumour is located, and what brain structures are close by. MRI stands for Magnetic Resonance Imaging. It is a type of scan that uses strong magnetic fields to produce detailed images. It is used for brain tumour surgery to obtain detailed images of your brain and specifically your brain tumour. There is no risk of radiation exposure. All MRI scans that you will receive are received by all those with a brain tumour, anything seen on these scans will be acted upon as per local NHS Trust and national guidelines.

This is combined with use of a chemical called 5-aminolevulinic acid (5-ALA), which is a drink taken a few hours before surgery. This allows the tumour cells to light up pink, when a blue light is shone on them during surgery. This has been shown to help surgeons remove more of the brain tumour, as they are able to see better the edges of the tumour and differentiate it from the surrounding normal brain. This makes sure as much of the tumour is removed as is possible, but it can never usually be totally removed.

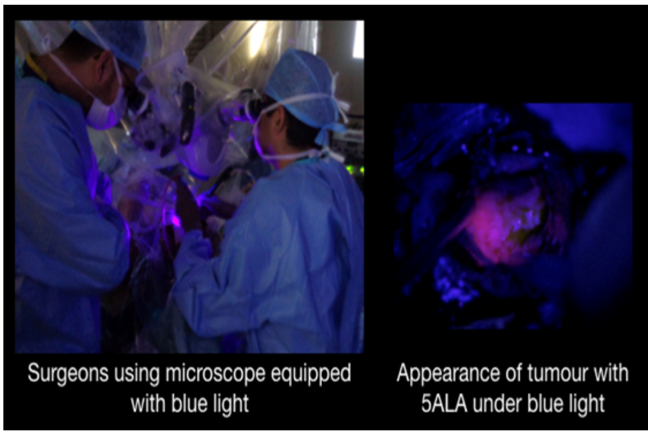

Surgeons using microscope equipped with blue light | Appearance of tumour with 5ALA under blue light

FUNDED BY NIHR National Institute for Health Research | OCTRU | SITU | UNIVERSITY OF OXFORD | Imperial College London

Newer technologies:

1) Diffusion Tensor Imaging (DTI) is an MRI technology which allows the surgeons to have a scan of all the nerve fibres which are involved in movement, speech etc. around a tumour. This means that when removing the tumour, the surgeon knows more easily where these are based on your DTI scan and can avoid them.

2) Intraoperative Ultrasound is a technology that uses high frequency sound waves to create an image of the brain tumour during the operation. The ultrasound provides "live" pictures of your tumour as surgery progresses and tumour is removed. The surgeon can use this as many times as necessary during your surgery. The ultrasound is the same as that used to provide a picture of a baby inside a pregnant woman.

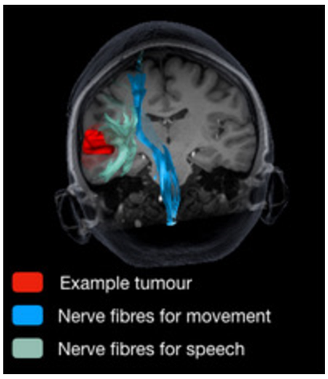

Both these technologies are safe and have been used in brain tumour surgery for a number of years. Your surgeon is familiar with their use and has used them during surgery. However, the benefit of using these 2 new technologies together, in addition to the present "standard of care" surgery has not been scientifically tested, or formally assessed. There are no extra drugs or chemicals used.

Further possible contacts: A researcher from the trial coordinating team may visit while you are having your operation so that we can check how the surgery is being undertaken. We will always check that you are happy for this to happen. If not, the researcher will not come into your operation. At the end of the study, we will report how well the treatments were delivered as it is important we fully understand this process.

Please note, no-one can ever be identified in any public report about the study.

There are 2 companies supporting this study by providing machinery and software to sites if they do not already have the equipment needed for this study. The companies are called BrainLab and Medtronic they are supporting doctors in the UK in using the new technologies. Neither company will be able to influence the results of the study.

What happens after my operation in FUTURE-GB?

Researchers will record information about your operation from your medical notes and directly into the study database, researchers will also collect information from your medical notes and scans when you are discharged home after your operation. The care, any tests, further outpatient appointments and any other surgery or treatments will not be changed by you agreeing to take part in FUTURE-GB. Researchers will check your medical notes for up to 6 months after your operation so find out how you are getting on – but there will be no further contact with you from the study team.

What are the benefits and risks of taking part in the study?

For those that take part in the study, your operation will be conducted by the same surgeon/surgical team whom you have already seen.

We hope the information from this study will answer the question:

Which imaging tools should be used by surgeons when removing a glioblastoma, to offer the highest chance of removing as much of the tumour as possible without causing functional problems, and therefore keeping a good quality of life?

We cannot promise the study will help you directly, but the information we get has the potential to be of benefit, potentially allowing more of your tumour to be removed safely.

The risks relating to the brain tumour surgery itself will be discussed with you in detail as part of the standard, routine consent for an operation. We do not think that being part of this study will change any of the risks of the operation but this is one of the things we be will be studying. The technologies will however add some time to the scan before your operation and during your operation.

We are undertaking this study because the extra imaging tools add significant costs to NHS treatment, and therefore we have also been funded to identify whether they provide a real benefit for people with brain tumours.

People sometimes feel uncomfortable answering certain questions about their health, or may be unable to answer. If you, or the person you nominate to answer for you, feel uncomfortable at any point, then you do not have to answer the questions.

Who will know that I am taking part?

The only people who will know that you are taking part in this study are the members of the research team and the healthcare professionals involved in your care. You can tell anyone you would like to that you are taking part.

The only people in the University of Oxford who will have access to information that identifies you will be people who need to contact you to about the study, or review the data. The people who analyse the information will not be able to identify you and will not be able to find out your name or contact details. Also, your de-identified scans will be reviewed by members of the research team and the companies providing the extra imaging technologies. The images will be transferred using cloud servers, however, nothing that could identify you will be included.

Representatives from the Sponsor, relevant regulatory organisations and [insert local Trust] may also need access to monitor or audit the study to ensure that the research is complying with applicable regulations.

Paperwork that is completed by you, the research team, or the treating clinical team, will be sent securely to the study team managing the FUTURE-GB study that are based at the University of Oxford.

Will my details be kept confidential?

Yes. All information collected about you and from you during the course of the research, including from your medical records, will be kept strictly confidential. Everyone who takes part in the study will be assigned a code number and all of the data relating to each person will be held on a computer database and will only be linked to that code number, and not to people's names or addresses. The study team will record into the study database your name, date of birth, NHS or CHI number, Hospital number and your email address. These details will allow the central study team and the local teams to ensure they are collecting data on the correct person. Your email address will only be used to allow you to complete the consent form at home, although this can also be done at the hospital, and to send you a copy of your consent form for your records. Your NHS or CHI number will be used to look up your status 6 months after agreeing to take part.

We will ask you for your permission for individuals from the University of Oxford and Imperial College London, and the regulatory authorities, to have access to your medical notes and data. This is in order that they may conduct checks on the study data that has been collected and to ensure all the study data has been completed correctly. We will also ask you for your permission to allow appropriate individuals from the NHS Trust that you are being approached at to also undertake this review.

At the end of the study, all of the data will be de-identified so that no-one can be identified. This de-identified data will be shared so that more researchers can gain a deeper understanding about patients

who have had surgery for glioblastoma. It may be shared with other researchers around the world and with commercial organisations but this information will not identify you, and will not be combined with other information in a way that could identify you. The information will only be used for the purpose of healthcare research, and cannot be used to contact you, nor will it affect your care.

In line with what happens in the NHS, the only situation that confidentiality would need to be broken would be if you told a health professional or research team member of something that could result in harm to yourself or others.

What will happen to my data?

Research is carried out in the public interest. The University of Oxford, as Sponsor, is the data controller. This means that we, as University of Oxford researchers, are responsible for looking after your information as part of FUTURE-GB, and using it properly. We will use the minimum possible personally-identifiable information, and this will be kept for 12 months after the study has finished. Non-identifiable research data and any research documents with personal information, will be stored securely at the University of Oxford for a maximum of 5 years after the end of the study, as part of the research record.

We will be using information from you and your medical records in order to carry out this study. The Oxford University Hospitals NHS Foundation Trust will use your NHS number and contact details to get in touch with you, and to make sure that relevant study information is recorded from your care records. They will keep your identifiable information safely for 12 months after the study has finished. Consent forms and study documents held at [local NHS Trust name] will be archived securely, in accordance with their local procedures.

Data protection regulation provides you with control over your personal data and how it is used. When you agree to your information being used in research, however, some of those rights may be limited, as we need to manage your information in specific ways in order for the research to be reliable and accurate. Further information about your rights with respect to your personal data, is available at https://compliance.web.ox.ac.uk/individual-rights

You can find out more about how we use your information by contacting the FUTURE-GB study team on: futuregb@nds.ox.ac.uk.

What will happen if I don't want to carry on with the study?

You are free to withdraw from taking part in the study at any time without giving a reason. Please remember, it is your decision to take part. If you agree to take part now, but you change your mind during the study, this will not change the standard of care you receive from the NHS. If you were to decide to stop taking part in the study at any time, any data collected on you would be kept. You would not be contacted about the study again or have any further data collected.

What happens at the end of the study?

We will share the results with healthcare researchers and professionals to improve future patient care. Also, we will present them in research reports, at scientific conferences, and publish them in scientific journals, and publish them on the study website futuregb.octru.ox.ac.uk.

We will not include any data that could identify you in the results. If the funders of this research ask us to make the study data available for other researchers, we will first de-identify your information (i.e. we will take your name and other identifying details out) so that you cannot be identified.

NHS
Oxford University Hospitals
NHS Foundation Trust

Who is organising and funding the research?

The University of Oxford is the Sponsor and is organising this study. It is being conducted by a research team led by Prof Puneet Plaha, Consultant Neurosurgeon at the Oxford University Hospitals NHS Foundation Trust and the University of Oxford, and Miss Sophie Camp and Prof. Dipankar Nandi (both Consultant Neurosurgeons at Imperial College Healthcare NHS Trust).

The National Institute of Health Research – Health Technology Assessment programme is funding the study. The funding for the NIHR comes from the UK Government.

Who has approved this study?

A panel of independent researchers and patient representatives, as well as a Research Ethics Committee (REC Reference 20/LO/0840) have reviewed and approved this study.

What if I have concerns?

The University of Oxford, as the study sponsor, has appropriate insurance in place in the unlikely event that you suffer any harm as a direct consequence of your participation in this study.

If you have any concerns or complaints about any aspect of the study, please contact the FUTURE-GB research team using the details below. You can also contact the University of Oxford Research Governance, Ethics & Assurance office on 01865 616480 or by email on ctrg@admin.ox.ac.uk.

If you would prefer to speak with someone who is not involved in the study, then please contact the Patient Advice and Liaison Service (PALS). PALS is a confidential NHS service that can provide you with support for any complaints or queries you have regarding the care you receive as an NHS patient. However, PALS cannot provide information about this research study.

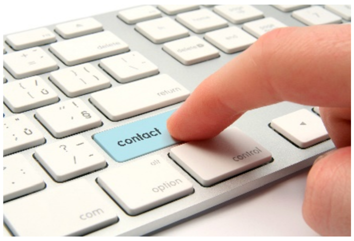

PALS phone number: [local PALS phone number]

PALS email: [local PALS email]

You can also contact your local clinical team directly:

<local PI/research team name and contact details>

If you have any questions about the study, please contact the FUTURE-GB team on:

Email: futuregb@nds.ox.ac.uk Telephone: 07917 101 649

Postal address: FUTURE-GB study, Botnar Research Centre, Nuffield Orthopaedic Centre, Windmill Road, Oxford, OX3 7LD.

Further information can be found on our study website – futuregb.octru.ox.ac.uk

Thank you for reading this information leaflet and considering taking part.

FUTURE GB

FUNDED BY NIHR National Institute for Health Research | OCTRU | SITU | UNIVERSITY OF OXFORD | Imperial College London
