# Supplementary material for: FUTURE-GB: functional and ultrasound-guided resection of glioblastoma – a two-stage randomised control trial
Source: BMJ Open. 2022 Nov 15;12(11):e064823. doi: 10.1136/bmjopen-2022-064823 (PMC9668053; doi:10.1136/bmjopen-2022-064823)
Supplement: Supplementary data [file bmjopen-2022-064823supp009.pdf]

LOCAL TRUST LOGO

Functional and Ultrasound guided Resection of Glioblastoma – the FUTURE-GB study – Stage 2 - Randomised Controlled Trial

Patient Information Leaflet

Invitation to join the FUTURE-GB study

We would like to invite you to take part in a research study (also called a clinical trial).

Before you decide whether to take part or not, it is important that you understand why we are doing this study and what it will involve.

Please take time to read the following information and talk to others about the study. If anything is unclear, or if you would like more information, please ask a member of the study team who will be happy to answer any questions.

What is the purpose of this study?

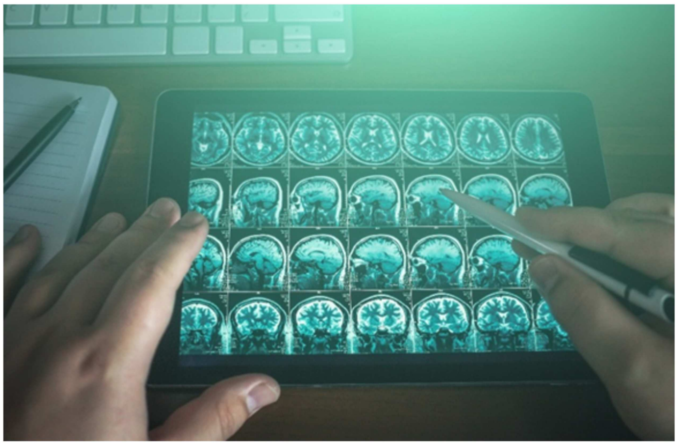

There are many different types of brain tumours. These can vary in how quickly they grow and what symptoms they cause. Studies have shown that when a brain tumour is growing quickly it is better to remove as much tumour as possible. Being able to do this without causing damage to the parts of the brain that are involved in things such as speaking and moving, surgeons need to be able to see clearly parts of the brain during surgery, using accurate imaging. This has led to an increase in the use of imaging (such as ultrasound and MRI scans) during operations. However, we don't know if all the extra imaging tools do definitely make a difference.

We have been funded by the National Institute of Health Research (NIHR) which receives its funding from the UK Government to find out whether some of these additional imaging tools available make a positive difference to quality of life for people with fast growing brain tumours who have surgery. We will also be looking to see if these imaging tools used during an operation mean people with a brain tumour:

- have a better quality of life?
- if it takes more/less time for their tumour to come back
- if they have more/fewer complications from the surgery.

LOCAL TRUST LOGO

This study will attempt to give a definite answer, so that surgeons know which imaging tools they should use during an operation to enable as much tumour as possible to be removed safely, whilst minimising the risks of damaging brain function and hence affecting quality of life.

The imaging tools that will be used in this study are available and in use across the NHS, and have been shown to be safe. However, no one knows if using them together will have a definite positive effect on outcome for those with a brain tumour.

Who is taking part and why have I been invited to take part?

Half of the people taking part in the study will have standard NHS imaging (scans), and the other half have standard NHS imaging (scans) and some additional imaging (scans).

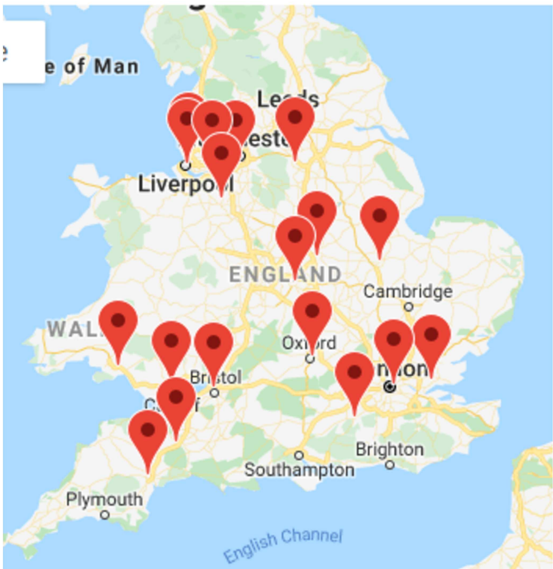

We want to enrol 357 people aged 18 - 70 from approximately 15 neurosurgical centres in the UK. will have surgery in your local neurosurgical unit, which is participating in this study.

You have been invited to take part because your scan suggests you have a brain tumour, which comes from the brain itself, rather than from a cancer elsewhere in the body which has spread to brain. Your scan also suggests the tumour is likely aggressive, called a glioblastoma, or high grade tumour.

Do I have to take part in this study?

No, you are under no obligation to take part in the study. Deciding not to will not affect the treatment/care you receive from your team. It is up to you to decide whether to take part or not. Please keep this leaflet and use it as it may to help you make your decision. If you decide to take part, you will be asked to sign another consent form, as well as that used for your NHS operation.

If you choose not to join the study, you will receive the routine NHS treatment, agreed by your local treating team of healthcare professionals, in accordance with standard NHS practice using the imaging your treating team deems appropriate. A note will be made of your age and gender, so that we can find out who decides not to take part. You cannot be identified from this data. A researcher may ask you if you would be happy to give a reason for not wanting to take part in the study. Giving this information is entirely voluntary.

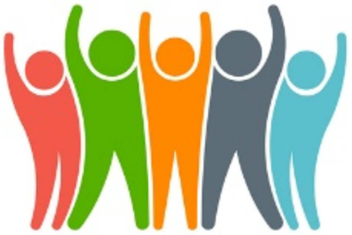

Should you decide to take part, you are still free to withdraw at any time and without giving a reason. This will not affect the standard of care you receive, as either an inpatient or an outpatient.

What will happen if I take part?

If you are happy to take part in this study, a researcher will ask you some simple questions and check your medical history to confirm that you are eligible.

Initial assessment: If you are eligible, you will be asked to sign and date a consent form for the study. We will also ask you to complete some short questionnaires about your health, the activities you are able to

LOCAL TRUST LOGO

carry out, and about your quality of life. These questionnaires should take you no more than 10-15 minutes to complete. (The questionnaires are electronic – but sometimes you may be given a paper questionnaire to complete, if the electronic system is not available). A researcher will also ask you to complete a brain activity and a recall test, check the strength in your arms and legs and talk to you about how you care for yourself.

Most significantly for those that agree to take part in FUTURE-GB the time taken for your preoperative scans (which you will have by being in the study or not) will perhaps take another 5 minutes. Your operation may also be slightly longer due to the technologies being used – this might perhaps extend it by 15 minutes. (Your doctors will talk to you about what happens in your preoperative scans – but we want you to know that some people find them quite claustrophobic –the scans are needed for your surgery regardless of taking part in FUTURE-GB). Also, those who have metal in their body may potentially not be able to have type of scan called an MRI scan – talk to the doctors if you think you have metal in your body.

Imaging (scans) allocation: You will then be randomly allocated to an imaging group by a computer, which has no information about you as an individual, i.e. allocation is by chance. You will have an equal chance of being allocated to either group, like the toss of a coin. There is a 50% chance that you will be put into the group in which the additional imaging tools will be used during your operation, in addition to the standard techniques, and a 50% chance that you will be in the group where the surgeon uses the present, standard imaging tools.

The random allocation is important because this way, we can test the different imaging tools fairly and nobody can influence into which group you are placed. If you enrol in the study, your healthcare and research teams will not be able to affect which imaging tools will get used in your operation and you will not be able to choose. You will not be aware into which arm of the study you have been allocated, just in case you answer the questionnaires differently based on the imaging used in your operation.

Please note: The design of this study has involved patients, their families and healthcare professionals, including brain surgeons, using their knowledge and experience at every stage of its development.

The FUTURE-GB study aims to find out if the new technologies do, or do not, improve the quality of life of those treated for a brain tumour. We need to know how you are, and your abilities during the study, before and after your operation. We are therefore asking everyone who agrees to take part to nominate a good friend/relative/partner to complete the same questionnaires at the same time as you. They will be asked the same questions as you are asked but they will be asked to answer what their opinion is of <u>your</u> health and abilities.

If you become unable to answer the questions at some point during the study, we would like to know the answers from your good friend/relative/partner to help us identify any changes in your quality of life up to that point in the study. It is helpful to have both assessments to be sure we know about any change in your health status during the study. This is why we would ask both you and your friend/relative/partner to complete the questionnaires throughout the study. The responses that you and your friend/relative/partner give will be used by the trial team to understand the impact of the new technologies on quality of life.
Note: All the data that you and your friend/relative/partner gives will be used by the trial team.

What technologies will be used in my operation?

If you are allocated to the standard techniques arm of the study, your surgeon will use the standard NHS imaging tools.

You will have a Magnetic Resonance Imaging (MRI) scan before your operation. This can be used during the surgery to help your surgeon identify where your brain tumour is located, and what brain structures are close by. This is a type of scan that uses strong magnetic fields to obtain detailed images

Plaha P, *et al. BMJ Open* 2022; 12:e064823. doi: 10.1136/bmjopen-2022-064823

LOCAL TRUST LOGO

of your brain and specifically your brain tumour. There is no risk of radiation exposure. All MRI scans that you will receive are received by all those with a brain tumour, anything seen on these scans will be acted upon as per local NHS Trust and national guidelines.

This is combined with use of a chemical called 5-aminolevulinic acid (5-ALA), which is a drink taken a few hours before surgery. This allows the tumour cells to light up pink, when a blue light is shone on them during surgery. This is known to help surgeons remove more of the brain tumour, as they are able to see better the edges of the tumour compared to the rest of the brain, making sure as much of the tumour is removed as is possible in each individual case.

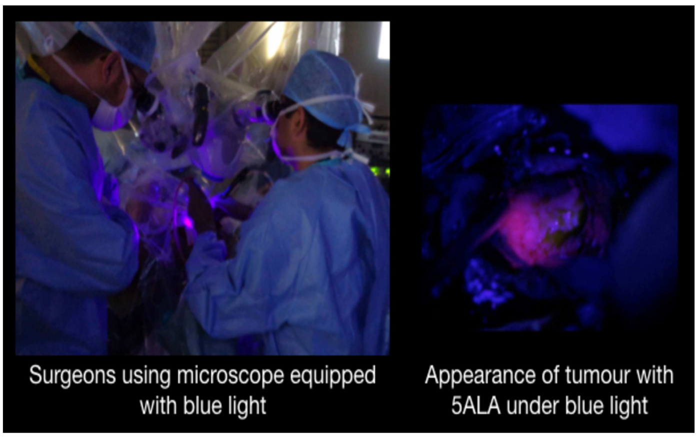

Surgeons using microscope equipped with blue light

Appearance of tumour with 5ALA under blue light

If you are allocated to the group that will use the additional imaging tools, your surgeon will undertake all of what is listed above, together with the additional imaging tools. You will have a slightly longer MRI scan (additional 5 minutes) and have the imaging outlined below also undertaken as part of your operation.

Diffusion Tensor Imaging (DTI) – this is an imaging tool that allows the surgeons to have a scan of all the nerve fibres which are involved in movement, speech etc. around a tumour. This means that when removing the tumour the surgeons potentially know, more accurately, where these are located specifically in your brain from this scan and so can avoid them. This scan is taken using an MRI machine – this is why your MRI scan would be 5 minutes longer.

Intraoperative Ultrasound – this is a technology whereby high frequency sound waves are used to create an image of the brain tumour during the operation. The ultrasound provides "live" pictures of your tumour as surgery progresses and tumour is removed. The surgeon can use this as many times as necessary during your surgery. The ultrasound is the same as that used to provide a picture of a baby inside a pregnant woman.

Both these imaging tools are safe and are used in brain tumour surgery already, but their benefit has not been formally assessed. There are no extra drugs or chemicals used for the additional imaging tools.

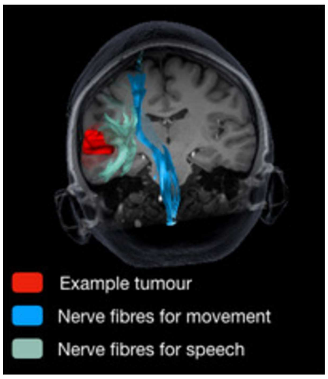

There are 2 companies supporting this study by providing machinery and software to sites if they do not already have the equipment needed for this study. The companies are called BrainLab and Medtronic they are supporting doctors in the UK in using the new technologies. Neither company will be able to influence the results of the study.

Further possible contacts: A researcher from the trial coordinating team may visit while you are having your operation so that we can check how the surgery is being undertaken. We will always check that you are happy for this to happen. If not, the researcher will not come into your operation. At the end of the study, we will report how well the treatments were delivered as it is important we fully understand this process.

LOCAL TRUST LOGO

What happens after my operation in FUTURE-GB?

If you take part in the study, we will not know whether the additional imaging tools are helpful or not until many months after your operation.

As part of the study you will be asked to complete some further questionnaires. The questionnaires ask about you, your health and activity, and your quality of life. We ask that you complete the questionnaires before you have surgery, when you leave hospital, at 6 and 12 weeks after surgery, and then every 3 months, for a maximum of 2 years. These time points are when you would usually be coming back to the hospital for routine NHS care, and it may be possible to complete them at these appointments. However, they will also be emailed to you for online completion. In addition, either when you are at the hospital for your outpatient appointments, or via telephone at those timepoints, a researcher will also ask you to complete a brain activity and a recall test, and talk to you about how you care for yourself, these will be the same tests and questions as before your operation.

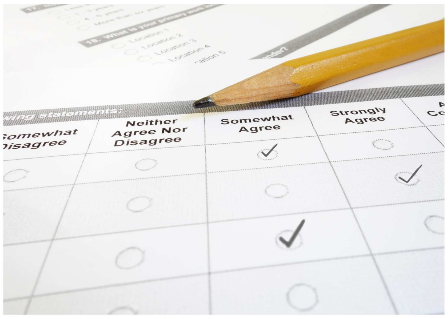

The questionnaires should take no more than 10 minutes to complete on paper/online, or over the telephone. The questionnaires are all completed online, but speak to your researcher if you want to take part but do not have access to the internet.

Please note, the care, any tests, further outpatient appointments and any other surgery or treatments will not be changed by you agreeing to take part in FUTURE-GB. Researchers will check your medical notes for up to 24 months after your operation to find out how you are getting on, and the trial team will use your details to send out the follow-up questionnaires.

Please note if you are deemed to lose capacity at any point during the study, you will not be asked to complete any further questionnaires.

What are the benefits and risks of taking part in the study?

For those that take part in the study, your operation will be conducted by the same surgeon/surgical team whom you have already met.

The information from this study we hope will answer the question:

Which imaging tools should be used by surgeons when removing a glioblastoma, to offer the highest chance of removing as much of the tumour as possible without causing functional problems, and therefore keeping a good quality of life?

We cannot promise the study will help you directly, but the information we get has the potential to be of benefit, potentially allowing more of your tumour to be removed safely.

The risks relating to the brain tumour surgery itself will be discussed with you in detail as part of the standard, routine consent for an operation. We don't think that being part of this study will change any of the risks of the operation but this is one of the things we be will be studying. The technologies will however add some time to the scan you have before your operation and your operation if you are put into the group where the new technologies are used.

We are undertaking this study because we want to find out whether the extra imaging tools provide a real benefit for people with brain tumours. These add significant costs to NHS treatment, and so we need to know if they are worth the extra cost.

LOCAL TRUST LOGO

People sometimes feel uncomfortable answering certain questions about their health, or may be unable to answer. If you, or the person you nominate to answer for you, feel uncomfortable at any point, then you do not have to answer the questions.

We are not able to pay travel expenses for you to attend your follow-up sessions, however any research questions asked will be as part of your routine out-patient follow-up appointments, via email, or over the telephone.

Who will know that I am taking part?

The only people who will know that you are taking part in this study are the members of the research team and the healthcare professionals involved in your care. You can tell anyone you would like to that you are taking part.

The only people in the University of Oxford who will have access to information that identifies you will be people who need to contact you to about the study, or review the data. The people who analyse the information will not be able to identify you and will not be able to find out your name or contact details. Also, your de-identified scans will be reviewed by members of the research team and the companies providing the extra imaging technologies. The images will be transferred using secure cloud servers, however, nothing that could identify you will be included.

Representatives from the sponsor, relevant regulatory organisations and [Insert local Trust] may also need access to monitor or audit the study to ensure that the research is complying with applicable regulations.

Paperwork that is completed by you, your nominee (friend/relative/partner), the research team, or the treating clinical team, will be sent securely to the study team managing the FUTURE-GB study that are based at the University of Oxford.

We will contact your GP (doctor) to tell them that you have agreed to take part in the FUTURE-GB study. However, we do not share with them anything you answer in your study questionnaires.

Will my details be kept confidential?

Yes. All information collected about you and from you and your nominee (friend/relative/partner) during the course of the research, including from your medical records, will be kept strictly confidential. Everyone who takes part in the study will be assigned a code number and all of the data relating to each person will be held on a computer database and will only be linked to that code number, and not to people's names or addresses. The study team will record into the study database your name, date of birth, NHS or CHI number, Hospital number, address, phone number, GP name and address, and your email address. These details will allow the central study team and the local teams to ensure they are collecting data on the correct person. Your email address will only be used to send you a copy of your consent form for your records and any follow-up questionnaires. This is also the reason your address will be kept on file – in case your questionnaires need to be posted to you to complete. Your phone number will be used by the researchers to call and ask you the questions about brain activity and recall if these cannot be completed at your outpatient appointment. Your NHS or CHI number will be used to check your status 24 months after agreeing to take part. Your GPs details will be used to send a letter to your GP informing them of you taking part in this study.

We will ask you for your permission for individuals from the University of Oxford and Imperial College London, and the regulatory authorities, to have access to your medical notes and data. This is in order that they may conduct checks on the study data that has been collected and to ensure all the study data has been completed correctly. We will also ask you for your permission to allow appropriate individuals from the NHS Trust that you are being approached at to also undertake this review.

LOCAL TRUST LOGO

At the end of the study, all of the data will be de-identified so that no-one can be identified. This de-identified data will be shared so that more researchers can gain a deeper understanding about patients who have had surgery for glioblastoma. It may be shared with other researchers around the world and with commercial organisations but this information will not identify you, and will not be combined with other information in a way that could identify you. The information will only be used for the purpose of healthcare research, and cannot be used to contact you, nor will it affect your care.

In line with what happens in the NHS, the only situation that confidentiality would need to be broken would be if you told a health professional or research team member of something that could result in harm to yourself or others.

What will happen to my data?

Research is carried out in the public interest. The University of Oxford, as Sponsor, is the data controller. This means that we, as University of Oxford researchers, are responsible for looking after your information as part of FUTURE-GB, and using it properly. We will use the minimum possible personally-identifiable information, and this will be kept for 12 months after the study has finished. Non-identifiable research data and any research documents with personal information, will be stored securely at the University of Oxford for a maximum of 5 years after the end of the study, as part of the research record.

We will be using information from you and your medical records in order to carry out this study. The [local NHS Trust name] will use your NHS number and contact details to get in touch with you, and to make sure that relevant study information is recorded from your care records. They will keep your identifiable information safely for 12 months after the study has finished. Consent forms and study documents held at [local NHS Trust name] will be archived securely, in accordance with their local procedures.

Data protection regulation provides you with control over your personal data and how it is used. When you agree to your information being used in research, however, some of those rights may be limited, as we need to manage your information in specific ways in order for the research to be reliable and accurate. Further information about your rights with respect to your personal data, is available at https://compliance.web.ox.ac.uk/individual-rights

You can find out more about how we use your information by contacting the FUTURE-GB study team on: futuregb@nds.ox.ac.uk.

What will happen if I don't want to carry on with the study?

You are free to withdraw from taking part in the study at any time without giving a reason. Please remember, it is your decision to take part. If you agree to take part now, but you change your mind during the study, this will not change the standard of care you receive from the NHS. If you were to decide to stop taking part in the study at any time, any data collected on you would be kept. You would not be contacted about the study again or have any further data collected about you from your medical records. If you withdraw or lose capacity please note we will not continue to contact your nominee (proxy).

What happens at the end of the study?

We will share the results with healthcare researchers and professionals to improve future patient care. Also, we will present them in research reports, at scientific conferences, and publish them in scientific journals, and publish them on the study website: futuregb.octru.ox.ac.uk.

Plaha P, et al. BMJ Open 2022; 12:e064823. doi: 10.1136/bmjopen-2022-064823

LOCAL TRUST LOGO

We will not include any data that could identify you in the results. If the funders of this research ask us to make the study data available for other researchers, we will first de-identify your information (i.e. we will take your name and other identifying details out) so that you cannot be identified.

Who is organising and funding the research?

The University of Oxford is the Sponsor and is organising this study. It is being conducted by a research team led by Prof. Puneet Plaha, Consultant Neurosurgeon at the Oxford University Hospitals NHS Foundation Trust and the University of Oxford, and Ms Sophie Camp and Prof. Dipankar Nandi (both Consultant Neurosurgeons at Imperial College Healthcare NHS Trust).

The National Institute of Health Research – Health Technology Assessment programme is funding the study. The funding for the NIHR comes from the UK Government.

Who has approved this study?

A panel of independent researchers and patient representatives, as well as a Research Ethics Committee (REC Reference 20/LO/0840) have reviewed and approved this study.

What if I have concerns?

The University of Oxford, as the study sponsor, has appropriate insurance in place in the unlikely event that you suffer any harm as a direct consequence of your participation in this study.

If you have any concerns or complaints about any aspect of the study, please contact the FUTURE-GB research team using the details below. You can also contact the University of Oxford Research Governance, Ethics & Assurance office on 01865 616480 or by email on ctrg@admin.ox.ac.uk.

If you would prefer to speak with someone who is not involved in the study, then please contact the Patient Advice and Liaison Service (PALS). PALS is a confidential NHS service that can provide you with support for any complaints or queries you have regarding the care you receive as an NHS patient. However, PALS cannot provide information about this research study.

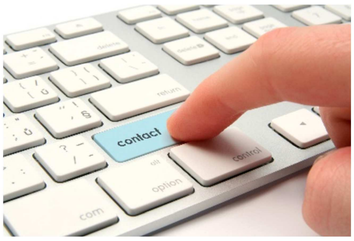

PALS phone number: <Insert local PALS number>

PALS email: <insert local PALS email address>

You can also contact your local clinical team directly:

<local PI/research team name and contact details>

If you have any questions about the study, please contact the FUTURE-GB team on:

Email: futuregb@nds.ox.ac.uk Telephone: 07917 101 649

Postal address: FUTURE-GB study, Botnar Research Centre, Nuffield Orthopaedic Centre, Windmill Road, Oxford, OX3 7LD.

Further information can be found on our study website – futuregb.octru.ox.ac.uk

Thank you for reading this information leaflet and considering taking part.

Plaha P, et al. BMJ Open 2022; 12:e064823. doi: 10.1136/bmjopen-2022-064823
