# Supplementary material for: FUTURE-GB: functional and ultrasound-guided resection of glioblastoma – a two-stage randomised control trial
Source: BMJ Open. 2022 Nov 15;12(11):e064823. doi: 10.1136/bmjopen-2022-064823 (PMC9668053; doi:10.1136/bmjopen-2022-064823)
Supplement: Supplementary data [file bmjopen-2022-064823supp010.pdf]

LOCAL TRUST LOGO

Functional and Ultrasound guided Resection of Glioblastoma – the FUTURE-GB study – This is for those that are asked to consider supporting a potential participant in the Future-GB study

Partner/Relative/Friend (Proxy) Information Leaflet

Invitation to join the FUTURE-GB study

You are receiving this information leaflet as a friend/relative/partner of yours has been approached to take part in this study. As part of the study they are asked to nominate a friend or relative who would be willing to answer some questionnaires about them (a 'proxy'). As the nominated person, we would like to invite you to take agree to part in a research study (also called a clinical trial), which for you will only involve answering questionnaires.

Before you decide whether to take part or not, it is important that you understand why we are doing this study and what it will involve.

Please take time to read the following information and talk to others about the study. If anything is unclear, or if you would like more information, please ask a member of the study team who will be happy to answer any questions.

What is the purpose of this study?

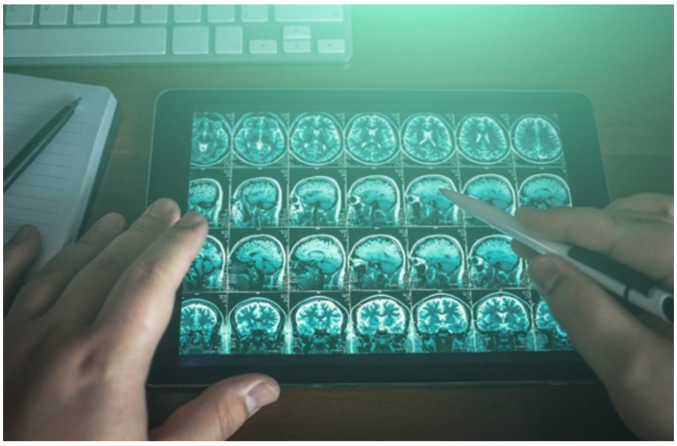

There are many different types of brain tumours. They can vary in how quickly they grow and what symptoms they cause. Studies have shown that when a brain tumour is growing quickly it is better to remove as much tumour as possible. Being able to do this without causing damage to the parts of the brain that are involved in things such as speaking and moving, surgeons need to be able to see clearly parts of the brain during surgery using accurate imaging. This has led to an increase in the use of imaging (such as ultrasound

Plaha P, et al. BMJ Open 2022; 12:e064823. doi: 10.1136/bmjopen-2022-064823

LOCAL TRUST LOGO

and MRI scans) during operations. However, we don't know if all the extra imaging tools do definitely make a difference.

We have been funded by the National Institute of Health Research (NIHR) which receives its funding from the UK Government, to find out whether some of these additional imaging tools available make a positive difference to quality of life for people with brain tumours who have surgery. We will also be looking to see if these imaging tools used during an operation mean people with a brain tumour:

- have a better quality of life?
- if it takes more/less time for their tumour to come back
- if they have more/fewer complications from the surgery

This study will attempt to find a definite answer, so that surgeons know which imaging tools they should use during an operation to enable as much tumour as possible to be removed safely, whilst minimising the risks of damaging brain function and hence affecting quality of life.

The imaging tools that will be used in this study are available across the NHS, and have been shown to be safe. However, no one knows if using them together will have a definite positive effect on outcome for those with a brain tumour. We would encourage you to read the Participant Information Sheet to find out more about the study.

Who is taking part and why have I been invited to take part?

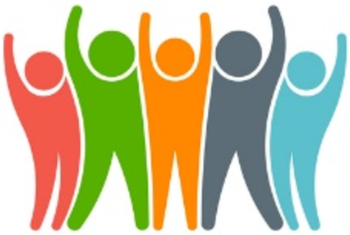

We are hoping to enrol a nominated relative/friend/partner (proxy) from each of the 357 people aged 18 -70 we plan to recruit to the trial, from approximately 15 neurosurgical centres in the UK who have agreed to take part in the FUTURE-GB study.

Specifically, the FUTURE-GB study aims to find out if the new technologies do or do not improve the quality of life of those treated for a brain tumour. We need to know how those taking part are and their abilities during the study, before and after their operation. If your friend/partner/relative becomes unable to answer the questionnaires at some point during the study, we would like to also have answers from you to help us identify any changes in their quality of life over the course of the study. However, if we need to use your answers instead of theirs – we can only do this if we know your answers at the start of the study, so that we can work out what changes have occurred. This is why we would ask both you and your friend/relative/partner to complete the questionnaires throughout the study. The responses that you and your friend/relative/partner give will be used by the trial team to understand the impact of the new technologies on quality of life

Do I have to take part in this study?

No, you are under no obligation to take part in the study. Deciding not to will not affect the treatment/care your friend/relative/partner receives. It is up to you to decide whether to take part or not. Please keep this leaflet and use it as it may to help you make your decision. If you decide to take part, you will be asked to sign a consent form.

FUNDED BY NIHR | National Institute for Health Research
OCTRU
SITU
UNIVERSITY OF OXFORD
Imperial College London

LOCAL TRUST LOGO

What will happen if I take part?

If you are happy to take part in this study, a researcher will contact you to complete questionnaires before your friend/relative/partner's operation, 5 days after their operation or when they leave hospital, 6 weeks after their operation and then every 3 months after your friend/relative/partner's operation for a maximum of 2 years.

Questionnaires take no more than 10 minutes to complete on paper/online, or over the telephone.

Please note if the person who has nominated you withdraws from the study or loses their capacity to consent this will complete your involvement with the study.

What are the benefits and risks of taking part in the study?

For those that take part in the study, your friend/relative/partner's operation will be conducted by the same surgeon/surgical team whom they have already seen.

The information from this study we hope will answer the question:

Which imaging tools should be used by surgeons when removing a glioblastoma, to offer the highest chance of removing as much of the tumour as possible without causing functional problems, and therefore keeping a good quality of life?

We cannot promise the study will help your friend/relative/partner directly, but the more information we collect, the greater the potential to be of benefit, as more of the tumour may be removed.

We are undertaking this study because we want to find out whether the extra imaging tools provide a real benefit for people with brain tumours. These add significant costs to NHS treatment, and so we need to know if they are worth the extra cost.

People sometimes feel uncomfortable answering certain questions about a person's health, or may be unable to answer. If you feel uncomfortable at any point, then you do not have to answer the questions.

Who will know that I am taking part?

The only people who will know that you are taking part in this study are the members of the research team and the person who nominated you. You can tell anyone you would like to that you are taking part.

The only people in the University of Oxford who will have access to information that identifies you will be people who need to contact you to about the study, or review the data. The people who analyse the information will not be able to identify you and will not be able to find out your name or contact details. Representatives from the sponsor, relevant regulatory organisations and [local NHS Trust name] may also need access to monitor or audit the study to ensure that the research is complying with applicable regulations.

Paperwork that is completed by you, your friend/relative/partner, the research team, or the treating clinical team, will be sent securely to the study team managing the FUTURE-GB study, who are based at the University of Oxford.

FUNDED BY NIHR | National Institute for Health Research OCTRU SITU UNIVERSITY OF OXFORD Imperial College London

Plaha P, *et al. BMJ Open* 2022; 12:e064823. doi: 10.1136/bmjopen-2022-064823

LOCAL TRUST LOGO

Will the details be kept confidential?

Yes. All information collected from you during the course of the research, will be kept strictly confidential. Everyone who takes part in the study will be assigned a code number and all of the data relating to each person will be held on a computer database and will only be linked to that code number, and not to people's names or addresses. The study team will record into the study database your name, age, relationship to the study participant, address, and your email address. These details will allow the central study team and the local teams to ensure they are collecting data on the correct person. Your email address will only be used to send you a copy of your consent form for your records and any follow-up questionnaires. This is also the reason your address will be kept on file – in case your questionnaires need to be posted to you to complete.

At the end of the study, all of the data will be de-identified so that no-one can be identified. This de-identified data will be shared so that more researchers can gain a deeper understanding about patients who have had surgery for glioblastoma. It may be shared with other researchers around the world and with commercial organisations but this information will not identify you, and will not be combined with other information in a way that could identify you. The information will only be used for the purpose of healthcare research, and cannot be used to contact you, nor will it affect your care.

In line with what happens in the NHS, the only situation that confidentiality would need to be broken would be if you told a health professional or research team member of something that could result in harm to yourself or others.

What will happen to my data?

Research is carried out in the public interest. The University of Oxford, as Sponsor, is the data controller. This means that we, as University of Oxford researchers, are responsible for looking after your information as part of FUTURE-GB, and using it properly. We will use the minimum possible personally-identifiable information, and this will be kept for 12 months after the study has finished. Non-identifiable research data and any research documents with personal information, will be stored securely at the University of Oxford for a maximum of 5 years after the end of the study, as part of the research record.

The local and central FUTURE-GB study team might use your contact details to get in touch with you. They will keep your identifiable information safely for 12 months after the study has finished. Consent forms and study documents held at [local NHS Trust name] will be archived securely, in accordance with their local procedures.

Data protection regulation provides you with control over your personal data and how it is used. When you agree to your information being used in research, however, some of those rights may be limited, as we need to manage your information in specific ways in order for the research to be reliable and accurate. Further information about your rights with respect to your personal data, is available at https://compliance.web.ox.ac.uk/individual-rights

You can find out more about how we use your information by contacting the FUTURE-GB study team on: futuregb@nds.ox.ac.uk.

FUNDED BY NIHR | National Institute for Health Research   OCTRU   SITU   UNIVERSITY OF OXFORD   Imperial College London

Plaha P, *et al. BMJ Open* 2022; 12:e064823. doi: 10.1136/bmjopen-2022-064823

LOCAL TRUST LOGO

Who is organising and funding the research?

The University of Oxford is the Sponsor and is organising this study. It is being conducted by a research team led by Professor Puneet Plaha, Consultant Neurosurgeon at the Oxford University Hospitals NHS Foundation Trust and the University of Oxford, and Miss Sophie Camp and Prof. Dipankar Nandi (both Consultant Neurosurgeons at Imperial College Healthcare NHS Trust, London).

The National Institute of Health Research – Health Technology Assessment programme is funding the study. The funding for the NIHR comes from the UK Government.

Who has approved this study?

A panel of independent researchers and patient representatives, as well as a Research Ethics Committee (REC Reference 20/LO/0840) have reviewed and approved this study.

What if I have concerns?

The University of Oxford, as the study sponsor, has appropriate insurance in place in the unlikely event that you suffer any harm as a direct consequence of your participation in this study.

If you have any concerns or complaints about any aspect of the study, please contact the FUTURE-GB research team using the details below. You can also contact the University of Oxford Research Governance, Ethics & Assurance office on 01865 616480 or by email on ctrg@admin.ox.ac.uk.

If you would prefer to speak with someone who is not involved in the study, then please contact the Patient Advice and Liaison Service (PALS). PALS is a confidential NHS service that can provide you with support for any complaints or queries you have regarding the care you receive as an NHS patient. However, PALS cannot provide information about this research study.

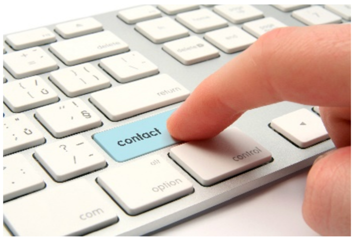

PALS phone number: <Insert local PALS number>

PALS email: <insert local PALS email address>

You can also contact your local clinical team directly:

<local PI/research team name and contact details>

If you have any questions about the study, please contact the FUTURE-GB team on:

Email: futuregb@nds.ox.ac.uk Telephone: 07917 101 649

Postal address: FUTURE-GB study, Botnar Research Centre, Nuffield Orthopaedic Centre, Windmill Road, Oxford, OX3 7LD.

Further information can be found on our study website – futuregb.octru.ox.ac.uk

Thank you for reading this information leaflet and considering taking part.

Plaha P, et al. BMJ Open 2022; 12:e064823. doi: 10.1136/bmjopen-2022-064823
