# Supplementary material for: FUTURE-GB: functional and ultrasound-guided resection of glioblastoma – a two-stage randomised control trial
Source: BMJ Open. 2022 Nov 15;12(11):e064823. doi: 10.1136/bmjopen-2022-064823 (PMC9668053; doi:10.1136/bmjopen-2022-064823)
Supplement: Supplementary data [file bmjopen-2022-064823supp011.pdf]

University of Oxford Logo

CONSENT FORM (Stage 1-IDEAL Phase)

FUTURE GB

Imperial Logo

If you agree,
please check box

1. I confirm that I have read and understood the Information Leaflet dated DDMon20YY version XX. I have had the opportunity to consider the information, ask questions and have had these answered satisfactorily.	
2. I understand that my participation is voluntary and that I am free to withdraw at any time without giving any reason, and without my medical care or legal rights being affected. After withdrawal from the study any data collection from databases will stop.	
3. I understand that relevant sections of my medical notes and data collected during the study may be looked at by individuals from the University of Oxford and Imperial College, London, from regulatory authorities and from the NHS Trusts, where it is relevant to me taking part in this research. I give permission for these individuals to have access to my records.	
4. I consent to the research team holding my contact details so that they can contact me about the study if required. I understand these details will be held securely and destroyed 12 months after the end of the study.	
5. I understand that the information held and maintained by NHS Digital / NHS Central Register may be used to help contact me or provide information about my health status over the next 12 months.<br>I understand and give permission for my NHS/CHI number to be used for this purpose.	
6. I agree to take part in the FUTURE-GB study.	
7. I agree that my operation may be observed for quality assurance purposes by a member of the FUTURE-GB study team. Yes / No	

Name of Participant **Date** **Signature**

Name of Person Taking Consent **Date** **Signature**

FUTUREGB_IDEAL_ICF_V5.0_21Apr2021.docx IRAS ID: 264482
Co- Investigators: Mr Puneet Plaha, Ms Sophie Camp and Prof Dipankar Nandi.
