# Supplementary material for: FUTURE-GB: functional and ultrasound-guided resection of glioblastoma – a two-stage randomised control trial
Source: BMJ Open. 2022 Nov 15;12(11):e064823. doi: 10.1136/bmjopen-2022-064823 (PMC9668053; doi:10.1136/bmjopen-2022-064823)
Supplement: Supplementary data [file bmjopen-2022-064823supp012.pdf]

University of Oxford Logo

Imperial Logo

# CONSENT FORM

FUTURE GB

If you agree,
**please check box**

| | |
|---|---|
| 1. I confirm that I have read and understood the Information Leaflet dated DDMon20YY version XX. I have had the opportunity to consider the information, ask questions and have had these answered satisfactorily. | |
| 2. I understand that my participation is voluntary and that I am free to withdraw at any time without giving any reason, and without my medical care or legal rights being affected. After withdrawal from the study any data collection from databases will stop. | |
| 3. I understand that relevant sections of my medical notes and data collected during the study may be looked at by individuals from the University of Oxford and Imperial College, London, from regulatory authorities and from the NHS Trusts, where it is relevant to me taking part in this research. I give permission for these individuals to have access to my records. | |
| 4. I consent to the research team holding my contact details so that they can contact me about the study. I understand these details will be held securely and destroyed 12 months after the end of the study. | |
| 5. I agree to my General Practitioner (GP) being informed of my participation in the study. | |
| 6. I understand that the information held and maintained by NHS Digital / NHS Central Register may be used to help contact me or provide information about my health status for the next 24 months. I understand and give permission for my NHS/CHI number to be used for this purpose. | |
| 7. If I complete any questionnaires online or via the telephone regarding the FUTURE-GB study, I agree for these to be passed to the hospital I was recruited at for this study. | |
| 8. I agree to take part in the FUTURE-GB study. | |
| 9. I nominate the following person to be my proxy and to answer questionnaires about me during my time in the FUTURE-GB study. They will no longer be asked to provide information about me if I withdraw from the study or lose capacity. Proxy name: (INSERT NAME HERE) | |
| 10. I agree that my operation may be observed for quality assurance purposes by a member of the FUTURE-GB study team. Yes / No | |

**Name of Participant** **Date** **Signature**

**Name of Person Taking Consent** **Date** **Signature**

FUTUREGB_ICF_V5.0_21Apr2021.docx IRAS ID: 264482
Co- Investigators: Prof Puneet Plaha, Ms Sophie Camp and Prof Dipankar Nandi.
