# Supplementary material for: FUTURE-GB: functional and ultrasound-guided resection of glioblastoma – a two-stage randomised control trial
Source: BMJ Open. 2022 Nov 15;12(11):e064823. doi: 10.1136/bmjopen-2022-064823 (PMC9668053; doi:10.1136/bmjopen-2022-064823)
Supplement: Supplementary data [file bmjopen-2022-064823supp013.pdf]

BMJ Publishing Group Limited (BMJ) disclaims all liability and responsibility arising from any reliance placed on this supplemental material which has been supplied by the author(s)

University of Oxford Logo

PROXY CONSENT FORM

Imperial Logo

	If you agree, **please check box**
1. I confirm that I have read and understood the Proxy Information Leaflet dated DDMon20YY version XX. I have had the opportunity to consider the information, ask questions and have had these answered satisfactorily.	
2. I understand that my participation is voluntary and that I am free to withdraw at any time without giving any reason, and without my relative/friend's medical care or legal rights being affected.	
3. I consent to the research team holding my contact details so that they can contact me about the study. I understand these details will be held securely and destroyed at the 12 months after the end of the study.	
4. I understand that as long as I feel able to I will complete regular questionnaires about the abilities and quality of life of the person I have been nominated to be the Proxy of. I understand if the person who nominated me withdraws or loses capacity – this will also end my participation.	
5. I agree to take part in the FUTURE-GB study as a Proxy Representative.	

Name of Participant: ______________________

Relationship to the participant: Partner ☐ Family member ☐ Carer ☐ Friend ☐
Other (please specify) ______________________

Approximately how much time do you spend with the FUTURE-GB participant per week?
- I am in contact with them daily ☐
- I am in contact with them every few days ☐
- I am in contact with them weekly ☐
- I am in contact with them every 2 weeks ☐
- I am in contact with them monthly ☐

Name of Proxy Representative | Signature: ______________ | Date: ______________

Name of Person Taking Consent | Signature: ______________ | Date: ______________

Deleted: FUTUREGB_ProxyICF_V3.0_10Jun2021_clean.docx

FUTUREGB_ProxyICF_V3.0_10Jun2021.docx IRAS ID: 264482
Co- Investigator: Prof Puneet Plaha, Ms Sophie Camp and Prof Dipankar Nandi.
