# Supplementary material for: FUTURE-GB: functional and ultrasound-guided resection of glioblastoma – a two-stage randomised control trial
Source: BMJ Open. 2022 Nov 15;12(11):e064823. doi: 10.1136/bmjopen-2022-064823 (PMC9668053; doi:10.1136/bmjopen-2022-064823)
Supplement: Author's manuscript [file bmjopen-2022-064823.draft_revisions.pdf]

BMJ Open is committed to open peer review. As part of this commitment we make the peer review history of every article we publish publicly available.

When an article is published we post the peer reviewers' comments and the authors' responses online. We also post the versions of the paper that were used during peer review. These are the versions that the peer review comments apply to.

The versions of the paper that follow are the versions that were submitted during the peer review process. They are not the versions of record or the final published versions. They should not be cited or distributed as the published version of this manuscript.

BMJ Open is an open access journal and the full, final, typeset and author-corrected version of record of the manuscript is available on our site with no access controls, subscription charges or pay-per-view fees (http://bmjopen.bmj.com).

If you have any questions on BMJ Open's open peer review process please email info.bmjopen@bmj.com

FUTURE-GB – Functional and Ultrasound guided Resection of Glioblastoma. A two-stage randomised control trial.

Journal:	*BMJ Open*
Manuscript ID	bmjopen-2022-064823
Article Type:	Protocol
Date Submitted by the Author:	02-Jun-2022
Complete List of Authors:	Plaha, Puneet; Oxford University Hospitals NHS Foundation Trust, Department of Neursurgery; University of Oxford, Nuffield Department of Surgical Sciences<br>Camp, Sophie; Imperial College Healthcare NHS Trust, Neurosurgery<br>Cook, Jonathan; University of Oxford Nuffield Department of Orthopaedics Rheumatology and Musculoskeletal Sciences, Oxford Clinical Trials Research Unit & Surgical Intervention Trials Unit<br>McCulloch, Peter; Oxford University, Nuffield Department of Surgical Sciences<br>Voets, Natalie; Oxford University, Nuffield Department of Clinical Neurosciences<br>Ma, Ruichong; Oxford University Hospitals NHS Foundation Trust, Department of Neurosurgery; University of Oxford, Nuffield Department of Surgical Sciences<br>Taphoorn, Martin J.B.; Leiden University Medical Center; Haaglanden Medical Center Bronovo<br>Dirven, Linda; Leiden University Medical Center<br>Grech-Sollars, Matthew; Royal Surrey NHS Foundation Trust; Imperial College London<br>Watts, Colin; University of Birmingham, Institute of Cancer and Genomic Studies; University Hospitals Birmingham NHS Foundation Trust, Department of Neurosurgery<br>Bulbeck, Helen; Brainstrust<br>Jenkinson, Michael; University of Liverpool; Walton Centre for Neurology and Neurosurgery<br>Williams, Matthew; Imperial College Healthcare NHS Trust<br>Lim, Adrian; Imperial College London<br>Dixon, Luke; Imperial College Healthcare NHS Trust, Neuroradiology<br>Price, Stephen; Cambridge University, Neurosurgery Division, Dept. Clinical Neurosciences<br>Ashkan, Keyoumars; King's College Hospital<br>Apostolopoulos, Vasileios; Oxford University Hospitals NHS Foundation Trust, Department of Neursurgery<br>Barber, Vicki; University of Oxford Nuffield Department of Orthopaedics Rheumatology and Musculoskeletal Sciences, Oxford Clinical Trials Research Unit & Surgical Intervention Trials Unit<br>GB, FUTURE; Oxford University<br>Nandi, Dipankar; Imperial College Healthcare NHS Trust, Neurosurgery; Imperial College London
Keywords:	Neurosurgery < SURGERY, Clinical trials < THERAPEUTICS, SURGERY

FUTURE-GB – Functional and Ultrasound guided Resection of Glioblastoma. A two-stage randomised control trial.

Puneet Plaha[1,2], Sophie Camp[3], Jonathan A Cook[4], Peter McCulloch[2], Natalie Voets[5], Ruichong Ma[1,2], Martin Taphoorn[6,7], Linda Dirven[6], Matthew Grech-Sollars[8,9], Colin Watts[10,11], Helen Bulbeck[12], Michael D Jenkinson[13], Matt Williams[3], Adrian Lim[9], Luke Dixon[3], Stephen Price[14], Keyoumars Ashkan[15], Vasileios Apostolopoulos[1], Vicki S Barber[4], FUTURE-GB Collaborators, Dipankar Nandi[3,9]

[1]Department of Neurosurgery, Oxford University Hospitals NHS Foundation Trust, Oxford, UK
[2]Nuffield Department of Surgical Sciences, University of Oxford, UK
[3]Imperial College Healthcare NHS Trust, London, UK
[4]Oxford Clinical Trials Research Unit & Surgical Intervention Trials Unit, Nuffield Department of Orthopaedics Rheumatology and Musculoskeletal Sciences, University of Oxford, UK
[5]Nuffield Department of Clinical Neurosciences, University of Oxford, UK
[6]Department of Neurology, Leiden University Medical Center, Leiden, Netherlands
[7]Department of Neurology, Haaglanden Medical Center, The Hague, Netherlands
[8]Royal Surrey NHS Foundation Trust, Guildford, Surrey, UK
[9]Imperial College London, London, UK
[10]Institute of Cancer and Genomic Sciences, University of Birmingham, UK
[11] Department of Neurosurgery, University Hospitals Birmingham Foundation Trust, UK
[12]brainstrust = the brain cancer people, UK
[13]University of Liverpool, Liverpool, UK
[14]Department of Neurosurgery, Cambridge University Hospitals NHS Foundation Trust, Cambridge, UK
[15]Deparment of Neurosurgery, King's College Hospital NHS Foundation Trust, London, UK

FUTURE-GB collaborators:
Giles Critchley (Brighton), George Eralil, Kathrin Whitehouse (Cardiff), Anna Solth (Dundee), Paul M Brennan (Edinburgh), Chittoor Rajaraman, Shailendra Achawal (Hull), Anil Varma (Middlesborough), Robert Corns, Ganamurthy Sivakumar (Leeds), Farouk Olubajo (Liverpool), Edward McKintosh, Grainne McKenna and Dimitrios Paraskevopoulos (Barts Health), Neil Barua (Bristol), James Palmer (Plymouth), Stuart Smith (Nottingham), Paul Grundy (Southampton), Erminia Albanese, Huan Chan (Stoke-on-Trent), Damian Holliman (Newcastle), Jose Lavrador, Maria Velicu (King's College Hospital)

Corresponding Author: Puneet Plaha (puneet.plaha@ouh.nhs.uk)

Word Count: 3977 excluding abstract, references, tables and figure legends.

For peer review only

Abstract

Introduction

Surgery remains the mainstay for treatment of primary glioblastoma, followed by radiotherapy and chemotherapy. Current standard of care during surgery involves the intraoperative use of image-guidance and 5-ALA. There are multiple other surgical adjuncts available to the neuro-oncology surgeon. However, access to, and utilisation of these varies widely in UK practice, with limited evidence of their utility. The aim of this trial is to investigate whether the addition of Diffusion Tensor Imaging (DTI) and intraoperative ultrasound (iUS) to standard of care surgery (Intra operative Neuronavigation and 5-ALA) impacts on deterioration free survival (DFS).

Methods and Analysis

This is a two-stage randomised control trial (RCT) consisting of an initial non-randomised cohort study based on the principles of the IDEAL Stage-IIb format, followed by a statistically powered randomised trial comparing the addition of DTI and iUS to standard of care surgery. A total of 357 patients will be recruited for the RCT. The primary outcome is DFS, defined as the time to either 10-point deterioration in HRQoL scores from baseline, without subsequent reversal, progressive disease, or death.

Ethics and Dissemination

The trial was registered in the Integrated Research Application System (Ref: 264482) and approved by a UK research and ethics committee (Ref: 20/LO/0840). Results will be published in a peer reviewed journal. Further dissemination to participants, patient groups and the wider medical community will utilize a range of approaches to maximize impact.

Registration details

ISRCTN: 38834571

Keywords

Glioblastoma, IDEAL, randomised control trial, DTI, intraoperative ultrasound

For peer review only

Article Summary

Strengths of trial:

- This is a randomised control trial comparing the quality of life of glioblastoma patients undergoing standard of care surgery (Intraoperative neuronavigation and 5-ALA) vs surgery with the addition of Diffusion Tensor imaging (DTI) and intraoperative ultrasound (iUS).

- To ensure standardisation and quality control of delivery of the DTI and iUS in the randomised trial, sites will be required to enter a minimum number of patients into an initial IDEAL Stage-IIB study prior to commencing recruitment to the randomised trial.

- Patient public involvement (PPI) determined the primary outcome measure of deterioration free survival (comprising a decline in health-related quality of life, disease progression or death), rather than overall survival. DFS is considered by patients to be most pertinent.

Limitations of the trial:

- The trial recruits patients aged 18-70years who can undergo surgery to maximally resect their glioblastoma.

- There is variability of the intraoperative ultrasound machines used by trial sites (sites use machines they are familiar with). However, this reflects real world iUS usage.

For peer review only

Introduction

Glioblastoma (GB) is the most frequent and aggressive form of primary brain cancer, with an incidence of 4.64/100,000persons/year in the UK[1]. Prognosis remains extremely poor with median survival of approximately 15 months[2], and as the tumour grows, patients experience a progressive decline in health-related quality of life (HRQoL), and caregivers report high levels of distress and carer burden[3]. Resistance to treatment leads to poor survival, with high costs to the patient, relatives, society, and the economy[4,5]. Although primary brain tumours represent only 3% of all cancers, a brain tumour reduces life expectancy by an average of 20years, the highest of any cancer, and accounts for more average years of life lost than any other cancer[4,5]. GB affects adults in their economic prime, and is a leading cause of death in those under 40years, costing the economy £578M per year[4,5]. To date, there has been little progress in improving outcome including quality of life, with many trials failing to show an effect[6].

Surgery is the mainstay of treatment for GB, but optimum surgical technologies remain unclear. Surgery to resect GB is integral to maximum first line treatment, with a greater impact on survival than non-operative treatments (radiotherapy and chemotherapy)[7]. It improves symptom control, reduces dependence on dexamethasone, and increases progression free (PFS) and overall survival (OS)[8,9]. However, maximising the extent of surgical resection must be balanced against the potential risk of causing neurological deficit, and hence impacting negatively on a patient's ability to tolerate adjuvant treatments.

The desire to achieve a safe, maximal resection, particularly in eloquent regions, has led to an increase in the use of intraoperative imaging. This attempts to eliminate the error produced by brain shift, an inherent problem in navigation systems based on preoperative imaging[6], to demonstrate residual tumour at operation, and to visualise accurately relevant white matter tracts and tumour margins. Two technologies that facilitate surgical resection intraoperatively are iUS and DTI.

1. iUS accommodates for brain shift if it is linked to neuronavigation systems, allowing the surgeon to track tumour resection in real time. iUS permits multiple, real time image acquisitions, and, potentially, if navigated, at each stage, comparison with the preoperative MRI navigation sequence, to evaluate brain shift and residual disease. iUS minimally augments operative time[6], allowing precise visualization of tumour resection. It is user friendly, widely available, and a pragmatic and cost-effective alternative to intraoperative MRI, which is prohibitively expensive for many UK units. iUS, and more recently navigated iUS, has a long history in brain tumour surgery[10,11], facilitating/extending resection[12–16], and improving survival[17]. It has also been evaluated with respect to histology[18,19]. However, there is a learning curve, and image interpretation, especially during resection, can be challenging[10]. iUS demonstrates residual tumour in real time. Indeed, it has been reported that navigated iUS and 5-ALA provide different information of tumour extent, and when combined, enhance extent of resection[20]. Despite this, there are no randomised trials assessing its efficacy.

2. DTI is a special magnetic resonance imaging (MRI) technique that can identify the location of white matter nerve tracts important for speech/language/visual/motor

For peer review only

functions. The location of white matter fibre pathways is the most frequent reason why surgery is halted early, to avoid compromising patient function[21]. DTI is the only method available to visualise functionally important white matter tracts in the vicinity of a tumour before surgery and can be fused with standard intraoperative navigation systems to enable visualisation of the spatial location of the tracts during surgery, allowing removal of tumour in close proximity. The usefulness of DTI in brain tumour surgery has recently been reviewed[20]. Intraoperative visualisation of DTI is reported to contribute to maximising safe resection[21–23], reducing visual field deficits[24], and predicting long term language problems after surgery[25]. A single centre randomised control trial (RCT), comparing DTI vs no-DTI, showed that DTI led to significantly better gross total resection (GTR) rates, a lower risk of movement loss, and improved life expectancy[27]. Furthermore, DTI-informed awake surgery reduced the occurrence and severity of behavioural problems postoperatively, leading to faster recovery, and shorter hospital stay[28]. DTI requires the collection of additional MRI data, specialist software for analysis, and detailed knowledge of white matter anatomy and function. In addition, tract visualisation may be restricted where there is peritumoural oedema. As a result, there is only limited data available on the sensitivity and specificity of DTI in GB surgery, particularly with reference to its value as an intraoperative tool and in predicting DFS.

There are wide variations of surgical standard of care across the UK. A survey of all 24 adult UK neurosurgical centres (telephone and email survey conducted in 2018 by the Oxford researchers), showed wide variation in the use of technologies employed during GB resection. Whilst all centres employ standard neuronavigation and 5-ALA, only 75% have access to iUS, 62% to DTI, and 16% to an intraoperative MRI scanner. It remains unclear which technologies should be employed intraoperatively, without worsening neurological function. Indeed, most of these technologies are not regularly used for tumour resection, with surgeons unclear of the efficacy of each, and what is the optimum combination. A recent Cochrane review emphasized the lack of high-quality evidence to support the use of any specific intraoperative imaging technology[29]. The National Institute for Health and Care Excellence (NICE) guidance[3] has suggested that the available range of intraoperative technologies are considered, as appropriate, in addition to standard techniques, for tumour resection.

The Functional and UlTrasound gUided Resection of GlioBlastoma – FUTURE-GB trial was developed in collaboration with the Society of British Neurosurgeons (SBNS) and multiple GB patient advocate groups to try and address some of the deficits in knowledge regarding the utility of addition surgical adjuncts. FUTURE-GB aims to evaluate the impact of DTI and iUS in addition to standard of care techniques with a view to providing high-quality evidence to shape standard practice in the future.

Methods and Analysis

Trial design and setting

For peer review only - http://bmjopen.bmj.com/site/about/guidelines.xhtml

FUTURE-GB is a two-stage trial (figure 1). Patient and public involvement (PPI) actively informed the rationale, design and development of the protocol and patient facing materials of the trial.

Stage-1: non-randomised multicentre learning and evaluation stage (IDEAL Stage IIB trial)
Stage-1 is a non-randomised, multicentre learning and harmonisation stage in which quality control measures and mentoring will be employed, to improve and evaluate standards of practice based on the principles of an IDEAL Framework Stage-IIB study[31,32]. It will evaluate standard care surgery with the addition of DTI imaging and the ultrasound imaging during the operation. This will ensure that the surgeons using the technologies to be employed in the RCT demonstrate acceptable expertise in delivering the new approach prior to proceeding with the randomisation stage. This stage ensures standardisation of the use of the technologies across all trial centres by expert mentoring, and will evaluate quality of delivery, including monitoring of the learning curve for the group as a whole.

Stage-1 is divided into 3 components:

1. Pre-trial Webinar
2. IDEAL Stage-IIB (Quality assurance, Mentoring and Trial centres evaluation)
3. End of Stage-1, Pre-Stage-2 RCT, each participating centre will have a data workflow review with the Lead Investigators to review the cases completed in Stage-1.

The IDEAL Framework Stage-IIB trial will comprise the following:

- Mentoring for local site surgeons.
- Quality assurance of operative procedure.

Mentoring by the CI and Lead Investigators will be provided through visits to participating centres and frequent meetings, together with a helpline for individual advice sessions from the CI and Lead Investigators and co-applicants, as appropriate. Neurosurgeons will contribute data to ensure standardisation of the protocol and acceptable expertise in delivering the new approach (supplementary files1,2). This will be evaluated using the following metrics: operation length; successful use of DTI neuronavigation and iUS to achieve maximal safe tumour resection without major neurological deficit; and extent of tumour resection assessed on postoperative MRI scan. The number of cases required for this may vary but is expected to be small (up to 5 cases) as most surgeons are already familiar with the component techniques and are not anticipated to require substantial assessment. Ensuring all participating surgeons are ready to take part will minimize performance bias in Stage-2 and ensure standardisation of intraoperative technique.

Stage-2: prospective, Stage-III, multicentre RCT with internal pilot
This is a parallel group two arm, multicentre, RCT. Patients who agree to take part will be allocated by chance. The trial will enrol 357 newly diagnosed patients with GB and will randomly allocate them to receive either surgical resection with standard methods without US and DTI, or this surgery with the addition of US and DTI, as well as standard tools. Patients will not know into which group they have been placed, nor will the research team assessing them before and after surgery. Patients will be recruited from at least 15 NHS hospitals that routinely undertake GB surgery and have access to these tools. The trial will be embedded within existing care pathways. After agreeing to take part, participants will be

asked to complete questionnaires (supplementary files3-7) about their health-related quality of life (HRQoL, reflecting symptoms as well as physical, emotional and psychological functioning. They will also have a brief physical and cognitive/functional assessment before their surgery. Afterwards, the questionnaires and assessments will be repeated, before leaving hospital, and at three monthly intervals until 24months after randomisation. These will be combined with planned hospital visits. OS will also be recorded. See Figure 1 for a Flowchart of the trial.

Population: 357 participants with GB suitable for maximal, safe resective surgery (attempted GTR of all enhancing tumour), as agreed at the local neuro-oncology Multi-Disciplinary Team (MDT) meeting.

Intervention: standard care surgery (neuronavigation based on preoperative imaging and intraoperative use of 5-ALA) with the addition of DTI neuronavigation and iUS.

Control: standard care surgery (neuronavigation based on preoperative imaging and intraoperative use of 5-ALA)

Outcome: Deterioration Free Survival, defined as the time to a 10-point deterioration in HRQoL scores from baseline, without subsequent 10-point improvement in scores compared with baseline; or progressive disease; or death in the absence of previous definitive deterioration before the next assessment. HRQoL is measured with the EORTC QLQ-C30 and QLQ-BN20 questionnaires.

Setting: At least 15 UK NHS Trusts undertaking GB surgery

Eligibility

Patients aged 18-70years with a primary GB tumour which is deemed maximally resectable (attempted GTR of all enhancing tumour) by the local neuro-oncology MDT meeting, will be potentially suitable for inclusion in the trial.

Inclusion Criteria

- Age 18-70years
- Neuro-oncology MDT decision that the imaging shows a primary GB tumour which is maximally resectable (attempted GTR of all contrast-enhancing tumour)
- Patient is suitable for concomintant adjuvant radiotherapy and Temozolomide (TMZ) chemotherapy followed by adjuvant TMZ at the time of MDT decision
- Able to receive 5-ALA
- Willing and able to give informed consent
- Able to complete trial questionnaires, this may be with support where English is not their first language (where compatible with the validation of questionnaires) (Stage-2 only)
- Able to provide a proxy who is willing to complete questionnaires as requested (Stage-2 only).

Exclusion Criteria

The participant may not enter the trial if any of the following apply:

For peer review only

- Midline/basal ganglia/cerebellum/brainstem GB
- Multifocal GB
- Recurrent GB
- Suspected secondary GB
- Contraindication to MRI

Proxy Inclusion (Stage-2 only)

Although it is widely recognised in HRQoL research that an individual may rate aspects of their functioning and well-being differently from how another person might, even if that person is close to the individual (e.g. carer, partner etc.), we will ask proxies to also rate HRQoL aspects of patients during the RCT.

The proxy/participant assessment is particularly important in cases where the patients are not able to complete the questionnaires, for example if have disease progression, or if their condition is too poor. These proxy measures can be used as substitute data in case the patient's rating of their HRQoL is lacking. When a participant dies, loses capacity, or withdraws from the trial – this will also automatically cease the proxy's involvement in the trial.

Inclusion Criteria for proxy

- Age 18-75years
- Nominated by an individual who has consented to participate in Stage-2
- Willing and able to give informed consent
- Able to understand written English to enable completion of trial questionnaires

Recruitment

Recruitment into the trial will be undertaken in two phases in conjunction with the separate stages of the trial. There will be a separate Patient Information Sheet and Consent Form for patients entering Stage-1 (IDEAL IIb) and Stage-2 (RCT) (supplementary files8-13). The stages are sequential at participating sites and the stages cannot be recruited to in parallel.

All potentially eligible participants will have the trial mentioned at the same time the options regarding their surgery are discussed. Depending upon the site, the resources available, and most importantly how the participant is dealing with their diagnosis, the recruitment process and approach may vary across and within sites. Potential participants may straight away be provided with the trial participant information sheet and asked to consider the trial, and that a member of the local research team will contact them. It may be the case that individuals are asked if it would be acceptable for their name to be passed to the research team who will make contact at a later timepoint, or potential participants may be given the participant information sheet and asked to call the number on it if they wish to find out more about the trial.

Randomisation

Randomisation of patients will only occur in Stage-2 of the trial. Every centre and each participating surgeon will offer surgery under both arms of the trial. Randomisation will be via the web-based service provided by OCTRU, using the method of minimisation. The minimisation factors will be trial site, age (≤55yrs or >55yrs), expected surgery status (under

For peer review only

general anaesthesia or awake), and eloquence of tumour location (non-eloquent or eloquent).

Participants will be randomised on a 1:1 basis, after having given written consent; however, they will remain blinded as to which arm of the trial they have been allocated. The local clinical team at site will receive an email from the randomisation system detailing the arm of the trial to which a participant has been randomised. Randomisation must occur before the pre-operative imaging takes place so that the assigned trial pre-operative imaging can be undertaken.

Pre and post randomisation withdrawals

Participants may decline to continue to take part in the trial at any time without prejudice. A decision not to participate or withdraw will not affect the standard of care the patient receives. Once withdrawn, the patient will be advised to discuss their further care plan with their surgeon. On withdrawal of the patient, any data collected up until the time of withdrawal will be retained by the research team and included in the final analysis.

Blinding

Stage-1 is not blinded; the participants will be receiving all the technologies during their surgery.

In Stage-2, the participant will be blinded to the allocation (intervention or control arm), and the treating clinician will be aware of the need to perform the surgery with the intraoperative technologies as allocated. In addition to the participant, the radiologist (reviewing the postoperative MRI) will be blinded to the trial arm. Given this, only on the operation CRF will data of the allocation be included.

Trial treatments

All participants will undergo surgery for removal of their GB. The choice of anaesthesia will be left to the discretion of the treating surgeon/anaesthetist/patient as per their normal practice and preference.

The trial will compare two imaging techniques for imaging the tumour. Participants will be randomised to either:

- Standard care surgery (neuronavigation based on preoperative imaging and intraoperative use of 5-ALA) (Control arm)
- Standard care surgery (neuronavigation based on preoperative imaging and intraoperative use of 5-ALA) **AND** of DTI neuronavigation and iUS (Intervention arm)

Objectives and Outcome Measures

Objectives and outcome measures are summarised in table 1.

For peer review only

Stage 1	Objectives	Outcome Measures	Timepoint(s)
**Primary** Outcomes	To demonstrate the feasibility of using DTI and iUS* in addition to standard of care (neuronavigation based on preoperative MRI and intraoperative use of 5-ALA) for neurosurgery (at selected UK NHS hospitals).	1. Operation length<br>2. Successful use of DTI neuronavigation and iUS* to achieve maximal safe tumour resection without major neurological deficit<br>3. Extent of tumour resection assessed on postoperative MRI scan.<br>4. Surgical Complication and Serious Adverse Events	Hospital discharge and 6 months post-op.
**Stage 2**	**Objectives**	**Outcome Measures**	**Timepoint(s)**
**Primary** Outcomes	To assess whether additional intraoperative imaging (DTI and iUS*) to standard of care (neuronavigation based on preoperative MRI scan and intraoperative 5-ALA) improves Deterioration Free Survival (DFS) (Where deterioration relates to global health status only)	Composite of global health status domain of the QLQ-C30 questionnaire, Progression Free Survival (PFS) and Overall Survival (OS) with an event defined as either deterioration, progression or death.	To be recorded at baseline; 6wks post-op., 3mths post-op., and then 3mthly up to 24 months
**Secondary** Outcomes	To assess whether additional intraoperative imaging (DTI and iUS*) to standard of care (Neuronavigation and intraoperative 5-ALA) improves DFS where deterioration relates to physical functioning, social functioning from the QLQ-C30, and motor dysfunction and communication deficit	4 composites using the respective domain of QLQ-C30 (physical functioning and social functioning) and BN20 (motor dysfunction and communication deficit) combined with PFS and OS.	To be recorded at baseline; 6wks post-op., 3mths post-op., and then 3mthly up to 24 months
**Secondary** Outcomes	To assess whether additional intraoperative imaging (DTI and iUS*) to standard of care (Neuronavigation and intraoperative 5-ALA) improves time to deterioration	Defined similar to DFS with the exception that progression is excluded as an event (i.e. only deterioration or death are considered). There will be five time to deterioration outcomes, one for each of the domains utilised in the primary and secondary DFS outcomes, used in turn to define deterioration.	To be recorded at 6wks post-op., 3mths post-op., and then 3mthly up to 24 months
**Secondary** Outcomes	To assess whether additional intraoperative imaging (DTI and IUS*) to standard of care (Neuronavigation and intraoperative 5-ALA) improves Overall Survival (OS)	OS (time from randomisation to death or trial closure)	To be recorded at 24 months
**Secondary** Outcomes	To assess whether additional intraoperative imaging (DTI and iUS*) to standard of care (Neuronavigation and intraoperative 5-ALA) improves Progression Free Survival (PFS)	PFS (time from randomisation to radiological tumour progression on imaging, as agreed in local MDT	MRI at 6 months post-op., and then 3mthly up to 24 months or an MRI performed outside protocol if patient is symptomatic
**Secondary** Outcomes	To assess whether additional intraoperative imaging (DTI and iUS*) to standard of care (Neuronavigation and intraoperative 5-ALA) improves the extent of tumour resection	Extent of resection as % of pre-operative tumour volume on postoperative contrast enhanced MRI	Post-operative review

For peer review only

**Secondary** Outcomes	To assess whether additional intraoperative imaging (DTI and iUS*) to standard of care (Neuronavigation and intraoperative 5-ALA) improves the incidence of surgical complications	Number and type of surgical complications	To be recorded at 5 days post-op, or discharge date (whichever is soonest); 6wks post-op., 3mths post-op., and then 3mthly up to 24 months
**Secondary** Outcomes	To assess whether additional intraoperative imaging (DTI and iUS*) to standard of care (Neuronavigation and intraoperative 5-ALA) improves the number of patients eligible for adjuvant treatment following surgery	Number of patients eligible for adjuvant treatment	3mths post-op.
**Secondary** Outcomes	To assess whether additional intraoperative imaging (DTI and iUS*) to standard of care (Neuronavigation and intraoperative 5-ALA) improves functional outcome postoperatively	WHO performance status<br>mini-MoCA (Montreal Version) Barthel Index<br>MRC grading of power in all 4 limbs	To be recorded at baseline, 5 days post-op., or discharge date (whichever is soonest); 6wks post-op., 3mths post-op., and then 3mthly up to 24 months.<br>(MRC grading to be assessed at baseline and 5 days post-op., or discharge date only)
**Secondary** Outcomes	Assess the correlation of proxy to participant classification assessment of quality of life	At a minimum, answers to questions 29 and 30 of the QLQ-C30. Ideally answers will be provided to all of the QLQ-C30 and BN20.	Baseline, 6wks post-op., 3mths post-op., and then 3mthly up to 24 months. Proxy will not complete questionnaires when participant stops completing them.
**Tertiary** Mechanistic Study Objectives – on a sub set of participants –	To assess the sensitivity and specificity of the anatomico-spatial location of DTI fibre tracts compared with intraoperative direct electrical stimulation/behavioural change without stimulation but related to adjacent white fibre tract in patients undergoing awake surgery, or motor evoked potential changes in patients undergoing surgery.	Sensitivity and specificity calculation using pre and post-surgery MRI images	Analysis will be undertaken post-surgery.
**Tertiary** Mechanistic Study Objectives – on a sub set of participants –	To assess the sensitivity and specificity of iUS* to identify the tumour boundary when compared with 5-ALA, navigated biopsies will be taken from tumour boundary tissue planned for resection.	Intra operative iUS* images and post-operative MRI scans and Intraoperative biopsy samples	Analysis will be undertaken post-surgery when biopsy results are available.

*if NiUS available, it is to be used

Table 1. Objectives and outcome measures for the trial.

End of trial

The end of the trial will be defined as the collection/receipt of the last follow-up questionnaire from the last participant and all data cleaning has been completed.

For peer review only

Statistical Methods
Full details of the statistical analysis will be detailed in a separate statistical analysis plan which will be drafted early in the trial and will be finalised after input from the TSC and DSMC. A summary of the planned statistical analysis is included here.

The analysis of the primary outcome will be a time-to-event analysis using a mixed effect Cox proportional hazard regression model. Minimisation factors (age, anticipated patient operative state and tumour location), radiotherapy, and MethylGuanine-DNA MethylTransferase (MGMT) status will be adjusted for as fixed effects. Centre will be included as a random effect.

The assumption of proportional hazard for the Cox model will be examined. If the proportional hazard assumption is not met, parametric survival analysis, such as the accelerated failure time method will be considered. A sensitivity analysis will look at the impact of adjusting for surgeon instead of centre. Secondary analysis will explore the influence of progression as an event by assessing DFS minus disease progression as an event. An unadjusted comparison using a log-rank test will also be carried out. Kaplan-Meier curves will also be generated. Secondary time-to-event outcomes (e.g. OS) will be analysed in a similar manner.

HRQoL amongst survivors will be quantified without a formal statistical comparison between treatment groups.

There are multiple factors that may influence how a patient rates their level of HRQoL, which may be related to factors other than the intervention. However, by using a randomized trial design, it is assumed that patients in both treatment arms are comparable on all aspects, both measured (e.g. age, performance status) and unmeasured (e.g. mood, coping strategy, personality). This means that the impact of the psychological state on the evaluation of HRQoL is treated as similar for the two trial arms. Thus, the trial will be able to measure whether the experimental intervention has an impact on HRQoL when compared to patients receiving standard treatment.

Sample Size Determination for IDEAL Framework IIB Trial (Stage-1)
There is no formal sample size for the IDEAL trial. Participants will be recruited at each centre, the number of cases required from each centre will vary depending upon caseload numbers and the number of neurosurgeons but is expected to be small for most sites (5), as the participating centres are already familiar with the component techniques.

Sample Size Determination for the RCT (Stage-2)
The sample size is based on a HRQoL aspect included in the primary outcome DFS, i.e. the global health status domain in the EORTC QLQ-C30 questionnaire version 3.0, and achieving a statistical power of 90% for the primary analysis (see below) with 2-sided significance level of 5%. Assuming a Hazard Ratio (HR) of 0.7, median DFS survival time of 5 months in the control arm, 24 months follow-up on all participants and allowing for 5% loss to follow-up occurring by month 3, this yields an overall target of 357 participants (178/179 per arm; 335 events overall) (Stata “artsurv”; www.stata.com). In a recent trial, the mean survival time of global health status DFS was 6 months in the standard treatment arm (surgical resection

For peer review only - http://bmjopen.bmj.com/site/about/guidelines.xhtml

with standard radiotherapy and chemotherapy)[33]. Additionally, the observed HR was 0.64, 95% CI (0.56, 0.74) for the DFS measures in this trial suggesting that a HR of 0.7 as assumed above is a plausible magnitude of effect to be observed for this population[34]. It would also be one which would be considered important to clinicians and patients given the definition of a DFS event (death, progression or a patient anchor determined clinically meaningful deterioration of 10 points). For key secondary outcomes (i.e. the other four DFS outcomes, PFS and OS) there is over 80% power for this size of trial, assuming a median OS of 6-9, 7 and 15months respectively in the control arm, a HR of 0.70 for both, and other inputs as per above.

Decision points

Stage-1 (IDEAL IIB trial)

The trial team will evaluate patient CRF and imaging data continuously on a case-by-case basis from each site and provide regular feedback and assessment. Any additional training/guidance is provided as needed. After a site has done an adequate number of cases and has objectively met the primary outcomes and workflow requirements, the completed data set will be revaluated by the trial team including the CI and Lead Investigators. A meeting between the trial team and site is then held to allow feedback from the site and discussion of lessons learned. This meeting is formally documented and if all the criteria are met, the site can then progress to Stage 2 (supplementary files).

Stage-2 (RCT)

Built into the trial is an internal pilot of recruitment to the RCT (Stage-2). There will be a formal stop/go review after 12months of recruitment to the RCT to review the number of randomisations over the pilot period – the stop-go criteria are listed in table 2. If the target of at least 80 randomisations has been met, the trial will continue to recruit for a further 15months. Data from the 80 patients will be included in the final analysis.

	**actual recruitment after 12 months of recruitment**		
**target = 80**	**>80 participants**	**65 - 80 participants**	**<65 participants**
recruitment rate (per centre per month)	0.6	0.45	0.37
stop-go criteria	recruitment feasible<br>proceed with trial	review recruitment strategies report to TSC<br>Continue but modify and monitor closely	recruitment not feasible<br>decision not to proceed

Table 2. Proposed stop-go criteria for the TSC at 12 months.

Data Management

Data will be collected from participants and proxies via questionnaires and case report forms that will be returned to the central trial office in Oxford, via post using a pre-addressed freepost envelope, NHS email as appropriate, or directly into an online secure database (REDCap). In addition, participant images will be stored within the cloud database

Quentry (BrainLab AG). As a third-party processor, BrainLab will not receive any data that could identify participants.

All trial-specific documents, except for the signed consent form and follow-up contact details, will refer to the participant with a unique trial participant number/code and not by name. The data will be stored and used in compliance with the relevant, current data protection laws (Data Protection Act 2018; General Data Protection Regulation (GDPR) 2018). The trial data (including data for SAEs) will be entered onto a validated REDCap trial database developed and maintained by OCTRU and which can only be accessed by authorised users via the application. After closure of the trial and data analyses, the data will be made publicly available at the time of publication. The Trial Master File will be archived for five years from the end of the trial.

Patient and public involvement (PPI)

The trial focuses on keeping good HRQoL for people living with a GB for as long as possible. It has been designed with the help of patient support groups at the Brain Tumour Charity and Brainstrust, the Patient Relative Advisory Group at the Oxford University Hospitals NHS Foundation Trust and the Brain Tumour PPI Group at Imperial College Healthcare NHS Trust. Dr Helen Bulbeck (Brainstrust's Director) has been part of the trial proposal and is one of the trial's investigators.

Trial oversight

The day-to-day management of the trial will be the responsibility of the clinical trial manager, based at Nuffield Department of Surgical Sciences and supported by the Oxford Clinical Trials Research Unit (OCTRU) and the Surgical Intervention Trials Unit (SITU) staff all based at the University of Oxford with the Chief Investigator. This will be overseen by the trial management group, who will meet monthly to assess progress.

A trial steering committee (TSC) and a DSMC will be set up. The DSMC will adopt a DAMOCLES based charter which defines its terms of reference and operation in relation to oversight of the trial. They will not be asked to perform any formal interim analyses of effectiveness. They will, however, see copies of data accrued to date and summaries of that data by treatment group. They will also consider emerging evidence from other related trials or research and review related SAEs that have been reported. They may advise the chair of the TSC at any time if, in their view, the trial should be stopped for ethical reasons, including concerns about participant safety. DSMC meetings will be held at least annually during the recruitment phase of the study.

Quality control

The study may be monitored, or audited in accordance with the current approved protocol, relevant regulations and standard operating procedures by the host organisation or sponsor. A monitoring plan will be developed according to OCTRU standard operating procedures which involves a risk assessment. The monitoring activities are based on the outcome of the risk assessment and may involve central and site monitoring.

Ethics

The trial has Health Research Authority approval (IRAS 264482) and NHS Research and Ethics Committee approval (20/LO/0840).

Author Contributions

PP, SC, DN were responsible for conceptualisation and design of the trial. RM is a trial clinician and drafted manuscript. JAC provided statistical input into the trial design and conduct. CW, MDJ, KA, SJP, VA are trial clinicians and provided input regarding neurosurgical expertise and trial design. MT, LD, MW provided input on neuro-oncology management. MT, LD (Linda Dirven) provided expert opinion on quality-of-life measures. AL, LD (Luke Dixon) developed section on intraoperative ultrasound techniques and analysis. PM was responsible for guidance on the IDEAL framework. NV, MGS developed section on DTI imaging and analysis. HB organised and provided PPI input. VSB provided input into the trial design. All authors provided critical appraisal of the protocol and manuscript.

Acknowledgments
We would like to thank our participants and other research team members (Amy Taylor, Nadjat Medehgri, Jack Morris, Lucy Eldridge, Ariel Wang and Tianshu Liu) involved in the day to day running of the trial. This trial will be conducted as part of the portfolio of trials in the UK Clinical Research Collaboration registered Clinical Trials Unit – the Oxford Clinical Trials Research Unit (OCTRU) and the Surgical Intervention Trial Unit (SITU) at the University of Oxford. It will follow their Standard Operating Procedures ensuring compliance with the principles of Good Clinical Practice and the Declaration of Helsinki and any applicable regulatory requirements.

Funding
This paper presents independent research funded by the National Institute for Health Research (NIHR) under its Efficacy and Mechanism Evaluation Programme (Grant Reference: NIHR 127930; https://fundingawards.nihr.ac.uk/award/NIHR127930). The views expressed are those of the author(s) and not necessarily those of the NIHR or the Department of Health and Social Care. The University of Oxford is the sponsor of the trial. The funders and the sponsor of the trial had no explicit role in the trial design.

Competing interests
The authors do not have any competing interests to disclose.

For peer review only

References

1 Brodbelt A, Greenberg D, Winters T, *et al.* Glioblastoma in England: 2007-2011. *Eur J Cancer* 2015;**51**:533–42. doi:10.1016/j.ejca.2014.12.014

2 Stupp R, Mason WP, van den Bent MJ, *et al.* Radiotherapy plus concomitant and adjuvant temozolomide for glioblastoma. *N Engl J Med* 2005;**352**:987–96. doi:10.1056/nejmoa043330

3 Overview | Brain tumours (primary) and brain metastases in adults | Guidance | NICE. https://www.nice.org.uk/guidance/ng99 (accessed 1 Sep 2021).

4 Creighton H, Beach B, Bamford S-M. Rethinking Cancer. 2015. www.ilcuk.org.uk (accessed 1 Sep 2021).

5 Burnet NG, Jefferies SJ, Benson RJ, *et al.* Years of life lost (YLL) from cancer is an important measure of population burden - And should be considered when allocating research funds. *Br J Cancer* 2005;**92**:241–5. doi:10.1038/sj.bjc.6602321

6 Cihoric N, Tsikkinis A, Minniti G, *et al.* Current status and perspectives of interventional clinical trials for glioblastoma - analysis of ClinicalTrials.gov. Radiat. Oncol. 2017;**12**:1. doi:10.1186/s13014-016-0740-5

7 Li YM, Suki D, Hess K, *et al.* The influence of maximum safe resection of glioblastoma on survival in 1229 patients: Can we do better than gross-total resection? *J Neurosurg* 2015;**124**:1–12. doi:10.3171/2015.5.JNS142087

8 Stummer W, Reulen HJ, Meinel T, *et al.* Extent of resection and survival in glioblastoma multiforme: identification of and adjustment for bias. *Neurosurgery* 2008;**62**:564–76. doi:00006123-200803000-00003 [pii] 10.1227/01.neu.0000317304.31579.17

9 Brown TJ, Brennan MC, Li M, *et al.* Association of the extent of resection with survival in glioblastoma a systematic review and meta-Analysis. *JAMA Oncol* 2016;**2**:1460–9. doi:10.1001/jamaoncol.2016.1373

10 Bal J, Camp SJ, Nandi D. The use of ultrasound in intracranial tumor surgery. Acta Neurochir. (Wien). 2016;**158**:1179–85. doi:10.1007/s00701-016-2803-7

11 Dixon L, Lim A, Grech-Sollars M, *et al.* Intraoperative Ultrasound in Brain Tumor Surgery: A review and implementation guide. *Neurosurg Rev* 2022.

12 Serra C, Stauffer A, Actor B, *et al.* Intraoperative high frequency ultrasound in intracerebral high-grade tumors. *Ultraschall der Medizin* 2012;**33**:E306–12. doi:10.1055/s-0032-1325369

13 Mahboob S, McPhillips R, Qiu Z, *et al.* Intraoperative Ultrasound-Guided Resection of Gliomas: A Meta-Analysis and Review of the Literature. *World Neurosurg* 2016;**92**:255–63. doi:10.1016/j.wneu.2016.05.007

14 Unsgaard G, Rygh OM, Selbekk T, *et al.* Intra-operative 3D ultrasound in neurosurgery. Acta Neurochir. (Wien). 2006;**148**:235–53. doi:10.1007/s00701-005-0688-y

15 Unsgaard G, Ommedal S, Muller T, *et al.* Neuronavigation by intraoperative three-dimensional ultrasound: Initial experience during brain tumor resection. *Neurosurgery* 2002;**50**:804–12. doi:10.1097/00006123-200204000-00022

16 Unsgaard G, Gronningsaeter A, Ommedal S, *et al.* Brain operations guided by real-time two-dimensional ultrasound: New possibilities as a result of improved image quality. *Neurosurgery* 2002;**51**:402–12. doi:10.1097/00006123-200208000-00019

17 Sæther CA, Torsteinsen M, Torp SH, *et al.* Did survival improve after the

For peer review only

implementation of intraoperative neuronavigation and 3d ultrasound in glioblastoma surgery? A retrospective analysis of 192 primary operations. *J Neurol Surgery, Part A Cent Eur Neurosurg* 2012;**73**:73–8. doi:10.1055/s-0031-1297247
18 Camp SJ, Apostolopoulos V, Raptopoulos V, *et al.* Objective image analysis of real-time three-dimensional intraoperative ultrasound for intrinsic brain tumour surgery. *J Ther Ultrasound* 2017;**5**:2. doi:10.1186/s40349-017-0084-0
19 Woydt M, Krone A, Becker G, *et al.* Correlation of Intra-Operative Ultrasound with Histopathologic Findings after Tumour Resection in Supratentorial Gliomas: A Method to Improve Gross Total Tumour Resection. *Acta Neurochir (Wien)* 1996;**138**:1391–8. doi:10.1007/BF01411117
20 Moiyadi A, Shetty P. Navigable Intraoperative Ultrasound and Fluorescence-Guided Resections Are Complementary in Resection Control of Malignant Gliomas: One Size Does Not Fit All. *J Neurol Surgery, Part A Cent Eur Neurosurg* 2014;**75**:434–41. doi:10.1055/s-0034-1372436
21 Voets NL, Bartsch A, Plaha P. Brain white matter fibre tracts: A review of functional neuro-oncological relevance. J. Neurol. Neurosurg. Psychiatry. 2017;**88**:1017–25. doi:10.1136/jnnp-2017-316170
22 Bello L, Riva M, Fava E, *et al.* Tailoring neurophysiological strategies with clinical context enhances resection and safety and expands indications in gliomas involving motor pathways. *Neuro Oncol* 2014;**16**:1110–28. doi:10.1093/neuonc/not327
23 Vassal F, Schneider F, Sontheimer A, *et al.* Intraoperative visualisation of language fascicles by diffusion tensor imaging-based tractography in glioma surgery. *Acta Neurochir (Wien)* 2013;**155**:437–48. doi:10.1007/s00701-012-1580-1
24 Herbet G, Maheu M, Costi E, *et al.* Mapping neuroplastic potential in brain-damaged patients. *Brain* 2016;**139**:829–44. doi:10.1093/brain/awv394
25 Winston GP, Daga P, White MJ, *et al.* Preventing visual field deficits from neurosurgery. *Neurology* 2014;**83**:604–11. doi:10.1212/WNL.0000000000000685
26 Caverzasi E, Hervey-Jumper SL, Jordan KM, *et al.* Identifying preoperative language tracts and predicting postoperative functional recovery using HARDI q-ball fiber tractography in patients with gliomas. *J Neurosurg* 2016;**125**:33–45. doi:10.3171/2015.6.JNS142203
27 Wu JS, Zhou LF, Tang WJ, *et al.* Clinical evaluation and follow-up outcome of diffusion tensor imaging-based functional neuronavigation: A prospective, controlled study in patients with gliomas involving pyramidal tracts. *Neurosurgery* 2007;**61**:935–48. doi:10.1227/01.neu.0000303189.80049.ab
28 Ma R, Livermore LJ, Plaha P. Fast Track Recovery Program After Endoscopic and Awake Intraparenchymal Brain Tumor Surgery. *World Neurosurg* 2016;**93**:246–52. doi:10.1016/j.wneu.2016.06.015
29 Jenkinson MD, Barone DG, Bryant A, *et al.* Intraoperative imaging technology to maximise extent of resection for glioma. Cochrane Database Syst. Rev. 2018;**2018**. doi:10.1002/14651858.CD012788.pub2
30 Hirst A, Philippou Y, Blazeby J, *et al.* No Surgical Innovation Without Evaluation: Evolution and Further Development of the IDEAL Framework and Recommendations. *Ann Surg* 2019;**269**:211–20. doi:10.1097/SLA.0000000000002794
31 McCulloch P, Cook JA, Altman DG, *et al.* IDEAL framework for surgical innovation 1: the idea and development stages. *BMJ* 2013;**346**:f3012. doi:10.1136/bmj.f3012
32 McCulloch P, Altman DG, Campbell WB, *et al.* No surgical innovation without

For peer review only

evaluation: the IDEAL recommendations. Lancet. 2009;**374**:1105–12. doi:10.1016/S0140-6736(09)61116-8

33 Taphoorn MJB, Henriksson R, Bottomley A, *et al.* Health-related quality of life in a randomized phase III study of bevacizumab, temozolomide, and radiotherapy in newly diagnosed glioblastoma. *J Clin Oncol* 2015;**33**:2166–75. doi:10.1200/JCO.2014.60.3217

34 Cook JA, Julious SA, Sones W, *et al.* DELTA 2 guidance on choosing the target difference and undertaking and reporting the sample size calculation for a randomised controlled trial. *BMJ* 2018;**363**. doi:10.1136/bmj.k3750

For peer review only

Figure 1. Trial Flowchart

For peer review only

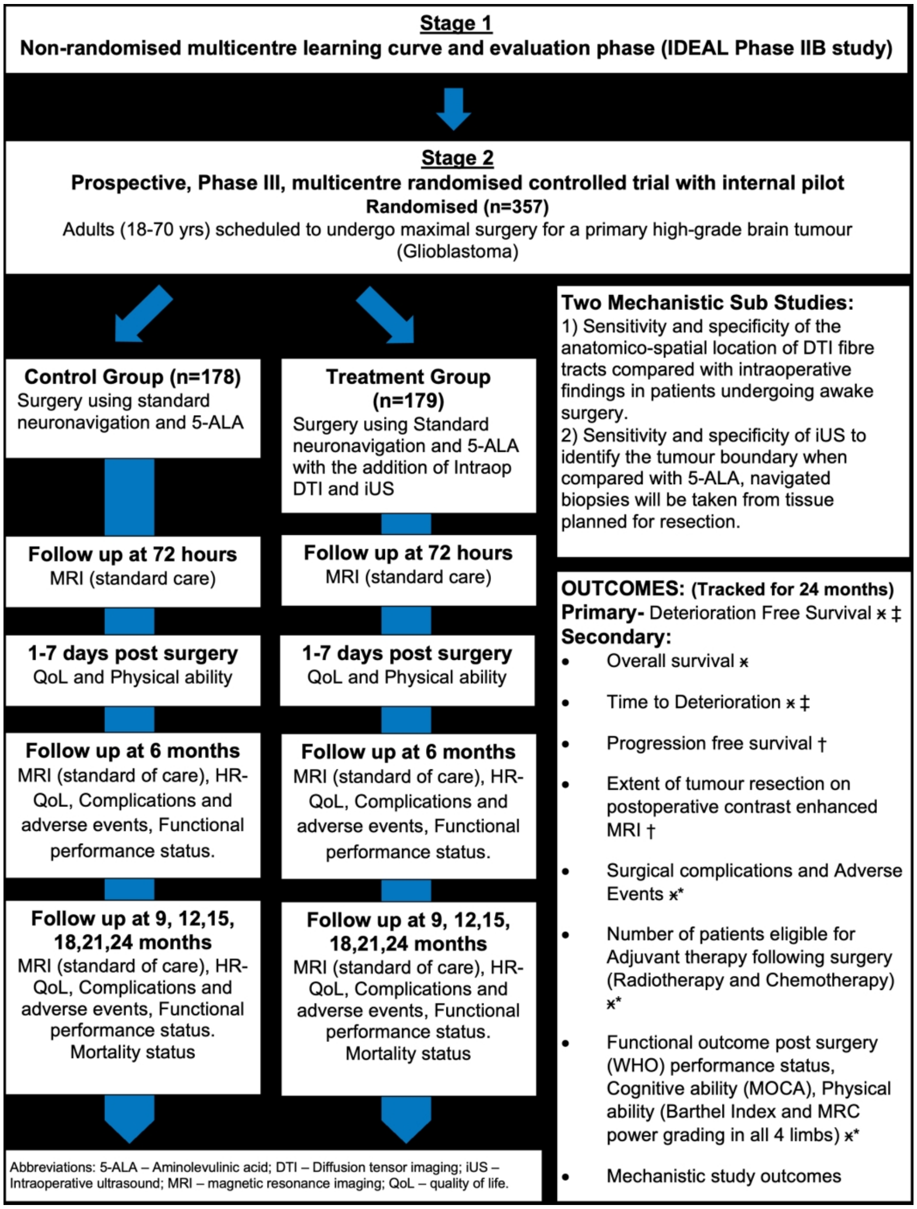

Trial flowchart

188x248mm (300 x 300 DPI)

FUTURE-GB STAGE 1 SITE DATA COMPLETION ASSESSMENT

**Reviewers:**	*Intra operative workflow & DTI:*	Prof Natalie Voets, Puneet Plaha, Miss Joy Roach, Amy Taylor
	Intra operative workflow & US:	Dipankar Nandi, Sophie Camp, Luke Dixon, Amy Taylor
	REDCap data entry & workflow:	Amy Taylor, Jack Morris, Puneet Plaha

**Site name:**		**Total Patients recruited:**		**Total Patients screened:**	

**Study ID**					
**Date of review**					
**Date of surgery**					
**Awake or GA surgery**					
**Pre -op tumour planning on MRI scans**					
**Pre-op tumour volume (cm^3)**					
**Post-op tumour volume (cm^3)**					
**Comments:**					
**MRI DTI and USG Acquisition**					
DTI – Site engaged with trials unit regarding DTI acquisition protocols ?	Yes/No	Yes/No	Yes/No	Yes/No	Yes/No
DTI scan acquired for surgery?	Yes/No	Yes/No	Yes/No	Yes/No	Yes/No
DTI Tracts reconstructed ?	Yes/No	Yes/No	Yes/No	Yes/No	Yes/No
USG used during surgery?	Yes/No	Yes/No	Yes/No	Yes/No	Yes/No
**Redcap data entry complete?**	Yes/No	Yes/No	Yes/No	Yes/No	Yes/No
Any difficulty entering data on REDCap ?	Yes/No	Yes/No	Yes/No	Yes/No	Yes/No
**Intraoperative workflow & Quentry imaging data transfer complete**	Yes/No	Yes/No	Yes/No	Yes/No	Yes/No
Data anonymised	Yes/No	Yes/No	Yes/No	Yes/No	Yes/No
Pre-op MRI scan performed	Yes/No	Yes/No	Yes/No	Yes/No	Yes/No
Pre -op MRI scan transferred					
Any Intra-op Screenshots acquired for awake surgery	Yes/No/ N/A	Yes/No/ N/A	Yes/No/ N/A	Yes/No/ N/A	Yes/No/ N/A
Intra-op Screenshots acquired for GA neurophysiology	Yes/No/ N/A	Yes/No/ N/A	Yes/No/ N/A	Yes/No/ N/A	Yes/No/ N/A
USG pre-resection – pictures/videos	Yes/No	Yes/No	Yes/No	Yes/No	Yes/No
USG post-resection- pictures/videos	Yes/No	Yes/No	Yes/No	Yes/No	Yes/No
Post-op MRI scan performed	Yes/No	Yes/No	Yes/No	Yes/No	Yes/No

FUTURE-GB_S1_Template_SiteDataCompletion_V3.0_18Oct2021.docx

FUTURE-GB STAGE 1 SITE DATA COMPLETION ASSESSMENT

**Post op MRI scan transferred**	Yes/No	Yes/No	Yes/No	Yes/No	Yes/No
**Difficulties in transferring Data to Quentry**	Yes/No	Yes/No	Yes/No	Yes/No	Yes/No
**Site PI - DTI comments**					
**Reviewer - DTI comments**					
**Reviewer - US comments**					
**Site PI - US comments**					
**Primary outcomes for Stage 1 complete**	Yes/No	Yes/No	Yes/No	Yes/No	Yes/No
Operation length					
Successful use of DTI neuronavigation and iUS to achieve complete tumour resection without major neurological deficit *(success defined here as appropriate and competent use of the imaging technologies to achieve the projected surgical outcome for each patient)*					
Extent of tumour resection assessed on postoperative MRI scan					
Surgical Complication and Serious Adverse Events (if applicable)	Yes/No	Yes/No	Yes/No	Yes/No	Yes/No
**Reviewer Comments:**					
**Analysis of imaging data to answer tertiary end point of the RCT study**					
Utility of imaging data to answer the DTI tertiary endpoint					
Utility of imaging data to answer the US tertiary endpoint					
**Reviewer Comments**					

**Overall reviewer comments**	

SITE FEEDBACK LOG

Date of meeting/discussion with site during Stage 1	Type of meeting	Feedback comments

FUTURE-GB_S1_Template_SiteDataCompletion_V3.0_18Oct2021.docx

FUTURE-GB STAGE 2 PROGRESSION AGREEMENT

The Co-Chief investigators of the FUTURE-GB trial agree that the trial site:

<site name and NHS Trust>

has met the following provisions in Stage 1 of the FUTURE-GB trial and recruited <number> participants

Trial team review was conducted on DDMonYYYY and it was agreed on this date (DDMonYYYY) that this site can proceed to Stage 2.

Co-Chief investigators report:

This should include:

1. *Objective endpoints of Stage 1*
2. *Quality of DT and US imaging data*
3. *Any difficulties regarding data-workflow from site*
4. *Any suggestions for improvement*

Note: Completion of this agreement by all signatories will result in the Stage 1 Registration System and screening system being closed by the Trial Manager on or after this date, and requesting that the site is opened to recruitment in the Stage 2 Screening, Randomisation and Database System. The Stage 1 Database system will not be closed to the site until all outstanding data has been entered and cleaned/queried as required by the Trial Statistician.

FUTURE-GB STAGE 2 PROGRESSION AGREEMENT

Name	Signature	Date
Professor Puneet Plaha		
Professor Dipankar Nandi		
Miss Sophie Camp		

PARTICIPANT ID: FG-XX-XXXX

PARTICIPANT QUESTIONNAIRE

Thank you for agreeing to participate in the FUTURE-GB study.

This is the baseline questionnaire.

We would be grateful if you could complete this questionnaire on how you are feeling and if you are having any issues due to your glioblastoma.

We will ask you to complete this same questionnaire again before you leave hospital, then at 6 weeks after your entered the study, 3 months after you entered the study and every 3 months thereafter up until 24 months.

If you have any questions about the study or this questionnaire, please do not hesitate to contact a member of the study team on future-gb@nds.ox.ac.uk or call 01865 xxxxxx (Monday to Friday, 9-4pm), there is an answering machine for messages outside of these times, or contact your local clinical team)

For peer review only

For peer review only - http://bmjopen.bmj.com/site/about/guidelines.xhtml

PARTICIPANT ID: FG-XX-XXXX

We are interested in some things about you and your health.

Please answer all of the questions yourself by circling the number that best applies to you. There are no “right” or “wrong” answers. The information that you provide will remain strictly confidential.

We appreciated that you may be very busy and this may be a distressing time, therefore if you are unable to complete this questionnaire either due to time or other reasons – if at all possible however we would be very grateful if could complete the date below and questions 29 and 30 on page 4.

What is today’s date: DD/MM/YYYY

For peer review only - http://bmjopen.bmj.com/site/about/guidelines.xhtml

PARTICIPANT ID: FG-XX-XXXX

		Not at All	A Little	Quite a Bit	Very Much
1.	Does you have any trouble doing strenuous activities, like carrying a heavy shopping bag or a suitcase?	1	2	3	4
2.	Do you have any trouble taking a long walk?	1	2	3	4
3.	Do you have any trouble taking a short walk outside of the house?	1	2	3	4
4.	Do you need to stay in bed or a chair during the day?	1	2	3	4
5.	Do you need help with eating, dressing, washing yourself or using the toilet?	1	2	3	4

During the past week:

		Not at All	A Little	Quite a Bit	Very Much
6.	Were you limited in doing either your work or other daily activities?	1	2	3	4
7.	Were you limited in pursuing their hobbies or other leisure time activities?	1	2	3	4
8.	Were you short of breath?	1	2	3	4
9.	Have you had pain?	1	2	3	4
10.	Did you need to rest?	1	2	3	4
11.	Have you had trouble sleeping?	1	2	3	4
12.	Have you felt weak?	1	2	3	4
13.	Have you lacked their appetite?	1	2	3	4
14.	Have you felt nauseated?	1	2	3	4
15.	Have you vomited?	1	2	3	4
16.	Have you been constipated?	1	2	3	4
17.	Have you had diarrhoea?	1	2	3	4
18.	Were you tired?	1	2	3	4
19.	Did pain interfere with your daily activities?	1	2	3	4
20.	Have you had difficulty concentrating on things, like reading a newspaper or watching television?	1	2	3	4
21.	Did you feel tense?	1	2	3	4
22.	Did you worry?	1	2	3	4
23.	Did you feel irritable?	1	2	3	4
24.	Did you feel depressed?	1	2	3	4
25.	Have you had difficulty remembering things?	1	2	3	4
26.	Has your physical condition or medical treatment interfered with your family life?	1	2	3	4
27.	Has your physical condition or medical treatment interfered with your social activities?	1	2	3	4

For peer review only

For peer review only - http://bmjopen.bmj.com/site/about/guidelines.xhtml

PARTICIPANT ID: FG-XX-XXXX

		Not at All	A Little	Quite a Bit	Very Much
28.	Has your physical condition or medical treatment caused you financial difficulties?	1	2	3	4

For the following questions please circle the number between 1 and 7 that best applies to you

29. How would you rate your overall health during the past week?

1 2 3 4 5 6 7

Very poor Excellent

30. How would you rate your overall quality of life during the past week?

1 2 3 4 5 6 7

Very poor Excellent

For peer review only

Patients sometimes report that they have the following symptoms. Please indicate the extent to which you have experienced the below symptoms or problems during the past week.

During the past week:

		**Not at All**	**A Little**	**Quite a Bit**	**Very Much**
31.	Did you feel uncertain about the future?	1	2	3	4
32.	Did you feel you had setbacks in your condition?	1	2	3	4
33.	Were you concerned about disruption of family life?	1	2	3	4
34.	Did you have headaches?	1	2	3	4
35.	Did your outlook on the future worsen?	1	2	3	4
36.	Did you have double vision?	1	2	3	4

For peer review only - http://bmjopen.bmj.com/site/about/guidelines.xhtml

PARTICIPANT ID: FG-XX-XXXX

		Not at All	A Little	Quite a Bit	Very Much
37.	Was your vision blurred?	1	2	3	4
38.	Did you have difficulty reading because of your vision?	1	2	3	4
39.	Did you have seizures?	1	2	3	4
40.	Did you have weakness on one side of your body?	1	2	3	4
41.	Did you have trouble finding the right words to express yourself?	1	2	3	4
42.	Did you have difficulty speaking?	1	2	3	4
43.	Did you trouble communicating your thoughts?	1	2	3	4
44.	Did you feel drowsy during the daytime?	1	2	3	4
45.	Did you have trouble with your coordination?	1	2	3	4
46.	Did hair loss bother you?				
47.	Did itching of their skin bother you?	1	2	3	4
48.	Did you have weakness of both legs?	1	2	3	4
49.	Did you feel unsteady on your feet?	1	2	3	4
50.	Did you have trouble controlling your bladder?	1	2	3	4

For peer review only

For peer review only - http://bmjopen.bmj.com/site/about/guidelines.xhtml

PARTICIPANT ID: FG-XX-XXXX

SITE ADMINISTERED QUESTIONNAIRE

Please tick which time point this questionnaire relates to:

Baseline	☐	12 months	☐
Hospital Discharge	☐	15 months	☐
6 weeks	☐	18 months	☐
3 months	☐	21 months	☐
6 months	☐	24 months	☐
9 months	☐		

Date of completion: DD/MM/YYYY

Completed by: (PRINT NAME)…………………………………………….

This document contains the following validated questionnaires:

- Barthel Index
- MOCA Assessment
- MRC Muscle Power Scale
- WHO Performance Status

For peer review only - http://bmjopen.bmj.com/site/about/guidelines.xhtml

PARTICIPANT ID: FG-XX-XXXX

BARTHEL INDEX

Activity	Score
**FEEDING**<br>0 = unable<br>5 = needs help cutting, spreading butter, etc., or requires modified diet<br>10 = independent	______
**BATHING**<br>0 = dependent<br>5 = independent (or in shower)	______
**GROOMING**<br>0 = needs to help with personal care<br>5 = independent face/hair/teeth/shaving (implements provided)	______
**DRESSING**<br>0 = dependent<br>5 = needs help but can do about half unaided<br>10 = independent (including buttons, zips, laces, etc.)	______
**BOWELS**<br>0 = incontinent (or needs to be given enemas)<br>5 = occasional accident<br>10 = continent	______
**BLADDER**<br>0 = incontinent, or catheterized and unable to manage alone<br>5 = occasional accident<br>10 = continent	______
**TOILET USE**<br>0 = dependent<br>5 = needs some help, but can do something alone<br>10 = independent (on and off, dressing, wiping)	______
**TRANSFERS (BED TO CHAIR AND BACK)**<br>0 = unable, no sitting balance<br>5 = major help (one or two people, physical), can sit<br>10 = minor help (verbal or physical)<br>15 = independent	______
**MOBILITY (ON LEVEL SURFACES)**<br>0 = immobile or < 50 yards<br>5 = wheelchair independent, including corners, > 50 yards<br>10 = walks with help of one person (verbal or physical) > 50 yards<br>15 = independent (but may use any aid; for example, stick) > 50 yards	______
**STAIRS**<br>0 = unable<br>5 = needs help (verbal, physical, carrying aid)<br>10 = independent	______
**TOTAL (0–100):**	______

For peer review only - http://bmjopen.bmj.com/site/about/guidelines.xhtml

PARTICIPANT ID: FG-XX-XXXX

MOCA ASSESSMENT

VISUOSPATIAL / EXECUTIVE			POINTS
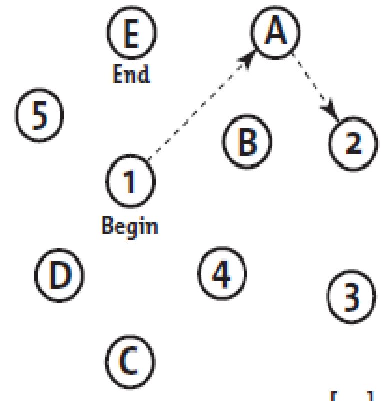 [ ]	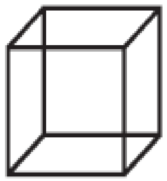 Copy cube [ ]	Draw CLOCK (Ten past eleven) (3 points)<br>[ ] Contour [ ] Numbers [ ] Hands	__/5

NAMING			POINTS
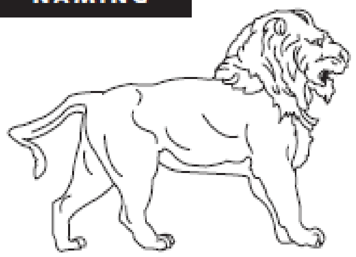 [ ]	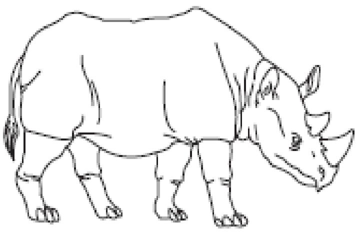 [ ]	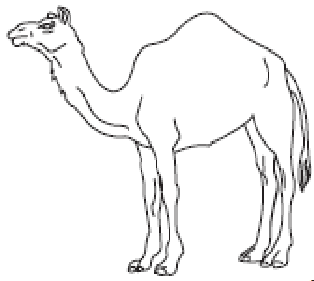 [ ]	__/3

MEMORY	Read list of words, subject must repeat them. Do 2 trials. Do a recall after 5 minutes.		FACE	VELVET	CHURCH	DAISY	RED	No points
		1st trial						
		2nd trial						

ATTENTION

		Points
Read list of digits (1 digit/ sec.).	Subject has to repeat them in the forward order [ ] 2 1 8 5 4<br>Subject has to repeat them in the backward order [ ] 7 4 2	__/2
Read list of letters. The subject must tap with his hand at each letter A. No points if ≥ 2 errors	[ ] F B A C M N A A J K L B A F A K D E A A A J A M O F A A B	__/1
Serial 7 subtraction starting at 100	[ ] 93 [ ] 86 [ ] 79 [ ] 72 [ ] 65<br>4 or 5 correct subtractions: **3 pts**, 2 or 3 correct: **2 pts**, 1 correct: **1 pt**, 0 correct: **0 pt**	__/3

LANGUAGE

	Points
Repeat : I only know that John is the one to help today. [ ]<br>The cat always hid under the couch when dogs were in the room. [ ]	__/2
Fluency / Name maximum number of words in one minute that begin with the letter F [ ] ____ (N ≥ 11 words)	__/1

ABSTRACTION	Similarity between e.g. banana - orange = fruit [ ] train – bicycle [ ] watch - ruler	__/2

DELAYED RECALL	Has to recall words WITH NO CUE	FACE [ ]	VELVET [ ]	CHURCH [ ]	DAISY [ ]	RED [ ]	Points for UNCUED recall only	__/5
Optional	Category cue							
	Multiple choice cue							

ORIENTATION	[ ] Date	[ ] Month	[ ] Year	[ ] Day	[ ] Place	[ ] City	__/6

© Z.Nasreddine MD Version November 7, 2004

www.mocatest.org

Normal ≥ 26 / 30

TOTAL __/30

Add 1 point if ≤ 12 yr edu

For peer review only - http://bmjopen.bmj.com/site/about/guidelines.xhtml

PARTICIPANT ID: FG-XX-XXXX

MRC MUSCLE POWER SCALE

Score	Description
**0**	No contraction
**1**	Flicker or trace of contraction
**2**	Active movement, with gravity eliminated
**3**	Active movement against gravity
**4**	Active movement against gravity and resistance
**5**	Normal power

Please grade the 4 limbs of the patient

Left arm: ☐

Right arm: ☐

Left leg: ☐

Right leg: ☐

For peer review

WHO PERFORMANCE STATUS

The WHO performance status classification categorises patients as:

- 0: able to carry out all normal activity without restriction
- 1: restricted in strenuous activity but ambulatory and able to carry out light work
- 2: ambulatory and capable of all self-care but unable to carry out any work activities; up and about more than 50% of waking hours
- 3: symptomatic and in a chair or in bed for greater than 50% of the day but not bedridden
- 4: completely disabled; cannot carry out any self-care; totally confined to bed or chair.

Please circle the WHO Performance status for the patient as at today:

0 1 2 3 4

For peer review only - http://bmjopen.bmj.com/site/about/guidelines.xhtml

PARTICIPANT ID: FG-XX-XXXX

PARTICIPANT QUESTIONNAIRE

Thank you for agreeing to participate in the FUTURE-GB study.

Please tick which time point this questionnaire relates to:

Hospital Discharge ☐
6 weeks ☐
3 months ☐
6 months ☐
9 months ☐
12 months ☐
15 months ☐
18 months ☐
21 months ☐
24 months ☐

We would be grateful if you could complete this questionnaire on how you are feeling and if you are having any issues due to your glioblastoma.

If you have any questions about the study or this questionnaire, please do not hesitate to contact a member of the study team on future-gb@nds.ox.ac.uk or call 01865 xxxxxx (Monday to Friday, 9-4pm), there is an answering machine for messages outside of these times, or contact your local clinical team)

For peer review only - http://bmjopen.bmj.com/site/about/guidelines.xhtml

PARTICIPANT ID: FG-XX-XXXX

We are interested in some things about you and your health.

Please answer all of the questions yourself by circling the number that best applies to you. There are no "right" or "wrong" answers. The information that you provide will remain strictly confidential.

For peer review only

We appreciated that you may be very busy and this may be a distressing time, therefore if you are unable to complete this questionnaire either due to time or other reasons – if at all possible however we would be very grateful if could complete the date below and questions 29 and 30 on page 4.

What is today's date: DD/MM/YYYY

For peer review only - http://bmjopen.bmj.com/site/about/guidelines.xhtml

PARTICIPANT ID: FG-XX-XXXX

		Not at All	A Little	Quite a Bit	Very Much
1.	Does you have any trouble doing strenuous activities, like carrying a heavy shopping bag or a suitcase?	1	2	3	4
2.	Do you have any trouble taking a <u>long</u> walk?	1	2	3	4
3.	Do you have any trouble taking a <u>short</u> walk outside of the house?	1	2	3	4
4.	Do you need to stay in bed or a chair during the day?	1	2	3	4
5.	Do you need help with eating, dressing, washing yourself or using the toilet?	1	2	3	4

During the past week:

		Not at All	A Little	Quite a Bit	Very Much
6.	Were you limited in doing either your work or other daily activities?	1	2	3	4
7.	Were you limited in pursuing their hobbies or other leisure time activities?	1	2	3	4
8.	Were you short of breath?	1	2	3	4
9.	Have you had pain?	1	2	3	4
10.	Did you need to rest?	1	2	3	4
11.	Have you had trouble sleeping?	1	2	3	4
12.	Have you felt weak?	1	2	3	4
13.	Have you lacked their appetite?	1	2	3	4
14.	Have you felt nauseated?	1	2	3	4
15.	Have you vomited?	1	2	3	4
16.	Have you been constipated?	1	2	3	4
17.	Have you had diarrhoea?	1	2	3	4
18.	Were you tired?	1	2	3	4
19.	Did pain interfere with your daily activities?	1	2	3	4
20.	Have you had difficulty concentrating on things, like reading a newspaper or watching television?	1	2	3	4
21.	Did you feel tense?	1	2	3	4
22.	Did you worry?	1	2	3	4
23.	Did you feel irritable?	1	2	3	4
24.	Did you feel depressed?	1	2	3	4
25.	Have you had difficulty remembering things?	1	2	3	4
26.	Has your physical condition or medical treatment interfered with your <u>family</u> life?	1	2	3	4
27.	Has your physical condition or medical treatment interfered with your <u>social</u> activities?	1	2	3	4

For peer review only - http://bmjopen.bmj.com/site/about/guidelines.xhtml
For peer review only

PARTICIPANT ID: FG-XX-XXXX

		Not at All	A Little	Quite a Bit	Very Much
28.	Has your physical condition or medical treatment caused you financial difficulties?	1	2	3	4

For the following questions please circle the number between 1 and 7 that best applies to you

29. How would you rate your overall <u>health</u> during the past week?

1 2 3 4 5 6 7

Very poor Excellent

30. How would you rate your overall <u>quality of life</u> during the past week?

1 2 3 4 5 6 7

Very poor Excellent

Patients sometimes report that they have the following symptoms. Please indicate the extent to which you have experienced the below symptoms or problems during the past week.

During the past week:

		**Not at All**	**A Little**	**Quite a Bit**	**Very Much**
31.	Did you feel uncertain about the future?	1	2	3	4
32.	Did you feel you had setbacks in your condition?	1	2	3	4
33.	Were you concerned about disruption of family life?	1	2	3	4
34.	Did you have headaches?	1	2	3	4
35.	Did your outlook on the future worsen?	1	2	3	4
36.	Did you have double vision?	1	2	3	4

For peer review only - http://bmjopen.bmj.com/site/about/guidelines.xhtml

For peer review only

PARTICIPANT ID: FG-XX-XXXX

		Not at All	A Little	Quite a Bit	Very Much
37.	Was your vision blurred?	1	2	3	4
38.	Did you have difficulty reading because of your vision?	1	2	3	4
39.	Did you have seizures?	1	2	3	4
40.	Did you have weakness on one side of your body?	1	2	3	4
41.	Did you have trouble finding the right words to express yourself?	1	2	3	4
42.	Did you have difficulty speaking?	1	2	3	4
43.	Did you trouble communicating your thoughts?	1	2	3	4
44.	Did you feel drowsy during the daytime?	1	2	3	4
45.	Did you have trouble with your coordination?	1	2	3	4
46.	Did hair loss bother you?				
47.	Did itching of their skin bother you?	1	2	3	4
48.	Did you have weakness of both legs?	1	2	3	4
49.	Did you feel unsteady on your feet?	1	2	3	4
50.	Did you have trouble controlling your bladder?	1	2	3	4

For peer review only

For peer review only - http://bmjopen.bmj.com/site/about/guidelines.xhtml

PARTICIPANT ID: FG-XX-XXXX

PARTICIPANT QUESTIONNAIRE

Thank you for agreeing to participate in the FUTURE-GB study.

This is the hospital discharge questionnaire.

We would be grateful if you could complete this questionnaire on how you are feeling and if you are having any issues due to your glioblastoma.

This is the same questionnaire as the one you have completed previously.

If you have any questions about the study or this questionnaire, please do not hesitate to contact a member of the study team on future-gb@nds.ox.ac.uk or call 01865 xxxxxx (Monday to Friday, 9-4pm), there is an answering machine for messages outside of these times, or contact your local clinical team)

For peer review only

For peer review only - http://bmjopen.bmj.com/site/about/guidelines.xhtml

PARTICIPANT ID: FG-XX-XXXX

We are interested in some things about you and your health.

Please answer all of the questions yourself by circling the number that best applies to you. There are no “right” or “wrong” answers. The information that you provide will remain strictly confidential.

For peer review only

We appreciated that you may be very busy and this may be a distressing time, therefore if you are unable to complete this questionnaire either due to time or other reasons – if at all possible however we would be very grateful if could complete the date below and questions 29 and 30 on page 4.

What is today’s date: DD/MM/YYYY

For peer review only - http://bmjopen.bmj.com/site/about/guidelines.xhtml

PARTICIPANT ID: FG-XX-XXXX

		Not at All	A Little	Quite a Bit	Very Much
1.	Does you have any trouble doing strenuous activities, like carrying a heavy shopping bag or a suitcase?	1	2	3	4
2.	Do you have any trouble taking a long walk?	1	2	3	4
3.	Do you have any trouble taking a short walk outside of the house?	1	2	3	4
4.	Do you need to stay in bed or a chair during the day?	1	2	3	4
5.	Do you need help with eating, dressing, washing yourself or using the toilet?	1	2	3	4

During the past week:

		Not at All	A Little	Quite a Bit	Very Much
6.	Were you limited in doing either your work or other daily activities?	1	2	3	4
7.	Were you limited in pursuing their hobbies or other leisure time activities?	1	2	3	4
8.	Were you short of breath?	1	2	3	4
9.	Have you had pain?	1	2	3	4
10.	Did you need to rest?	1	2	3	4
11.	Have you had trouble sleeping?	1	2	3	4
12.	Have you felt weak?	1	2	3	4
13.	Have you lacked their appetite?	1	2	3	4
14.	Have you felt nauseated?	1	2	3	4
15.	Have you vomited?	1	2	3	4
16.	Have you been constipated?	1	2	3	4
17.	Have you had diarrhoea?	1	2	3	4
18.	Were you tired?	1	2	3	4
19.	Did pain interfere with your daily activities?	1	2	3	4
20.	Have you had difficulty concentrating on things, like reading a newspaper or watching television?	1	2	3	4
21.	Did you feel tense?	1	2	3	4
22.	Did you worry?	1	2	3	4
23.	Did you feel irritable?	1	2	3	4
24.	Did you feel depressed?	1	2	3	4
25.	Have you had difficulty remembering things?	1	2	3	4
26.	Has your physical condition or medical treatment interfered with your family life?	1	2	3	4
27.	Has your physical condition or medical treatment interfered with your social activities?	1	2	3	4

For peer review only - http://bmjopen.bmj.com/site/about/guidelines.xhtml

For peer review only

PARTICIPANT ID: FG-XX-XXXX

		Not at All	A Little	Quite a Bit	Very Much
28.	Has your physical condition or medical treatment caused you financial difficulties?	1	2	3	4

For the following questions please circle the number between 1 and 7 that best applies to you

29. How would you rate your overall health during the past week?

1 2 3 4 5 6 7

Very poor Excellent

30. How would you rate your overall quality of life during the past week?

1 2 3 4 5 6 7

Very poor Excellent

Patients sometimes report that they have the following symptoms. Please indicate the extent to which you have experienced the below symptoms or problems during the past week.

During the past week:

		**Not at All**	**A Little**	**Quite a Bit**	**Very Much**
31.	Did you feel uncertain about the future?	1	2	3	4
32.	Did you feel you had setbacks in your condition?	1	2	3	4
33.	Were you concerned about disruption of family life?	1	2	3	4
34.	Did you have headaches?	1	2	3	4
35.	Did your outlook on the future worsen?	1	2	3	4
36.	Did you have double vision?	1	2	3	4

For peer review only - http://bmjopen.bmj.com/site/about/guidelines.xhtml

PARTICIPANT ID: FG-XX-XXXX

		Not at All	A Little	Quite a Bit	Very Much
37.	Was your vision blurred?	1	2	3	4
38.	Did you have difficulty reading because of your vision?	1	2	3	4
39.	Did you have seizures?	1	2	3	4
40.	Did you have weakness on one side of your body?	1	2	3	4
41.	Did you have trouble finding the right words to express yourself?	1	2	3	4
42.	Did you have difficulty speaking?	1	2	3	4
43.	Did you trouble communicating your thoughts?	1	2	3	4
44.	Did you feel drowsy during the daytime?	1	2	3	4
45.	Did you have trouble with your coordination?	1	2	3	4
46.	Did hair loss bother you?				
47.	Did itching of their skin bother you?	1	2	3	4
48.	Did you have weakness of both legs?	1	2	3	4
49.	Did you feel unsteady on your feet?	1	2	3	4
50.	Did you have trouble controlling your bladder?	1	2	3	4

For peer review only

For peer review only - http://bmjopen.bmj.com/site/about/guidelines.xhtml

PROXY ID: FG-XX-XXXX

PROXY QUESTIONNAIRE

Thank you for agreeing to participate in the FUTURE-GB study as a proxy.

This is the 3 months post operation questionnaire.

We would be grateful if you could complete the questionnaire on your opinion of how you friend/spouse/relative is coping in their glioblastoma journey. This is the same questionnaire as all the others you have completed to date.

If you have any questions about the study or this questionnaire, please do not hesitate to contact a member of the study team on future-gb@nds.ox.ac.uk or call 01865 xxxxxx (Monday to Friday, 9-4pm, there is an answering machine for messages outside of these times)

For peer review only

PROXY ID: FG-XX-XXXX

We are interested in some things about the person and their health of the person you have agreed to act as a proxy for. The below questionnaire will use the term friend – we appreciate though that you may be answering about a relative/friend or spouse. We hope you will allow the use of this term for the purposes of this questionnaire.

Please answer all of the questions yourself by circling the number that best applies to them. There are no "right" or "wrong" answers. The information that you provide will remain strictly confidential.

We appreciated that you may be very busy and this may be a distressing time, therefore if you are unable to complete this questionnaire either due to time or other reasons – if at all possible however we would be very grateful if could complete the date below and questions 29 and 30 on page 4.

What is today's date: DD/MM/YYYY

This document contains the following validated questionnaires:

- EORTC QLQ-C30
- EORTC QLQ - BN20

For peer review only - http://bmjopen.bmj.com/site/about/guidelines.xhtml

PROXY ID: FG-XX-XXXX

		Not at All	A Little	Quite a Bit	Very Much
1.	Does your friend have any trouble doing strenuous activities, like carrying a heavy shopping bag or a suitcase?	1	2	3	4
2.	Does your friend have any trouble taking a long walk?	1	2	3	4
3.	Does your friend have any trouble taking a short walk outside of the house?	1	2	3	4
4.	Does your friend need to stay in bed or a chair during the day?	1	2	3	4
5.	Does your friend need help with eating, dressing, washing themselves or using the toilet?	1	2	3	4

During the past week:

		Not at All	A Little	Quite a Bit	Very Much
6.	Was your friend limited in doing either work or other daily activities?	1	2	3	4
7.	Was your friend limited in pursuing their hobbies or other leisure time activities?	1	2	3	4
8.	Were they short of breath?	1	2	3	4
9.	Have they had pain?	1	2	3	4
10.	Have they needed to rest?	1	2	3	4
11.	Have they had trouble sleeping?	1	2	3	4
12.	Have they felt weak?	1	2	3	4
13.	Have they lacked their appetite?	1	2	3	4
14.	Have they felt nauseated?	1	2	3	4
15.	Have they vomited?	1	2	3	4
16.	Have they been constipated?	1	2	3	4
17.	Have they had diarrhoea?	1	2	3	4
18.	Have they been tired?	1	2	3	4
19.	Has pain interfered with their daily activities?	1	2	3	4
20.	Have they have difficulty concentrating on things, like reading a newspaper or watching television?	1	2	3	4
21.	Have they felt tense?	1	2	3	4
22.	Have they worried?	1	2	3	4
23.	Have they felt irritable?	1	2	3	4
24.	Have they felt depressed?	1	2	3	4
25.	Have they had difficulty remembering things?	1	2	3	4
26.	Has their physical condition or medical treatment interfered with their family life?	1	2	3	4

For peer review only - http://bmjopen.bmj.com/site/about/guidelines.xhtml

PROXY ID: FG-XX-XXXX

		Not at All	A Little	Quite a Bit	Very Much
27.	Has their physical condition or medical treatment interfered with their social activities?	1	2	3	4
28.	Has their physical condition or medical treatment caused them financial difficulties?	1	2	3	4

For the following questions please circle the number between 1 and 7 that best applies to the person you are a proxy for in your opinion

29. How would you rate their overall health during the past week?

1 2 3 4 5 6 7

Very poor Excellent

30. How would you rate their overall quality of life during the past week?

1 2 3 4 5 6 7

Very poor Excellent

Patients sometimes report that they have the following symptoms. Please indicate the extent to which your relative/proxy/friend have experienced the below symptoms or problems during the past week.

During the past week:

		**Not at All**	**A Little**	**Quite a Bit**	**Very Much**
31.	Did they feel uncertain about the future?	1	2	3	4
32.	Did they feel that have had setbacks in their condition?	1	2	3	4
33.	Were they concerned about disruption of family life?	1	2	3	4
34.	Did they have headaches?	1	2	3	4

For peer review only - http://bmjopen.bmj.com/site/about/guidelines.xhtml

PROXY ID: FG-XX-XXXX

		Not at All	A Little	Quite a Bit	Very Much
35.	Did their outlook on the future worsen?	1	2	3	4
36.	Did they have double vision?	1	2	3	4
37.	Has their vision blurred?	1	2	3	4
38.	Did they have difficulty reading because of their vision?	1	2	3	4
39.	Did they have seizures?	1	2	3	4
40.	Did they have weakness on one side of their body?	1	2	3	4
41.	Did they have trouble finding the right words to express themselves?	1	2	3	4
42.	Did they have difficulty speaking?	1	2	3	4
43.	Did they have trouble communicating their thoughts?	1	2	3	4
44.	Did they feel drowsy during the daytime?	1	2	3	4
45.	Did they trouble with their coordination?	1	2	3	4
46.	Did hair loss bother them?				
47.	Did itching of their skin bother them?	1	2	3	4
48.	Did they have weakness of both of their legs?	1	2	3	4
49.	Did they feel unsteady on their feet?	1	2	3	4
50.	Did they have trouble controlling their bladder?	1	2	3	4

For peer review only

For peer review only - http://bmjopen.bmj.com/site/about/guidelines.xhtml

Oxford University Hospitals
NHS Foundation Trust

Functional and Ultrasound guided Resection of Glioblastoma – the FUTURE-GB study –

Stage 1- IDEAL 2b Phase

Patient Information Leaflet

For peer review only

Invitation to join the FUTURE-GB study

We would like to invite you to take part in a research study (also called a clinical trial).

Before you decide whether to take part or not, it is important that you understand why we are doing this study and what it will involve.

Please take time to read the following information and talk to others about the study. If anything is unclear, or if you would like more information, please ask a member of the study team who will be happy to answer any questions.

What is the purpose of this study?

There are many different types of brain tumours. These can vary in how quickly they grow and what symptoms they cause. For a brain tumour that grows quickly it is important to remove as much tumour tissue as possible. To do this without causing damage to important functional parts of the brain involved in speaking, moving etc., we need accurate imaging during surgery. Several different types of imaging are used during operations, but at present we don't know how effective they are and whether they are actually better than standard care.

We have been funded by the National Institute of Health Research (NIHR) which receives its funding from the UK Government, to find out whether some of these available newer technologies improve quality of life, and life expectancy for people with brain tumours who undergo surgery. We also want to see if using these techniques during an operation means it takes more time for a tumour to come back, and if people have fewer complications from the surgery.

Stage 1

In the first part of the study (Stage 1) we are studying how to use these technologies in combination to achieve the best effect. This means that surgeons will be mentored in the best use of the imaging methods. The surgical teams too will be familiarised with a standard way of using them together. We will carefully monitor how the new methods are used during this phase and discuss them with the surgeons. The aim of this stage of the study is to standardise their use, as we want to have them used in the same way in all participating hospitals by the start of Stage 2. Everyone who agrees to take part in this stage may be helping the doctors treating you familiarise themselves with the new technologies. It is highly likely that your surgeon is already familiar with the use of these technologies.

Note: <u>You are only being asked to take part in Stage 1 and if you agree to take part you will only be part of Stage 1 of this study</u>

FUNDED BY NIHR | National Institute for Health Research · OCTRU · SITU · UNIVERSITY OF OXFORD · Imperial College London

For peer review only - http://bmjopen.bmj.com/site/about/guidelines.xhtml

Oxford University Hospitals
NHS Foundation Trust

For information – once approximately 5 people have agreed to take part in Stage 1 and their operations have taken place at this hospital, Stage 2 of the study will start at this hospital– the information below tells you more about Stage 2.

Stage 2

Stage 2 of the study will compare the operation using the new imaging techniques with the standard operation - so half of those that agree will have the additional, newer, technologies and half will have the standard operation. We hope this will allow us to find a definite answer about which technologies should be used during an operation. The aim of the surgery is to remove safely as much tumour as possible, whilst minimising the risks of damaging brain function and hence affecting quality of life.

The technologies that will be used in this study are all available and in use across NHS practice, and have been shown to be safe. No one knows whether using all of them together will have a definite positive effect on outcome, but it is logical to expect that it should.

The design of this study (FUTURE-GB) has involved patients, their families and healthcare professionals, including brain surgeons, using their knowledge and experience at every stage of project development.

Who is taking part and why have I been invited to take part?

We are hoping to enrol 75 people aged 18 or over, from approximately15 neurosurgical centres in the UK. You will have surgery in your local hospital, which is participating in this trial.

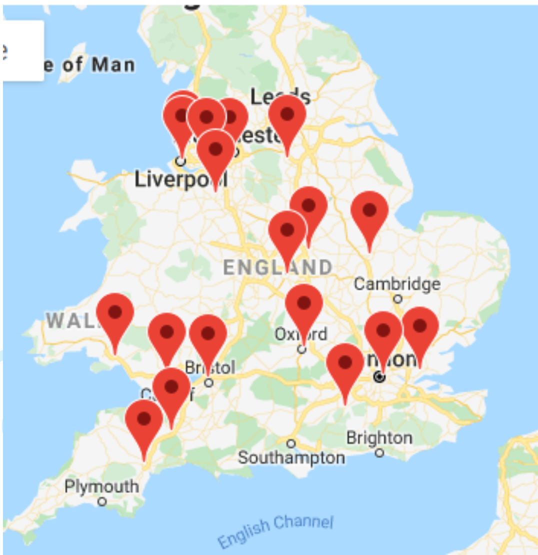

You have been invited to take part because your brain scan suggests you have a brain tumour which comes from the brain itself, rather than from a cancer elsewhere in the body which has spread to the brain. Your scan also suggests the tumour is likely to be aggressive, called a glioblastoma or high-grade tumour.

Do I have to take part in this study?

No, you are under no obligation to take part in the study. Deciding not to will not affect the treatment/care you receive from your team. It is up to you to decide whether to take part or not.

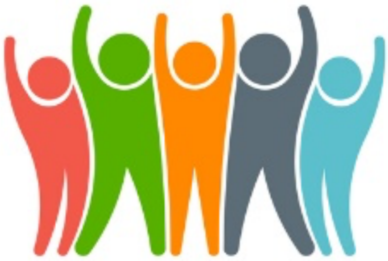

Please keep this leaflet and use it as it may to help you make your decision. If you decide to take part, you will be asked to sign another consent form, as well as that used for your NHS operation.

If you choose not to join the study, you will receive the routine NHS treatment, as agreed by your local treating team of healthcare professionals, in accordance with standard NHS practice as deemed appropriate by your treating team. A note will be made of your age and gender, so that we can find out who decides not to take part. You cannot be identified from this data. A researcher may ask you if you would be happy to give a reason for not wanting to take part in the study. Giving this information is entirely voluntary.

Should you decide to take part, you are still free to withdraw at any time and without giving a reason. This will not affect the standard of care you receive as either an inpatient or an outpatient.

FUNDED BY NIHR | National Institute for Health Research
OCTRU
SITU
UNIVERSITY OF OXFORD
Imperial College London

For peer review only - http://bmjopen.bmj.com/site/about/guidelines.xhtml

Oxford University Hospitals
NHS Foundation Trust

What will happen if I take part?

If you are happy to take part in this study, a researcher will ask you some simple questions and check your medical history to confirm that you are eligible.

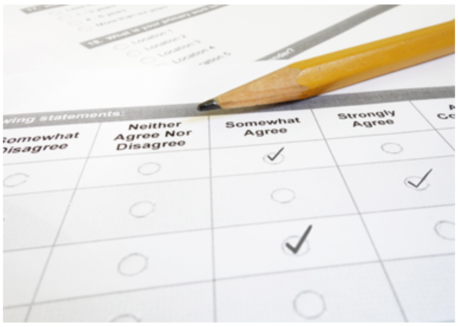

Initial assessment: If you are eligible and wish to participate, you will be asked to sign and date a consent form for the study. Researchers will then take from your medical notes, information about your brain tumour history including the symptoms you have had and where your tumour is.

The researchers would then record details about your operation and details of when you leave hospital after your operation. No questions will need to be asked of you as this will either be standard things already being recorded in your notes or when the technologies are being used, the settings being used in your operation.

Most significantly for those that agree to take part in FUTURE-GB the time taken for your preoperative scans (which you will have by being in the study or not) will perhaps take another 5 minutes. Your operation may also be slightly longer due to the technologies being used – this might perhaps extend it by 15 minutes. (Your doctors will talk to you about what happens in your preoperative scans – but we want you to know that some people find them quite claustrophobic – but the scans are needed for your surgery regardless of taking part in FUTURE-GB). Also, those who have metal in their body may potentially not be able to have type of scan called an MRI scan – talk to the doctors if you think you have metal in your body.

Please note: The design of this study has involved patients, their families and healthcare professionals, including brain surgeons, using their knowledge and experience at every stage of its development.

What technologies will be used in my operation?

Surgery will involve standard care and newer technologies

Standard care:

You will have an MRI scan before your operation. This can be used during the surgery to help your surgeon identify where your brain tumour is located, and what brain structures are close by. MRI stands for Magnetic Resonance Imaging. It is a type of scan that uses strong magnetic fields to produce detailed images. It is used for brain tumour surgery to obtain detailed images of your brain and specifically your brain tumour. There is no risk of radiation exposure. All MRI scans that you will receive are received by all those with a brain tumour, anything seen on these scans will be acted upon as per local NHS Trust and national guidelines.

This is combined with use of a chemical called 5-aminolevulinic acid (5-ALA), which is a drink taken a few hours before surgery. This allows the tumour cells to light up pink, when a blue light is shone on them during surgery. This has been shown to help surgeons remove more of the brain tumour, as they are able to see better the edges of the tumour and differentiate it from the surrounding normal brain. This makes sure as much of the tumour is removed as is possible, but it can never usually be totally removed.

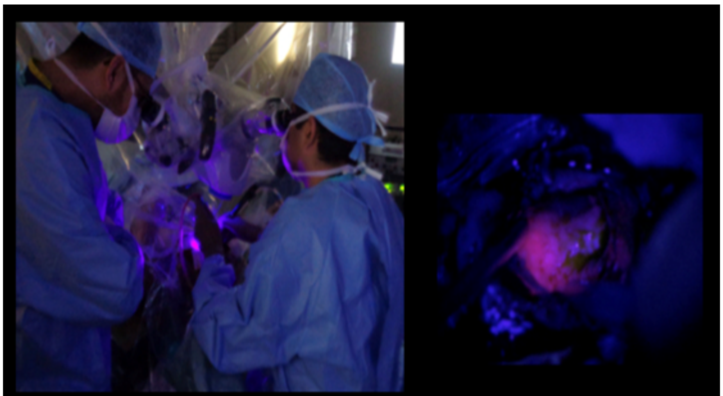

Surgeons using microscope equipped with blue light | Appearance of tumour with 5ALA under blue light

FUNDED BY NIHR National Institute for Health Research | OCTRU | SITU | UNIVERSITY OF OXFORD | Imperial College London

For peer review only - http://bmjopen.bmj.com/site/about/guidelines.xhtml

Oxford University Hospitals
NHS Foundation Trust

Newer technologies:

1) Diffusion Tensor Imaging (DTI) is an MRI technology which allows the surgeons to have a scan of all the nerve fibres which are involved in movement, speech etc. around a tumour. This means that when removing the tumour, the surgeon knows more easily where these are based on your DTI scan and can avoid them.

2) Intraoperative Ultrasound is a technology that uses high frequency sound waves to create an image of the brain tumour during the operation. The ultrasound provides "live" pictures of your tumour as surgery progresses and tumour is removed. The surgeon can use this as many times as necessary during your surgery. The ultrasound is the same as that used to provide a picture of a baby inside a pregnant woman.

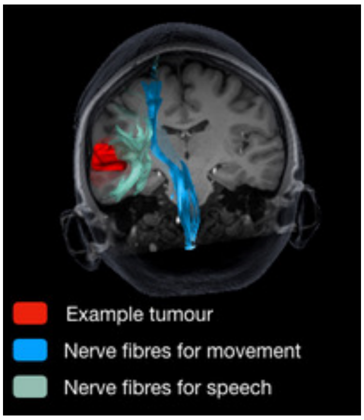

Both these technologies are safe and have been used in brain tumour surgery for a number of years. Your surgeon is familiar with their use and has used them during surgery. However, the benefit of using these 2 new technologies together, in addition to the present "standard of care" surgery has not been scientifically tested, or formally assessed. There are no extra drugs or chemicals used.

Further possible contacts: A researcher from the trial coordinating team may visit while you are having your operation so that we can check how the surgery is being undertaken. We will always check that you are happy for this to happen. If not, the researcher will not come into your operation. At the end of the study, we will report how well the treatments were delivered as it is important we fully understand this process.

Please note, no-one can ever be identified in any public report about the study.

There are 2 companies supporting this study by providing machinery and software to sites if they do not already have the equipment needed for this study. The companies are called BrainLab and Medtronic they are supporting doctors in the UK in using the new technologies. Neither company will be able to influence the results of the study.

What happens after my operation in FUTURE-GB?

Researchers will record information about your operation from your medical notes and directly into the study database, researchers will also collect information from your medical notes and scans when you are discharged home after your operation. The care, any tests, further outpatient appointments and any other surgery or treatments will not be changed by you agreeing to take part in FUTURE-GB. Researchers will check your medical notes for up to 6 months after your operation so find out how you are getting on – but there will be no further contact with you from the study team.

What are the benefits and risks of taking part in the study?

For those that take part in the study, your operation will be conducted by the same surgeon/surgical team whom you have already seen.

We hope the information from this study will answer the question:

Which imaging tools should be used by surgeons when removing a glioblastoma, to offer the highest chance of removing as much of the tumour as possible without causing functional problems, and therefore keeping a good quality of life?

For peer review only - http://bmjopen.bmj.com/site/about/guidelines.xhtml

Oxford University Hospitals
NHS Foundation Trust

We cannot promise the study will help you directly, but the information we get has the potential to be of benefit, potentially allowing more of your tumour to be removed safely.

The risks relating to the brain tumour surgery itself will be discussed with you in detail as part of the standard, routine consent for an operation. We do not think that being part of this study will change any of the risks of the operation but this is one of the things we be will be studying. The technologies will however add some time to the scan before your operation and during your operation.

We are undertaking this study because the extra imaging tools add significant costs to NHS treatment, and therefore we have also been funded to identify whether they provide a real benefit for people with brain tumours.

People sometimes feel uncomfortable answering certain questions about their health, or may be unable to answer. If you, or the person you nominate to answer for you, feel uncomfortable at any point, then you do not have to answer the questions.

Who will know that I am taking part?

The only people who will know that you are taking part in this study are the members of the research team and the healthcare professionals involved in your care. You can tell anyone you would like to that you are taking part.

The only people in the University of Oxford who will have access to information that identifies you will be people who need to contact you to about the study, or review the data. The people who analyse the information will not be able to identify you and will not be able to find out your name or contact details. Also, your de-identified scans will be reviewed by members of the research team and the companies providing the extra imaging technologies. The images will be transferred using cloud servers, however, nothing that could identify you will be included.

Representatives from the Sponsor, relevant regulatory organisations and [insert local Trust] may also need access to monitor or audit the study to ensure that the research is complying with applicable regulations.

Paperwork that is completed by you, the research team, or the treating clinical team, will be sent securely to the study team managing the FUTURE-GB study that are based at the University of Oxford.

Will my details be kept confidential?

Yes. All information collected about you and from you during the course of the research, including from your medical records, will be kept strictly confidential. Everyone who takes part in the study will be assigned a code number and all of the data relating to each person will be held on a computer database and will only be linked to that code number, and not to people's names or addresses. The study team will record into the study database your name, date of birth, NHS or CHI number, Hospital number and your email address. These details will allow the central study team and the local teams to ensure they are collecting data on the correct person. Your email address will only be used to allow you to complete the consent form at home, although this can also be done at the hospital, and to send you a copy of your consent form for your records. Your NHS or CHI number will be used to look up your status 6 months after agreeing to take part.

We will ask you for your permission for individuals from the University of Oxford and Imperial College London, and the regulatory authorities, to have access to your medical notes and data. This is in order that they may conduct checks on the study data that has been collected and to ensure all the study data has been completed correctly. We will also ask you for your permission to allow appropriate individuals from the NHS Trust that you are being approached at to also undertake this review.

At the end of the study, all of the data will be de-identified so that no-one can be identified. This de-identified data will be shared so that more researchers can gain a deeper understanding about patients

For peer review only - http://bmjopen.bmj.com/site/about/guidelines.xhtml

Oxford University Hospitals
NHS Foundation Trust

who have had surgery for glioblastoma. It may be shared with other researchers around the world and with commercial organisations but this information will not identify you, and will not be combined with other information in a way that could identify you. The information will only be used for the purpose of healthcare research, and cannot be used to contact you, nor will it affect your care.

In line with what happens in the NHS, the only situation that confidentiality would need to be broken would be if you told a health professional or research team member of something that could result in harm to yourself or others.

What will happen to my data?

Research is carried out in the public interest. The University of Oxford, as Sponsor, is the data controller. This means that we, as University of Oxford researchers, are responsible for looking after your information as part of FUTURE-GB, and using it properly. We will use the minimum possible personally-identifiable information, and this will be kept for 12 months after the study has finished. Non-identifiable research data and any research documents with personal information, will be stored securely at the University of Oxford for a maximum of 5 years after the end of the study, as part of the research record.

We will be using information from you and your medical records in order to carry out this study. The Oxford University Hospitals NHS Foundation Trust will use your NHS number and contact details to get in touch with you, and to make sure that relevant study information is recorded from your care records. They will keep your identifiable information safely for 12 months after the study has finished. Consent forms and study documents held at [local NHS Trust name] will be archived securely, in accordance with their local procedures.

Data protection regulation provides you with control over your personal data and how it is used. When you agree to your information being used in research, however, some of those rights may be limited, as we need to manage your information in specific ways in order for the research to be reliable and accurate. Further information about your rights with respect to your personal data, is available at https://compliance.web.ox.ac.uk/individual-rights

You can find out more about how we use your information by contacting the FUTURE-GB study team on: futuregb@nds.ox.ac.uk.

What will happen if I don't want to carry on with the study?

You are free to withdraw from taking part in the study at any time without giving a reason. Please remember, it is your decision to take part. If you agree to take part now, but you change your mind during the study, this will not change the standard of care you receive from the NHS. If you were to decide to stop taking part in the study at any time, any data collected on you would be kept. You would not be contacted about the study again or have any further data collected.

What happens at the end of the study?

We will share the results with healthcare researchers and professionals to improve future patient care. Also, we will present them in research reports, at scientific conferences, and publish them in scientific journals, and publish them on the study website futuregb.octru.ox.ac.uk.

We will not include any data that could identify you in the results. If the funders of this research ask us to make the study data available for other researchers, we will first de-identify your information (i.e. we will take your name and other identifying details out) so that you cannot be identified.

For peer review only - http://bmjopen.bmj.com/site/about/guidelines.xhtml

Oxford University Hospitals
NHS Foundation Trust

Who is organising and funding the research?

The University of Oxford is the Sponsor and is organising this study. It is being conducted by a research team led by Prof Puneet Plaha, Consultant Neurosurgeon at the Oxford University Hospitals NHS Foundation Trust and the University of Oxford, and Miss Sophie Camp and Prof. Dipankar Nandi (both Consultant Neurosurgeons at Imperial College Healthcare NHS Trust).

The National Institute of Health Research – Health Technology Assessment programme is funding the study. The funding for the NIHR comes from the UK Government.

Who has approved this study?

A panel of independent researchers and patient representatives, as well as a Research Ethics Committee (REC Reference 20/LO/0840) have reviewed and approved this study.

For peer review only

What if I have concerns?

The University of Oxford, as the study sponsor, has appropriate insurance in place in the unlikely event that you suffer any harm as a direct consequence of your participation in this study.

If you have any concerns or complaints about any aspect of the study, please contact the FUTURE-GB research team using the details below. You can also contact the University of Oxford Research Governance, Ethics & Assurance office on 01865 616480 or by email on ctrg@admin.ox.ac.uk.

If you would prefer to speak with someone who is not involved in the study, then please contact the Patient Advice and Liaison Service (PALS). PALS is a confidential NHS service that can provide you with support for any complaints or queries you have regarding the care you receive as an NHS patient. However, PALS cannot provide information about this research study.

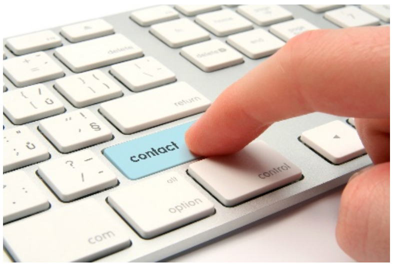

PALS phone number: [local PALS phone number]

PALS email: [local PALS email]

You can also contact your local clinical team directly:

<local PI/research team name and contact details>

If you have any questions about the study, please contact the FUTURE-GB team on:

Email: futuregb@nds.ox.ac.uk   Telephone: 07917 101 649

Postal address: FUTURE-GB study, Botnar Research Centre, Nuffield Orthopaedic Centre, Windmill Road, Oxford, OX3 7LD.

Further information can be found on our study website – futuregb.octru.ox.ac.uk

Thank you for reading this information leaflet and considering taking part.

FUNDED BY NIHR | National Institute for Health Research   OCTRU   SITU   UNIVERSITY OF OXFORD   Imperial College London

LOCAL TRUST LOGO

Functional and Ultrasound guided Resection of Glioblastoma – the FUTURE-GB study – Stage 2 - Randomised Controlled Trial

Patient Information Leaflet

Invitation to join the FUTURE-GB study

We would like to invite you to take part in a research study (also called a clinical trial).

Before you decide whether to take part or not, it is important that you understand why we are doing this study and what it will involve.

Please take time to read the following information and talk to others about the study. If anything is unclear, or if you would like more information, please ask a member of the study team who will be happy to answer any questions.

What is the purpose of this study?

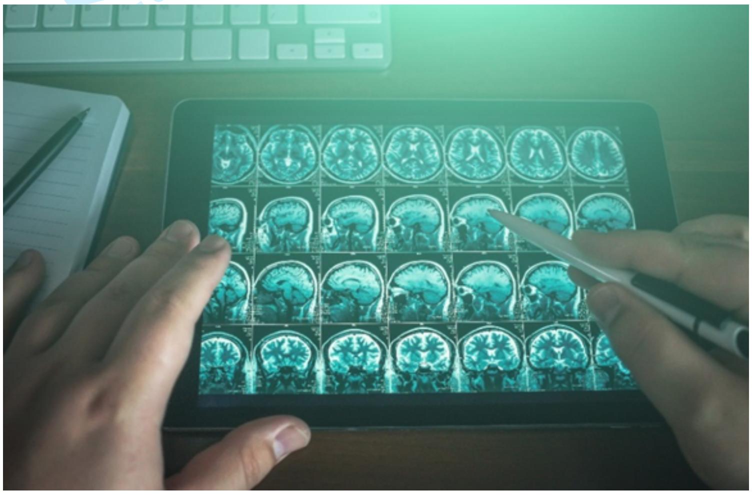

There are many different types of brain tumours. These can vary in how quickly they grow and what symptoms they cause. Studies have shown that when a brain tumour is growing quickly it is better to remove as much tumour as possible. Being able to do this without causing damage to the parts of the brain that are involved in things such as speaking and moving, surgeons need to be able to see clearly parts of the brain during surgery, using accurate imaging. This has led to an increase in the use of imaging (such as ultrasound and MRI scans) during operations. However, we don't know if all the extra imaging tools do definitely make a difference.

We have been funded by the National Institute of Health Research (NIHR) which receives its funding from the UK Government to find out whether some of these additional imaging tools available make a positive difference to quality of life for people with fast growing brain tumours who have surgery. We will also be looking to see if these imaging tools used during an operation mean people with a brain tumour:

- have a better quality of life?
- if it takes more/less time for their tumour to come back
- if they have more/fewer complications from the surgery.

For peer review only - http://bmjopen.bmj.com/site/about/guidelines.xhtml

LOCAL TRUST LOGO

This study will attempt to give a definite answer, so that surgeons know which imaging tools they should use during an operation to enable as much tumour as possible to be removed safely, whilst minimising the risks of damaging brain function and hence affecting quality of life.

The imaging tools that will be used in this study are available and in use across the NHS, and have been shown to be safe. However, no one knows if using them together will have a definite positive effect on outcome for those with a brain tumour.

Who is taking part and why have I been invited to take part?

Half of the people taking part in the study will have standard NHS imaging (scans), and the other half have standard NHS imaging (scans) and some additional imaging (scans).

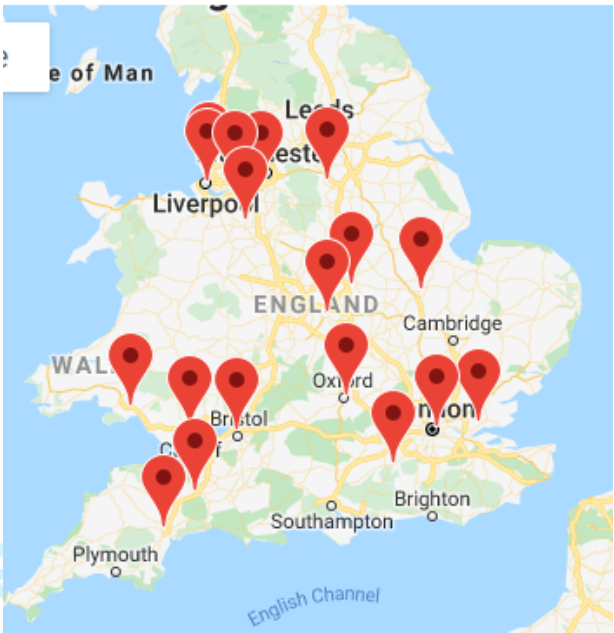

will

We want to enrol 357 people aged 18 - 70 from approximately 15 neurosurgical centres in the UK. will have surgery in your local neurosurgical unit, which is participating in this study.

You

You have been invited to take part because your scan suggests you have a brain tumour, which comes from the brain itself, rather than from a cancer elsewhere in the body which has spread to brain. Your scan also suggests the tumour is likely aggressive, called a glioblastoma, or high grade tumour.

brain

the

to be

Do I have to take part in this study?

No, you are under no obligation to take part in the study. Deciding not to will not affect the treatment/care you receive from your team. It is up to you to decide whether to take part or not. Please keep this leaflet and use it as it may to help you make your decision. If you decide to take part, you will be asked to sign another consent form, as well as that used for your NHS operation.

If you choose not to join the study, you will receive the routine NHS treatment, agreed by your local treating team of healthcare professionals, in accordance with standard NHS practice using the imaging your treating team deems appropriate. A note will be made of your age and gender, so that we can find out who decides not to take part. You cannot be identified from this data. A researcher may ask you if you would be happy to give a reason for not wanting to take part in the study. Giving this information is entirely voluntary.

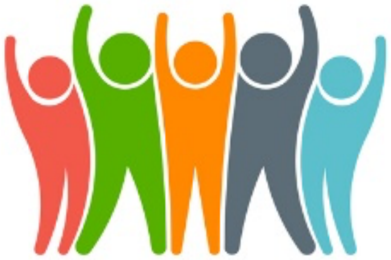

Should you decide to take part, you are still free to withdraw at any time and without giving a reason. This will not affect the standard of care you receive, as either an inpatient or an outpatient.

What will happen if I take part?

If you are happy to take part in this study, a researcher will ask you some simple questions and check your medical history to confirm that you are eligible.

Initial assessment: If you are eligible, you will be asked to sign and date a consent form for the study. We will also ask you to complete some short questionnaires about your health, the activities you are able to

FUNDED BY NIHR | National Institute for Health Research   OCTRU   SITU   UNIVERSITY OF OXFORD   Imperial College London

For peer review only - http://bmjopen.bmj.com/site/about/guidelines.xhtml

LOCAL TRUST LOGO

carry out, and about your quality of life. These questionnaires should take you no more than 10-15 minutes to complete. (The questionnaires are electronic – but sometimes you may be given a paper questionnaire to complete, if the electronic system is not available). A researcher will also ask you to complete a brain activity and a recall test, check the strength in your arms and legs and talk to you about how you care for yourself.

Most significantly for those that agree to take part in FUTURE-GB the time taken for your preoperative scans (which you will have by being in the study or not) will perhaps take another 5 minutes. Your operation may also be slightly longer due to the technologies being used – this might perhaps extend it by 15 minutes. (Your doctors will talk to you about what happens in your preoperative scans – but we want you to know that some people find them quite claustrophobic –the scans are needed for your surgery regardless of taking part in FUTURE-GB). Also, those who have metal in their body may potentially not be able to have type of scan called an MRI scan – talk to the doctors if you think you have metal in your body.

Imaging (scans) allocation: You will then be randomly allocated to an imaging group by a computer, which has no information about you as an individual, i.e. allocation is by chance. You will have an equal chance of being allocated to either group, like the toss of a coin. There is a 50% chance that you will be put into the group in which the additional imaging tools will be used during your operation, in addition to the standard techniques, and a 50% chance that you will be in the group where the surgeon uses the present, standard imaging tools.

The random allocation is important because this way, we can test the different imaging tools fairly and nobody can influence into which group you are placed. If you enrol in the study, your healthcare and research teams will not be able to affect which imaging tools will get used in your operation and you will not be able to choose. You will not be aware into which arm of the study you have been allocated, just in case you answer the questionnaires differently based on the imaging used in your operation.

Please note: The design of this study has involved patients, their families and healthcare professionals, including brain surgeons, using their knowledge and experience at every stage of its development.

The FUTURE-GB study aims to find out if the new technologies do, or do not, improve the quality of life of those treated for a brain tumour. We need to know how you are, and your abilities during the study, before and after your operation. We are therefore asking everyone who agrees to take part to nominate a good friend/relative/partner to complete the same questionnaires at the same time as you. They will be asked the same questions as you are asked but they will be asked to answer what their opinion is of <u>your</u> health and abilities.

If you become unable to answer the questions at some point during the study, we would like to know the answers from your good friend/relative/partner to help us identify any changes in your quality of life up to that point in the study. It is helpful to have both assessments to be sure we know about any change in your health status during the study. This is why we would ask both you and your friend/relative/partner to complete the questionnaires throughout the study. The responses that you and your friend/relative/partner give will be used by the trial team to understand the impact of the new technologies on quality of life.
Note: All the data that you and your friend/relative/partner gives will be used by the trial team.

What technologies will be used in my operation?

If you are allocated to the standard techniques arm of the study, your surgeon will use the standard NHS imaging tools.

You will have a Magnetic Resonance Imaging (MRI) scan before your operation. This can be used during the surgery to help your surgeon identify where your brain tumour is located, and what brain structures are close by. This is a type of scan that uses strong magnetic fields to obtain detailed images

FUNDED BY NIHR | National Institute for Health Research OCTRU SITU UNIVERSITY OF OXFORD Imperial College London

For peer review only - http://bmjopen.bmj.com/site/about/guidelines.xhtml

LOCAL TRUST LOGO

of your brain and specifically your brain tumour. There is no risk of radiation exposure. All MRI scans that you will receive are received by all those with a brain tumour, anything seen on these scans will be acted upon as per local NHS Trust and national guidelines.

This is combined with use of a chemical called 5-aminolevulinic acid (5-ALA), which is a drink taken a few hours before surgery. This allows the tumour cells to light up pink, when a blue light is shone on them during surgery. This is known to help surgeons remove more of the brain tumour, as they are able to see better the edges of the tumour compared to the rest of the brain, making sure as much of the tumour is removed as is possible in each individual case.

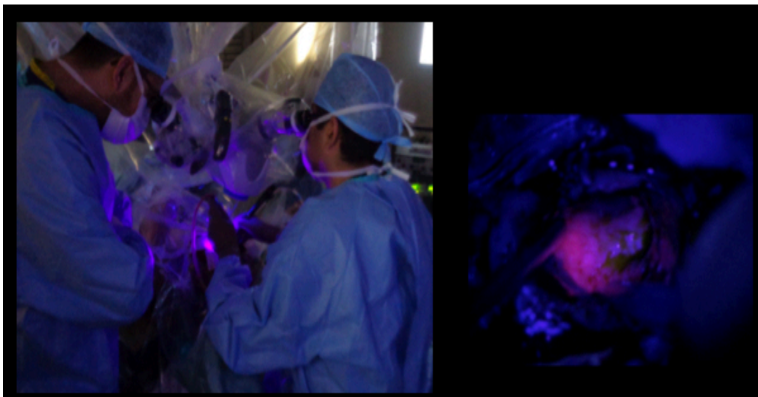
Surgeons using microscope equipped with blue light
Appearance of tumour with 5ALA under blue light

If you are allocated to the group that will use the additional imaging tools, your surgeon will undertake all of what is listed above, together with the additional imaging tools. You will have a slightly longer MRI scan (additional 5 minutes) and have the imaging outlined below also undertaken as part of your operation.

Diffusion Tensor Imaging (DTI) – this is an imaging tool that allows the surgeons to have a scan of all the nerve fibres which are involved in movement, speech etc. around a tumour. This means that when removing the tumour the surgeons potentially know, more accurately, where these are located specifically in your brain from this scan and so can avoid them. This scan is taken using an MRI machine – this is why your MRI scan would be 5 minutes longer.

Intraoperative Ultrasound – this is a technology whereby high frequency sound waves are used to create an image of the brain tumour during the operation. The ultrasound provides "live" pictures of your tumour as surgery progresses and tumour is removed. The surgeon can use this as many times as necessary during your surgery. The ultrasound is the same as that used to provide a picture of a baby inside a pregnant woman.

Both these imaging tools are safe and are used in brain tumour surgery already, but their benefit has not been formally assessed. There are no extra drugs or chemicals used for the additional imaging tools.

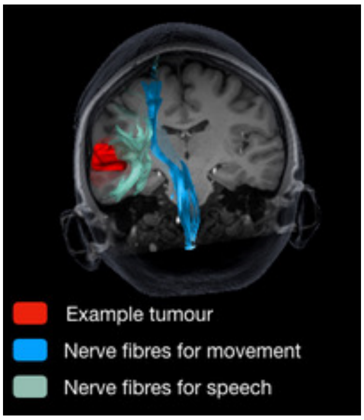

There are 2 companies supporting this study by providing machinery and software to sites if they do not already have the equipment needed for this study. The companies are called BrainLab and Medtronic they are supporting doctors in the UK in using the new technologies. Neither company will be able to influence the results of the study.

Further possible contacts: A researcher from the trial coordinating team may visit while you are having your operation so that we can check how the surgery is being undertaken. We will always check that you are happy for this to happen. If not, the researcher will not come into your operation. At the end of the study, we will report how well the treatments were delivered as it is important we fully understand this process.

For peer review only - http://bmjopen.bmj.com/site/about/guidelines.xhtml

LOCAL TRUST LOGO

What happens after my operation in FUTURE-GB?

If you take part in the study, we will not know whether the additional imaging tools are helpful or not until many months after your operation.

As part of the study you will be asked to complete some further questionnaires. The questionnaires ask about you, your health and activity, and your quality of life. We ask that you complete the questionnaires before you have surgery, when you leave hospital, at 6 and 12 weeks after surgery, and then every 3 months, for a maximum of 2 years. These time points are when you would usually be coming back to the hospital for routine NHS care, and it may be possible to complete them at these appointments. However, they will also be emailed to you for online completion. In addition, either when you are at the hospital for your outpatient appointments, or via telephone at those timepoints, a researcher will also ask you to complete a brain activity and a recall test, and talk to you about how you care for yourself, these will be the same tests and questions as before your operation.

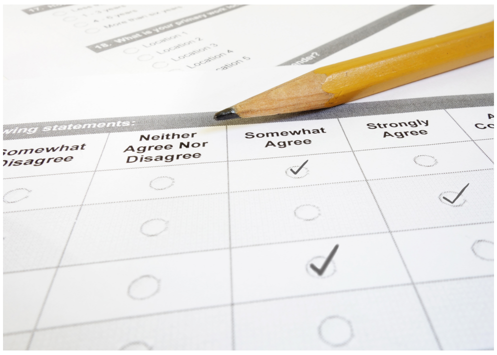

The questionnaires should take no more than 10 minutes to complete on paper/online, or over the telephone. The questionnaires are all completed online, but speak to your researcher if you want to take part but do not have access to the internet.

Please note, the care, any tests, further outpatient appointments and any other surgery or treatments will not be changed by you agreeing to take part in FUTURE-GB. Researchers will check your medical notes for up to 24 months after your operation to find out how you are getting on, and the trial team will use your details to send out the follow-up questionnaires.

Please note if you are deemed to lose capacity at any point during the study, you will not be asked to complete any further questionnaires.

What are the benefits and risks of taking part in the study?

For those that take part in the study, your operation will be conducted by the same surgeon/surgical team whom you have already met.

The information from this study we hope will answer the question:

Which imaging tools should be used by surgeons when removing a glioblastoma, to offer the highest chance of removing as much of the tumour as possible without causing functional problems, and therefore keeping a good quality of life?

We cannot promise the study will help you directly, but the information we get has the potential to be of benefit, potentially allowing more of your tumour to be removed safely.

The risks relating to the brain tumour surgery itself will be discussed with you in detail as part of the standard, routine consent for an operation. We don't think that being part of this study will change any of the risks of the operation but this is one of the things we be will be studying. The technologies will however add some time to the scan you have before your operation and your operation if you are put into the group where the new technologies are used.

We are undertaking this study because we want to find out whether the extra imaging tools provide a real benefit for people with brain tumours. These add significant costs to NHS treatment, and so we need to know if they are worth the extra cost.

For peer review only

For peer review only - http://bmjopen.bmj.com/site/about/guidelines.xhtml

LOCAL TRUST LOGO

People sometimes feel uncomfortable answering certain questions about their health, or may be unable to answer. If you, or the person you nominate to answer for you, feel uncomfortable at any point, then you do not have to answer the questions.

We are not able to pay travel expenses for you to attend your follow-up sessions, however any research questions asked will be as part of your routine out-patient follow-up appointments, via email, or over the telephone.

Who will know that I am taking part?

The only people who will know that you are taking part in this study are the members of the research team and the healthcare professionals involved in your care. You can tell anyone you would like to that you are taking part.

The only people in the University of Oxford who will have access to information that identifies you will be people who need to contact you to about the study, or review the data. The people who analyse the information will not be able to identify you and will not be able to find out your name or contact details. Also, your de-identified scans will be reviewed by members of the research team and the companies providing the extra imaging technologies. The images will be transferred using secure cloud servers, however, nothing that could identify you will be included.

Representatives from the sponsor, relevant regulatory organisations and [Insert local Trust] may also need access to monitor or audit the study to ensure that the research is complying with applicable regulations.

Paperwork that is completed by you, your nominee (friend/relative/partner), the research team, or the treating clinical team, will be sent securely to the study team managing the FUTURE-GB study that are based at the University of Oxford.

We will contact your GP (doctor) to tell them that you have agreed to take part in the FUTURE-GB study. However, we do not share with them anything you answer in your study questionnaires.

Will my details be kept confidential?

Yes. All information collected about you and from you and your nominee (friend/relative/partner) during the course of the research, including from your medical records, will be kept strictly confidential. Everyone who takes part in the study will be assigned a code number and all of the data relating to each person will be held on a computer database and will only be linked to that code number, and not to people's names or addresses. The study team will record into the study database your name, date of birth, NHS or CHI number, Hospital number, address, phone number, GP name and address, and your email address. These details will allow the central study team and the local teams to ensure they are collecting data on the correct person. Your email address will only be used to send you a copy of your consent form for your records and any follow-up questionnaires. This is also the reason your address will be kept on file – in case your questionnaires need to be posted to you to complete. Your phone number will be used by the researchers to call and ask you the questions about brain activity and recall if these cannot be completed at your outpatient appointment. Your NHS or CHI number will be used to check your status 24 months after agreeing to take part. Your GPs details will be used to send a letter to your GP informing them of you taking part in this study.

We will ask you for your permission for individuals from the University of Oxford and Imperial College London, and the regulatory authorities, to have access to your medical notes and data. This is in order that they may conduct checks on the study data that has been collected and to ensure all the study data has been completed correctly. We will also ask you for your permission to allow appropriate individuals from the NHS Trust that you are being approached at to also undertake this review.

For peer review only - http://bmjopen.bmj.com/site/about/guidelines.xhtml

LOCAL TRUST LOGO

At the end of the study, all of the data will be de-identified so that no-one can be identified. This de-identified data will be shared so that more researchers can gain a deeper understanding about patients who have had surgery for glioblastoma. It may be shared with other researchers around the world and with commercial organisations but this information will not identify you, and will not be combined with other information in a way that could identify you. The information will only be used for the purpose of healthcare research, and cannot be used to contact you, nor will it affect your care.

In line with what happens in the NHS, the only situation that confidentiality would need to be broken would be if you told a health professional or research team member of something that could result in harm to yourself or others.

What will happen to my data?

For peer review only

Research is carried out in the public interest. The University of Oxford, as Sponsor, is the data controller. This means that we, as University of Oxford researchers, are responsible for looking after your information as part of FUTURE-GB, and using it properly. We will use the minimum possible personally-identifiable information, and this will be kept for 12 months after the study has finished. Non-identifiable research data and any research documents with personal information, will be stored securely at the University of Oxford for a maximum of 5 years after the end of the study, as part of the research record.

We will be using information from you and your medical records in order to carry out this study. The [local NHS Trust name] will use your NHS number and contact details to get in touch with you, and to make sure that relevant study information is recorded from your care records. They will keep your identifiable information safely for 12 months after the study has finished. Consent forms and study documents held at [local NHS Trust name] will be archived securely, in accordance with their local procedures.

Data protection regulation provides you with control over your personal data and how it is used. When you agree to your information being used in research, however, some of those rights may be limited, as we need to manage your information in specific ways in order for the research to be reliable and accurate. Further information about your rights with respect to your personal data, is available at https://compliance.web.ox.ac.uk/individual-rights

You can find out more about how we use your information by contacting the FUTURE-GB study team on: futuregb@nds.ox.ac.uk.

What will happen if I don't want to carry on with the study?

You are free to withdraw from taking part in the study at any time without giving a reason. Please remember, it is your decision to take part. If you agree to take part now, but you change your mind during the study, this will not change the standard of care you receive from the NHS. If you were to decide to stop taking part in the study at any time, any data collected on you would be kept. You would not be contacted about the study again or have any further data collected about you from your medical records. If you withdraw or lose capacity please note we will not continue to contact your nominee (proxy).

What happens at the end of the study?

We will share the results with healthcare researchers and professionals to improve future patient care. Also, we will present them in research reports, at scientific conferences, and publish them in scientific journals, and publish them on the study website: futuregb.octru.ox.ac.uk.

For peer review only - http://bmjopen.bmj.com/site/about/guidelines.xhtml

LOCAL TRUST LOGO

We will not include any data that could identify you in the results. If the funders of this research ask us to make the study data available for other researchers, we will first de-identify your information (i.e. we will take your name and other identifying details out) so that you cannot be identified.

Who is organising and funding the research?

The University of Oxford is the Sponsor and is organising this study. It is being conducted by a research team led by Prof. Puneet Plaha, Consultant Neurosurgeon at the Oxford University Hospitals NHS Foundation Trust and the University of Oxford, and Ms Sophie Camp and Prof. Dipankar Nandi (both Consultant Neurosurgeons at Imperial College Healthcare NHS Trust).

The National Institute of Health Research – Health Technology Assessment programme is funding the study. The funding for the NIHR comes from the UK Government.

Who has approved this study?

A panel of independent researchers and patient representatives, as well as a Research Ethics Committee (REC Reference 20/LO/0840) have reviewed and approved this study.

What if I have concerns?

The University of Oxford, as the study sponsor, has appropriate insurance in place in the unlikely event that you suffer any harm as a direct consequence of your participation in this study.

If you have any concerns or complaints about any aspect of the study, please contact the FUTURE-GB research team using the details below. You can also contact the University of Oxford Research Governance, Ethics & Assurance office on 01865 616480 or by email on ctrg@admin.ox.ac.uk.

If you would prefer to speak with someone who is not involved in the study, then please contact the Patient Advice and Liaison Service (PALS). PALS is a confidential NHS service that can provide you with support for any complaints or queries you have regarding the care you receive as an NHS patient. However, PALS cannot provide information about this research study.

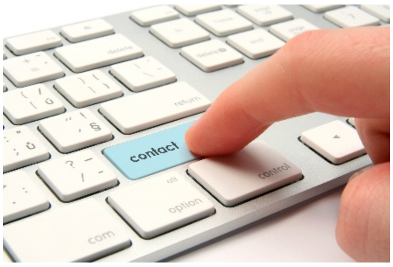

PALS phone number: <Insert local PALS number>

PALS email: <insert local PALS email address>

You can also contact your local clinical team directly:

<local PI/research team name and contact details>

If you have any questions about the study, please contact the FUTURE-GB team on:

Email: futuregb@nds.ox.ac.uk Telephone: 07917 101 649

Postal address: FUTURE-GB study, Botnar Research Centre, Nuffield Orthopaedic Centre, Windmill Road, Oxford, OX3 7LD.

Further information can be found on our study website – futuregb.octru.ox.ac.uk

Thank you for reading this information leaflet and considering taking part.

For peer review only - http://bmjopen.bmj.com/site/about/guidelines.xhtml

LOCAL TRUST LOGO

Functional and Ultrasound guided Resection of Glioblastoma – the FUTURE-GB study – This is for those that are asked to consider supporting a potential participant in the Future-GB study

Partner/Relative/Friend (Proxy) Information Leaflet

Invitation to join the FUTURE-GB study

You are receiving this information leaflet as a friend/relative/partner of yours has been approached to take part in this study. As part of the study they are asked to nominate a friend or relative who would be willing to answer some questionnaires about them (a 'proxy'). As the nominated person, we would like to invite you to take agree to part in a research study (also called a clinical trial), which for you will only involve answering questionnaires.

Before you decide whether to take part or not, it is important that you understand why we are doing this study and what it will involve.

Please take time to read the following information and talk to others about the study. If anything is unclear, or if you would like more information, please ask a member of the study team who will be happy to answer any questions.

What is the purpose of this study?

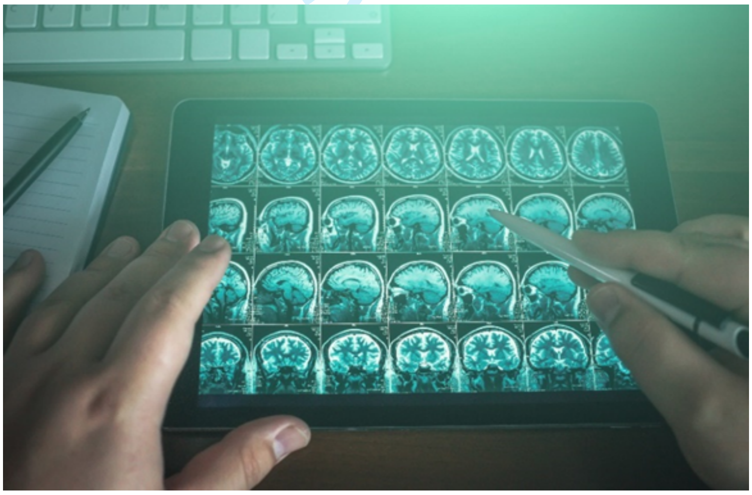

There are many different types of brain tumours. They can vary in how quickly they grow and what symptoms they cause. Studies have shown that when a brain tumour is growing quickly it is better to remove as much tumour as possible. Being able to do this without causing damage to the parts of the brain that are involved in things such as speaking and moving, surgeons need to be able to see clearly parts of the brain during surgery using accurate imaging. This has led to an increase in the use of imaging (such as ultrasound

For peer review only - http://bmjopen.bmj.com/site/about/guidelines.xhtml

LOCAL TRUST LOGO

and MRI scans) during operations. However, we don't know if all the extra imaging tools do definitely make a difference.

We have been funded by the National Institute of Health Research (NIHR) which receives its funding from the UK Government, to find out whether some of these additional imaging tools available make a positive difference to quality of life for people with brain tumours who have surgery. We will also be looking to see if these imaging tools used during an operation mean people with a brain tumour:

- have a better quality of life?
- if it takes more/less time for their tumour to come back
- if they have more/fewer complications from the surgery

This study will attempt to find a definite answer, so that surgeons know which imaging tools they should use during an operation to enable as much tumour as possible to be removed safely, whilst minimising the risks of damaging brain function and hence affecting quality of life.

The imaging tools that will be used in this study are available across the NHS, and have been shown to be safe. However, no one knows if using them together will have a definite positive effect on outcome for those with a brain tumour. We would encourage you to read the Participant Information Sheet to find out more about the study.

Who is taking part and why have I been invited to take part?

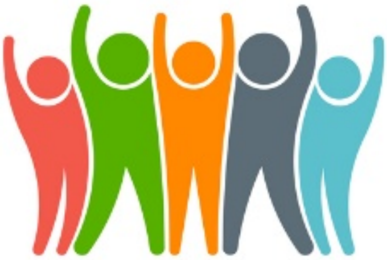

We are hoping to enrol a nominated relative/friend/partner (proxy) from each of the 357 people aged 18 -70 we plan to recruit to the trial, from approximately 15 neurosurgical centres in the UK who have agreed to take part in the FUTURE-GB study.

Specifically, the FUTURE-GB study aims to find out if the new technologies do or do not improve the quality of life of those treated for a brain tumour. We need to know how those taking part are and their abilities during the study, before and after their operation. If your friend/partner/relative becomes unable to answer the questionnaires at some point during the study, we would like to also have answers from you to help us identify any changes in their quality of life over the course of the study. However, if we need to use your answers instead of theirs – we can only do this if we know your answers at the start of the study, so that we can work out what changes have occurred. This is why we would ask both you and your friend/relative/partner to complete the questionnaires throughout the study. The responses that you and your friend/relative/partner give will be used by the trial team to understand the impact of the new technologies on quality of life

Do I have to take part in this study?

No, you are under no obligation to take part in the study. Deciding not to will not affect the treatment/care your friend/relative/partner receives. It is up to you to decide whether to take part or not. Please keep this leaflet and use it as it may to help you make your decision. If you decide to take part, you will be asked to sign a consent form.

For peer review only - http://bmjopen.bmj.com/site/about/guidelines.xhtml

LOCAL TRUST LOGO

What will happen if I take part?

If you are happy to take part in this study, a researcher will contact you to complete questionnaires before your friend/relative/partner's operation, 5 days after their operation or when they leave hospital, 6 weeks after their operation and then every 3 months after your friend/relative/partner's operation for a maximum of 2 years.

Questionnaires take no more than 10 minutes to complete on paper/online, or over the telephone.

Please note if the person who has nominated you withdraws from the study or loses their capacity to consent this will complete your involvement with the study.

What are the benefits and risks of taking part in the study?

For those that take part in the study, your friend/relative/partner's operation will be conducted by the same surgeon/surgical team whom they have already seen.

The information from this study we hope will answer the question:

Which imaging tools should be used by surgeons when removing a glioblastoma, to offer the highest chance of removing as much of the tumour as possible without causing functional problems, and therefore keeping a good quality of life?

We cannot promise the study will help your friend/relative/partner directly, but the more information we collect, the greater the potential to be of benefit, as more of the tumour may be removed.

We are undertaking this study because we want to find out whether the extra imaging tools provide a real benefit for people with brain tumours. These add significant costs to NHS treatment, and so we need to know if they are worth the extra cost.

People sometimes feel uncomfortable answering certain questions about a person's health, or may be unable to answer. If you feel uncomfortable at any point, then you do not have to answer the questions.

Who will know that I am taking part?

The only people who will know that you are taking part in this study are the members of the research team and the person who nominated you. You can tell anyone you would like to that you are taking part.

The only people in the University of Oxford who will have access to information that identifies you will be people who need to contact you to about the study, or review the data. The people who analyse the information will not be able to identify you and will not be able to find out your name or contact details. Representatives from the sponsor, relevant regulatory organisations and [local NHS Trust name] may also need access to monitor or audit the study to ensure that the research is complying with applicable regulations.

Paperwork that is completed by you, your friend/relative/partner, the research team, or the treating clinical team, will be sent securely to the study team managing the FUTURE-GB study, who are based at the University of Oxford.

FUNDED BY NIHR | National Institute for Health Research

For peer review only - http://bmjopen.bmj.com/site/about/guidelines.xhtml

LOCAL TRUST LOGO

Will the details be kept confidential?

Yes. All information collected from you during the course of the research, will be kept strictly confidential. Everyone who takes part in the study will be assigned a code number and all of the data relating to each person will be held on a computer database and will only be linked to that code number, and not to people's names or addresses. The study team will record into the study database your name, age, relationship to the study participant, address, and your email address. These details will allow the central study team and the local teams to ensure they are collecting data on the correct person. Your email address will only be used to send you a copy of your consent form for your records and any follow-up questionnaires. This is also the reason your address will be kept on file – in case your questionnaires need to be posted to you to complete.

At the end of the study, all of the data will be de-identified so that no-one can be identified. This de-identified data will be shared so that more researchers can gain a deeper understanding about patients who have had surgery for glioblastoma. It may be shared with other researchers around the world and with commercial organisations but this information will not identify you, and will not be combined with other information in a way that could identify you. The information will only be used for the purpose of healthcare research, and cannot be used to contact you, nor will it affect your care.

In line with what happens in the NHS, the only situation that confidentiality would need to be broken would be if you told a health professional or research team member of something that could result in harm to yourself or others.

What will happen to my data?

Research is carried out in the public interest. The University of Oxford, as Sponsor, is the data controller. This means that we, as University of Oxford researchers, are responsible for looking after your information as part of FUTURE-GB, and using it properly. We will use the minimum possible personally-identifiable information, and this will be kept for 12 months after the study has finished. Non-identifiable research data and any research documents with personal information, will be stored securely at the University of Oxford for a maximum of 5 years after the end of the study, as part of the research record.

The local and central FUTURE-GB study team might use your contact details to get in touch with you. They will keep your identifiable information safely for 12 months after the study has finished. Consent forms and study documents held at [local NHS Trust name] will be archived securely, in accordance with their local procedures.

Data protection regulation provides you with control over your personal data and how it is used. When you agree to your information being used in research, however, some of those rights may be limited, as we need to manage your information in specific ways in order for the research to be reliable and accurate. Further information about your rights with respect to your personal data, is available at https://compliance.web.ox.ac.uk/individual-rights

You can find out more about how we use your information by contacting the FUTURE-GB study team on: futuregb@nds.ox.ac.uk.

FUNDED BY NIHR | National Institute for Health Research · OCTRU · SITU · UNIVERSITY OF OXFORD · Imperial College London

For peer review only - http://bmjopen.bmj.com/site/about/guidelines.xhtml

LOCAL TRUST LOGO

Who is organising and funding the research?

The University of Oxford is the Sponsor and is organising this study. It is being conducted by a research team led by Professor Puneet Plaha, Consultant Neurosurgeon at the Oxford University Hospitals NHS Foundation Trust and the University of Oxford, and Miss Sophie Camp and Prof. Dipankar Nandi (both Consultant Neurosurgeons at Imperial College Healthcare NHS Trust, London).

The National Institute of Health Research – Health Technology Assessment programme is funding the study. The funding for the NIHR comes from the UK Government.

Who has approved this study?

A panel of independent researchers and patient representatives, as well as a Research Ethics Committee (REC Reference 20/LO/0840) have reviewed and approved this study.

What if I have concerns?

The University of Oxford, as the study sponsor, has appropriate insurance in place in the unlikely event that you suffer any harm as a direct consequence of your participation in this study.

If you have any concerns or complaints about any aspect of the study, please contact the FUTURE-GB research team using the details below. You can also contact the University of Oxford Research Governance, Ethics & Assurance office on 01865 616480 or by email on ctrg@admin.ox.ac.uk.

If you would prefer to speak with someone who is not involved in the study, then please contact the Patient Advice and Liaison Service (PALS). PALS is a confidential NHS service that can provide you with support for any complaints or queries you have regarding the care you receive as an NHS patient. However, PALS cannot provide information about this research study.

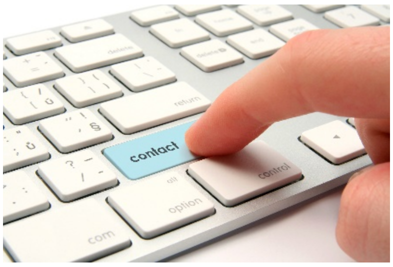

PALS phone number: <Insert local PALS number>

PALS email: <insert local PALS email address>

You can also contact your local clinical team directly:

<local PI/research team name and contact details>

If you have any questions about the study, please contact the FUTURE-GB team on:

Email: futuregb@nds.ox.ac.uk Telephone: 07917 101 649

Postal address: FUTURE-GB study, Botnar Research Centre, Nuffield Orthopaedic Centre, Windmill Road, Oxford, OX3 7LD.

Further information can be found on our study website – futuregb.octru.ox.ac.uk

Thank you for reading this information leaflet and considering taking part.

For peer review only - http://bmjopen.bmj.com/site/about/guidelines.xhtml

University of Oxford Logo

CONSENT FORM
(Stage 1-IDEAL Phase)

Imperial Logo

If you agree,
please check box

1. I confirm that I have read and understood the Information Leaflet dated DDMon20YY version XX. I have had the opportunity to consider the information, ask questions and have had these answered satisfactorily.	
2. I understand that my participation is voluntary and that I am free to withdraw at any time without giving any reason, and without my medical care or legal rights being affected. After withdrawal from the study any data collection from databases will stop.	
3. I understand that relevant sections of my medical notes and data collected during the study may be looked at by individuals from the University of Oxford and Imperial College, London, from regulatory authorities and from the NHS Trusts, where it is relevant to me taking part in this research. I give permission for these individuals to have access to my records.	
4. I consent to the research team holding my contact details so that they can contact me about the study if required. I understand these details will be held securely and destroyed 12 months after the end of the study.	
5. I understand that the information held and maintained by NHS Digital / NHS Central Register may be used to help contact me or provide information about my health status over the next 12 months.<br>I understand and give permission for my NHS/CHI number to be used for this purpose.	
6. I agree to take part in the FUTURE-GB study.	
7. I agree that my operation may be observed for quality assurance purposes by a member of the FUTURE-GB study team. Yes / No	

**Name of Participant**	**Date**	**Signature**
**Name of Person Taking Consent**	**Date**	**Signature**

For peer review only

For peer review only - http://bmjopen.bmj.com/site/about/guidelines.xhtml

University of Oxford Logo	Imperial Logo

CONSENT FORM

If you agree,
please check box

1. I confirm that I have read and understood the Information Leaflet dated DDMon20YY version XX. I have had the opportunity to consider the information, ask questions and have had these answered satisfactorily.	
2. I understand that my participation is voluntary and that I am free to withdraw at any time without giving any reason, and without my medical care or legal rights being affected. After withdrawal from the study any data collection from databases will stop.	
3. I understand that relevant sections of my medical notes and data collected during the study may be looked at by individuals from the University of Oxford and Imperial College, London, from regulatory authorities and from the NHS Trusts, where it is relevant to me taking part in this research. I give permission for these individuals to have access to my records.	
4. I consent to the research team holding my contact details so that they can contact me about the study. I understand these details will be held securely and destroyed 12 months after the end of the study.	
5. I agree to my General Practitioner (GP) being informed of my participation in the study.	
6. I understand that the information held and maintained by NHS Digital / NHS Central Register may be used to help contact me or provide information about my health status for the next 24 months. I understand and give permission for my NHS/CHI number to be used for this purpose.	
7. If I complete any questionnaires online or via the telephone regarding the FUTURE-GB study, I agree for these to be passed to the hospital I was recruited at for this study.	
8. I agree to take part in the FUTURE-GB study.	
9. I nominate the following person to be my proxy and to answer questionnaires about me during my time in the FUTURE-GB study. They will no longer be asked to provide information about me if I withdraw from the study or lose capacity. Proxy name: (INSERT NAME HERE)	
10. I agree that my operation may be observed for quality assurance purposes by a member of the FUTURE-GB study team. Yes / No	

Name of Participant **Date** **Signature**

Name of Person Taking Consent **Date** **Signature**

For peer review only - http://bmjopen.bmj.com/site/about/guidelines.xhtml

University of Oxford Logo

PROXY CONSENT FORM

Imperial Logo

	If you agree, **please check box**
1. I confirm that I have read and understood the Proxy Information Leaflet dated DDMon20YY version XX. I have had the opportunity to consider the information, ask questions and have had these answered satisfactorily.	
2. I understand that my participation is voluntary and that I am free to withdraw at any time without giving any reason, and without my relative/friend's medical care or legal rights being affected.	
3. I consent to the research team holding my contact details so that they can contact me about the study. I understand these details will be held securely and destroyed at the 12 months after the end of the study.	
4. I understand that as long as I feel able to I will complete regular questionnaires about the abilities and quality of life of the person I have been nominated to be the Proxy of. I understand if the person who nominated me withdraws or loses capacity – this will also end my participation.	
5. I agree to take part in the FUTURE-GB study as a Proxy Representative.	

Name of Participant:		
Relationship to the participant:	Partner ☐ Family member ☐ Carer ☐ Friend ☐<br>Other (please specify) ____	
Approximately how much time do you spend with the FUTURE-GB participant per week?	I am in contact with them daily ☐<br>I am in contact with them every few days ☐<br>I am in contact with them weekly ☐<br>I am in contact with them every 2 weeks ☐<br>I am in contact with them monthly ☐	
Name of Proxy Representative	Signature:	Date:
Name of Person Taking Consent	Signature:	Date:

Deleted: FUTUREGB_ProxyICF_V3.0_10Jun2021_clean.docx

For peer review only

For peer review only - http://bmjopen.bmj.com/site/about/guidelines.xhtml

Reporting checklist for protocol of a clinical trial.

Based on the SPIRIT guidelines.

Instructions to authors

Complete this checklist by entering the page numbers from your manuscript where readers will find each of the items listed below.

Your article may not currently address all the items on the checklist. Please modify your text to include the missing information. If you are certain that an item does not apply, please write "n/a" and provide a short explanation.

Upload your completed checklist as an extra file when you submit to a journal.

In your methods section, say that you used the SPIRITreporting guidelines, and cite them as:

Chan A-W, Tetzlaff JM, Gøtzsche PC, Altman DG, Mann H, Berlin J, Dickersin K, Hróbjartsson A, Schulz KF, Parulekar WR, Krleža-Jerić K, Laupacis A, Moher D. SPIRIT 2013 Explanation and Elaboration: Guidance for protocols of clinical trials. BMJ. 2013;346:e7586

		Reporting Item	Page Number
**Administrative information**			
Title	#1	Descriptive title identifying the study design, population, interventions, and, if applicable, trial acronym	1
Trial registration	#2a	Trial identifier and registry name. If not yet registered, name of intended registry	2
Trial registration: data set	#2b	All items from the World Health Organization Trial Registration Data Set	
Protocol version	#3	Date and version identifier	1
Funding	#4	Sources and types of financial, material, and other support	14
Roles and responsibilities: contributorship	#5a	Names, affiliations, and roles of protocol contributors	1
Roles and	#5b	Name and contact information for the trial sponsor	

For peer review only

responsibilities: sponsor contact information			
Roles and responsibilities: sponsor and funder	#5c	Role of study sponsor and funders, if any, in study design; collection, management, analysis, and interpretation of data; writing of the report; and the decision to submit the report for publication, including whether they will have ultimate authority over any of these activities	
Roles and responsibilities: committees	#5d	Composition, roles, and responsibilities of the coordinating centre, steering committee, endpoint adjudication committee, data management team, and other individuals or groups overseeing the trial, if applicable (see Item 21a for data monitoring committee)	
**Introduction**			
Background and rationale	#6a	Description of research question and justification for undertaking the trial, including summary of relevant studies (published and unpublished) examining benefits and harms for each intervention	4-5
Background and rationale: choice of comparators	#6b	Explanation for choice of comparators	4-5
Objectives	#7	Specific objectives or hypotheses	6
Trial design	#8	Description of trial design including type of trial (eg, parallel group, crossover, factorial, single group), allocation ratio, and framework (eg, superiority, equivalence, non-inferiority, exploratory)	6
**Methods: Participants, interventions, and outcomes**			
Study setting	#9	Description of study settings (eg, community clinic, academic hospital) and list of countries where data will be collected. Reference to where list of study sites can be obtained	6

For peer review only

Eligibility criteria	#10	Inclusion and exclusion criteria for participants. If applicable, eligibility criteria for study centres and individuals who will perform the interventions (eg, surgeons, psychotherapists)	7
Interventions: description	#11a	Interventions for each group with sufficient detail to allow replication, including how and when they will be administered	6-7
Interventions: modifications	#11b	Criteria for discontinuing or modifying allocated interventions for a given trial participant (eg, drug dose change in response to harms, participant request, or improving / worsening disease)	6
Interventions: adherance	#11c	Strategies to improve adherence to intervention protocols, and any procedures for monitoring adherence (eg, drug tablet return; laboratory tests)	
Interventions: concomitant care	#11d	Relevant concomitant care and interventions that are permitted or prohibited during the trial	
Outcomes	#12	Primary, secondary, and other outcomes, including the specific measurement variable (eg, systolic blood pressure), analysis metric (eg, change from baseline, final value, time to event), method of aggregation (eg, median, proportion), and time point for each outcome. Explanation of the clinical relevance of chosen efficacy and harm outcomes is strongly recommended	5, table 1
Participant timeline	#13	Time schedule of enrolment, interventions (including any run-ins and washouts), assessments, and visits for participants. A schematic diagram is highly recommended (see Figure)	Fig 1
Sample size	#14	Estimated number of participants needed to achieve study objectives and how it was determined, including clinical and statistical assumptions supporting any sample size calculations	9
Recruitment	#15	Strategies for achieving adequate participant enrolment to reach target sample size	8

Methods: Assignment of

For peer review only

**interventions (for controlled trials)**			
Allocation: sequence generation	#16a	Method of generating the allocation sequence (eg, computer-generated random numbers), and list of any factors for stratification. To reduce predictability of a random sequence, details of any planned restriction (eg, blocking) should be provided in a separate document that is unavailable to those who enrol participants or assign interventions	8
Allocation concealment mechanism	#16b	Mechanism of implementing the allocation sequence (eg, central telephone; sequentially numbered, opaque, sealed envelopes), describing any steps to conceal the sequence until interventions are assigned	8
Allocation: implementation	#16c	Who will generate the allocation sequence, who will enrol participants, and who will assign participants to interventions	8
Blinding (masking)	#17a	Who will be blinded after assignment to interventions (eg, trial participants, care providers, outcome assessors, data analysts), and how	8
Blinding (masking): emergency unblinding	#17b	If blinded, circumstances under which unblinding is permissible, and procedure for revealing a participant's allocated intervention during the trial	8
**Methods: Data collection, management, and analysis**			
Data collection plan	#18a	Plans for assessment and collection of outcome, baseline, and other trial data, including any related processes to promote data quality (eg, duplicate measurements, training of assessors) and a description of study instruments (eg, questionnaires, laboratory tests) along with their reliability and validity, if known. Reference to where data collection forms can be found, if not in the protocol	10
Data collection plan: retention	#18b	Plans to promote participant retention and complete follow-up, including list of any outcome data to be collected for participants who discontinue or deviate from intervention protocols	10

For peer review only

Data management	#19	Plans for data entry, coding, security, and storage, including any related processes to promote data quality (eg, double data entry; range checks for data values). Reference to where details of data management procedures can be found, if not in the protocol	10
Statistics: outcomes	#20a	Statistical methods for analysing primary and secondary outcomes. Reference to where other details of the statistical analysis plan can be found, if not in the protocol	9
Statistics: additional analyses	#20b	Methods for any additional analyses (eg, subgroup and adjusted analyses)	9
Statistics: analysis population and missing data	#20c	Definition of analysis population relating to protocol non-adherence (eg, as randomised analysis), and any statistical methods to handle missing data (eg, multiple imputation)	9
**Methods: Monitoring**			
Data monitoring: formal committee	#21a	Composition of data monitoring committee (DMC); summary of its role and reporting structure; statement of whether it is independent from the sponsor and competing interests; and reference to where further details about its charter can be found, if not in the protocol. Alternatively, an explanation of why a DMC is not needed	10-11
Data monitoring: interim analysis	#21b	Description of any interim analyses and stopping guidelines, including who will have access to these interim results and make the final decision to terminate the trial	
Harms	#22	Plans for collecting, assessing, reporting, and managing solicited and spontaneously reported adverse events and other unintended effects of trial interventions or trial conduct	
Auditing	#23	Frequency and procedures for auditing trial conduct, if any, and whether the process will be independent from investigators and the sponsor	10
**Ethics and dissemination**			
Research ethics approval	#24	Plans for seeking research ethics committee / institutional review board (REC / IRB) approval	11

For peer review only

Protocol amendments	#25	Plans for communicating important protocol modifications (eg, changes to eligibility criteria, outcomes, analyses) to relevant parties (eg, investigators, REC / IRBs, trial participants, trial registries, journals, regulators)	
Consent or assent	#26a	Who will obtain informed consent or assent from potential trial participants or authorised surrogates, and how (see Item 32)	8
Consent or assent: ancillary studies	#26b	Additional consent provisions for collection and use of participant data and biological specimens in ancillary studies, if applicable	
Confidentiality	#27	How personal information about potential and enrolled participants will be collected, shared, and maintained in order to protect confidentiality before, during, and after the trial	10
Declaration of interests	#28	Financial and other competing interests for principal investigators for the overall trial and each study site	14
Data access	#29	Statement of who will have access to the final trial dataset, and disclosure of contractual agreements that limit such access for investigators	10
Ancillary and post trial care	#30	Provisions, if any, for ancillary and post-trial care, and for compensation to those who suffer harm from trial participation	
Dissemination policy: trial results	#31a	Plans for investigators and sponsor to communicate trial results to participants, healthcare professionals, the public, and other relevant groups (eg, via publication, reporting in results databases, or other data sharing arrangements), including any publication restrictions	
Dissemination policy: authorship	#31b	Authorship eligibility guidelines and any intended use of professional writers	
Dissemination policy: reproducible research	#31c	Plans, if any, for granting public access to the full protocol, participant-level dataset, and statistical code	
**Appendices**			
Informed consent materials	#32	Model consent form and other related documentation given to participants and authorised surrogates	Supplementary files

For peer review only

Biological specimens	#33	Plans for collection, laboratory evaluation, and storage of biological specimens for genetic or molecular analysis in the current trial and for future use in ancillary studies, if applicable

None The SPIRIT Explanation and Elaboration paper is distributed under the terms of the Creative Commons Attribution License CC-BY-NC. This checklist can be completed online using https://www.goodreports.org/, a tool made by the EQUATOR Network in collaboration with Penelope.ai

For peer review only

BMJ Open

FUTURE-GB – Functional and Ultrasound guided Resection of Glioblastoma. A two-stage randomised control trial.

Journal:	*BMJ Open*
Manuscript ID	bmjopen-2022-064823.R1
Article Type:	Protocol
Date Submitted by the Author:	26-Aug-2022

Complete List of Authors:	Plaha, Puneet; Oxford University Hospitals NHS Foundation Trust, Department of Neursurgery; University of Oxford, Nuffield Department of Surgical Sciences<br>Camp, Sophie; Imperial College Healthcare NHS Trust, Neurosurgery<br>Cook, Jonathan; University of Oxford Nuffield Department of Orthopaedics Rheumatology and Musculoskeletal Sciences, Oxford Clinical Trials Research Unit & Surgical Intervention Trials Unit<br>McCulloch, Peter; Oxford University, Nuffield Department of Surgical Sciences<br>Voets, Natalie; Oxford University, Nuffield Department of Clinical Neurosciences<br>Ma, Ruichong; Oxford University Hospitals NHS Foundation Trust, Department of Neurosurgery; University of Oxford, Nuffield Department of Surgical Sciences<br>Taphoorn, Martin J.B.; Leiden University Medical Center; Haaglanden Medical Center Bronovo<br>Dirven, Linda; Leiden University Medical Center<br>Grech-Sollars, Matthew; UCL, Department of computer sciences; National Hospital for Neurology and Neurosurgery, Lysholm Department of Neuroradiology<br>Watts, Colin; University of Birmingham, Institute of Cancer and Genomic Studies; University Hospitals Birmingham NHS Foundation Trust, Department of Neurosurgery<br>Bulbeck, Helen; Brainstrust<br>Jenkinson, Michael; University of Liverpool; Walton Centre for Neurology and Neurosurgery<br>Williams, Matthew; Imperial College Healthcare NHS Trust<br>Lim, Adrian; Imperial College London<br>Dixon, Luke; Imperial College Healthcare NHS Trust, Neuroradiology<br>Price, Stephen; Cambridge University, Neurosurgery Division, Dept. Clinical Neurosciences<br>Ashkan, Keyoumars; King's College Hospital<br>Apostolopoulos, Vasileios; Oxford University Hospitals NHS Foundation Trust, Department of Neursurgery<br>Barber, Vicki; University of Oxford Nuffield Department of Orthopaedics Rheumatology and Musculoskeletal Sciences, Oxford Clinical Trials Research Unit & Surgical Intervention Trials Unit<br>GB, FUTURE; Oxford University<br>Nandi, Dipankar; Imperial College Healthcare NHS Trust, Neurosurgery; Imperial College London

**Primary Subject Heading**:	Oncology
Secondary Subject Heading:	Surgery, Patient-centred medicine
Keywords:	Neurosurgery < SURGERY, Clinical trials < THERAPEUTICS, SURGERY

FUTURE-GB – Functional and Ultrasound guided Resection of Glioblastoma. A two-stage randomised control trial.

Puneet Plaha[1,2], Sophie Camp[3], Jonathan A Cook[4], Peter McCulloch[2], Natalie Voets[5], Ruichong Ma[1,2], Martin Taphoorn[6,7], Linda Dirven[6], Matthew Grech-Sollars[8,9,10], Colin Watts[11,12], Helen Bulbeck[13], Michael D Jenkinson[14], Matt Williams[3], Adrian Lim[10], Luke Dixon[3], Stephen Price[15], Keyoumars Ashkan[16], Vasileios Apostolopoulos[1], Vicki S Barber[4], FUTURE-GB Collaborators, Dipankar Nandi[3,10]

[1]Department of Neurosurgery, Oxford University Hospitals NHS Foundation Trust, Oxford, UK
[2]Nuffield Department of Surgical Sciences, University of Oxford, UK
[3]Imperial College Healthcare NHS Trust, London, UK
[4]Oxford Clinical Trials Research Unit & Surgical Intervention Trials Unit, Nuffield Department of Orthopaedics Rheumatology and Musculoskeletal Sciences, University of Oxford, UK
[5]Nuffield Department of Clinical Neurosciences, University of Oxford, UK
[6]Department of Neurology, Leiden University Medical Center, Leiden, Netherlands
[7]Department of Neurology, Haaglanden Medical Center, The Hague, Netherlands
[8] [illegible]
[9] [illegible]
[10]Imperial College London, London, UK
[11]Institute of Cancer and Genomic Sciences, University of Birmingham, UK
[12] Department of Neurosurgery, University Hospitals Birmingham Foundation Trust, UK
[13]brainstrust = the brain cancer people, UK
[14]University of Liverpool, Liverpool, UK
[15]Department of Neurosurgery, Cambridge University Hospitals NHS Foundation Trust, Cambridge, UK
[16]Deparment of Neurosurgery, King's College Hospital NHS Foundation Trust, London, UK

FUTURE-GB collaborators:
Giles Critchley (Brighton), George Eralil, Kathrin Whitehouse (Cardiff), Anna Solth (Dundee), Paul M Brennan (Edinburgh), Chittoor Rajaraman, Shailendra Achawal (Hull), Anil Varma (Middlesborough), Robert Corns, Ganamurthy Sivakumar (Leeds), Farouk Olubajo (Liverpool), Edward McKintosh, Grainne McKenna and Dimitrios Paraskevopoulos (Barts Health), Neil Barua (Bristol), James Palmer (Plymouth), Stuart Smith (Nottingham), Paul Grundy (Southampton), Erminia Albanese, Huan Chan (Stoke-on-Trent), Damian Holliman (Newcastle), Jose Lavrador, Maria Velicu (King's College Hospital)

Corresponding Author: Puneet Plaha (puneet.plaha@ouh.nhs.uk)

Word Count: 3977 excluding abstract, references, tables and figure legends.

For peer review only

For peer review only

Abstract

Introduction
Surgery remains the mainstay for treatment of primary glioblastoma, followed by radiotherapy and chemotherapy. Current standard of care during surgery involves the intraoperative use of image-guidance and 5-ALA. There are multiple other surgical adjuncts available to the neuro-oncology surgeon. However, access to, and utilisation of these varies widely in UK practice, with limited evidence of their utility. The aim of this trial is to investigate whether the addition of Diffusion Tensor Imaging (DTI) and intraoperative ultrasound (iUS) to standard of care surgery (Intra operative Neuronavigation and 5-ALA) impacts on deterioration free survival (DFS).

Methods and Analysis
This is a two-stage randomised control trial (RCT) consisting of an initial non-randomised cohort study based on the principles of the IDEAL Stage-IIb format, followed by a statistically powered randomised trial comparing the addition of DTI and iUS to standard of care surgery. A total of 357 patients will be recruited for the RCT. The primary outcome is DFS, defined as the time to either 10-point deterioration in HRQoL scores from baseline, without subsequent reversal, progressive disease, or death.

Ethics and Dissemination
The trial was registered in the Integrated Research Application System (Ref: 264482) and approved by a UK research and ethics committee (Ref: 20/LO/0840). Results will be published in a peer reviewed journal. Further dissemination to participants, patient groups and the wider medical community will utilize a range of approaches to maximize impact.

Registration details
ISRCTN: 38834571

Keywords
Glioblastoma, IDEAL, randomised control trial, DTI, intraoperative ultrasound

For peer review only

Article Summary

Strengths of trial:

- This is a randomised control trial comparing the quality of life of glioblastoma patients undergoing standard of care surgery (Intraoperative neuronavigation and 5-ALA) vs surgery with the addition of Diffusion Tensor imaging (DTI) and intraoperative ultrasound (iUS).
- To ensure standardisation and quality control of delivery of the DTI and iUS in the randomised trial, sites will be required to enter a minimum number of patients into an initial IDEAL Stage-IIB study prior to commencing recruitment to the randomised trial.
- Patient public involvement (PPI) determined the primary outcome measure of deterioration free survival (comprising a decline in health-related quality of life, disease progression or death), rather than overall survival. DFS is considered by patients to be most pertinent.

Limitations of the trial:

- The trial recruits patients aged 18-70years who can undergo surgery to maximally resect their glioblastoma.
- There is variability of the intraoperative ultrasound machines used by trial sites (sites use machines they are familiar with). However, this reflects real world iUS usage.

For peer review only

Introduction

Glioblastoma (GB) is the most frequent and aggressive form of primary brain cancer, with an incidence of 4.64/100,000persons/year in the UK[1]. Prognosis remains extremely poor with median survival of approximately 15 months[2], and as the tumour grows, patients experience a progressive decline in health-related quality of life (HRQoL), and caregivers report high levels of distress and carer burden[3]. Resistance to treatment leads to poor survival, with high costs to the patient, relatives, society, and the economy[4,5]. Although primary brain tumours represent only 3% of all cancers, a brain tumour reduces life expectancy by an average of 20years, the highest of any cancer, and accounts for more average years of life lost than any other cancer[4,5]. GB affects adults in their economic prime, and is a leading cause of death in those under 40years, costing the economy £578M per year[4,5]. To date, there has been little progress in improving outcome including quality of life, with many trials failing to show an effect[6].

Surgery is the mainstay of treatment for GB, but optimum surgical technologies remain unclear. Surgery to resect GB is integral to maximum first line treatment, with a greater impact on survival than non-operative treatments (radiotherapy and chemotherapy)[7]. It improves symptom control, reduces dependence on dexamethasone, and increases progression free (PFS) and overall survival (OS)[8,9]. However, maximising the extent of surgical resection must be balanced against the potential risk of causing neurological deficit, and hence impacting negatively on a patient's ability to tolerate adjuvant treatments.

The desire to achieve a safe, maximal resection, particularly in eloquent regions, has led to an increase in the use of intraoperative imaging. This attempts to eliminate the error produced by brain shift, an inherent problem in navigation systems based on preoperative imaging[6], to demonstrate residual tumour at operation, and to visualise accurately relevant white matter tracts and tumour margins. Two technologies that facilitate surgical resection intraoperatively are iUS and DTI.

1. iUS accommodates for brain shift if it is linked to neuronavigation systems, allowing the surgeon to track tumour resection in real time. iUS permits multiple, real time image acquisitions, and, potentially, if navigated, at each stage, comparison with the preoperative MRI navigation sequence, to evaluate brain shift and residual disease. iUS minimally augments operative time[6], allowing precise visualization of tumour resection. It is user friendly, widely available, and a pragmatic and cost-effective alternative to intraoperative MRI, which is prohibitively expensive for many UK units. iUS, and more recently navigated iUS, has a long history in brain tumour surgery[10,11], facilitating/extending resection[12–16], and improving survival[17]. It has also been evaluated with respect to histology[18,19]. However, there is a learning curve, and image interpretation, especially during resection, can be challenging[10]. iUS demonstrates residual tumour in real time. Indeed, it has been reported that navigated iUS and 5-ALA provide different information of tumour extent, and when combined, enhance extent of resection[20]. Despite this, there are no randomised trials assessing its efficacy.

2. DTI is a special magnetic resonance imaging (MRI) technique that can identify the location of white matter nerve tracts important for speech/language/visual/motor

For peer review only

functions. The location of white matter fibre pathways is the most frequent reason why surgery is halted early, to avoid compromising patient function[21]. DTI is the only method available to visualise functionally important white matter tracts in the vicinity of a tumour before surgery and can be fused with standard intraoperative navigation systems to enable visualisation of the spatial location of the tracts during surgery, allowing removal of tumour in close proximity. The usefulness of DTI in brain tumour surgery has recently been reviewed[21]. Intraoperative visualisation of DTI is reported to contribute to maximising safe resection[22–24], reducing visual field deficits[25], and predicting long term language problems after surgery[26]. A single centre randomised control trial (RCT), comparing DTI vs no-DTI, showed that DTI led to significantly better gross total resection (GTR) rates, a lower risk of movement loss, and improved life expectancy[27]. Furthermore, DTI-informed awake surgery reduced the occurrence and severity of behavioural problems postoperatively, leading to faster recovery, and shorter hospital stay[28]. DTI requires the collection of additional MRI data, specialist software for analysis, and detailed knowledge of white matter anatomy and function. In addition, tract visualisation may be restricted where there is peritumoural oedema. As a result, there is only limited data available on the sensitivity and specificity of DTI in GB surgery, particularly with reference to its value as an intraoperative tool and in predicting DFS.

There are wide variations of surgical standard of care across the UK. A survey of all 24 adult UK neurosurgical centres (telephone and email survey conducted in 2018 by the Oxford researchers), showed wide variation in the use of technologies employed during GB resection. Whilst all centres employ standard neuronavigation and 5-ALA, only 75% have access to iUS, 62% to DTI, and 16% to an intraoperative MRI scanner. It remains unclear which technologies should be employed intraoperatively, without worsening neurological function. Indeed, most of these technologies are not regularly used for tumour resection, with surgeons unclear of the efficacy of each, and what is the optimum combination. A recent Cochrane review emphasized the lack of high-quality evidence to support the use of any specific intraoperative imaging technology[29]. The National Institute for Health and Care Excellence (NICE) guidance[3] has suggested that the available range of intraoperative technologies are considered, as appropriate, in addition to standard techniques, for tumour resection.

The Functional and UlTrasound gUided Resection of GlioBlastoma – FUTURE-GB trial was developed in collaboration with the Society of British Neurosurgeons (SBNS) and multiple GB patient advocate groups to try and address some of the deficits in knowledge regarding the utility of addition surgical adjuncts. FUTURE-GB aims to evaluate the impact of DTI and iUS in addition to standard of care techniques with a view to providing high-quality evidence to shape standard practice in the future.

Methods and Analysis

Trial design and setting

For peer review only

FUTURE-GB is a two-stage trial (figure 1). Patient and public involvement (PPI) actively informed the rationale, design and development of the protocol and patient facing materials of the trial.

Stage-1: non-randomised multicentre learning and evaluation stage (IDEAL Stage IIB trial)
Stage-1 is a non-randomised, multicentre learning and harmonisation stage in which quality control measures and mentoring will be employed, to improve and evaluate standards of practice based on the principles of an IDEAL Framework Stage-IIB study[30,31]. It will evaluate standard care surgery with the addition of DTI imaging and the ultrasound imaging during the operation. This will ensure that the surgeons using the technologies to be employed in the RCT demonstrate acceptable expertise in delivering the new approach prior to proceeding with the randomisation stage. This stage ensures standardisation of the use of the technologies across all trial centres by expert mentoring, and will evaluate quality of delivery, including monitoring of the learning curve for the group as a whole.

Stage-1 is divided into 3 components:

1. Pre-trial Webinar
2. IDEAL Stage-IIB (Quality assurance, Mentoring and Trial centres evaluation)
3. End of Stage-1, Pre-Stage-2 RCT, each participating centre will have a data workflow review with the Lead Investigators to review the cases completed in Stage-1.

The IDEAL Framework Stage-IIB trial will comprise the following:

- Mentoring for local site surgeons.
- Quality assurance of operative procedure.

Mentoring by the CI and Lead Investigators will be provided through visits to participating centres and frequent meetings, together with a helpline for individual advice sessions from the CI and Lead Investigators and co-applicants, as appropriate. Neurosurgeons will contribute data to ensure standardisation of the protocol and acceptable expertise in delivering the new approach (supplementary files1,2). This will be evaluated using the following metrics: operation length; successful use of DTI neuronavigation and iUS to achieve maximal safe tumour resection without major neurological deficit; and extent of tumour resection assessed on postoperative MRI scan. The number of cases required for this may vary but is expected to be small (up to 5 cases) as most surgeons are already familiar with the component techniques and are not anticipated to require substantial assessment. Ensuring all participating surgeons are ready to take part will minimize performance bias in Stage-2 and ensure standardisation of intraoperative technique.

Stage-2: prospective, Stage-III, multicentre RCT with internal pilot
This is a parallel group two arm, multicentre, RCT. Patients who agree to take part will be allocated by chance. The trial will enrol 357 newly diagnosed patients with GB and will randomly allocate them to receive either surgical resection with standard methods without US and DTI, or this surgery with the addition of US and DTI, as well as standard tools. Patients will not know into which group they have been placed, nor will the research team assessing them before and after surgery. Patients will be recruited from at least 15 NHS hospitals that routinely undertake GB surgery and have access to these tools. The trial will be embedded within existing care pathways. After agreeing to take part, participants will be

For peer review only - http://bmjopen.bmj.com/site/about/guidelines.xhtml

asked to complete questionnaires (supplementary files3-7) about their health-related quality of life (HRQoL, reflecting symptoms as well as physical, emotional and psychological functioning. They will also have a brief physical and cognitive/functional assessment before their surgery. Afterwards, the questionnaires and assessments will be repeated, before leaving hospital, and at three monthly intervals until 24months after randomisation. These will be combined with planned hospital visits. OS will also be recorded. See Figure 1 for a Flowchart of the trial.

Population: 357 participants with GB suitable for maximal, safe resective surgery (attempted GTR of all enhancing tumour), as agreed at the local neuro-oncology Multi-Disciplinary Team (MDT) meeting.

Intervention: standard care surgery (neuronavigation based on preoperative imaging and intraoperative use of 5-ALA) with the addition of DTI neuronavigation and iUS.

Control: standard care surgery (neuronavigation based on preoperative imaging and intraoperative use of 5-ALA)

Outcome: Deterioration Free Survival, defined as the time to a 10-point deterioration in HRQoL scores from baseline, without subsequent 10-point improvement in scores compared with baseline; or progressive disease; or death in the absence of previous definitive deterioration before the next assessment. HRQoL is measured with the EORTC QLQ-C30 and QLQ-BN20 questionnaires.

Setting: At least 15 UK NHS Trusts undertaking GB surgery

Eligibility
Patients aged 18-70years with a primary GB tumour which is deemed maximally resectable (attempted GTR of all enhancing tumour) by the local neuro-oncology MDT meeting, will be potentially suitable for inclusion in the trial.

Inclusion Criteria

- Age 18-70years
- Neuro-oncology MDT decision that the imaging shows a primary GB tumour which is maximally resectable (attempted GTR of all contrast-enhancing tumour)
- Patient is suitable for concomintant adjuvant radiotherapy and Temozolomide (TMZ) chemotherapy followed by adjuvant TMZ at the time of MDT decision
- Able to receive 5-ALA
- Willing and able to give informed consent
- Able to complete trial questionnaires, this may be with support where English is not their first language (where compatible with the validation of questionnaires) (Stage-2 only)
- Able to provide a proxy who is willing to complete questionnaires as requested (Stage-2 only).

Exclusion Criteria
The participant may not enter the trial if any of the following apply:

For peer review only

- Midline/basal ganglia/cerebellum/brainstem GB
- Multifocal GB
- Recurrent GB
- Suspected secondary GB
- Contraindication to MRI

Proxy Inclusion (Stage-2 only)
Although it is widely recognised in HRQoL research that an individual may rate aspects of their functioning and well-being differently from how another person might, even if that person is close to the individual (e.g. carer, partner etc.), we will ask proxies to also rate HRQoL aspects of patients during the RCT.

The proxy/participant assessment is particularly important in cases where the patients are not able to complete the questionnaires, for example if have disease progression, or if their condition is too poor. These proxy measures can be used as substitute data in case the patient's rating of their HRQoL is lacking. When a participant dies, loses capacity, or withdraws from the trial – this will also automatically cease the proxy's involvement in the trial.

Inclusion Criteria for proxy
- Age 18-75years
- Nominated by an individual who has consented to participate in Stage-2
- Willing and able to give informed consent
- Able to understand written English to enable completion of trial questionnaires

Recruitment
Recruitment into the trial will be undertaken in two phases in conjunction with the separate stages of the trial. There will be a separate Patient Information Sheet and Consent Form for patients entering Stage-1 (IDEAL IIb) and Stage-2 (RCT) (supplementary files8-13). The stages are sequential at participating sites and the stages cannot be recruited to in parallel.

All potentially eligible participants will have the trial mentioned at the same time the options regarding their surgery are discussed. Depending upon the site, the resources available, and most importantly how the participant is dealing with their diagnosis, the recruitment process and approach may vary across and within sites. Potential participants may straight away be provided with the trial participant information sheet and asked to consider the trial, and that a member of the local research team will contact them. It may be the case that individuals are asked if it would be acceptable for their name to be passed to the research team who will make contact at a later timepoint, or potential participants may be given the participant information sheet and asked to call the number on it if they wish to find out more about the trial.

Randomisation
Randomisation of patients will only occur in Stage-2 of the trial. Every centre and each participating surgeon will offer surgery under both arms of the trial. Randomisation will be via the web-based service provided by OCTRU, using the method of minimisation. The minimisation factors will be trial site, age (≤55yrs or >55yrs), expected surgery status (under

general anaesthesia or awake), and eloquence of tumour location (non-eloquent or eloquent).

Participants will be randomised on a 1:1 basis, after having given written consent; however, they will remain blinded as to which arm of the trial they have been allocated. The local clinical team at site will receive an email from the randomisation system detailing the arm of the trial to which a participant has been randomised. Randomisation must occur before the pre-operative imaging takes place so that the assigned trial pre-operative imaging can be undertaken.

Pre and post randomisation withdrawals
Participants may decline to continue to take part in the trial at any time without prejudice. A decision not to participate or withdraw will not affect the standard of care the patient receives. Once withdrawn, the patient will be advised to discuss their further care plan with their surgeon. On withdrawal of the patient, any data collected up until the time of withdrawal will be retained by the research team and included in the final analysis.

Blinding
Stage-1 is not blinded; the participants will be receiving all the technologies during their surgery.

In Stage-2, the participant will be blinded to the allocation (intervention or control arm), and the treating clinician will be aware of the need to perform the surgery with the intraoperative technologies as allocated. In addition to the participant, the radiologist (reviewing the postoperative MRI) will be blinded to the trial arm. Given this, only on the operation CRF will data of the allocation be included.

Trial treatments
All participants will undergo surgery for removal of their GB. The choice of anaesthesia will be left to the discretion of the treating surgeon/anaesthetist/patient as per their normal practice and preference.

The trial will compare two imaging techniques for imaging the tumour. Participants will be randomised to either:

- Standard care surgery (neuronavigation based on preoperative imaging and intraoperative use of 5-ALA) (Control arm)
- Standard care surgery (neuronavigation based on preoperative imaging and intraoperative use of 5-ALA) **AND** of DTI neuronavigation and iUS (Intervention arm)

Objectives and Outcome Measures
Objectives and outcome measures are summarised in table 1.

For peer review only

Stage 1	Objectives	Outcome Measures	Timepoint(s)
**Primary** Outcomes	To demonstrate the feasibility of using DTI and iUS* in addition to standard of care (neuronavigation based on preoperative MRI and intraoperative use of 5-ALA) for neurosurgery (at selected UK NHS hospitals).	1. Operation length<br>2. Successful use of DTI neuronavigation and iUS* to achieve maximal safe tumour resection without major neurological deficit<br>3. Extent of tumour resection assessed on postoperative MRI scan.<br>4. Surgical Complication and Serious Adverse Events	Hospital discharge and 6 months post-op.
**Stage 2**	**Objectives**	**Outcome Measures**	**Timepoint(s)**
**Primary** Outcomes	To assess whether additional intraoperative imaging (DTI and iUS*) to standard of care (neuronavigation based on preoperative MRI scan and intraoperative 5-ALA) improves Deterioration Free Survival (DFS) (Where deterioration relates to global health status only)	Composite of global health status domain of the QLQ-C30 questionnaire, Progression Free Survival (PFS) and Overall Survival (OS) with an event defined as either deterioration, progression or death.	To be recorded at baseline; 6wks post-op., 3mths post-op., and then 3mthly up to 24 months
**Secondary** Outcomes	To assess whether additional intraoperative imaging (DTI and iUS*) to standard of care (Neuronavigation and intraoperative 5-ALA) improves DFS where deterioration relates to physical functioning, social functioning from the QLQ-C30, and motor dysfunction and communication deficit	4 composites using the respective domain of QLQ-C30 (physical functioning and social functioning) and BN20 (motor dysfunction and communication deficit) combined with PFS and OS.	To be recorded at baseline; 6wks post-op., 3mths post-op., and then 3mthly up to 24 months
**Secondary** Outcomes	To assess whether additional intraoperative imaging (DTI and iUS*) to standard of care (Neuronavigation and intraoperative 5-ALA) improves time to deterioration	Defined similar to DFS with the exception that progression is excluded as an event (i.e. only deterioration or death are considered). There will be five time to deterioration outcomes, one for each of the domains utilised in the primary and secondary DFS outcomes, used in turn to define deterioration.	To be recorded at 6wks post-op., 3mths post-op., and then 3mthly up to 24 months
**Secondary** Outcomes	To assess whether additional intraoperative imaging (DTI and IUS*) to standard of care (Neuronavigation and intraoperative 5-ALA) improves Overall Survival (OS)	OS (time from randomisation to death or trial closure)	To be recorded at 24 months
**Secondary** Outcomes	To assess whether additional intraoperative imaging (DTI and iUS*) to standard of care (Neuronavigation and intraoperative 5-ALA) improves Progression Free Survival (PFS)	PFS (time from randomisation to radiological tumour progression on imaging, as agreed in local MDT	MRI at 6 months post-op., and then 3mthly up to 24 months or an MRI performed outside protocol if patient is symptomatic
**Secondary** Outcomes	To assess whether additional intraoperative imaging (DTI and iUS*) to standard of care (Neuronavigation and intraoperative 5-ALA) improves the extent of tumour resection	Extent of resection as volume of residual tumour postoperative contrast enhanced MRI. Extent of resection as % of pre-operative tumour volume on postoperative contrast enhanced MRI.	Post-operative review

For peer review only - http://bmjopen.bmj.com/site/about/guidelines.xhtml

**Secondary** Outcomes	To assess whether additional intraoperative imaging (DTI and iUS*) to standard of care (Neuronavigation and intraoperative 5-ALA) improves the incidence of surgical complications	Number and type of surgical complications	To be recorded at 5 days post-op, or discharge date (whichever is soonest); 6wks post-op., 3mths post-op., and then 3mthly up to 24 months
**Secondary** Outcomes	To assess whether additional intraoperative imaging (DTI and iUS*) to standard of care (Neuronavigation and intraoperative 5-ALA) improves the number of patients eligible for adjuvant treatment following surgery	Number of patients eligible for adjuvant treatment	3mths post-op.
**Secondary** Outcomes	To assess whether additional intraoperative imaging (DTI and iUS*) to standard of care (Neuronavigation and intraoperative 5-ALA) improves functional outcome postoperatively	WHO performance status<br>mini-MoCA (Montreal Version) Barthel Index<br>MRC grading of power in all 4 limbs	To be recorded at baseline, 5 days post-op., or discharge date (whichever is soonest); 6wks post-op., 3mths post-op., and then 3mthly up to 24 months.<br>(MRC grading to be assessed at baseline and 5 days post-op., or discharge date only)
**Secondary** Outcomes	Assess the correlation of proxy to participant classification assessment of quality of life	At a minimum, answers to questions 29 and 30 of the QLQ-C30. Ideally answers will be provided to all of the QLQ-C30 and BN20.	Baseline, 6wks post-op., 3mths post-op., and then 3mthly up to 24 months. Proxy will not complete questionnaires when participant stops completing them.
**Tertiary** Mechanistic Study Objectives – on a sub set of participants –	To assess the sensitivity and specificity of the anatomico-spatial location of DTI fibre tracts compared with intraoperative direct electrical stimulation/behavioural change without stimulation but related to adjacent white fibre tract in patients undergoing awake surgery, or motor evoked potential changes in patients undergoing surgery.	Sensitivity and specificity calculation using pre and post-surgery MRI images	Analysis will be undertaken post-surgery.
**Tertiary** Mechanistic Study Objectives – on a sub set of participants –	To assess the sensitivity and specificity of iUS* to identify the tumour boundary when compared with 5-ALA, navigated biopsies will be taken from tumour boundary tissue planned for resection.	Intra operative iUS* images and post-operative MRI scans and Intraoperative biopsy samples	Analysis will be undertaken post-surgery when biopsy results are available.

*if NiUS available, it is to be used

Table 1. Objectives and outcome measures for the trial.

End of trial

The end of the trial will be defined as the collection/receipt of the last follow-up questionnaire from the last participant and all data cleaning has been completed.

For peer review only

Statistical Methods
Full details of the statistical analysis will be detailed in a separate statistical analysis plan which will be drafted early in the trial and will be finalised after input from the TSC and DSMC. A summary of the planned statistical analysis is included here.

The analysis of the primary outcome will be a time-to-event analysis using a mixed effect Cox proportional hazard regression model. Minimisation factors (age, anticipated patient operative state and tumour location), radiotherapy, and MethylGuanine-DNA MethylTransferase (MGMT) status will be adjusted for as fixed effects. Centre will be included as a random effect.

The assumption of proportional hazard for the Cox model will be examined. If the proportional hazard assumption is not met, parametric survival analysis, such as the accelerated failure time method will be considered. A sensitivity analysis will look at the impact of adjusting for surgeon instead of centre. Secondary analysis will explore the influence of progression as an event by assessing DFS minus disease progression as an event. An unadjusted comparison using a log-rank test will also be carried out. Kaplan-Meier curves will also be generated. Secondary time-to-event outcomes (e.g. OS) will be analysed in a similar manner.

HRQoL amongst survivors will be quantified without a formal statistical comparison between treatment groups.

There are multiple factors that may influence how a patient rates their level of HRQoL, which may be related to factors other than the intervention. However, by using a randomized trial design, it is assumed that patients in both treatment arms are comparable on all aspects, both measured (e.g. age, performance status) and unmeasured (e.g. mood, coping strategy, personality). This means that the impact of the psychological state on the evaluation of HRQoL is treated as similar for the two trial arms. Thus, the trial will be able to measure whether the experimental intervention has an impact on HRQoL when compared to patients receiving standard treatment.

The trial will attempt to collect data as completely as possible. The main analysis will include participants for whom endpoint data are available, with other participants being censored after their last available relevant outcome measure. Sensitivity analyses will examine the effects of alternative assumptions about the missing data. Further details on will be provided in the the Statistical Analysis Plan, and the Data Monitoring Plan.

Sample Size Determination for IDEAL Framework IIB Trial (Stage-1)
There is no formal sample size for the IDEAL trial. Participants will be recruited at each centre, the number of cases required from each centre will vary depending upon caseload numbers and the number of neurosurgeons but is expected to be small for most sites (5), as the participating centres are already familiar with the component techniques.

Sample Size Determination for the RCT (Stage-2)
The sample size is based on a HRQoL aspect included in the primary outcome DFS, i.e. the global health status domain in the EORTC QLQ-C30 questionnaire version 3.0, and achieving

For peer review only

a statistical power of 90% for the primary analysis (see below) with 2-sided significance level of 5%. Assuming a Hazard Ratio (HR) of 0.7, median DFS survival time of 5 months in the control arm, 24 months follow-up on all participants and allowing for 5% loss to follow-up occurring by month 3, this yields an overall target of 357 participants (178/179 per arm; 335 events overall) (Stata “artsurv”; www.stata.com). In a recent trial, the mean survival time of global health status DFS was 6 months in the standard treatment arm (surgical resection with standard radiotherapy and chemotherapy)[32]. Additionally, the observed HR was 0.64, 95% CI (0.56, 0.74) for the DFS measures in this trial suggesting that a HR of 0.7 as assumed above is a plausible magnitude of effect to be observed for this population[33]. It would also be one which would be considered important to clinicians and patients given the definition of a DFS event (death, progression or a patient anchor determined clinically meaningful deterioration of 10 points). For key secondary outcomes (i.e. the other four DFS outcomes, PFS and OS) there is over 80% power for this size of trial, assuming a median OS of 6-9, 7 and 15months respectively in the control arm, a HR of 0.70 for both, and other inputs as per above.

Decision points

Stage-1 (IDEAL IIB trial)

The trial team will evaluate patient CRF and imaging data continuously on a case-by-case basis from each site and provide regular feedback and assessment. Any additional training/guidance is provided as needed. After a site has done an adequate number of cases and has objectively met the primary outcomes and workflow requirements, the completed data set will be revaluated by the trial team including the CI and Lead Investigators. A meeting between the trial team and site is then held to allow feedback from the site and discussion of lessons learned. This meeting is formally documented and if all the criteria are met, the site can then progress to Stage 2 (supplementary files).

Stage-2 (RCT)

Built into the trial is an internal pilot of recruitment to the RCT (Stage-2). There will be a formal stop/go review after 12months of recruitment to the RCT to review the number of randomisations over the pilot period – the stop-go criteria are listed in table 2. If the target of at least 80 randomisations has been met, the trial will continue to recruit for a further 15months. Data from the 80 patients will be included in the final analysis.

	**actual recruitment after 12 months of recruitment**		
**target = 80**	**>80 participants**	**65 - 80 participants**	**<65 participants**
recruitment rate (per centre per month)	0.6	0.45	0.37
stop-go criteria	recruitment feasible<br>proceed with trial	review recruitment strategies report to TSC<br>Continue but modify and monitor closely	recruitment not feasible<br>decision not to proceed

Table 2. Proposed stop-go criteria for the TSC at 12 months.

For peer review only

Data Management

Data will be collected from participants and proxies via questionnaires and case report forms that will be returned to the central trial office in Oxford, via post using a pre-addressed freepost envelope, NHS email as appropriate, or directly into an online secure database (REDCap). In addition, participant images will be stored within the cloud database Quentry (BrainLab AG). As a third-party processor, BrainLab will not receive any data that could identify participants.

All trial-specific documents, except for the signed consent form and follow-up contact details, will refer to the participant with a unique trial participant number/code and not by name. The data will be stored and used in compliance with the relevant, current data protection laws (Data Protection Act 2018; General Data Protection Regulation (GDPR) 2018). The trial data (including data for SAEs) will be entered onto a validated REDCap trial database developed and maintained by OCTRU and which can only be accessed by authorised users via the application. After closure of the trial and data analyses, the data will be made publicly available at the time of publication. The Trial Master File will be archived for five years from the end of the trial.

Patient and public involvement (PPI)

The trial focuses on keeping good HRQoL for people living with a GB for as long as possible. It has been designed with the help of patient support groups at the Brain Tumour Charity and Brainstrust, the Patient Relative Advisory Group at the Oxford University Hospitals NHS Foundation Trust and the Brain Tumour PPI Group at Imperial College Healthcare NHS Trust. Dr Helen Bulbeck (Brainstrust's Director) has been part of the trial proposal and is one of the trial's investigators.

Trial oversight

The day-to-day management of the trial will be the responsibility of the clinical trial manager, based at Nuffield Department of Surgical Sciences and supported by the Oxford Clinical Trials Research Unit (OCTRU) and the Surgical Intervention Trials Unit (SITU) staff all based at the University of Oxford with the Chief Investigator. This will be overseen by the trial management group, who will meet monthly to assess progress.

A trial steering committee (TSC) and a DSMC will be set up. The DSMC will adopt a DAMOCLES based charter which defines its terms of reference and operation in relation to oversight of the trial. They will not be asked to perform any formal interim analyses of effectiveness. They will, however, see copies of data accrued to date and summaries of that data by treatment group. They will also consider emerging evidence from other related trials or research and review related SAEs that have been reported. They may advise the chair of the TSC at any time if, in their view, the trial should be stopped for ethical reasons, including concerns about participant safety. DSMC meetings will be held at least annually during the recruitment phase of the study.

Quality control

The study may be monitored, or audited in accordance with the current approved protocol, relevant regulations and standard operating procedures by the host organisation or sponsor. A monitoring plan will be developed according to OCTRU standard operating

For peer review only - http://bmjopen.bmj.com/site/about/guidelines.xhtml

procedures which involves a risk assessment. The monitoring activities are based on the outcome of the risk assessment and may involve central and site monitoring.

Ethics and Dissemination

The trial was registered in the Integrated Research Application System (Ref: 264482) and approved by a UK research and ethics committee (Ref: 20/LO/0840). Results will be published in a peer reviewed journal. Further dissemination to participants, patient groups and the wider medical community will utilize a range of approaches to maximize impact.

Author Contributions

PP, SC, DN were responsible for conceptualisation and design of the trial. RM is a trial clinician and drafted manuscript. JAC provided statistical input into the trial design and conduct. CW, MDJ, KA, SJP, VA are trial clinicians and provided input regarding neurosurgical expertise and trial design. MT, LD, MW provided input on neuro-oncology management. MT, LD (Linda Dirven) provided expert opinion on quality-of-life measures. AL, LD (Luke Dixon) developed section on intraoperative ultrasound techniques and analysis. PM was responsible for guidance on the IDEAL framework. NV, MGS developed section on DTI imaging and analysis. HB organised and provided PPI input. VSB provided input into the trial design. All authors provided critical appraisal of the protocol and manuscript.

Acknowledgments

We would like to thank our participants and other research team members (Amy Taylor, Nadjat Medehgri, Jack Morris, Lucy Eldridge, Ariel Wang and Tianshu Liu) involved in the day to day running of the trial. This trial will be conducted as part of the portfolio of trials in the UK Clinical Research Collaboration registered Clinical Trials Unit – the Oxford Clinical Trials Research Unit (OCTRU) and the Surgical Intervention Trial Unit (SITU) at the University of Oxford. It will follow their Standard Operating Procedures ensuring compliance with the principles of Good Clinical Practice and the Declaration of Helsinki and any applicable regulatory requirements.

Funding

This paper presents independent research funded by the National Institute for Health Research (NIHR) under its Efficacy and Mechanism Evaluation Programme (Grant Reference: NIHR 127930; https://fundingawards.nihr.ac.uk/award/NIHR127930). The views expressed are those of the author(s) and not necessarily those of the NIHR or the Department of Health and Social Care. The University of Oxford is the sponsor of the trial. The funders and the sponsor of the trial had no explicit role in the trial design.

Competing interests

The authors do not have any competing interests to disclose.

For peer review only

References

1 Brodbelt A, Greenberg D, Winters T, *et al.* Glioblastoma in England: 2007-2011. *Eur J Cancer* 2015;**51**:533–42. doi:10.1016/j.ejca.2014.12.014

2 Stupp R, Mason WP, van den Bent MJ, *et al.* Radiotherapy plus concomitant and adjuvant temozolomide for glioblastoma. *N Engl J Med* 2005;**352**:987–96. doi:10.1056/nejmoa043330

3 Overview | Brain tumours (primary) and brain metastases in adults | Guidance | NICE. https://www.nice.org.uk/guidance/ng99 (accessed 1 Sep 2021).

4 Creighton H, Beach B, Bamford S-M. Rethinking Cancer. 2015. www.ilcuk.org.uk (accessed 1 Sep 2021).

5 Burnet NG, Jefferies SJ, Benson RJ, *et al.* Years of life lost (YLL) from cancer is an important measure of population burden - And should be considered when allocating research funds. *Br J Cancer* 2005;**92**:241–5. doi:10.1038/sj.bjc.6602321

6 Cihoric N, Tsikkinis A, Minniti G, *et al.* Current status and perspectives of interventional clinical trials for glioblastoma - analysis of ClinicalTrials.gov. Radiat. Oncol. 2017;**12**:1. doi:10.1186/s13014-016-0740-5

7 Li YM, Suki D, Hess K, *et al.* The influence of maximum safe resection of glioblastoma on survival in 1229 patients: Can we do better than gross-total resection? *J Neurosurg* 2015;**124**:1–12. doi:10.3171/2015.5.JNS142087

8 Stummer W, Reulen HJ, Meinel T, *et al.* Extent of resection and survival in glioblastoma multiforme: identification of and adjustment for bias. *Neurosurgery* 2008;**62**:564–76. doi:00006123-200803000-00003 [pii] 10.1227/01.neu.0000317304.31579.17

9 Brown TJ, Brennan MC, Li M, *et al.* Association of the extent of resection with survival in glioblastoma a systematic review and meta-Analysis. *JAMA Oncol* 2016;**2**:1460–9. doi:10.1001/jamaoncol.2016.1373

10 Bal J, Camp SJ, Nandi D. The use of ultrasound in intracranial tumor surgery. Acta Neurochir. (Wien). 2016;**158**:1179–85. doi:10.1007/s00701-016-2803-7

11 Dixon L, Lim A, Grech-Sollars M, *et al.* Intraoperative Ultrasound in Brain Tumor Surgery: A review and implementation guide. *Neurosurg Rev* 2022.

12 Serra C, Stauffer A, Actor B, *et al.* Intraoperative high frequency ultrasound in intracerebral high-grade tumors. *Ultraschall der Medizin* 2012;**33**:E306–12. doi:10.1055/s-0032-1325369

13 Mahboob S, McPhillips R, Qiu Z, *et al.* Intraoperative Ultrasound-Guided Resection of Gliomas: A Meta-Analysis and Review of the Literature. *World Neurosurg* 2016;**92**:255–63. doi:10.1016/j.wneu.2016.05.007

14 Unsgaard G, Rygh OM, Selbekk T, *et al.* Intra-operative 3D ultrasound in neurosurgery. Acta Neurochir. (Wien). 2006;**148**:235–53. doi:10.1007/s00701-005-0688-y

15 Unsgaard G, Ommedal S, Muller T, *et al.* Neuronavigation by intraoperative three-dimensional ultrasound: Initial experience during brain tumor resection. *Neurosurgery* 2002;**50**:804–12. doi:10.1097/00006123-200204000-00022

16 Unsgaard G, Gronningsaeter A, Ommedal S, *et al.* Brain operations guided by real-time two-dimensional ultrasound: New possibilities as a result of improved image quality. *Neurosurgery* 2002;**51**:402–12. doi:10.1097/00006123-200208000-00019

17 Sæther CA, Torsteinsen M, Torp SH, *et al.* Did survival improve after the

For peer review only

implementation of intraoperative neuronavigation and 3d ultrasound in glioblastoma surgery? A retrospective analysis of 192 primary operations. *J Neurol Surgery, Part A Cent Eur Neurosurg* 2012;**73**:73–8. doi:10.1055/s-0031-1297247
18 Camp SJ, Apostolopoulos V, Raptopoulos V, *et al.* Objective image analysis of real-time three-dimensional intraoperative ultrasound for intrinsic brain tumour surgery. *J Ther Ultrasound* 2017;**5**:2. doi:10.1186/s40349-017-0084-0
19 Woydt M, Krone A, Becker G, *et al.* Correlation of Intra-Operative Ultrasound with Histopathologic Findings after Tumour Resection in Supratentorial Gliomas: A Method to Improve Gross Total Tumour Resection. *Acta Neurochir (Wien)* 1996;**138**:1391–8. doi:10.1007/BF01411117
20 Moiyadi A, Shetty P. Navigable Intraoperative Ultrasound and Fluorescence-Guided Resections Are Complementary in Resection Control of Malignant Gliomas: One Size Does Not Fit All. *J Neurol Surgery, Part A Cent Eur Neurosurg* 2014;**75**:434–41. doi:10.1055/s-0034-1372436
21 Voets NL, Bartsch A, Plaha P. Brain white matter fibre tracts: A review of functional neuro-oncological relevance. J. Neurol. Neurosurg. Psychiatry. 2017;**88**:1017–25. doi:10.1136/jnnp-2017-316170
22 Bello L, Riva M, Fava E, *et al.* Tailoring neurophysiological strategies with clinical context enhances resection and safety and expands indications in gliomas involving motor pathways. *Neuro Oncol* 2014;**16**:1110–28. doi:10.1093/neuonc/not327
23 Vassal F, Schneider F, Sontheimer A, *et al.* Intraoperative visualisation of language fascicles by diffusion tensor imaging-based tractography in glioma surgery. *Acta Neurochir (Wien)* 2013;**155**:437–48. doi:10.1007/s00701-012-1580-1
24 Herbet G, Maheu M, Costi E, *et al.* Mapping neuroplastic potential in brain-damaged patients. *Brain* 2016;**139**:829–44. doi:10.1093/brain/awv394
25 Winston GP, Daga P, White MJ, *et al.* Preventing visual field deficits from neurosurgery. *Neurology* 2014;**83**:604–11. doi:10.1212/WNL.0000000000000685
26 Caverzasi E, Hervey-Jumper SL, Jordan KM, *et al.* Identifying preoperative language tracts and predicting postoperative functional recovery using HARDI q-ball fiber tractography in patients with gliomas. *J Neurosurg* 2016;**125**:33–45. doi:10.3171/2015.6.JNS142203
27 Wu JS, Zhou LF, Tang WJ, *et al.* Clinical evaluation and follow-up outcome of diffusion tensor imaging-based functional neuronavigation: A prospective, controlled study in patients with gliomas involving pyramidal tracts. *Neurosurgery* 2007;**61**:935–48. doi:10.1227/01.neu.0000303189.80049.ab
28 Ma R, Livermore LJ, Plaha P. Fast Track Recovery Program After Endoscopic and Awake Intraparenchymal Brain Tumor Surgery. *World Neurosurg* 2016;**93**:246–52. doi:10.1016/j.wneu.2016.06.015
29 Jenkinson MD, Barone DG, Bryant A, *et al.* Intraoperative imaging technology to maximise extent of resection for glioma. Cochrane Database Syst. Rev. 2018;**2018**. doi:10.1002/14651858.CD012788.pub2
30 McCulloch P, Cook JA, Altman DG, *et al.* IDEAL framework for surgical innovation 1: the idea and development stages. *BMJ* 2013;**346**:f3012. doi:10.1136/bmj.f3012
31 McCulloch P, Altman DG, Campbell WB, *et al.* No surgical innovation without evaluation: the IDEAL recommendations. Lancet. 2009;**374**:1105–12. doi:10.1016/S0140-6736(09)61116-8
32 Taphoorn MJB, Henriksson R, Bottomley A, *et al.* Health-related quality of life in a

For peer review only

randomized phase III study of bevacizumab, temozolomide, and radiotherapy in newly diagnosed glioblastoma. *J Clin Oncol* 2015;**33**:2166–75. doi:10.1200/JCO.2014.60.3217

33 Cook JA, Julious SA, Sones W, *et al.* DELTA 2 guidance on choosing the target difference and undertaking and reporting the sample size calculation for a randomised controlled trial. *BMJ* 2018;**363**. doi:10.1136/bmj.k3750

For peer review only

Figure 1. Trial Flowchart

For peer review only

Stage 1

Non-randomised multicentre learning curve and evaluation phase (IDEAL Phase IIB study)

Stage 2

Prospective, Phase III, multicentre randomised controlled trial with internal pilot

Randomised (n=357)

Adults (18-70 yrs) scheduled to undergo maximal surgery for a primary high-grade brain tumour (Glioblastoma)

Control Group (n=178)
Surgery using standard neuronavigation and 5-ALA

Follow up at 72 hours
MRI (standard care)

1-7 days post surgery
QoL and Physical ability

Follow up at 6 months
MRI (standard of care), HR-QoL, Complications and adverse events, Functional performance status.

Follow up at 9, 12,15, 18,21,24 months
MRI (standard of care), HR-QoL, Complications and adverse events, Functional performance status.
Mortality status

Treatment Group (n=179)
Surgery using Standard neuronavigation and 5-ALA with the addition of Intraop DTI and iUS

Follow up at 72 hours
MRI (standard care)

1-7 days post surgery
QoL and Physical ability

Follow up at 6 months
MRI (standard of care), HR-QoL, Complications and adverse events, Functional performance status.

Follow up at 9, 12,15, 18,21,24 months
MRI (standard of care), HR-QoL, Complications and adverse events, Functional performance status.
Mortality status

Abbreviations: 5-ALA – Aminolevulinic acid; DTI – Diffusion tensor imaging; iUS – Intraoperative ultrasound; MRI – magnetic resonance imaging; QoL – quality of life.

Two Mechanistic Sub Studies:
1) Sensitivity and specificity of the anatomico-spatial location of DTI fibre tracts compared with intraoperative findings in patients undergoing awake surgery.
2) Sensitivity and specificity of iUS to identify the tumour boundary when compared with 5-ALA, navigated biopsies will be taken from tissue planned for resection.

OUTCOMES: (Tracked for 24 months)
Primary- Deterioration Free Survival ⁎ ‡
Secondary:

- Overall survival ⁎
- Time to Deterioration ⁎ ‡
- Progression free survival †
- Extent of tumour resection on postoperative contrast enhanced MRI †
- Surgical complications and Adverse Events ⁎*
- Number of patients eligible for Adjuvant therapy following surgery (Radiotherapy and Chemotherapy) ⁎*
- Functional outcome post surgery (WHO) performance status, Cognitive ability (MOCA), Physical ability (Barthel Index and MRC power grading in all 4 limbs) ⁎*
- Mechanistic study outcomes

FUTURE-GB STAGE 1 SITE DATA COMPLETION ASSESSMENT

**Reviewers:**	*Intra operative workflow & DTI:*	Prof Natalie Voets, Puneet Plaha, Miss Joy Roach, Amy Taylor
	Intra operative workflow & US:	Dipankar Nandi, Sophie Camp, Luke Dixon, Amy Taylor
	REDCap data entry & workflow:	Amy Taylor, Jack Morris, Puneet Plaha

**Site name:**		**Total Patients recruited:**		**Total Patients screened:**	

**Study ID**					
**Date of review**					
**Date of surgery**					
**Awake or GA surgery**					
**Pre -op tumour planning on MRI scans**					
**Pre-op tumour volume (cm^3)**					
**Post-op tumour volume (cm^3)**					
**Comments:**					
**MRI DTI and USG Acquisition**					
DTI – Site engaged with trials unit regarding DTI acquisition protocols ?	Yes/No	Yes/No	Yes/No	Yes/No	Yes/No
DTI scan acquired for surgery?	Yes/No	Yes/No	Yes/No	Yes/No	Yes/No
DTI Tracts reconstructed ?	Yes/No	Yes/No	Yes/No	Yes/No	Yes/No
USG used during surgery?	Yes/No	Yes/No	Yes/No	Yes/No	Yes/No
**Redcap data entry complete?**	Yes/No	Yes/No	Yes/No	Yes/No	Yes/No
Any difficulty entering data on REDCap ?	Yes/No	Yes/No	Yes/No	Yes/No	Yes/No
**Intraoperative workflow & Quentry imaging data transfer complete**	Yes/No	Yes/No	Yes/No	Yes/No	Yes/No
Data anonymised	Yes/No	Yes/No	Yes/No	Yes/No	Yes/No
Pre-op MRI scan performed	Yes/No	Yes/No	Yes/No	Yes/No	Yes/No
Pre -op MRI scan transferred					
Any Intra-op Screenshots acquired for awake surgery	Yes/No/ N/A	Yes/No/ N/A	Yes/No/ N/A	Yes/No/ N/A	Yes/No/ N/A
Intra-op Screenshots acquired for GA neurophysiology	Yes/No/ N/A	Yes/No/ N/A	Yes/No/ N/A	Yes/No/ N/A	Yes/No/ N/A
USG pre-resection – pictures/videos	Yes/No	Yes/No	Yes/No	Yes/No	Yes/No
USG post-resection- pictures/videos	Yes/No	Yes/No	Yes/No	Yes/No	Yes/No
Post-op MRI scan performed	Yes/No	Yes/No	Yes/No	Yes/No	Yes/No

FUTURE-GB_S1_Template_SiteDataCompletion_V3.0_18Oct2021.docx

FUTURE-GB STAGE 1 SITE DATA COMPLETION ASSESSMENT

**Post op MRI scan transferred**	Yes/No	Yes/No	Yes/No	Yes/No	Yes/No
**Difficulties in transferring Data to Quentry**	Yes/No	Yes/No	Yes/No	Yes/No	Yes/No
**Site PI - DTI comments**					
**Reviewer - DTI comments**					
**Reviewer - US comments**					
**Site PI - US comments**					
**Primary outcomes for Stage 1 complete**	Yes/No	Yes/No	Yes/No	Yes/No	Yes/No
Operation length					
Successful use of DTI neuronavigation and iUS to achieve complete tumour resection without major neurological deficit *(success defined here as appropriate and competent use of the imaging technologies to achieve the projected surgical outcome for each patient)*					
Extent of tumour resection assessed on postoperative MRI scan					
Surgical Complication and Serious Adverse Events (if applicable)	Yes/No	Yes/No	Yes/No	Yes/No	Yes/No
**Reviewer Comments:**					
**Analysis of imaging data to answer tertiary end point of the RCT study**					
Utility of imaging data to answer the DTI tertiary endpoint					
Utility of imaging data to answer the US tertiary endpoint					
**Reviewer Comments**					

**Overall reviewer comments**	

SITE FEEDBACK LOG

Date of meeting/discussion with site during Stage 1	Type of meeting	Feedback comments

FUTURE-GB_S1_Template_SiteDataCompletion_V3.0_18Oct2021.docx

FUTURE-GB STAGE 2 PROGRESSION AGREEMENT

The Co-Chief investigators of the FUTURE-GB trial agree that the trial site:

<site name and NHS Trust>

has met the following provisions in Stage 1 of the FUTURE-GB trial and recruited <number> participants

Trial team review was conducted on DDMonYYYY and it was agreed on this date (DDMonYYYY) that this site can proceed to Stage 2.

Co-Chief investigators report:

This should include:

1. *Objective endpoints of Stage 1*
2. *Quality of DT and US imaging data*
3. *Any difficulties regarding data-workflow from site*
4. *Any suggestions for improvement*

Note: Completion of this agreement by all signatories will result in the Stage 1 Registration System and screening system being closed by the Trial Manager on or after this date, and requesting that the site is opened to recruitment in the Stage 2 Screening, Randomisation and Database System. The Stage 1 Database system will not be closed to the site until all outstanding data has been entered and cleaned/queried as required by the Trial Statistician.

FUTURE-GB STAGE 2 PROGRESSION AGREEMENT

Name	Signature	Date
Professor Puneet Plaha		
Professor Dipankar Nandi		
Miss Sophie Camp		

PARTICIPANT ID: FG-XX-XXXX

PARTICIPANT QUESTIONNAIRE

Thank you for agreeing to participate in the FUTURE-GB study.

This is the baseline questionnaire.

We would be grateful if you could complete this questionnaire on how you are feeling and if you are having any issues due to your glioblastoma.

We will ask you to complete this same questionnaire again before you leave hospital, then at 6 weeks after your entered the study, 3 months after you entered the study and every 3 months thereafter up until 24 months.

If you have any questions about the study or this questionnaire, please do not hesitate to contact a member of the study team on future-gb@nds.ox.ac.uk or call 01865 xxxxxx (Monday to Friday, 9-4pm), there is an answering machine for messages outside of these times, or contact your local clinical team)

For peer review only - http://bmjopen.bmj.com/site/about/guidelines.xhtml

PARTICIPANT ID: FG-XX-XXXX

We are interested in some things about you and your health.

Please answer all of the questions yourself by circling the number that best applies to you. There are no "right" or "wrong" answers. The information that you provide will remain strictly confidential.

We appreciated that you may be very busy and this may be a distressing time, therefore if you are unable to complete this questionnaire either due to time or other reasons – if at all possible however we would be very grateful if could complete the date below and questions 29 and 30 on page 4.

What is today's date: DD/MM/YYYY

For peer review only - http://bmjopen.bmj.com/site/about/guidelines.xhtml

PARTICIPANT ID: FG-XX-XXXX

		Not at All	A Little	Quite a Bit	Very Much
1.	Does you have any trouble doing strenuous activities, like carrying a heavy shopping bag or a suitcase?	1	2	3	4
2.	Do you have any trouble taking a long walk?	1	2	3	4
3.	Do you have any trouble taking a short walk outside of the house?	1	2	3	4
4.	Do you need to stay in bed or a chair during the day?	1	2	3	4
5.	Do you need help with eating, dressing, washing yourself or using the toilet?	1	2	3	4

During the past week:

		Not at All	A Little	Quite a Bit	Very Much
6.	Were you limited in doing either your work or other daily activities?	1	2	3	4
7.	Were you limited in pursuing their hobbies or other leisure time activities?	1	2	3	4
8.	Were you short of breath?	1	2	3	4
9.	Have you had pain?	1	2	3	4
10.	Did you need to rest?	1	2	3	4
11.	Have you had trouble sleeping?	1	2	3	4
12.	Have you felt weak?	1	2	3	4
13.	Have you lacked their appetite?	1	2	3	4
14.	Have you felt nauseated?	1	2	3	4
15.	Have you vomited?	1	2	3	4
16.	Have you been constipated?	1	2	3	4
17.	Have you had diarrhoea?	1	2	3	4
18.	Were you tired?	1	2	3	4
19.	Did pain interfere with your daily activities?	1	2	3	4
20.	Have you had difficulty concentrating on things, like reading a newspaper or watching television?	1	2	3	4
21.	Did you feel tense?	1	2	3	4
22.	Did you worry?	1	2	3	4
23.	Did you feel irritable?	1	2	3	4
24.	Did you feel depressed?	1	2	3	4
25.	Have you had difficulty remembering things?	1	2	3	4
26.	Has your physical condition or medical treatment interfered with your family life?	1	2	3	4
27.	Has your physical condition or medical treatment interfered with your social activities?	1	2	3	4

For peer review only - http://bmjopen.bmj.com/site/about/guidelines.xhtml

PARTICIPANT ID: FG-XX-XXXX

		Not at All	A Little	Quite a Bit	Very Much
28.	Has your physical condition or medical treatment caused you financial difficulties?	1	2	3	4

For the following questions please circle the number between 1 and 7 that best applies to you

29. How would you rate your overall <u>health</u> during the past week?

1 2 3 4 5 6 7

Very poor Excellent

30. How would you rate your overall <u>quality of life</u> during the past week?

1 2 3 4 5 6 7

Very poor Excellent

Patients sometimes report that they have the following symptoms. Please indicate the extent to which you have experienced the below symptoms or problems during the past week.

During the past week:

		**Not at All**	**A Little**	**Quite a Bit**	**Very Much**
31.	Did you feel uncertain about the future?	1	2	3	4
32.	Did you feel you had setbacks in your condition?	1	2	3	4
33.	Were you concerned about disruption of family life?	1	2	3	4
34.	Did you have headaches?	1	2	3	4
35.	Did your outlook on the future worsen?	1	2	3	4
36.	Did you have double vision?	1	2	3	4

For peer review only - http://bmjopen.bmj.com/site/about/guidelines.xhtml

PARTICIPANT ID: FG-XX-XXXX

		Not at All	A Little	Quite a Bit	Very Much
37.	Was your vision blurred?	1	2	3	4
38.	Did you have difficulty reading because of your vision?	1	2	3	4
39.	Did you have seizures?	1	2	3	4
40.	Did you have weakness on one side of your body?	1	2	3	4
41.	Did you have trouble finding the right words to express yourself?	1	2	3	4
42.	Did you have difficulty speaking?	1	2	3	4
43.	Did you trouble communicating your thoughts?	1	2	3	4
44.	Did you feel drowsy during the daytime?	1	2	3	4
45.	Did you have trouble with your coordination?	1	2	3	4
46.	Did hair loss bother you?				
47.	Did itching of their skin bother you?	1	2	3	4
48.	Did you have weakness of both legs?	1	2	3	4
49.	Did you feel unsteady on your feet?	1	2	3	4
50.	Did you have trouble controlling your bladder?	1	2	3	4

For peer review only

For peer review only - http://bmjopen.bmj.com/site/about/guidelines.xhtml

PARTICIPANT ID: FG-XX-XXXX

SITE ADMINISTERED QUESTIONNAIRE

Please tick which time point this questionnaire relates to:

Baseline	☐	12 months	☐
Hospital Discharge	☐	15 months	☐
6 weeks	☐	18 months	☐
3 months	☐	21 months	☐
6 months	☐	24 months	☐
9 months	☐		

Date of completion: DD/MM/YYYY

Completed by: (PRINT NAME)……………………………………………

This document contains the following validated questionnaires:

- Barthel Index
- MOCA Assessment
- MRC Muscle Power Scale
- WHO Performance Status

For peer review only - http://bmjopen.bmj.com/site/about/guidelines.xhtml

PARTICIPANT ID: FG-XX-XXXX

BARTHEL INDEX

Activity	Score
**FEEDING**<br>0 = unable<br>5 = needs help cutting, spreading butter, etc., or requires modified diet<br>10 = independent	______
**BATHING**<br>0 = dependent<br>5 = independent (or in shower)	______
**GROOMING**<br>0 = needs to help with personal care<br>5 = independent face/hair/teeth/shaving (implements provided)	______
**DRESSING**<br>0 = dependent<br>5 = needs help but can do about half unaided<br>10 = independent (including buttons, zips, laces, etc.)	______
**BOWELS**<br>0 = incontinent (or needs to be given enemas)<br>5 = occasional accident<br>10 = continent	______
**BLADDER**<br>0 = incontinent, or catheterized and unable to manage alone<br>5 = occasional accident<br>10 = continent	______
**TOILET USE**<br>0 = dependent<br>5 = needs some help, but can do something alone<br>10 = independent (on and off, dressing, wiping)	______
**TRANSFERS (BED TO CHAIR AND BACK)**<br>0 = unable, no sitting balance<br>5 = major help (one or two people, physical), can sit<br>10 = minor help (verbal or physical)<br>15 = independent	______
**MOBILITY (ON LEVEL SURFACES)**<br>0 = immobile or < 50 yards<br>5 = wheelchair independent, including corners, > 50 yards<br>10 = walks with help of one person (verbal or physical) > 50 yards<br>15 = independent (but may use any aid; for example, stick) > 50 yards	______
**STAIRS**<br>0 = unable<br>5 = needs help (verbal, physical, carrying aid)<br>10 = independent	______
**TOTAL (0–100):**	______

For peer review only - http://bmjopen.bmj.com/site/about/guidelines.xhtml

PARTICIPANT ID: FG-XX-XXXX

MOCA ASSESSMENT

Section	Task	Points
**VISUOSPATIAL / EXECUTIVE**	Trail-making [ ] · Copy cube [ ] · Draw CLOCK (Ten past eleven) (3 points) [ ] Contour [ ] Numbers [ ] Hands	__/5

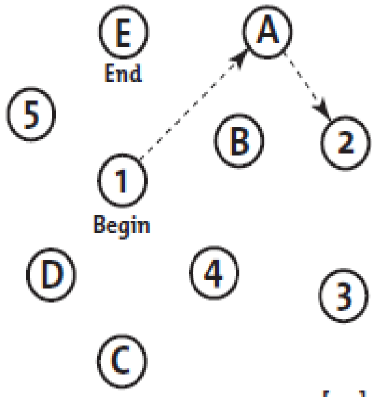

Copy cube

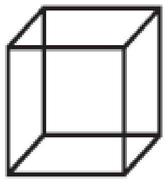

Draw CLOCK (Ten past eleven)
(3 points)

[ ] Contour [ ] Numbers [ ] Hands __/5

NAMING

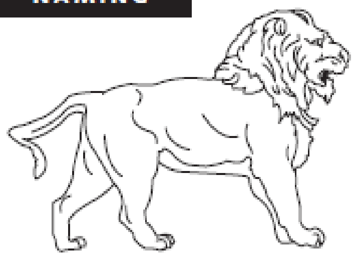
[ ]

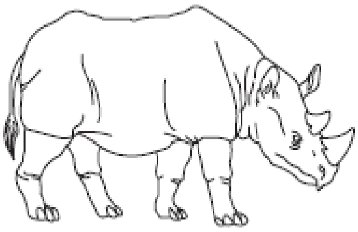
[ ]

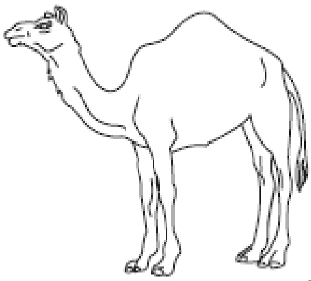
[ ] __/3

MEMORY

Read list of words, subject must repeat them. Do 2 trials. Do a recall after 5 minutes.

	FACE	VELVET	CHURCH	DAISY	RED
1st trial					
2nd trial					

No points

ATTENTION

Read list of digits (1 digit/ sec.). Subject has to repeat them in the forward order [ ] 2 1 8 5 4
Subject has to repeat them in the backward order [ ] 7 4 2 __/2

Read list of letters. The subject must tap with his hand at each letter A. No points if ≥ 2 errors
[ ] F B A C M N A A J K L B A F A K D E A A A J A M O F A A B __/1

Serial 7 subtraction starting at 100 [ ] 93 [ ] 86 [ ] 79 [ ] 72 [ ] 65
4 or 5 correct subtractions: **3 pts**, 2 or 3 correct: **2 pts**, 1 correct: **1 pt**, 0 correct: **0 pt** __/3

LANGUAGE

Repeat : I only know that John is the one to help today. [ ]
The cat always hid under the couch when dogs were in the room. [ ] __/2

Fluency / Name maximum number of words in one minute that begin with the letter F [ ] ____ (N ≥ 11 words) __/1

ABSTRACTION

Similarity between e.g. banana - orange = fruit [ ] train – bicycle [ ] watch - ruler __/2

DELAYED RECALL

Has to recall words	FACE	VELVET	CHURCH	DAISY	RED	Points for UNCUED recall only
WITH NO CUE	[ ]	[ ]	[ ]	[ ]	[ ]	__/5
Optional — Category cue						
Optional — Multiple choice cue						

ORIENTATION

[ ] Date [ ] Month [ ] Year [ ] Day [ ] Place [ ] City __/6

© Z.Nasreddine MD Version November 7, 2004
www.mocatest.org

Normal ≥ 26 / 30 **TOTAL** __/30
Add 1 point if ≤ 12 yr edu

For peer review only - http://bmjopen.bmj.com/site/about/guidelines.xhtml

PARTICIPANT ID: FG-XX-XXXX

MRC MUSCLE POWER SCALE

Score	Description
**0**	No contraction
**1**	Flicker or trace of contraction
**2**	Active movement, with gravity eliminated
**3**	Active movement against gravity
**4**	Active movement against gravity and resistance
**5**	Normal power

Please grade the 4 limbs of the patient

Left arm: ☐

Right arm: ☐

Left leg: ☐

Right leg: ☐

WHO PERFORMANCE STATUS

The WHO performance status classification categorises patients as:

- 0: able to carry out all normal activity without restriction
- 1: restricted in strenuous activity but ambulatory and able to carry out light work
- 2: ambulatory and capable of all self-care but unable to carry out any work activities; up and about more than 50% of waking hours
- 3: symptomatic and in a chair or in bed for greater than 50% of the day but not bedridden
- 4: completely disabled; cannot carry out any self-care; totally confined to bed or chair.

Please circle the WHO Performance status for the patient as at today:

0 1 2 3 4

For peer review only - http://bmjopen.bmj.com/site/about/guidelines.xhtml

PARTICIPANT ID: FG-XX-XXXX

Please tick which time point this questionnaire relates to:

- Hospital Discharge ☐
- 6 weeks ☐
- 3 months ☐
- 6 months ☐
- 9 months ☐
- 12 months ☐
- 15 months ☐
- 18 months ☐
- 21 months ☐
- 24 months ☐

PARTICIPANT QUESTIONNAIRE

Thank you for agreeing to participate in the FUTURE-GB study.

We would be grateful if you could complete this questionnaire on how you are feeling and if you are having any issues due to your glioblastoma.

If you have any questions about the study or this questionnaire, please do not hesitate to contact a member of the study team on future-gb@nds.ox.ac.uk or call 01865 xxxxxx (Monday to Friday, 9-4pm), there is an answering machine for messages outside of these times, or contact your local clinical team)

For peer review only - http://bmjopen.bmj.com/site/about/guidelines.xhtml

For peer review only

PARTICIPANT ID: FG-XX-XXXX

We are interested in some things about you and your health.

Please answer all of the questions yourself by circling the number that best applies to you. There are no “right” or “wrong” answers. The information that you provide will remain strictly confidential.

We appreciated that you may be very busy and this may be a distressing time, therefore if you are unable to complete this questionnaire either due to time or other reasons – if at all possible however we would be very grateful if could complete the date below and questions 29 and 30 on page 4.

What is today’s date: DD/MM/YYYY

For peer review only - http://bmjopen.bmj.com/site/about/guidelines.xhtml

PARTICIPANT ID: FG-XX-XXXX

		Not at All	A Little	Quite a Bit	Very Much
1.	Does you have any trouble doing strenuous activities, like carrying a heavy shopping bag or a suitcase?	1	2	3	4
2.	Do you have any trouble taking a long walk?	1	2	3	4
3.	Do you have any trouble taking a short walk outside of the house?	1	2	3	4
4.	Do you need to stay in bed or a chair during the day?	1	2	3	4
5.	Do you need help with eating, dressing, washing yourself or using the toilet?	1	2	3	4

During the past week:

		Not at All	A Little	Quite a Bit	Very Much
6.	Were you limited in doing either your work or other daily activities?	1	2	3	4
7.	Were you limited in pursuing their hobbies or other leisure time activities?	1	2	3	4
8.	Were you short of breath?	1	2	3	4
9.	Have you had pain?	1	2	3	4
10.	Did you need to rest?	1	2	3	4
11.	Have you had trouble sleeping?	1	2	3	4
12.	Have you felt weak?	1	2	3	4
13.	Have you lacked their appetite?	1	2	3	4
14.	Have you felt nauseated?	1	2	3	4
15.	Have you vomited?	1	2	3	4
16.	Have you been constipated?	1	2	3	4
17.	Have you had diarrhoea?	1	2	3	4
18.	Were you tired?	1	2	3	4
19.	Did pain interfere with your daily activities?	1	2	3	4
20.	Have you had difficulty concentrating on things, like reading a newspaper or watching television?	1	2	3	4
21.	Did you feel tense?	1	2	3	4
22.	Did you worry?	1	2	3	4
23.	Did you feel irritable?	1	2	3	4
24.	Did you feel depressed?	1	2	3	4
25.	Have you had difficulty remembering things?	1	2	3	4
26.	Has your physical condition or medical treatment interfered with your family life?	1	2	3	4
27.	Has your physical condition or medical treatment interfered with your social activities?	1	2	3	4

For peer review only - http://bmjopen.bmj.com/site/about/guidelines.xhtml

PARTICIPANT ID: FG-XX-XXXX

		Not at All	A Little	Quite a Bit	Very Much
28.	Has your physical condition or medical treatment caused you financial difficulties?	1	2	3	4

For the following questions please circle the number between 1 and 7 that best applies to you

29. How would you rate your overall <u>health</u> during the past week?

1 2 3 4 5 6 7

Very poor Excellent

30. How would you rate your overall <u>quality of life</u> during the past week?

1 2 3 4 5 6 7

Very poor Excellent

For peer review only

Patients sometimes report that they have the following symptoms. Please indicate the extent to which you have experienced the below symptoms or problems during the past week.

During the past week:

		**Not at All**	**A Little**	**Quite a Bit**	**Very Much**
31.	Did you feel uncertain about the future?	1	2	3	4
32.	Did you feel you had setbacks in your condition?	1	2	3	4
33.	Were you concerned about disruption of family life?	1	2	3	4
34.	Did you have headaches?	1	2	3	4
35.	Did your outlook on the future worsen?	1	2	3	4
36.	Did you have double vision?	1	2	3	4

For peer review only - http://bmjopen.bmj.com/site/about/guidelines.xhtml

PARTICIPANT ID: FG-XX-XXXX

		Not at All	A Little	Quite a Bit	Very Much
37.	Was your vision blurred?	1	2	3	4
38.	Did you have difficulty reading because of your vision?	1	2	3	4
39.	Did you have seizures?	1	2	3	4
40.	Did you have weakness on one side of your body?	1	2	3	4
41.	Did you have trouble finding the right words to express yourself?	1	2	3	4
42.	Did you have difficulty speaking?	1	2	3	4
43.	Did you trouble communicating your thoughts?	1	2	3	4
44.	Did you feel drowsy during the daytime?	1	2	3	4
45.	Did you have trouble with your coordination?	1	2	3	4
46.	Did hair loss bother you?				
47.	Did itching of their skin bother you?	1	2	3	4
48.	Did you have weakness of both legs?	1	2	3	4
49.	Did you feel unsteady on your feet?	1	2	3	4
50.	Did you have trouble controlling your bladder?	1	2	3	4

For peer review only

For peer review only - http://bmjopen.bmj.com/site/about/guidelines.xhtml

PARTICIPANT ID: FG-XX-XXXX

PARTICIPANT QUESTIONNAIRE

Thank you for agreeing to participate in the FUTURE-GB study.

This is the hospital discharge questionnaire.

We would be grateful if you could complete this questionnaire on how you are feeling and if you are having any issues due to your glioblastoma.

This is the same questionnaire as the one you have completed previously.

If you have any questions about the study or this questionnaire,
please do not hesitate to contact a member of the study team on future-gb@nds.ox.ac.uk or call 01865 xxxxxx (Monday to Friday, 9-4pm), there is an answering machine for messages outside of these times, or contact your local clinical team)

For peer review only - http://bmjopen.bmj.com/site/about/guidelines.xhtml

PARTICIPANT ID: FG-XX-XXXX

We are interested in some things about you and your health.

Please answer all of the questions yourself by circling the number that best applies to you. There are no "right" or "wrong" answers. The information that you provide will remain strictly confidential.

We appreciated that you may be very busy and this may be a distressing time, therefore if you are unable to complete this questionnaire either due to time or other reasons – if at all possible however we would be very grateful if could complete the date below and questions 29 and 30 on page 4.

What is today's date: DD/MM/YYYY

For peer review only - http://bmjopen.bmj.com/site/about/guidelines.xhtml

PARTICIPANT ID: FG-XX-XXXX

		Not at All	A Little	Quite a Bit	Very Much
1.	Does you have any trouble doing strenuous activities, like carrying a heavy shopping bag or a suitcase?	1	2	3	4
2.	Do you have any trouble taking a long walk?	1	2	3	4
3.	Do you have any trouble taking a short walk outside of the house?	1	2	3	4
4.	Do you need to stay in bed or a chair during the day?	1	2	3	4
5.	Do you need help with eating, dressing, washing yourself or using the toilet?	1	2	3	4

During the past week:

		Not at All	A Little	Quite a Bit	Very Much
6.	Were you limited in doing either your work or other daily activities?	1	2	3	4
7.	Were you limited in pursuing their hobbies or other leisure time activities?	1	2	3	4
8.	Were you short of breath?	1	2	3	4
9.	Have you had pain?	1	2	3	4
10.	Did you need to rest?	1	2	3	4
11.	Have you had trouble sleeping?	1	2	3	4
12.	Have you felt weak?	1	2	3	4
13.	Have you lacked their appetite?	1	2	3	4
14.	Have you felt nauseated?	1	2	3	4
15.	Have you vomited?	1	2	3	4
16.	Have you been constipated?	1	2	3	4
17.	Have you had diarrhoea?	1	2	3	4
18.	Were you tired?	1	2	3	4
19.	Did pain interfere with your daily activities?	1	2	3	4
20.	Have you had difficulty concentrating on things, like reading a newspaper or watching television?	1	2	3	4
21.	Did you feel tense?	1	2	3	4
22.	Did you worry?	1	2	3	4
23.	Did you feel irritable?	1	2	3	4
24.	Did you feel depressed?	1	2	3	4
25.	Have you had difficulty remembering things?	1	2	3	4
26.	Has your physical condition or medical treatment interfered with your family life?	1	2	3	4
27.	Has your physical condition or medical treatment interfered with your social activities?	1	2	3	4

For peer review only - http://bmjopen.bmj.com/site/about/guidelines.xhtml
For peer review only

PARTICIPANT ID: FG-XX-XXXX

		Not at All	A Little	Quite a Bit	Very Much
28.	Has your physical condition or medical treatment caused you financial difficulties?	1	2	3	4

For the following questions please circle the number between 1 and 7 that best applies to you

29. How would you rate your overall health during the past week?

1 2 3 4 5 6 7

Very poor Excellent

30. How would you rate your overall quality of life during the past week?

1 2 3 4 5 6 7

Very poor Excellent

For peer review only

Patients sometimes report that they have the following symptoms. Please indicate the extent to which you have experienced the below symptoms or problems during the past week.

During the past week:

		**Not at All**	**A Little**	**Quite a Bit**	**Very Much**
31.	Did you feel uncertain about the future?	1	2	3	4
32.	Did you feel you had setbacks in your condition?	1	2	3	4
33.	Were you concerned about disruption of family life?	1	2	3	4
34.	Did you have headaches?	1	2	3	4
35.	Did your outlook on the future worsen?	1	2	3	4
36.	Did you have double vision?	1	2	3	4

For peer review only - http://bmjopen.bmj.com/site/about/guidelines.xhtml

PARTICIPANT ID: FG-XX-XXXX

		Not at All	A Little	Quite a Bit	Very Much
37.	Was your vision blurred?	1	2	3	4
38.	Did you have difficulty reading because of your vision?	1	2	3	4
39.	Did you have seizures?	1	2	3	4
40.	Did you have weakness on one side of your body?	1	2	3	4
41.	Did you have trouble finding the right words to express yourself?	1	2	3	4
42.	Did you have difficulty speaking?	1	2	3	4
43.	Did you trouble communicating your thoughts?	1	2	3	4
44.	Did you feel drowsy during the daytime?	1	2	3	4
45.	Did you have trouble with your coordination?	1	2	3	4
46.	Did hair loss bother you?				
47.	Did itching of their skin bother you?	1	2	3	4
48.	Did you have weakness of both legs?	1	2	3	4
49.	Did you feel unsteady on your feet?	1	2	3	4
50.	Did you have trouble controlling your bladder?	1	2	3	4

For peer review only

For peer review only - http://bmjopen.bmj.com/site/about/guidelines.xhtml

PROXY ID: FG-XX-XXXX

PROXY QUESTIONNAIRE

Thank you for agreeing to participate in the FUTURE-GB study as a proxy.

This is the 3 months post operation questionnaire.

We would be grateful if you could complete the questionnaire on your opinion of how you friend/spouse/relative is coping in their glioblastoma journey. This is the same questionnaire as all the others you have completed to date.

If you have any questions about the study or this questionnaire, please do not hesitate to contact a member of the study team on future-gb@nds.ox.ac.uk or call 01865 xxxxxx (Monday to Friday, 9-4pm, there is an answering machine for messages outside of these times)

For peer review only - http://bmjopen.bmj.com/site/about/guidelines.xhtml

PROXY ID: FG-XX-XXXX

We are interested in some things about the person and their health of the person you have agreed to act as a proxy for. The below questionnaire will use the term friend – we appreciate though that you may be answering about a relative/friend or spouse. We hope you will allow the use of this term for the purposes of this questionnaire.

Please answer all of the questions yourself by circling the number that best applies to them. There are no “right” or “wrong” answers. The information that you provide will remain strictly confidential.

We appreciated that you may be very busy and this may be a distressing time, therefore if you are unable to complete this questionnaire either due to time or other reasons – if at all possible however we would be very grateful if could complete the date below and questions 29 and 30 on page 4.

What is today’s date: DD/MM/YYYY

For peer review only

This document contains the following validated questionnaires:

- EORTC QLQ-C30
- EORTC QLQ - BN20

For peer review only - http://bmjopen.bmj.com/site/about/guidelines.xhtml

PROXY ID: FG-XX-XXXX

		Not at All	A Little	Quite a Bit	Very Much
1.	Does your friend have any trouble doing strenuous activities, like carrying a heavy shopping bag or a suitcase?	1	2	3	4
2.	Does your friend have any trouble taking a long walk?	1	2	3	4
3.	Does your friend have any trouble taking a short walk outside of the house?	1	2	3	4
4.	Does your friend need to stay in bed or a chair during the day?	1	2	3	4
5.	Does your friend need help with eating, dressing, washing themselves or using the toilet?	1	2	3	4

During the past week:

		Not at All	A Little	Quite a Bit	Very Much
6.	Was your friend limited in doing either work or other daily activities?	1	2	3	4
7.	Was your friend limited in pursuing their hobbies or other leisure time activities?	1	2	3	4
8.	Were they short of breath?	1	2	3	4
9.	Have they had pain?	1	2	3	4
10.	Have they needed to rest?	1	2	3	4
11.	Have they had trouble sleeping?	1	2	3	4
12.	Have they felt weak?	1	2	3	4
13.	Have they lacked their appetite?	1	2	3	4
14.	Have they felt nauseated?	1	2	3	4
15.	Have they vomited?	1	2	3	4
16.	Have they been constipated?	1	2	3	4
17.	Have they had diarrhoea?	1	2	3	4
18.	Have they been tired?	1	2	3	4
19.	Has pain interfered with their daily activities?	1	2	3	4
20.	Have they have difficulty concentrating on things, like reading a newspaper or watching television?	1	2	3	4
21.	Have they felt tense?	1	2	3	4
22.	Have they worried?	1	2	3	4
23.	Have they felt irritable?	1	2	3	4
24.	Have they felt depressed?	1	2	3	4
25.	Have they had difficulty remembering things?	1	2	3	4
26.	Has their physical condition or medical treatment interfered with their family life?	1	2	3	4

For peer review only - http://bmjopen.bmj.com/site/about/guidelines.xhtml

PROXY ID: FG-XX-XXXX

		Not at All	A Little	Quite a Bit	Very Much
27.	Has their physical condition or medical treatment interfered with their social activities?	1	2	3	4
28.	Has their physical condition or medical treatment caused them financial difficulties?	1	2	3	4

For the following questions please circle the number between 1 and 7 that best applies to the person you are a proxy for in your opinion

29. How would you rate their overall health during the past week?

1 2 3 4 5 6 7

Very poor Excellent

30. How would you rate their overall quality of life during the past week?

1 2 3 4 5 6 7

Very poor Excellent

For peer review only

Patients sometimes report that they have the following symptoms. Please indicate the extent to which your relative/proxy/friend have experienced the below symptoms or problems during the past week.

During the past week:

		**Not at All**	**A Little**	**Quite a Bit**	**Very Much**
31.	Did they feel uncertain about the future?	1	2	3	4
32.	Did they feel that have had setbacks in their condition?	1	2	3	4
33.	Were they concerned about disruption of family life?	1	2	3	4
34.	Did they have headaches?	1	2	3	4

For peer review only - http://bmjopen.bmj.com/site/about/guidelines.xhtml

PROXY ID: FG-XX-XXXX

		Not at All	A Little	Quite a Bit	Very Much
35.	Did their outlook on the future worsen?	1	2	3	4
36.	Did they have double vision?	1	2	3	4
37.	Has their vision blurred?	1	2	3	4
38.	Did they have difficulty reading because of their vision?	1	2	3	4
39.	Did they have seizures?	1	2	3	4
40.	Did they have weakness on one side of their body?	1	2	3	4
41.	Did they have trouble finding the right words to express themselves?	1	2	3	4
42.	Did they have difficulty speaking?	1	2	3	4
43.	Did they have trouble communicating their thoughts?	1	2	3	4
44.	Did they feel drowsy during the daytime?	1	2	3	4
45.	Did they trouble with their coordination?	1	2	3	4
46.	Did hair loss bother them?				
47.	Did itching of their skin bother them?	1	2	3	4
48.	Did they have weakness of both of their legs?	1	2	3	4
49.	Did they feel unsteady on their feet?	1	2	3	4
50.	Did they have trouble controlling their bladder?	1	2	3	4

For peer review only

For peer review only - http://bmjopen.bmj.com/site/about/guidelines.xhtml

Oxford University Hospitals
NHS Foundation Trust

Functional and Ultrasound guided Resection of Glioblastoma – the FUTURE-GB study –

Stage 1- IDEAL 2b Phase

Patient Information Leaflet

Invitation to join the FUTURE-GB study

We would like to invite you to take part in a research study (also called a clinical trial).

Before you decide whether to take part or not, it is important that you understand why we are doing this study and what it will involve.

Please take time to read the following information and talk to others about the study. If anything is unclear, or if you would like more information, please ask a member of the study team who will be happy to answer any questions.

What is the purpose of this study?

There are many different types of brain tumours. These can vary in how quickly they grow and what symptoms they cause. For a brain tumour that grows quickly it is important to remove as much tumour tissue as possible. To do this without causing damage to important functional parts of the brain involved in speaking, moving etc., we need accurate imaging during surgery. Several different types of imaging are used during operations, but at present we don't know how effective they are and whether they are actually better than standard care.

We have been funded by the National Institute of Health Research (NIHR) which receives its funding from the UK Government, to find out whether some of these available newer technologies improve quality of life, and life expectancy for people with brain tumours who undergo surgery. We also want to see if using these techniques during an operation means it takes more time for a tumour to come back, and if people have fewer complications from the surgery.

Stage 1

In the first part of the study (Stage 1) we are studying how to use these technologies in combination to achieve the best effect. This means that surgeons will be mentored in the best use of the imaging methods. The surgical teams too will be familiarised with a standard way of using them together. We will carefully monitor how the new methods are used during this phase and discuss them with the surgeons. The aim of this stage of the study is to standardise their use, as we want to have them used in the same way in all participating hospitals by the start of Stage 2. Everyone who agrees to take part in this stage may be helping the doctors treating you familiarise themselves with the new technologies. It is highly likely that your surgeon is already familiar with the use of these technologies.

Note: <u>You are only being asked to take part in Stage 1 and if you agree to take part you will only be part of Stage 1 of this study</u>

FUNDED BY NIHR | National Institute for Health Research · OCTRU · SITU · UNIVERSITY OF OXFORD · Imperial College London

For peer review only - http://bmjopen.bmj.com/site/about/guidelines.xhtml

Oxford University Hospitals
NHS Foundation Trust

For information – once approximately 5 people have agreed to take part in Stage 1 and their operations have taken place at this hospital, Stage 2 of the study will start at this hospital– the information below tells you more about Stage 2.

Stage 2

Stage 2 of the study will compare the operation using the new imaging techniques with the standard operation - so half of those that agree will have the additional, newer, technologies and half will have the standard operation. We hope this will allow us to find a definite answer about which technologies should be used during an operation. The aim of the surgery is to remove safely as much tumour as possible, whilst minimising the risks of damaging brain function and hence affecting quality of life.

The technologies that will be used in this study are all available and in use across NHS practice, and have been shown to be safe. No one knows whether using all of them together will have a definite positive effect on outcome, but it is logical to expect that it should.

The design of this study (FUTURE-GB) has involved patients, their families and healthcare professionals, including brain surgeons, using their knowledge and experience at every stage of project development.

Who is taking part and why have I been invited to take part?

We are hoping to enrol 75 people aged 18 or over, from approximately15 neurosurgical centres in the UK. You will have surgery in your local hospital, which is participating in this trial.

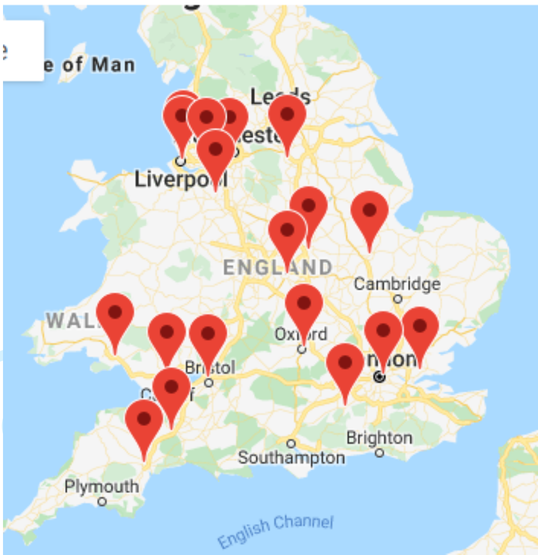

You have been invited to take part because your brain scan suggests you have a brain tumour which comes from the brain itself, rather than from a cancer elsewhere in the body which has spread to the brain. Your scan also suggests the tumour is likely to be aggressive, called a glioblastoma or high-grade tumour.

Do I have to take part in this study?

No, you are under no obligation to take part in the study. Deciding not to will not affect the treatment/care you receive from your team. It is up to you to decide whether to take part or not.

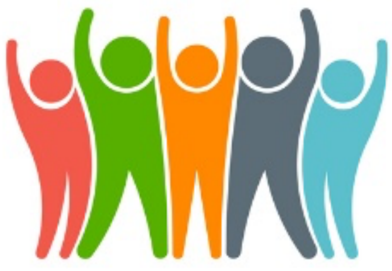

Please keep this leaflet and use it as it may to help you make your decision. If you decide to take part, you will be asked to sign another consent form, as well as that used for your NHS operation.

If you choose not to join the study, you will receive the routine NHS treatment, as agreed by your local treating team of healthcare professionals, in accordance with standard NHS practice as deemed appropriate by your treating team. A note will be made of your age and gender, so that we can find out who decides not to take part. You cannot be identified from this data. A researcher may ask you if you would be happy to give a reason for not wanting to take part in the study. Giving this information is entirely voluntary.

Should you decide to take part, you are still free to withdraw at any time and without giving a reason. This will not affect the standard of care you receive as either an inpatient or an outpatient.

For peer review only - http://bmjopen.bmj.com/site/about/guidelines.xhtml

Oxford University Hospitals
NHS Foundation Trust

What will happen if I take part?

If you are happy to take part in this study, a researcher will ask you some simple questions and check your medical history to confirm that you are eligible.

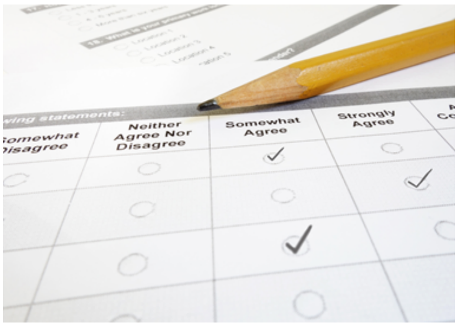

Initial assessment: If you are eligible and wish to participate, you will be asked to sign and date a consent form for the study. Researchers will then take from your medical notes, information about your brain tumour history including the symptoms you have had and where your tumour is.

The researchers would then record details about your operation and details of when you leave hospital after your operation. No questions will need to be asked of you as this will either be standard things already being recorded in your notes or when the technologies are being used, the settings being used in your operation.

Most significantly for those that agree to take part in FUTURE-GB the time taken for your preoperative scans (which you will have by being in the study or not) will perhaps take another 5 minutes. Your operation may also be slightly longer due to the technologies being used – this might perhaps extend it by 15 minutes. (Your doctors will talk to you about what happens in your preoperative scans – but we want you to know that some people find them quite claustrophobic – but the scans are needed for your surgery regardless of taking part in FUTURE-GB). Also, those who have metal in their body may potentially not be able to have type of scan called an MRI scan – talk to the doctors if you think you have metal in your body.

Please note: The design of this study has involved patients, their families and healthcare professionals, including brain surgeons, using their knowledge and experience at every stage of its development.

What technologies will be used in my operation?

Surgery will involve standard care and newer technologies

Standard care:

You will have an MRI scan before your operation. This can be used during the surgery to help your surgeon identify where your brain tumour is located, and what brain structures are close by. MRI stands for Magnetic Resonance Imaging. It is a type of scan that uses strong magnetic fields to produce detailed images. It is used for brain tumour surgery to obtain detailed images of your brain and specifically your brain tumour. There is no risk of radiation exposure. All MRI scans that you will receive are received by all those with a brain tumour, anything seen on these scans will be acted upon as per local NHS Trust and national guidelines.

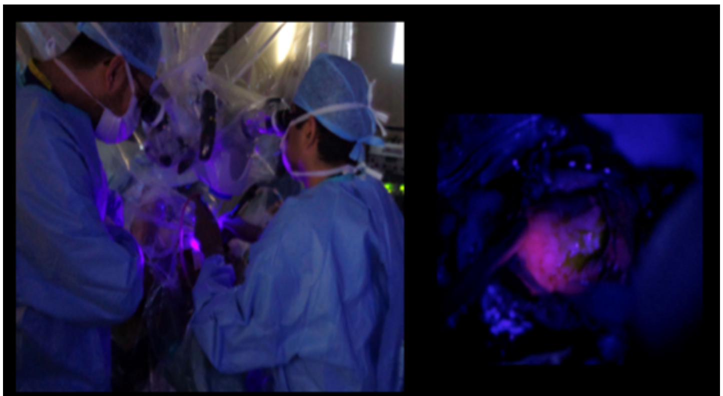
Surgeons using microscope equipped with blue light
Appearance of tumour with 5ALA under blue light

This is combined with use of a chemical called 5-aminolevulinic acid (5-ALA), which is a drink taken a few hours before surgery. This allows the tumour cells to light up pink, when a blue light is shone on them during surgery. This has been shown to help surgeons remove more of the brain tumour, as they are able to see better the edges of the tumour and differentiate it from the surrounding normal brain. This makes sure as much of the tumour is removed as is possible, but it can never usually be totally removed.

FUNDED BY NIHR | National Institute for Health Research

For peer review only - http://bmjopen.bmj.com/site/about/guidelines.xhtml

Oxford University Hospitals
NHS Foundation Trust

Newer technologies:

1) Diffusion Tensor Imaging (DTI) is an MRI technology which allows the surgeons to have a scan of all the nerve fibres which are involved in movement, speech etc. around a tumour. This means that when removing the tumour, the surgeon knows more easily where these are based on your DTI scan and can avoid them.

2) Intraoperative Ultrasound is a technology that uses high frequency sound waves to create an image of the brain tumour during the operation. The ultrasound provides “live” pictures of your tumour as surgery progresses and tumour is removed. The surgeon can use this as many times as necessary during your surgery. The ultrasound is the same as that used to provide a picture of a baby inside a pregnant woman.

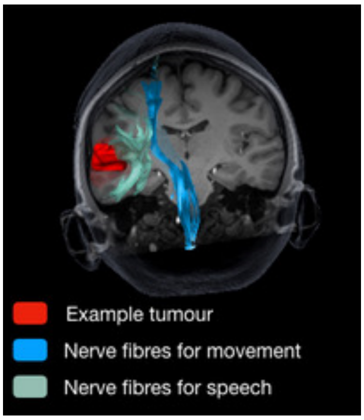

Both these technologies are safe and have been used in brain tumour surgery for a number of years. Your surgeon is familiar with their use and has used them during surgery. However, the benefit of using these 2 new technologies together, in addition to the present “standard of care” surgery has not been scientifically tested, or formally assessed. There are no extra drugs or chemicals used.

Further possible contacts: A researcher from the trial coordinating team may visit while you are having your operation so that we can check how the surgery is being undertaken. We will always check that you are happy for this to happen. If not, the researcher will not come into your operation. At the end of the study, we will report how well the treatments were delivered as it is important we fully understand this process.

Please note, no-one can ever be identified in any public report about the study.

There are 2 companies supporting this study by providing machinery and software to sites if they do not already have the equipment needed for this study. The companies are called BrainLab and Medtronic they are supporting doctors in the UK in using the new technologies. Neither company will be able to influence the results of the study.

What happens after my operation in FUTURE-GB?

Researchers will record information about your operation from your medical notes and directly into the study database, researchers will also collect information from your medical notes and scans when you are discharged home after your operation. The care, any tests, further outpatient appointments and any other surgery or treatments will not be changed by you agreeing to take part in FUTURE-GB. Researchers will check your medical notes for up to 6 months after your operation so find out how you are getting on – but there will be no further contact with you from the study team.

What are the benefits and risks of taking part in the study?

For those that take part in the study, your operation will be conducted by the same surgeon/surgical team whom you have already seen.

We hope the information from this study will answer the question:

Which imaging tools should be used by surgeons when removing a glioblastoma, to offer the highest chance of removing as much of the tumour as possible without causing functional problems, and therefore keeping a good quality of life?

For peer review only - http://bmjopen.bmj.com/site/about/guidelines.xhtml

Oxford University Hospitals
NHS Foundation Trust

We cannot promise the study will help you directly, but the information we get has the potential to be of benefit, potentially allowing more of your tumour to be removed safely.

The risks relating to the brain tumour surgery itself will be discussed with you in detail as part of the standard, routine consent for an operation. We do not think that being part of this study will change any of the risks of the operation but this is one of the things we be will be studying. The technologies will however add some time to the scan before your operation and during your operation.

We are undertaking this study because the extra imaging tools add significant costs to NHS treatment, and therefore we have also been funded to identify whether they provide a real benefit for people with brain tumours.

People sometimes feel uncomfortable answering certain questions about their health, or may be unable to answer. If you, or the person you nominate to answer for you, feel uncomfortable at any point, then you do not have to answer the questions.

Who will know that I am taking part?

The only people who will know that you are taking part in this study are the members of the research team and the healthcare professionals involved in your care. You can tell anyone you would like to that you are taking part.

The only people in the University of Oxford who will have access to information that identifies you will be people who need to contact you to about the study, or review the data. The people who analyse the information will not be able to identify you and will not be able to find out your name or contact details. Also, your de-identified scans will be reviewed by members of the research team and the companies providing the extra imaging technologies. The images will be transferred using cloud servers, however, nothing that could identify you will be included.

Representatives from the Sponsor, relevant regulatory organisations and [insert local Trust] may also need access to monitor or audit the study to ensure that the research is complying with applicable regulations.

Paperwork that is completed by you, the research team, or the treating clinical team, will be sent securely to the study team managing the FUTURE-GB study that are based at the University of Oxford.

Will my details be kept confidential?

Yes. All information collected about you and from you during the course of the research, including from your medical records, will be kept strictly confidential. Everyone who takes part in the study will be assigned a code number and all of the data relating to each person will be held on a computer database and will only be linked to that code number, and not to people's names or addresses. The study team will record into the study database your name, date of birth, NHS or CHI number, Hospital number and your email address. These details will allow the central study team and the local teams to ensure they are collecting data on the correct person. Your email address will only be used to allow you to complete the consent form at home, although this can also be done at the hospital, and to send you a copy of your consent form for your records. Your NHS or CHI number will be used to look up your status 6 months after agreeing to take part.

We will ask you for your permission for individuals from the University of Oxford and Imperial College London, and the regulatory authorities, to have access to your medical notes and data. This is in order that they may conduct checks on the study data that has been collected and to ensure all the study data has been completed correctly. We will also ask you for your permission to allow appropriate individuals from the NHS Trust that you are being approached at to also undertake this review.

At the end of the study, all of the data will be de-identified so that no-one can be identified. This de-identified data will be shared so that more researchers can gain a deeper understanding about patients

For peer review only - http://bmjopen.bmj.com/site/about/guidelines.xhtml

Oxford University Hospitals
NHS Foundation Trust

who have had surgery for glioblastoma. It may be shared with other researchers around the world and with commercial organisations but this information will not identify you, and will not be combined with other information in a way that could identify you. The information will only be used for the purpose of healthcare research, and cannot be used to contact you, nor will it affect your care.

In line with what happens in the NHS, the only situation that confidentiality would need to be broken would be if you told a health professional or research team member of something that could result in harm to yourself or others.

What will happen to my data?

Research is carried out in the public interest. The University of Oxford, as Sponsor, is the data controller. This means that we, as University of Oxford researchers, are responsible for looking after your information as part of FUTURE-GB, and using it properly. We will use the minimum possible personally-identifiable information, and this will be kept for 12 months after the study has finished. Non-identifiable research data and any research documents with personal information, will be stored securely at the University of Oxford for a maximum of 5 years after the end of the study, as part of the research record.

We will be using information from you and your medical records in order to carry out this study. The Oxford University Hospitals NHS Foundation Trust will use your NHS number and contact details to get in touch with you, and to make sure that relevant study information is recorded from your care records. They will keep your identifiable information safely for 12 months after the study has finished. Consent forms and study documents held at [local NHS Trust name] will be archived securely, in accordance with their local procedures.

Data protection regulation provides you with control over your personal data and how it is used. When you agree to your information being used in research, however, some of those rights may be limited, as we need to manage your information in specific ways in order for the research to be reliable and accurate. Further information about your rights with respect to your personal data, is available at https://compliance.web.ox.ac.uk/individual-rights

You can find out more about how we use your information by contacting the FUTURE-GB study team on: futuregb@nds.ox.ac.uk.

What will happen if I don't want to carry on with the study?

You are free to withdraw from taking part in the study at any time without giving a reason. Please remember, it is your decision to take part. If you agree to take part now, but you change your mind during the study, this will not change the standard of care you receive from the NHS. If you were to decide to stop taking part in the study at any time, any data collected on you would be kept. You would not be contacted about the study again or have any further data collected.

What happens at the end of the study?

We will share the results with healthcare researchers and professionals to improve future patient care. Also, we will present them in research reports, at scientific conferences, and publish them in scientific journals, and publish them on the study website futuregb.octru.ox.ac.uk.

We will not include any data that could identify you in the results. If the funders of this research ask us to make the study data available for other researchers, we will first de-identify your information (i.e. we will take your name and other identifying details out) so that you cannot be identified.

For peer review only - http://bmjopen.bmj.com/site/about/guidelines.xhtml

Oxford University Hospitals
NHS Foundation Trust

Who is organising and funding the research?

The University of Oxford is the Sponsor and is organising this study. It is being conducted by a research team led by Prof Puneet Plaha, Consultant Neurosurgeon at the Oxford University Hospitals NHS Foundation Trust and the University of Oxford, and Miss Sophie Camp and Prof. Dipankar Nandi (both Consultant Neurosurgeons at Imperial College Healthcare NHS Trust).

The National Institute of Health Research – Health Technology Assessment programme is funding the study. The funding for the NIHR comes from the UK Government.

Who has approved this study?

A panel of independent researchers and patient representatives, as well as a Research Ethics Committee (REC Reference 20/LO/0840) have reviewed and approved this study.

What if I have concerns?

The University of Oxford, as the study sponsor, has appropriate insurance in place in the unlikely event that you suffer any harm as a direct consequence of your participation in this study.

If you have any concerns or complaints about any aspect of the study, please contact the FUTURE-GB research team using the details below. You can also contact the University of Oxford Research Governance, Ethics & Assurance office on 01865 616480 or by email on ctrg@admin.ox.ac.uk.

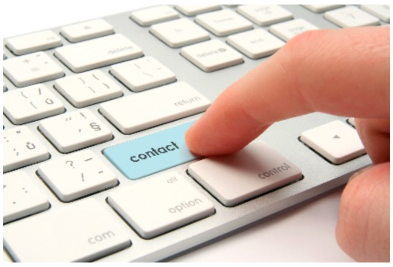

If you would prefer to speak with someone who is not involved in the study, then please contact the Patient Advice and Liaison Service (PALS). PALS is a confidential NHS service that can provide you with support for any complaints or queries you have regarding the care you receive as an NHS patient. However, PALS cannot provide information about this research study.

PALS phone number: [local PALS phone number]

PALS email: [local PALS email]

You can also contact your local clinical team directly:

<local PI/research team name and contact details>

If you have any questions about the study, please contact the FUTURE-GB team on:

Email: futuregb@nds.ox.ac.uk Telephone: 07917 101 649

Postal address: FUTURE-GB study, Botnar Research Centre, Nuffield Orthopaedic Centre, Windmill Road, Oxford, OX3 7LD.

Further information can be found on our study website – futuregb.octru.ox.ac.uk

Thank you for reading this information leaflet and considering taking part.

For peer review only

For peer review only - http://bmjopen.bmj.com/site/about/guidelines.xhtml

LOCAL TRUST LOGO

Functional and Ultrasound guided Resection of Glioblastoma – the FUTURE-GB study – Stage 2 - Randomised Controlled Trial

Patient Information Leaflet

Invitation to join the FUTURE-GB study

We would like to invite you to take part in a research study (also called a clinical trial).

Before you decide whether to take part or not, it is important that you understand why we are doing this study and what it will involve.

Please take time to read the following information and talk to others about the study. If anything is unclear, or if you would like more information, please ask a member of the study team who will be happy to answer any questions.

What is the purpose of this study?

There are many different types of brain tumours. These can vary in how quickly they grow and what symptoms they cause. Studies have shown that when a brain tumour is growing quickly it is better to remove as much tumour as possible. Being able to do this without causing damage to the parts of the brain that are involved in things such as speaking and moving, surgeons need to be able to see clearly parts of the brain during surgery, using accurate imaging. This has led to an increase in the use of imaging (such as ultrasound and MRI scans) during operations. However, we don't know if all the extra imaging tools do definitely make a difference.

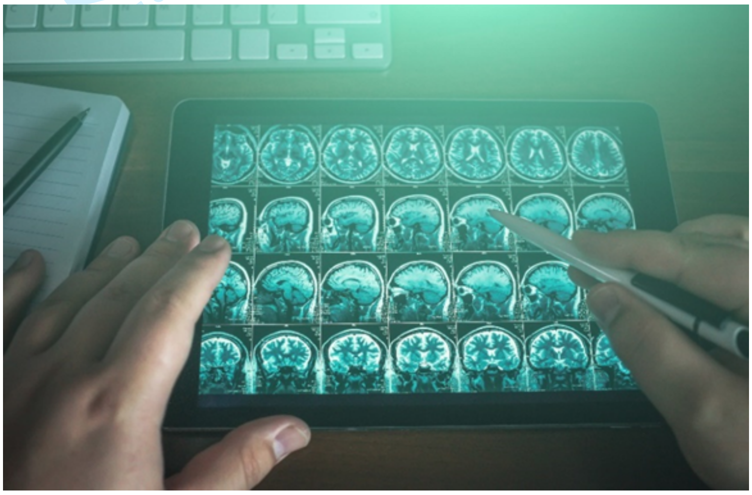

We have been funded by the National Institute of Health Research (NIHR) which receives its funding from the UK Government to find out whether some of these additional imaging tools available make a positive difference to quality of life for people with fast growing brain tumours who have surgery. We will also be looking to see if these imaging tools used during an operation mean people with a brain tumour:

- have a better quality of life?
- if it takes more/less time for their tumour to come back
- if they have more/fewer complications from the surgery.

For peer review only - http://bmjopen.bmj.com/site/about/guidelines.xhtml

LOCAL TRUST LOGO

This study will attempt to give a definite answer, so that surgeons know which imaging tools they should use during an operation to enable as much tumour as possible to be removed safely, whilst minimising the risks of damaging brain function and hence affecting quality of life.

The imaging tools that will be used in this study are available and in use across the NHS, and have been shown to be safe. However, no one knows if using them together will have a definite positive effect on outcome for those with a brain tumour.

Who is taking part and why have I been invited to take part?

Half of the people taking part in the study will have standard NHS imaging (scans), and the other half have standard NHS imaging (scans) and some additional imaging (scans).

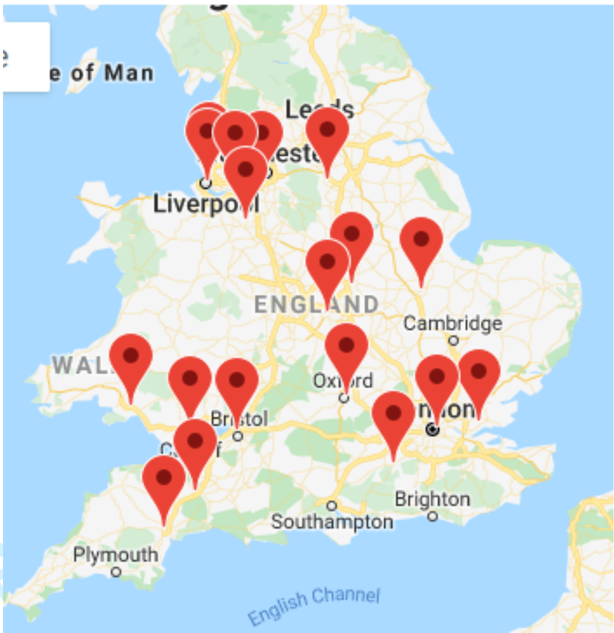

will

We want to enrol 357 people aged 18 - 70 from approximately 15 neurosurgical centres in the UK. You will have surgery in your local neurosurgical unit, which is participating in this study.

You have been invited to take part because your brain scan suggests you have a brain tumour, which comes from the brain itself, rather than from a cancer elsewhere in the body which has spread to the brain. Your scan also suggests the tumour is likely to be aggressive, called a glioblastoma, or high grade tumour.

Do I have to take part in this study?

No, you are under no obligation to take part in the study. Deciding not to will not affect the treatment/care you receive from your team. It is up to you to decide whether to take part or not. Please keep this leaflet and use it as it may to help you make your decision. If you decide to take part, you will be asked to sign another consent form, as well as that used for your NHS operation.

If you choose not to join the study, you will receive the routine NHS treatment, agreed by your local treating team of healthcare professionals, in accordance with standard NHS practice using the imaging your treating team deems appropriate. A note will be made of your age and gender, so that we can find out who decides not to take part. You cannot be identified from this data. A researcher may ask you if you would be happy to give a reason for not wanting to take part in the study. Giving this information is entirely voluntary.

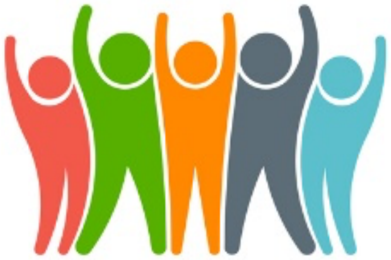

Should you decide to take part, you are still free to withdraw at any time and without giving a reason. This will not affect the standard of care you receive, as either an inpatient or an outpatient.

What will happen if I take part?

If you are happy to take part in this study, a researcher will ask you some simple questions and check your medical history to confirm that you are eligible.

Initial assessment: If you are eligible, you will be asked to sign and date a consent form for the study. We will also ask you to complete some short questionnaires about your health, the activities you are able to

FUNDED BY NIHR National Institute for Health Research | OCTRU | SITU | UNIVERSITY OF OXFORD | Imperial College London

For peer review only - http://bmjopen.bmj.com/site/about/guidelines.xhtml

LOCAL TRUST LOGO

carry out, and about your quality of life. These questionnaires should take you no more than 10-15 minutes to complete. (The questionnaires are electronic – but sometimes you may be given a paper questionnaire to complete, if the electronic system is not available). A researcher will also ask you to complete a brain activity and a recall test, check the strength in your arms and legs and talk to you about how you care for yourself.

Most significantly for those that agree to take part in FUTURE-GB the time taken for your preoperative scans (which you will have by being in the study or not) will perhaps take another 5 minutes. Your operation may also be slightly longer due to the technologies being used – this might perhaps extend it by 15 minutes. (Your doctors will talk to you about what happens in your preoperative scans – but we want you to know that some people find them quite claustrophobic –the scans are needed for your surgery regardless of taking part in FUTURE-GB). Also, those who have metal in their body may potentially not be able to have type of scan called an MRI scan – talk to the doctors if you think you have metal in your body.

Imaging (scans) allocation: You will then be randomly allocated to an imaging group by a computer, which has no information about you as an individual, i.e. allocation is by chance. You will have an equal chance of being allocated to either group, like the toss of a coin. There is a 50% chance that you will be put into the group in which the additional imaging tools will be used during your operation, in addition to the standard techniques, and a 50% chance that you will be in the group where the surgeon uses the present, standard imaging tools.

The random allocation is important because this way, we can test the different imaging tools fairly and nobody can influence into which group you are placed. If you enrol in the study, your healthcare and research teams will not be able to affect which imaging tools will get used in your operation and you will not be able to choose. You will not be aware into which arm of the study you have been allocated, just in case you answer the questionnaires differently based on the imaging used in your operation.

Please note: The design of this study has involved patients, their families and healthcare professionals, including brain surgeons, using their knowledge and experience at every stage of its development.

The FUTURE-GB study aims to find out if the new technologies do, or do not, improve the quality of life of those treated for a brain tumour. We need to know how you are, and your abilities during the study, before and after your operation. We are therefore asking everyone who agrees to take part to nominate a good friend/relative/partner to complete the same questionnaires at the same time as you. They will be asked the same questions as you are asked but they will be asked to answer what their opinion is of your health and abilities.

If you become unable to answer the questions at some point during the study, we would like to know the answers from your good friend/relative/partner to help us identify any changes in your quality of life up to that point in the study. It is helpful to have both assessments to be sure we know about any change in your health status during the study. This is why we would ask both you and your friend/relative/partner to complete the questionnaires throughout the study. The responses that you and your friend/relative/partner give will be used by the trial team to understand the impact of the new technologies on quality of life.
Note: All the data that you and your friend/relative/partner gives will be used by the trial team.

What technologies will be used in my operation?

If you are allocated to the standard techniques arm of the study, your surgeon will use the standard NHS imaging tools.

You will have a Magnetic Resonance Imaging (MRI) scan before your operation. This can be used during the surgery to help your surgeon identify where your brain tumour is located, and what brain structures are close by. This is a type of scan that uses strong magnetic fields to obtain detailed images

FUNDED BY NIHR National Institute for Health Research | OCTRU | SITU | UNIVERSITY OF OXFORD | Imperial College London

For peer review only - http://bmjopen.bmj.com/site/about/guidelines.xhtml

LOCAL TRUST LOGO

of your brain and specifically your brain tumour. There is no risk of radiation exposure. All MRI scans that you will receive are received by all those with a brain tumour, anything seen on these scans will be acted upon as per local NHS Trust and national guidelines.

This is combined with use of a chemical called 5-aminolevulinic acid (5-ALA), which is a drink taken a few hours before surgery. This allows the tumour cells to light up pink, when a blue light is shone on them during surgery. This is known to help surgeons remove more of the brain tumour, as they are able to see better the edges of the tumour compared to the rest of the brain, making sure as much of the tumour is removed as is possible in each individual case.

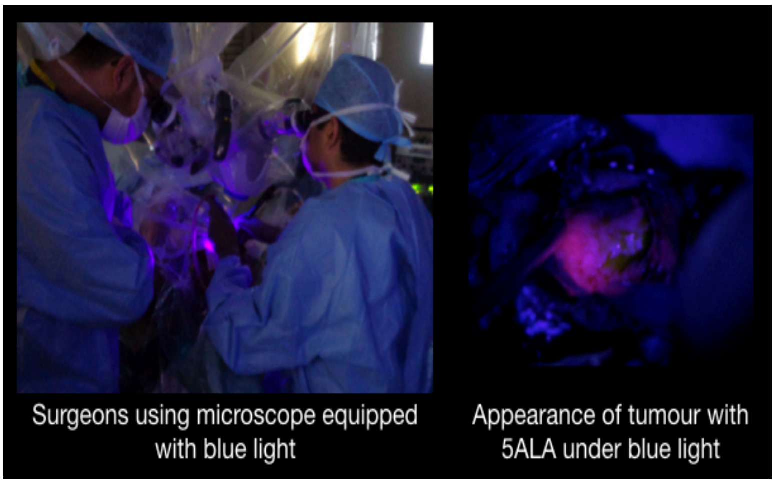

If you are allocated to the group that will use the additional imaging tools, your surgeon will undertake all of what is listed above, together with the additional imaging tools. You will have a slightly longer MRI scan (additional 5 minutes) and have the imaging outlined below also undertaken as part of your operation.

Diffusion Tensor Imaging (DTI) – this is an imaging tool that allows the surgeons to have a scan of all the nerve fibres which are involved in movement, speech etc. around a tumour. This means that when removing the tumour the surgeons potentially know, more accurately, where these are located specifically in your brain from this scan and so can avoid them. This scan is taken using an MRI machine – this is why your MRI scan would be 5 minutes longer.

Intraoperative Ultrasound – this is a technology whereby high frequency sound waves are used to create an image of the brain tumour during the operation. The ultrasound provides “live” pictures of your tumour as surgery progresses and tumour is removed. The surgeon can use this as many times as necessary during your surgery. The ultrasound is the same as that used to provide a picture of a baby inside a pregnant woman.

Both these imaging tools are safe and are used in brain tumour surgery already, but their benefit has not been formally assessed. There are no extra drugs or chemicals used for the additional imaging tools.

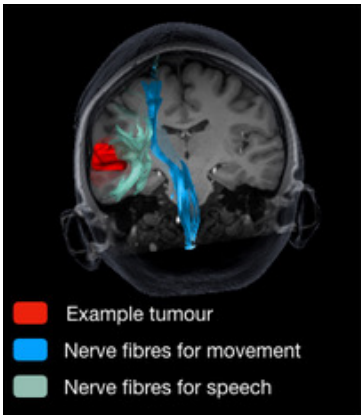

There are 2 companies supporting this study by providing machinery and software to sites if they do not already have the equipment needed for this study. The companies are called BrainLab and Medtronic they are supporting doctors in the UK in using the new technologies. Neither company will be able to influence the results of the study.

Further possible contacts: A researcher from the trial coordinating team may visit while you are having your operation so that we can check how the surgery is being undertaken. We will always check that you are happy for this to happen. If not, the researcher will not come into your operation. At the end of the study, we will report how well the treatments were delivered as it is important we fully understand this process.

For peer review only

For peer review only - http://bmjopen.bmj.com/site/about/guidelines.xhtml

LOCAL TRUST LOGO

What happens after my operation in FUTURE-GB?

If you take part in the study, we will not know whether the additional imaging tools are helpful or not until many months after your operation.

As part of the study you will be asked to complete some further questionnaires. The questionnaires ask about you, your health and activity, and your quality of life. We ask that you complete the questionnaires before you have surgery, when you leave hospital, at 6 and 12 weeks after surgery, and then every 3 months, for a maximum of 2 years. These time points are when you would usually be coming back to the hospital for routine NHS care, and it may be possible to complete them at these appointments. However, they will also be emailed to you for online completion. In addition, either when you are at the hospital for your outpatient appointments, or via telephone at those timepoints, a researcher will also ask you to complete a brain activity and a recall test, and talk to you about how you care for yourself, these will be the same tests and questions as before your operation.

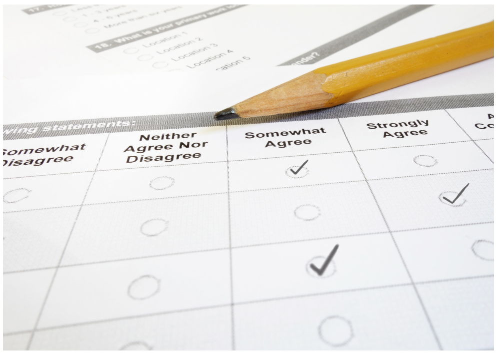

The questionnaires should take no more than 10 minutes to complete on paper/online, or over the telephone. The questionnaires are all completed online, but speak to your researcher if you want to take part but do not have access to the internet.

Please note, the care, any tests, further outpatient appointments and any other surgery or treatments will not be changed by you agreeing to take part in FUTURE-GB. Researchers will check your medical notes for up to 24 months after your operation to find out how you are getting on, and the trial team will use your details to send out the follow-up questionnaires.

Please note if you are deemed to lose capacity at any point during the study, you will not be asked to complete any further questionnaires.

What are the benefits and risks of taking part in the study?

For those that take part in the study, your operation will be conducted by the same surgeon/surgical team whom you have already met.

The information from this study we hope will answer the question:

Which imaging tools should be used by surgeons when removing a glioblastoma, to offer the highest chance of removing as much of the tumour as possible without causing functional problems, and therefore keeping a good quality of life?

We cannot promise the study will help you directly, but the information we get has the potential to be of benefit, potentially allowing more of your tumour to be removed safely.

The risks relating to the brain tumour surgery itself will be discussed with you in detail as part of the standard, routine consent for an operation. We don't think that being part of this study will change any of the risks of the operation but this is one of the things we be will be studying. The technologies will however add some time to the scan you have before your operation and your operation if you are put into the group where the new technologies are used.

We are undertaking this study because we want to find out whether the extra imaging tools provide a real benefit for people with brain tumours. These add significant costs to NHS treatment, and so we need to know if they are worth the extra cost.

For peer review only

For peer review only - http://bmjopen.bmj.com/site/about/guidelines.xhtml

LOCAL TRUST LOGO

People sometimes feel uncomfortable answering certain questions about their health, or may be unable to answer. If you, or the person you nominate to answer for you, feel uncomfortable at any point, then you do not have to answer the questions.

We are not able to pay travel expenses for you to attend your follow-up sessions, however any research questions asked will be as part of your routine out-patient follow-up appointments, via email, or over the telephone.

Who will know that I am taking part?

The only people who will know that you are taking part in this study are the members of the research team and the healthcare professionals involved in your care. You can tell anyone you would like to that you are taking part.

The only people in the University of Oxford who will have access to information that identifies you will be people who need to contact you to about the study, or review the data. The people who analyse the information will not be able to identify you and will not be able to find out your name or contact details. Also, your de-identified scans will be reviewed by members of the research team and the companies providing the extra imaging technologies. The images will be transferred using secure cloud servers, however, nothing that could identify you will be included.

Representatives from the sponsor, relevant regulatory organisations and [Insert local Trust] may also need access to monitor or audit the study to ensure that the research is complying with applicable regulations.

Paperwork that is completed by you, your nominee (friend/relative/partner), the research team, or the treating clinical team, will be sent securely to the study team managing the FUTURE-GB study that are based at the University of Oxford.

We will contact your GP (doctor) to tell them that you have agreed to take part in the FUTURE-GB study. However, we do not share with them anything you answer in your study questionnaires.

Will my details be kept confidential?

Yes. All information collected about you and from you and your nominee (friend/relative/partner) during the course of the research, including from your medical records, will be kept strictly confidential. Everyone who takes part in the study will be assigned a code number and all of the data relating to each person will be held on a computer database and will only be linked to that code number, and not to people's names or addresses. The study team will record into the study database your name, date of birth, NHS or CHI number, Hospital number, address, phone number, GP name and address, and your email address. These details will allow the central study team and the local teams to ensure they are collecting data on the correct person. Your email address will only be used to send you a copy of your consent form for your records and any follow-up questionnaires. This is also the reason your address will be kept on file – in case your questionnaires need to be posted to you to complete. Your phone number will be used by the researchers to call and ask you the questions about brain activity and recall if these cannot be completed at your outpatient appointment. Your NHS or CHI number will be used to check your status 24 months after agreeing to take part. Your GPs details will be used to send a letter to your GP informing them of you taking part in this study.

We will ask you for your permission for individuals from the University of Oxford and Imperial College London, and the regulatory authorities, to have access to your medical notes and data. This is in order that they may conduct checks on the study data that has been collected and to ensure all the study data has been completed correctly. We will also ask you for your permission to allow appropriate individuals from the NHS Trust that you are being approached at to also undertake this review.

FUNDED BY NIHR | National Institute for Health Research — OCTRU — SITU — UNIVERSITY OF OXFORD — Imperial College London

For peer review only - http://bmjopen.bmj.com/site/about/guidelines.xhtml

LOCAL TRUST LOGO

At the end of the study, all of the data will be de-identified so that no-one can be identified. This de-identified data will be shared so that more researchers can gain a deeper understanding about patients who have had surgery for glioblastoma. It may be shared with other researchers around the world and with commercial organisations but this information will not identify you, and will not be combined with other information in a way that could identify you. The information will only be used for the purpose of healthcare research, and cannot be used to contact you, nor will it affect your care.

In line with what happens in the NHS, the only situation that confidentiality would need to be broken would be if you told a health professional or research team member of something that could result in harm to yourself or others.

What will happen to my data?

Research is carried out in the public interest. The University of Oxford, as Sponsor, is the data controller. This means that we, as University of Oxford researchers, are responsible for looking after your information as part of FUTURE-GB, and using it properly. We will use the minimum possible personally-identifiable information, and this will be kept for 12 months after the study has finished. Non-identifiable research data and any research documents with personal information, will be stored securely at the University of Oxford for a maximum of 5 years after the end of the study, as part of the research record.

We will be using information from you and your medical records in order to carry out this study. The [local NHS Trust name] will use your NHS number and contact details to get in touch with you, and to make sure that relevant study information is recorded from your care records. They will keep your identifiable information safely for 12 months after the study has finished. Consent forms and study documents held at [local NHS Trust name] will be archived securely, in accordance with their local procedures.

Data protection regulation provides you with control over your personal data and how it is used. When you agree to your information being used in research, however, some of those rights may be limited, as we need to manage your information in specific ways in order for the research to be reliable and accurate. Further information about your rights with respect to your personal data, is available at https://compliance.web.ox.ac.uk/individual-rights

You can find out more about how we use your information by contacting the FUTURE-GB study team on: futuregb@nds.ox.ac.uk.

What will happen if I don't want to carry on with the study?

You are free to withdraw from taking part in the study at any time without giving a reason. Please remember, it is your decision to take part. If you agree to take part now, but you change your mind during the study, this will not change the standard of care you receive from the NHS. If you were to decide to stop taking part in the study at any time, any data collected on you would be kept. You would not be contacted about the study again or have any further data collected about you from your medical records. If you withdraw or lose capacity please note we will not continue to contact your nominee (proxy).

What happens at the end of the study?

We will share the results with healthcare researchers and professionals to improve future patient care. Also, we will present them in research reports, at scientific conferences, and publish them in scientific journals, and publish them on the study website: futuregb.octru.ox.ac.uk.

For peer review only - http://bmjopen.bmj.com/site/about/guidelines.xhtml

LOCAL TRUST LOGO

We will not include any data that could identify you in the results. If the funders of this research ask us to make the study data available for other researchers, we will first de-identify your information (i.e. we will take your name and other identifying details out) so that you cannot be identified.

Who is organising and funding the research?

The University of Oxford is the Sponsor and is organising this study. It is being conducted by a research team led by Prof. Puneet Plaha, Consultant Neurosurgeon at the Oxford University Hospitals NHS Foundation Trust and the University of Oxford, and Ms Sophie Camp and Prof. Dipankar Nandi (both Consultant Neurosurgeons at Imperial College Healthcare NHS Trust).

The National Institute of Health Research – Health Technology Assessment programme is funding the study. The funding for the NIHR comes from the UK Government.

Who has approved this study?

A panel of independent researchers and patient representatives, as well as a Research Ethics Committee (REC Reference 20/LO/0840) have reviewed and approved this study.

What if I have concerns?

The University of Oxford, as the study sponsor, has appropriate insurance in place in the unlikely event that you suffer any harm as a direct consequence of your participation in this study.

If you have any concerns or complaints about any aspect of the study, please contact the FUTURE-GB research team using the details below. You can also contact the University of Oxford Research Governance, Ethics & Assurance office on 01865 616480 or by email on ctrg@admin.ox.ac.uk.

If you would prefer to speak with someone who is not involved in the study, then please contact the Patient Advice and Liaison Service (PALS). PALS is a confidential NHS service that can provide you with support for any complaints or queries you have regarding the care you receive as an NHS patient. However, PALS cannot provide information about this research study.

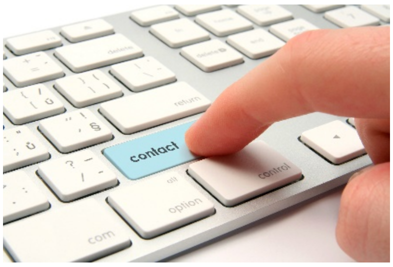

PALS phone number: <Insert local PALS number>

PALS email: <insert local PALS email address>

You can also contact your local clinical team directly:

<local PI/research team name and contact details>

If you have any questions about the study, please contact the FUTURE-GB team on:

Email: futuregb@nds.ox.ac.uk Telephone: 07917 101 649

Postal address: FUTURE-GB study, Botnar Research Centre, Nuffield Orthopaedic Centre, Windmill Road, Oxford, OX3 7LD.

Further information can be found on our study website – futuregb.octru.ox.ac.uk

Thank you for reading this information leaflet and considering taking part.

FUNDED BY NIHR | National Institute for Health Research   OCTRU   SITU   UNIVERSITY OF OXFORD   Imperial College London

For peer review only - http://bmjopen.bmj.com/site/about/guidelines.xhtml

LOCAL TRUST LOGO

Functional and Ultrasound guided Resection of Glioblastoma – the FUTURE-GB study – This is for those that are asked to consider supporting a potential participant in the Future-GB study

Partner/Relative/Friend (Proxy) Information Leaflet

Invitation to join the FUTURE-GB study

You are receiving this information leaflet as a friend/relative/partner of yours has been approached to take part in this study. As part of the study they are asked to nominate a friend or relative who would be willing to answer some questionnaires about them (a 'proxy'). As the nominated person, we would like to invite you to take agree to part in a research study (also called a clinical trial), which for you will only involve answering questionnaires.

Before you decide whether to take part or not, it is important that you understand why we are doing this study and what it will involve.

Please take time to read the following information and talk to others about the study. If anything is unclear, or if you would like more information, please ask a member of the study team who will be happy to answer any questions.

What is the purpose of this study?

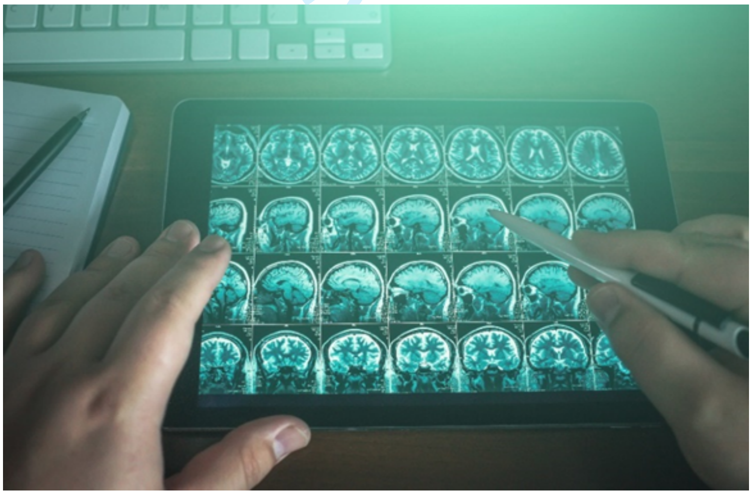

There are many different types of brain tumours. They can vary in how quickly they grow and what symptoms they cause. Studies have shown that when a brain tumour is growing quickly it is better to remove as much tumour as possible. Being able to do this without causing damage to the parts of the brain that are involved in things such as speaking and moving, surgeons need to be able to see clearly parts of the brain during surgery using accurate imaging. This has led to an increase in the use of imaging (such as ultrasound

FUNDED BY NIHR | National Institute for Health Research
OCTRU
SITU
UNIVERSITY OF OXFORD
Imperial College London

For peer review only - http://bmjopen.bmj.com/site/about/guidelines.xhtml

LOCAL TRUST LOGO

and MRI scans) during operations. However, we don't know if all the extra imaging tools do definitely make a difference.

We have been funded by the National Institute of Health Research (NIHR) which receives its funding from the UK Government, to find out whether some of these additional imaging tools available make a positive difference to quality of life for people with brain tumours who have surgery. We will also be looking to see if these imaging tools used during an operation mean people with a brain tumour:

- have a better quality of life?
- if it takes more/less time for their tumour to come back
- if they have more/fewer complications from the surgery

This study will attempt to find a definite answer, so that surgeons know which imaging tools they should use during an operation to enable as much tumour as possible to be removed safely, whilst minimising the risks of damaging brain function and hence affecting quality of life.

The imaging tools that will be used in this study are available across the NHS, and have been shown to be safe. However, no one knows if using them together will have a definite positive effect on outcome for those with a brain tumour. We would encourage you to read the Participant Information Sheet to find out more about the study.

Who is taking part and why have I been invited to take part?

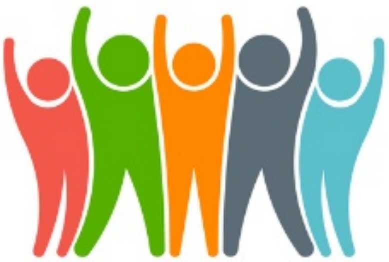

We are hoping to enrol a nominated relative/friend/partner (proxy) from each of the 357 people aged 18 -70 we plan to recruit to the trial, from approximately 15 neurosurgical centres in the UK who have agreed to take part in the FUTURE-GB study.

Specifically, the FUTURE-GB study aims to find out if the new technologies do or do not improve the quality of life of those treated for a brain tumour. We need to know how those taking part are and their abilities during the study, before and after their operation. If your friend/partner/relative becomes unable to answer the questionnaires at some point during the study, we would like to also have answers from you to help us identify any changes in their quality of life over the course of the study. However, if we need to use your answers instead of theirs – we can only do this if we know your answers at the start of the study, so that we can work out what changes have occurred. This is why we would ask both you and your friend/relative/partner to complete the questionnaires throughout the study. The responses that you and your friend/relative/partner give will be used by the trial team to understand the impact of the new technologies on quality of life

Do I have to take part in this study?

No, you are under no obligation to take part in the study. Deciding not to will not affect the treatment/care your friend/relative/partner receives. It is up to you to decide whether to take part or not. Please keep this leaflet and use it as it may to help you make your decision. If you decide to take part, you will be asked to sign a consent form.

FUNDED BY NIHR | National Institute for Health Research OCTRU SITU UNIVERSITY OF OXFORD Imperial College London

For peer review only - http://bmjopen.bmj.com/site/about/guidelines.xhtml

LOCAL TRUST LOGO

What will happen if I take part?

If you are happy to take part in this study, a researcher will contact you to complete questionnaires before your friend/relative/partner's operation, 5 days after their operation or when they leave hospital, 6 weeks after their operation and then every 3 months after your friend/relative/partner's operation for a maximum of 2 years.

Questionnaires take no more than 10 minutes to complete on paper/online, or over the telephone.

Please note if the person who has nominated you withdraws from the study or loses their capacity to consent this will complete your involvement with the study.

What are the benefits and risks of taking part in the study?

For those that take part in the study, your friend/relative/partner's operation will be conducted by the same surgeon/surgical team whom they have already seen.

The information from this study we hope will answer the question:

Which imaging tools should be used by surgeons when removing a glioblastoma, to offer the highest chance of removing as much of the tumour as possible without causing functional problems, and therefore keeping a good quality of life?

We cannot promise the study will help your friend/relative/partner directly, but the more information we collect, the greater the potential to be of benefit, as more of the tumour may be removed.

We are undertaking this study because we want to find out whether the extra imaging tools provide a real benefit for people with brain tumours. These add significant costs to NHS treatment, and so we need to know if they are worth the extra cost.

People sometimes feel uncomfortable answering certain questions about a person's health, or may be unable to answer. If you feel uncomfortable at any point, then you do not have to answer the questions.

Who will know that I am taking part?

The only people who will know that you are taking part in this study are the members of the research team and the person who nominated you. You can tell anyone you would like to that you are taking part.

The only people in the University of Oxford who will have access to information that identifies you will be people who need to contact you to about the study, or review the data. The people who analyse the information will not be able to identify you and will not be able to find out your name or contact details. Representatives from the sponsor, relevant regulatory organisations and [local NHS Trust name] may also need access to monitor or audit the study to ensure that the research is complying with applicable regulations.

Paperwork that is completed by you, your friend/relative/partner, the research team, or the treating clinical team, will be sent securely to the study team managing the FUTURE-GB study, who are based at the University of Oxford.

For peer review only - http://bmjopen.bmj.com/site/about/guidelines.xhtml

LOCAL TRUST LOGO

Will the details be kept confidential?

Yes. All information collected from you during the course of the research, will be kept strictly confidential. Everyone who takes part in the study will be assigned a code number and all of the data relating to each person will be held on a computer database and will only be linked to that code number, and not to people's names or addresses. The study team will record into the study database your name, age, relationship to the study participant, address, and your email address. These details will allow the central study team and the local teams to ensure they are collecting data on the correct person. Your email address will only be used to send you a copy of your consent form for your records and any follow-up questionnaires. This is also the reason your address will be kept on file – in case your questionnaires need to be posted to you to complete.

At the end of the study, all of the data will be de-identified so that no-one can be identified. This de-identified data will be shared so that more researchers can gain a deeper understanding about patients who have had surgery for glioblastoma. It may be shared with other researchers around the world and with commercial organisations but this information will not identify you, and will not be combined with other information in a way that could identify you. The information will only be used for the purpose of healthcare research, and cannot be used to contact you, nor will it affect your care.

In line with what happens in the NHS, the only situation that confidentiality would need to be broken would be if you told a health professional or research team member of something that could result in harm to yourself or others.

What will happen to my data?

Research is carried out in the public interest. The University of Oxford, as Sponsor, is the data controller. This means that we, as University of Oxford researchers, are responsible for looking after your information as part of FUTURE-GB, and using it properly. We will use the minimum possible personally-identifiable information, and this will be kept for 12 months after the study has finished. Non-identifiable research data and any research documents with personal information, will be stored securely at the University of Oxford for a maximum of 5 years after the end of the study, as part of the research record.

The local and central FUTURE-GB study team might use your contact details to get in touch with you. They will keep your identifiable information safely for 12 months after the study has finished. Consent forms and study documents held at [local NHS Trust name] will be archived securely, in accordance with their local procedures.

Data protection regulation provides you with control over your personal data and how it is used. When you agree to your information being used in research, however, some of those rights may be limited, as we need to manage your information in specific ways in order for the research to be reliable and accurate. Further information about your rights with respect to your personal data, is available at https://compliance.web.ox.ac.uk/individual-rights

You can find out more about how we use your information by contacting the FUTURE-GB study team on: futuregb@nds.ox.ac.uk.

FUNDED BY NIHR | National Institute for Health Research

For peer review only - http://bmjopen.bmj.com/site/about/guidelines.xhtml

LOCAL TRUST LOGO

Who is organising and funding the research?

The University of Oxford is the Sponsor and is organising this study. It is being conducted by a research team led by Professor Puneet Plaha, Consultant Neurosurgeon at the Oxford University Hospitals NHS Foundation Trust and the University of Oxford, and Miss Sophie Camp and Prof. Dipankar Nandi (both Consultant Neurosurgeons at Imperial College Healthcare NHS Trust, London).

The National Institute of Health Research – Health Technology Assessment programme is funding the study. The funding for the NIHR comes from the UK Government.

Who has approved this study?

A panel of independent researchers and patient representatives, as well as a Research Ethics Committee (REC Reference 20/LO/0840) have reviewed and approved this study.

What if I have concerns?

The University of Oxford, as the study sponsor, has appropriate insurance in place in the unlikely event that you suffer any harm as a direct consequence of your participation in this study.

If you have any concerns or complaints about any aspect of the study, please contact the FUTURE-GB research team using the details below. You can also contact the University of Oxford Research Governance, Ethics & Assurance office on 01865 616480 or by email on ctrg@admin.ox.ac.uk.

If you would prefer to speak with someone who is not involved in the study, then please contact the Patient Advice and Liaison Service (PALS). PALS is a confidential NHS service that can provide you with support for any complaints or queries you have regarding the care you receive as an NHS patient. However, PALS cannot provide information about this research study.

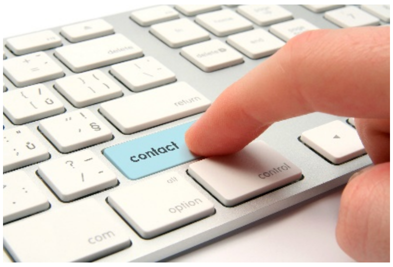

PALS phone number: <Insert local PALS number>

PALS email: <insert local PALS email address>

You can also contact your local clinical team directly:

<local PI/research team name and contact details>

If you have any questions about the study, please contact the FUTURE-GB team on:

Email: futuregb@nds.ox.ac.uk Telephone: 07917 101 649

Postal address: FUTURE-GB study, Botnar Research Centre, Nuffield Orthopaedic Centre, Windmill Road, Oxford, OX3 7LD.

Further information can be found on our study website – futuregb.octru.ox.ac.uk

Thank you for reading this information leaflet and considering taking part.

For peer review only - http://bmjopen.bmj.com/site/about/guidelines.xhtml

University of Oxford Logo

CONSENT FORM (Stage 1-IDEAL Phase)

Imperial Logo

If you agree, **please check box**

1. I confirm that I have read and understood the Information Leaflet dated DDMon20YY version XX. I have had the opportunity to consider the information, ask questions and have had these answered satisfactorily.	
2. I understand that my participation is voluntary and that I am free to withdraw at any time without giving any reason, and without my medical care or legal rights being affected. After withdrawal from the study any data collection from databases will stop.	
3. I understand that relevant sections of my medical notes and data collected during the study may be looked at by individuals from the University of Oxford and Imperial College, London, from regulatory authorities and from the NHS Trusts, where it is relevant to me taking part in this research. I give permission for these individuals to have access to my records.	
4. I consent to the research team holding my contact details so that they can contact me about the study if required. I understand these details will be held securely and destroyed 12 months after the end of the study.	
5. I understand that the information held and maintained by NHS Digital / NHS Central Register may be used to help contact me or provide information about my health status over the next 12 months.<br>I understand and give permission for my NHS/CHI number to be used for this purpose.	
6. I agree to take part in the FUTURE-GB study.	
7. I agree that my operation may be observed for quality assurance purposes by a member of the FUTURE-GB study team. Yes / No	

For peer review only

Name of Participant **Date** **Signature**

Name of Person Taking Consent **Date** **Signature**

For peer review only - http://bmjopen.bmj.com/site/about/guidelines.xhtml

University of Oxford Logo

Imperial Logo

CONSENT FORM

If you agree, **please check box**

1. I confirm that I have read and understood the Information Leaflet dated DDMon20YY version XX. I have had the opportunity to consider the information, ask questions and have had these answered satisfactorily.	
2. I understand that my participation is voluntary and that I am free to withdraw at any time without giving any reason, and without my medical care or legal rights being affected. After withdrawal from the study any data collection from databases will stop.	
3. I understand that relevant sections of my medical notes and data collected during the study may be looked at by individuals from the University of Oxford and Imperial College, London, from regulatory authorities and from the NHS Trusts, where it is relevant to me taking part in this research. I give permission for these individuals to have access to my records.	
4. I consent to the research team holding my contact details so that they can contact me about the study. I understand these details will be held securely and destroyed 12 months after the end of the study.	
5. I agree to my General Practitioner (GP) being informed of my participation in the study.	
6. I understand that the information held and maintained by NHS Digital / NHS Central Register may be used to help contact me or provide information about my health status for the next 24 months. I understand and give permission for my NHS/CHI number to be used for this purpose.	
7. If I complete any questionnaires online or via the telephone regarding the FUTURE-GB study, I agree for these to be passed to the hospital I was recruited at for this study.	
8. I agree to take part in the FUTURE-GB study.	
9. I nominate the following person to be my proxy and to answer questionnaires about me during my time in the FUTURE-GB study. They will no longer be asked to provide information about me if I withdraw from the study or lose capacity. Proxy name: (INSERT NAME HERE)	
10. I agree that my operation may be observed for quality assurance purposes by a member of the FUTURE-GB study team. Yes / No	

Name of Participant **Date** **Signature**

Name of Person Taking Consent **Date** **Signature**

For peer review only - http://bmjopen.bmj.com/site/about/guidelines.xhtml

University of Oxford Logo

FUTURE GB **PROXY CONSENT FORM**

Imperial Logo

	If you agree, **please check box**
1. I confirm that I have read and understood the Proxy Information Leaflet dated DDMon20YY version XX. I have had the opportunity to consider the information, ask questions and have had these answered satisfactorily.	
2. I understand that my participation is voluntary and that I am free to withdraw at any time without giving any reason, and without my relative/friend's medical care or legal rights being affected.	
3. I consent to the research team holding my contact details so that they can contact me about the study. I understand these details will be held securely and destroyed at the 12 months after the end of the study.	
4. I understand that as long as I feel able to I will complete regular questionnaires about the abilities and quality of life of the person I have been nominated to be the Proxy of. I understand if the person who nominated me withdraws or loses capacity – this will also end my participation.	
5. I agree to take part in the FUTURE-GB study as a Proxy Representative.	

Name of Participant:		
Relationship to the participant:	Partner ☐ Family member ☐ Carer ☐ Friend ☐<br>Other (please specify) ______	
Approximately how much time do you spend with the FUTURE-GB participant per week?	I am in contact with them daily ☐<br>I am in contact with them every few days ☐<br>I am in contact with them weekly ☐<br>I am in contact with them every 2 weeks ☐<br>I am in contact with them monthly ☐	
Name of Proxy Representative	Signature:	Date:
Name of Person Taking Consent	Signature:	Date:

Deleted: FUTUREGB_ProxyICF_V3.0_10Jun2021_clean.docx

FUTUREGB_ProxyICF_V3.0_10Jun2021.docx IRAS ID: 264482
Co- Investigator: Prof Puneet Plaha, Ms Sophie Camp and Prof Dipankar Nandi.

For peer review only

Reporting checklist for protocol of a clinical trial.

Based on the SPIRIT guidelines.

Instructions to authors

Complete this checklist by entering the page numbers from your manuscript where readers will find each of the items listed below.

Your article may not currently address all the items on the checklist. Please modify your text to include the missing information. If you are certain that an item does not apply, please write "n/a" and provide a short explanation.

Upload your completed checklist as an extra file when you submit to a journal.

In your methods section, say that you used the SPIRITreporting guidelines, and cite them as:

Chan A-W, Tetzlaff JM, Gøtzsche PC, Altman DG, Mann H, Berlin J, Dickersin K, Hróbjartsson A, Schulz KF, Parulekar WR, Krleža-Jerić K, Laupacis A, Moher D. SPIRIT 2013 Explanation and Elaboration: Guidance for protocols of clinical trials. BMJ. 2013;346:e7586

		Reporting Item	Page Number
**Administrative information**			
Title	#1	Descriptive title identifying the study design, population, interventions, and, if applicable, trial acronym	1
Trial registration	#2a	Trial identifier and registry name. If not yet registered, name of intended registry	2
Trial registration: data set	#2b	All items from the World Health Organization Trial Registration Data Set	
Protocol version	#3	Date and version identifier	1
Funding	#4	Sources and types of financial, material, and other support	14
Roles and responsibilities: contributorship	#5a	Names, affiliations, and roles of protocol contributors	1
Roles and	#5b	Name and contact information for the trial sponsor	

For peer review only

responsibilities: sponsor contact information			
Roles and responsibilities: sponsor and funder	#5c	Role of study sponsor and funders, if any, in study design; collection, management, analysis, and interpretation of data; writing of the report; and the decision to submit the report for publication, including whether they will have ultimate authority over any of these activities	
Roles and responsibilities: committees	#5d	Composition, roles, and responsibilities of the coordinating centre, steering committee, endpoint adjudication committee, data management team, and other individuals or groups overseeing the trial, if applicable (see Item 21a for data monitoring committee)	
**Introduction**			
Background and rationale	#6a	Description of research question and justification for undertaking the trial, including summary of relevant studies (published and unpublished) examining benefits and harms for each intervention	4-5
Background and rationale: choice of comparators	#6b	Explanation for choice of comparators	4-5
Objectives	#7	Specific objectives or hypotheses	6
Trial design	#8	Description of trial design including type of trial (eg, parallel group, crossover, factorial, single group), allocation ratio, and framework (eg, superiority, equivalence, non-inferiority, exploratory)	6
**Methods: Participants, interventions, and outcomes**			
Study setting	#9	Description of study settings (eg, community clinic, academic hospital) and list of countries where data will be collected. Reference to where list of study sites can be obtained	6

For peer review only

Eligibility criteria	#10	Inclusion and exclusion criteria for participants. If applicable, eligibility criteria for study centres and individuals who will perform the interventions (eg, surgeons, psychotherapists)	7
Interventions: description	#11a	Interventions for each group with sufficient detail to allow replication, including how and when they will be administered	6-7
Interventions: modifications	#11b	Criteria for discontinuing or modifying allocated interventions for a given trial participant (eg, drug dose change in response to harms, participant request, or improving / worsening disease)	6
Interventions: adherance	#11c	Strategies to improve adherence to intervention protocols, and any procedures for monitoring adherence (eg, drug tablet return; laboratory tests)	
Interventions: concomitant care	#11d	Relevant concomitant care and interventions that are permitted or prohibited during the trial	
Outcomes	#12	Primary, secondary, and other outcomes, including the specific measurement variable (eg, systolic blood pressure), analysis metric (eg, change from baseline, final value, time to event), method of aggregation (eg, median, proportion), and time point for each outcome. Explanation of the clinical relevance of chosen efficacy and harm outcomes is strongly recommended	5, table 1
Participant timeline	#13	Time schedule of enrolment, interventions (including any run-ins and washouts), assessments, and visits for participants. A schematic diagram is highly recommended (see Figure)	Fig 1
Sample size	#14	Estimated number of participants needed to achieve study objectives and how it was determined, including clinical and statistical assumptions supporting any sample size calculations	9
Recruitment	#15	Strategies for achieving adequate participant enrolment to reach target sample size	8
**Methods: Assignment of**			

For peer review only

**interventions (for controlled trials)**			
Allocation: sequence generation	#16a	Method of generating the allocation sequence (eg, computer-generated random numbers), and list of any factors for stratification. To reduce predictability of a random sequence, details of any planned restriction (eg, blocking) should be provided in a separate document that is unavailable to those who enrol participants or assign interventions	8
Allocation concealment mechanism	#16b	Mechanism of implementing the allocation sequence (eg, central telephone; sequentially numbered, opaque, sealed envelopes), describing any steps to conceal the sequence until interventions are assigned	8
Allocation: implementation	#16c	Who will generate the allocation sequence, who will enrol participants, and who will assign participants to interventions	8
Blinding (masking)	#17a	Who will be blinded after assignment to interventions (eg, trial participants, care providers, outcome assessors, data analysts), and how	8
Blinding (masking): emergency unblinding	#17b	If blinded, circumstances under which unblinding is permissible, and procedure for revealing a participant's allocated intervention during the trial	8
**Methods: Data collection, management, and analysis**			
Data collection plan	#18a	Plans for assessment and collection of outcome, baseline, and other trial data, including any related processes to promote data quality (eg, duplicate measurements, training of assessors) and a description of study instruments (eg, questionnaires, laboratory tests) along with their reliability and validity, if known. Reference to where data collection forms can be found, if not in the protocol	10
Data collection plan: retention	#18b	Plans to promote participant retention and complete follow-up, including list of any outcome data to be collected for participants who discontinue or deviate from intervention protocols	10

For peer review only

Data management	#19	Plans for data entry, coding, security, and storage, including any related processes to promote data quality (eg, double data entry; range checks for data values). Reference to where details of data management procedures can be found, if not in the protocol	10
Statistics: outcomes	#20a	Statistical methods for analysing primary and secondary outcomes. Reference to where other details of the statistical analysis plan can be found, if not in the protocol	9
Statistics: additional analyses	#20b	Methods for any additional analyses (eg, subgroup and adjusted analyses)	9
Statistics: analysis population and missing data	#20c	Definition of analysis population relating to protocol non-adherence (eg, as randomised analysis), and any statistical methods to handle missing data (eg, multiple imputation)	9
**Methods: Monitoring**			
Data monitoring: formal committee	#21a	Composition of data monitoring committee (DMC); summary of its role and reporting structure; statement of whether it is independent from the sponsor and competing interests; and reference to where further details about its charter can be found, if not in the protocol. Alternatively, an explanation of why a DMC is not needed	10-11
Data monitoring: interim analysis	#21b	Description of any interim analyses and stopping guidelines, including who will have access to these interim results and make the final decision to terminate the trial	
Harms	#22	Plans for collecting, assessing, reporting, and managing solicited and spontaneously reported adverse events and other unintended effects of trial interventions or trial conduct	
Auditing	#23	Frequency and procedures for auditing trial conduct, if any, and whether the process will be independent from investigators and the sponsor	10
**Ethics and dissemination**			
Research ethics approval	#24	Plans for seeking research ethics committee / institutional review board (REC / IRB) approval	11

For peer review only

Protocol amendments	#25	Plans for communicating important protocol modifications (eg, changes to eligibility criteria, outcomes, analyses) to relevant parties (eg, investigators, REC / IRBs, trial participants, trial registries, journals, regulators)	
Consent or assent	#26a	Who will obtain informed consent or assent from potential trial participants or authorised surrogates, and how (see Item 32)	8
Consent or assent: ancillary studies	#26b	Additional consent provisions for collection and use of participant data and biological specimens in ancillary studies, if applicable	
Confidentiality	#27	How personal information about potential and enrolled participants will be collected, shared, and maintained in order to protect confidentiality before, during, and after the trial	10
Declaration of interests	#28	Financial and other competing interests for principal investigators for the overall trial and each study site	14
Data access	#29	Statement of who will have access to the final trial dataset, and disclosure of contractual agreements that limit such access for investigators	10
Ancillary and post trial care	#30	Provisions, if any, for ancillary and post-trial care, and for compensation to those who suffer harm from trial participation	
Dissemination policy: trial results	#31a	Plans for investigators and sponsor to communicate trial results to participants, healthcare professionals, the public, and other relevant groups (eg, via publication, reporting in results databases, or other data sharing arrangements), including any publication restrictions	
Dissemination policy: authorship	#31b	Authorship eligibility guidelines and any intended use of professional writers	
Dissemination policy: reproducible research	#31c	Plans, if any, for granting public access to the full protocol, participant-level dataset, and statistical code	
**Appendices**			
Informed consent materials	#32	Model consent form and other related documentation given to participants and authorised surrogates	Supplementary files

For peer review only

Biological specimens	#33	Plans for collection, laboratory evaluation, and storage of biological specimens for genetic or molecular analysis in the current trial and for future use in ancillary studies, if applicable

None The SPIRIT Explanation and Elaboration paper is distributed under the terms of the Creative Commons Attribution License CC-BY-NC. This checklist can be completed online using https://www.goodreports.org/, a tool made by the EQUATOR Network in collaboration with Penelope.ai

For peer review only

BMJ Open

FUTURE-GB – Functional and Ultrasound guided Resection of Glioblastoma. A two-stage randomised control trial.

Journal:	*BMJ Open*
Manuscript ID	bmjopen-2022-064823.R2
Article Type:	Protocol
Date Submitted by the Author:	05-Sep-2022
Complete List of Authors:	Plaha, Puneet; Oxford University Hospitals NHS Foundation Trust, Department of Neursurgery; University of Oxford, Nuffield Department of Surgical Sciences<br>Camp, Sophie; Imperial College Healthcare NHS Trust, Neurosurgery<br>Cook, Jonathan; University of Oxford Nuffield Department of Orthopaedics Rheumatology and Musculoskeletal Sciences, Oxford Clinical Trials Research Unit & Surgical Intervention Trials Unit<br>McCulloch, Peter; Oxford University, Nuffield Department of Surgical Sciences<br>Voets, Natalie; Oxford University, Nuffield Department of Clinical Neurosciences<br>Ma, Ruichong; Oxford University Hospitals NHS Foundation Trust, Department of Neurosurgery; University of Oxford, Nuffield Department of Surgical Sciences<br>Taphoorn, Martin J.B.; Leiden University Medical Center; Haaglanden Medical Center Bronovo<br>Dirven, Linda; Leiden University Medical Center<br>Grech-Sollars, Matthew; UCL, Department of computer sciences; National Hospital for Neurology and Neurosurgery, Lysholm Department of Neuroradiology<br>Watts, Colin; University of Birmingham, Institute of Cancer and Genomic Studies; University Hospitals Birmingham NHS Foundation Trust, Department of Neurosurgery<br>Bulbeck, Helen; Brainstrust<br>Jenkinson, Michael; University of Liverpool; Walton Centre for Neurology and Neurosurgery<br>Williams, Matthew; Imperial College Healthcare NHS Trust<br>Lim, Adrian; Imperial College London<br>Dixon, Luke; Imperial College Healthcare NHS Trust, Neuroradiology<br>Price, Stephen; Cambridge University, Neurosurgery Division, Dept. Clinical Neurosciences<br>Ashkan, Keyoumars; King's College Hospital<br>Apostolopoulos, Vasileios; Oxford University Hospitals NHS Foundation Trust, Department of Neursurgery<br>Barber, Vicki; University of Oxford Nuffield Department of Orthopaedics Rheumatology and Musculoskeletal Sciences, Oxford Clinical Trials Research Unit & Surgical Intervention Trials Unit<br>Taylor, Amy; University of Oxford, Nuffield Department of Surgical Sciences<br>GB, FUTURE; Oxford University<br>Nandi, Dipankar; Imperial College Healthcare NHS Trust, Neurosurgery;

	Imperial College London
**Primary Subject Heading**:	Oncology
Secondary Subject Heading:	Surgery, Patient-centred medicine
Keywords:	Neurosurgery < SURGERY, Clinical trials < THERAPEUTICS, SURGERY

SCHOLARONE™
Manuscripts

FUTURE-GB – Functional and Ultrasound guided Resection of Glioblastoma. A two-stage randomised control trial.

Puneet Plaha[1,2], Sophie Camp[3], Jonathan A Cook[4], Peter McCulloch[2], Natalie Voets[5], Ruichong Ma[1,2], Martin Taphoorn[6,7], Linda Dirven[6], Matthew Grech-Sollars[8,9,10], Colin Watts[11,12], Helen Bulbeck[13], Michael D Jenkinson[14], Matt Williams[3], Adrian Lim[10], Luke Dixon[3], Stephen Price[15], Keyoumars Ashkan[16], Vasileios Apostolopoulos[1], Vicki S Barber[4], Amy Taylor[2], FUTURE-GB Collaborators, Dipankar Nandi[3,10]

[1]Department of Neurosurgery, Oxford University Hospitals NHS Foundation Trust, Oxford, UK
[2]Nuffield Department of Surgical Sciences, University of Oxford, UK
[3]Imperial College Healthcare NHS Trust, London, UK
[4]Oxford Clinical Trials Research Unit & Surgical Intervention Trials Unit, Nuffield Department of Orthopaedics Rheumatology and Musculoskeletal Sciences, University of Oxford, UK
[5]Nuffield Department of Clinical Neurosciences, University of Oxford, UK
[6]Department of Neurology, Leiden University Medical Center, Leiden, Netherlands
[7]Department of Neurology, Haaglanden Medical Center, The Hague, Netherlands
[8]
[9]
[10]Imperial College London, London, UK
[11]Institute of Cancer and Genomic Sciences, University of Birmingham, UK
[12] Department of Neurosurgery, University Hospitals Birmingham Foundation Trust, UK
[13]brainstrust = the brain cancer people, UK
[14]University of Liverpool, Liverpool, UK
[15]Department of Neurosurgery, Cambridge University Hospitals NHS Foundation Trust, Cambridge, UK
[16]Deparment of Neurosurgery, King's College Hospital NHS Foundation Trust, London, UK

FUTURE-GB collaborators:
Giles Critchley (Brighton), George Eralil, Kathrin Whitehouse (Cardiff), Anna Solth (Dundee), Paul M Brennan (Edinburgh), Chittoor Rajaraman, Shailendra Achawal (Hull), Anil Varma (Middlesborough), Robert Corns, Ganamurthy Sivakumar (Leeds), Farouk Olubajo (Liverpool), Edward McKintosh, Grainne McKenna and Dimitrios Paraskevopoulos (Barts Health), Neil Barua (Bristol), James Palmer (Plymouth), Stuart Smith (Nottingham), Paul Grundy (Southampton), Erminia Albanese, Huan Chan (Stoke-on-Trent), Damian Holliman (Newcastle), Jose Lavrador, Maria Velicu (King's College Hospital)

Corresponding Author: Puneet Plaha (puneet.plaha@ouh.nhs.uk)

Word Count: 3977 excluding abstract, references, tables and figure legends.

For peer review only

Abstract

Introduction

Surgery remains the mainstay for treatment of primary glioblastoma, followed by radiotherapy and chemotherapy. Current standard of care during surgery involves the intraoperative use of image-guidance and 5-ALA. There are multiple other surgical adjuncts available to the neuro-oncology surgeon. However, access to, and utilisation of these varies widely in UK practice, with limited evidence of their utility. The aim of this trial is to investigate whether the addition of Diffusion Tensor Imaging (DTI) and intraoperative ultrasound (iUS) to standard of care surgery (Intra operative Neuronavigation and 5-ALA) impacts on deterioration free survival (DFS).

Methods and Analysis

This is a two-stage randomised control trial (RCT) consisting of an initial non-randomised cohort study based on the principles of the IDEAL Stage-IIb format, followed by a statistically powered randomised trial comparing the addition of DTI and iUS to standard of care surgery. A total of 357 patients will be recruited for the RCT. The primary outcome is DFS, defined as the time to either 10-point deterioration in HRQoL scores from baseline, without subsequent reversal, progressive disease, or death.

Ethics and Dissemination

The trial was registered in the Integrated Research Application System (Ref: 264482) and approved by a UK research and ethics committee (Ref: 20/LO/0840). Results will be published in a peer reviewed journal. Further dissemination to participants, patient groups and the wider medical community will utilize a range of approaches to maximize impact.

Registration details

ISRCTN: 38834571

Keywords

Glioblastoma, IDEAL, randomised control trial, DTI, intraoperative ultrasound

For peer review only

Article Summary

Strengths of trial:

- This is a randomised control trial comparing the quality of life of glioblastoma patients undergoing standard of care surgery (Intraoperative neuronavigation and 5-ALA) vs surgery with the addition of Diffusion Tensor imaging (DTI) and intraoperative ultrasound (iUS).
- To ensure standardisation and quality control of delivery of the DTI and iUS in the randomised trial, sites will be required to enter a minimum number of patients into an initial IDEAL Stage-IIB study prior to commencing recruitment to the randomised trial.
- Patient public involvement (PPI) determined the primary outcome measure of deterioration free survival (comprising a decline in health-related quality of life, disease progression or death), rather than overall survival. DFS is considered by patients to be most pertinent.

Limitations of the trial:

- The trial recruits patients aged 18-70years who can undergo surgery to maximally resect their glioblastoma.
- There is variability of the intraoperative ultrasound machines used by trial sites (sites use machines they are familiar with). However, this reflects real world iUS usage.

For peer review only

Introduction

Glioblastoma (GB) is the most frequent and aggressive form of primary brain cancer, with an incidence of 4.64/100,000persons/year in the UK[1]. Prognosis remains extremely poor with median survival of approximately 15 months[2], and as the tumour grows, patients experience a progressive decline in health-related quality of life (HRQoL), and caregivers report high levels of distress and carer burden[3]. Resistance to treatment leads to poor survival, with high costs to the patient, relatives, society, and the economy[4,5]. Although primary brain tumours represent only 3% of all cancers, a brain tumour reduces life expectancy by an average of 20years, the highest of any cancer, and accounts for more average years of life lost than any other cancer[4,5]. GB affects adults in their economic prime, and is a leading cause of death in those under 40years, costing the economy £578M per year[4,5]. To date, there has been little progress in improving outcome including quality of life, with many trials failing to show an effect[6].

Surgery is the mainstay of treatment for GB, but optimum surgical technologies remain unclear. Surgery to resect GB is integral to maximum first line treatment, with a greater impact on survival than non-operative treatments (radiotherapy and chemotherapy)[7]. It improves symptom control, reduces dependence on dexamethasone, and increases progression free (PFS) and overall survival (OS)[8,9]. However, maximising the extent of surgical resection must be balanced against the potential risk of causing neurological deficit, and hence impacting negatively on a patient's ability to tolerate adjuvant treatments.

The desire to achieve a safe, maximal resection, particularly in eloquent regions, has led to an increase in the use of intraoperative imaging. This attempts to eliminate the error produced by brain shift, an inherent problem in navigation systems based on preoperative imaging[6], to demonstrate residual tumour at operation, and to visualise accurately relevant white matter tracts and tumour margins. Two technologies that facilitate surgical resection intraoperatively are iUS and DTI.

1. iUS accommodates for brain shift if it is linked to neuronavigation systems, allowing the surgeon to track tumour resection in real time. iUS permits multiple, real time image acquisitions, and, potentially, if navigated, at each stage, comparison with the preoperative MRI navigation sequence, to evaluate brain shift and residual disease. iUS minimally augments operative time[6], allowing precise visualization of tumour resection. It is user friendly, widely available, and a pragmatic and cost-effective alternative to intraoperative MRI, which is prohibitively expensive for many UK units. iUS, and more recently navigated iUS, has a long history in brain tumour surgery[10,11], facilitating/extending resection[12–16], and improving survival[17]. It has also been evaluated with respect to histology[18,19]. However, there is a learning curve, and image interpretation, especially during resection, can be challenging[10]. iUS demonstrates residual tumour in real time. Indeed, it has been reported that navigated iUS and 5-ALA provide different information of tumour extent, and when combined, enhance extent of resection[20]. Despite this, there are no randomised trials assessing its efficacy.

2. DTI is a special magnetic resonance imaging (MRI) technique that can identify the location of white matter nerve tracts important for speech/language/visual/motor

For peer review only - http://bmjopen.bmj.com/site/about/guidelines.xhtml

functions. The location of white matter fibre pathways is the most frequent reason why surgery is halted early, to avoid compromising patient function[21]. DTI is the only method available to visualise functionally important white matter tracts in the vicinity of a tumour before surgery and can be fused with standard intraoperative navigation systems to enable visualisation of the spatial location of the tracts during surgery, allowing removal of tumour in close proximity. The usefulness of DTI in brain tumour surgery has recently been reviewed[21]. Intraoperative visualisation of DTI is reported to contribute to maximising safe resection[22–24], reducing visual field deficits[25], and predicting long term language problems after surgery[26]. A single centre randomised control trial (RCT), comparing DTI vs no-DTI, showed that DTI led to significantly better gross total resection (GTR) rates, a lower risk of movement loss, and improved life expectancy[27]. Furthermore, DTI-informed awake surgery reduced the occurrence and severity of behavioural problems postoperatively, leading to faster recovery, and shorter hospital stay[28]. DTI requires the collection of additional MRI data, specialist software for analysis, and detailed knowledge of white matter anatomy and function. In addition, tract visualisation may be restricted where there is peritumoural oedema. As a result, there is only limited data available on the sensitivity and specificity of DTI in GB surgery, particularly with reference to its value as an intraoperative tool and in predicting DFS.

There are wide variations of surgical standard of care across the UK. A survey of all 24 adult UK neurosurgical centres (telephone and email survey conducted in 2018 by the Oxford researchers), showed wide variation in the use of technologies employed during GB resection. Whilst all centres employ standard neuronavigation and 5-ALA, only 75% have access to iUS, 62% to DTI, and 16% to an intraoperative MRI scanner. It remains unclear which technologies should be employed intraoperatively, without worsening neurological function. Indeed, most of these technologies are not regularly used for tumour resection, with surgeons unclear of the efficacy of each, and what is the optimum combination. A recent Cochrane review emphasized the lack of high-quality evidence to support the use of any specific intraoperative imaging technology[29]. The National Institute for Health and Care Excellence (NICE) guidance[3] has suggested that the available range of intraoperative technologies are considered, as appropriate, in addition to standard techniques, for tumour resection.

The Functional and UlTrasound gUided Resection of GlioBlastoma – FUTURE-GB trial was developed in collaboration with the Society of British Neurosurgeons (SBNS) and multiple GB patient advocate groups to try and address some of the deficits in knowledge regarding the utility of addition surgical adjuncts. FUTURE-GB aims to evaluate the impact of DTI and iUS in addition to standard of care techniques with a view to providing high-quality evidence to shape standard practice in the future.

Methods and Analysis

Trial design and setting

For peer review only

FUTURE-GB is a two-stage trial (figure 1). Patient and public involvement (PPI) actively informed the rationale, design and development of the protocol and patient facing materials of the trial.

Stage-1: non-randomised multicentre learning and evaluation stage (IDEAL Stage IIB trial)
Stage-1 is a non-randomised, multicentre learning and harmonisation stage in which quality control measures and mentoring will be employed, to improve and evaluate standards of practice based on the principles of an IDEAL Framework Stage-IIB study[30,31]. It will evaluate standard care surgery with the addition of DTI imaging and the ultrasound imaging during the operation. This will ensure that the surgeons using the technologies to be employed in the RCT demonstrate acceptable expertise in delivering the new approach prior to proceeding with the randomisation stage. This stage ensures standardisation of the use of the technologies across all trial centres by expert mentoring, and will evaluate quality of delivery, including monitoring of the learning curve for the group as a whole.

Stage-1 is divided into 3 components:

1. Pre-trial Webinar
2. IDEAL Stage-IIB (Quality assurance, Mentoring and Trial centres evaluation)
3. End of Stage-1, Pre-Stage-2 RCT, each participating centre will have a data workflow review with the Lead Investigators to review the cases completed in Stage-1.

The IDEAL Framework Stage-IIB trial will comprise the following:

- Mentoring for local site surgeons.
- Quality assurance of operative procedure.

Mentoring by the CI and Lead Investigators will be provided through visits to participating centres and frequent meetings, together with a helpline for individual advice sessions from the CI and Lead Investigators and co-applicants, as appropriate. Neurosurgeons will contribute data to ensure standardisation of the protocol and acceptable expertise in delivering the new approach (supplementary files1,2). This will be evaluated using the following metrics: operation length; successful use of DTI neuronavigation and iUS to achieve maximal safe tumour resection without major neurological deficit; and extent of tumour resection assessed on postoperative MRI scan. The number of cases required for this may vary but is expected to be small (up to 5 cases) as most surgeons are already familiar with the component techniques and are not anticipated to require substantial assessment. Ensuring all participating surgeons are ready to take part will minimize performance bias in Stage-2 and ensure standardisation of intraoperative technique.

Stage-2: prospective, Stage-III, multicentre RCT with internal pilot
This is a parallel group two arm, multicentre, RCT. Patients who agree to take part will be allocated by chance. The trial will enrol 357 newly diagnosed patients with GB and will randomly allocate them to receive either surgical resection with standard methods without US and DTI, or this surgery with the addition of US and DTI, as well as standard tools. Patients will not know into which group they have been placed, nor will the research team assessing them before and after surgery. Patients will be recruited from at least 15 NHS hospitals that routinely undertake GB surgery and have access to these tools. The trial will be embedded within existing care pathways. After agreeing to take part, participants will be

asked to complete questionnaires (supplementary files3-7) about their health-related quality of life (HRQoL, reflecting symptoms as well as physical, emotional and psychological functioning. They will also have a brief physical and cognitive/functional assessment before their surgery. Afterwards, the questionnaires and assessments will be repeated, before leaving hospital, and at three monthly intervals until 24months after randomisation. These will be combined with planned hospital visits. OS will also be recorded. See Figure 1 for a Flowchart of the trial.

Population: 357 participants with GB suitable for maximal, safe resective surgery (attempted GTR of all enhancing tumour), as agreed at the local neuro-oncology Multi-Disciplinary Team (MDT) meeting.

Intervention: standard care surgery (neuronavigation based on preoperative imaging and intraoperative use of 5-ALA) with the addition of DTI neuronavigation and iUS.

Control: standard care surgery (neuronavigation based on preoperative imaging and intraoperative use of 5-ALA)

Outcome: Deterioration Free Survival, defined as the time to a 10-point deterioration in HRQoL scores from baseline, without subsequent 10-point improvement in scores compared with baseline; or progressive disease; or death in the absence of previous definitive deterioration before the next assessment. HRQoL is measured with the EORTC QLQ-C30 and QLQ-BN20 questionnaires.

Setting: At least 15 UK NHS Trusts undertaking GB surgery

Eligibility
Patients aged 18-70years with a primary GB tumour which is deemed maximally resectable (attempted GTR of all enhancing tumour) by the local neuro-oncology MDT meeting, will be potentially suitable for inclusion in the trial.
Inclusion Criteria

- Age 18-70years
- Neuro-oncology MDT decision that the imaging shows a primary GB tumour which is maximally resectable (attempted GTR of all contrast-enhancing tumour)
- Patient is suitable for concomintant adjuvant radiotherapy and Temozolomide (TMZ) chemotherapy followed by adjuvant TMZ at the time of MDT decision
- Able to receive 5-ALA
- Willing and able to give informed consent
- Able to complete trial questionnaires, this may be with support where English is not their first language (where compatible with the validation of questionnaires) (Stage-2 only)
- Able to provide a proxy who is willing to complete questionnaires as requested (Stage-2 only).

Exclusion Criteria
The participant may not enter the trial if any of the following apply:

For peer review only

- Midline/basal ganglia/cerebellum/brainstem GB
- Multifocal GB
- Recurrent GB
- Suspected secondary GB
- Contraindication to MRI

Proxy Inclusion (Stage-2 only)
Although it is widely recognised in HRQoL research that an individual may rate aspects of their functioning and well-being differently from how another person might, even if that person is close to the individual (e.g. carer, partner etc.), we will ask proxies to also rate HRQoL aspects of patients during the RCT.

The proxy/participant assessment is particularly important in cases where the patients are not able to complete the questionnaires, for example if have disease progression, or if their condition is too poor. These proxy measures can be used as substitute data in case the patient's rating of their HRQoL is lacking. When a participant dies, loses capacity, or withdraws from the trial – this will also automatically cease the proxy's involvement in the trial.

Inclusion Criteria for proxy
- Age 18-75years
- Nominated by an individual who has consented to participate in Stage-2
- Willing and able to give informed consent
- Able to understand written English to enable completion of trial questionnaires

Recruitment
Recruitment into the trial will be undertaken in two phases in conjunction with the separate stages of the trial. There will be a separate Patient Information Sheet and Consent Form for patients entering Stage-1 (IDEAL IIb) and Stage-2 (RCT) (supplementary files8-13). The stages are sequential at participating sites and the stages cannot be recruited to in parallel.

All potentially eligible participants will have the trial mentioned at the same time the options regarding their surgery are discussed. Depending upon the site, the resources available, and most importantly how the participant is dealing with their diagnosis, the recruitment process and approach may vary across and within sites. Potential participants may straight away be provided with the trial participant information sheet and asked to consider the trial, and that a member of the local research team will contact them. It may be the case that individuals are asked if it would be acceptable for their name to be passed to the research team who will make contact at a later timepoint, or potential participants may be given the participant information sheet and asked to call the number on it if they wish to find out more about the trial.

Randomisation
Randomisation of patients will only occur in Stage-2 of the trial. Every centre and each participating surgeon will offer surgery under both arms of the trial. Randomisation will be via the web-based service provided by OCTRU, using the method of minimisation. The minimisation factors will be trial site, age (≤55yrs or >55yrs), expected surgery status (under

For peer review only

general anaesthesia or awake), and eloquence of tumour location (non-eloquent or eloquent).

Participants will be randomised on a 1:1 basis, after having given written consent; however, they will remain blinded as to which arm of the trial they have been allocated. The local clinical team at site will receive an email from the randomisation system detailing the arm of the trial to which a participant has been randomised. Randomisation must occur before the pre-operative imaging takes place so that the assigned trial pre-operative imaging can be undertaken.

Pre and post randomisation withdrawals

Participants may decline to continue to take part in the trial at any time without prejudice. A decision not to participate or withdraw will not affect the standard of care the patient receives. Once withdrawn, the patient will be advised to discuss their further care plan with their surgeon. On withdrawal of the patient, any data collected up until the time of withdrawal will be retained by the research team and included in the final analysis.

Blinding

Stage-1 is not blinded; the participants will be receiving all the technologies during their surgery.

In Stage-2, the participant will be blinded to the allocation (intervention or control arm), and the treating clinician will be aware of the need to perform the surgery with the intraoperative technologies as allocated. In addition to the participant, the radiologist (reviewing the postoperative MRI) will be blinded to the trial arm. Given this, only on the operation CRF will data of the allocation be included.

Trial treatments

All participants will undergo surgery for removal of their GB. The choice of anaesthesia will be left to the discretion of the treating surgeon/anaesthetist/patient as per their normal practice and preference.

The trial will compare two imaging techniques for imaging the tumour. Participants will be randomised to either:

- Standard care surgery (neuronavigation based on preoperative imaging and intraoperative use of 5-ALA) (Control arm)
- Standard care surgery (neuronavigation based on preoperative imaging and intraoperative use of 5-ALA) **AND** of DTI neuronavigation and iUS (Intervention arm)

Objectives and Outcome Measures

Objectives and outcome measures are summarised in table 1.

For peer review only

Stage 1	Objectives	Outcome Measures	Timepoint(s)
**Primary** Outcomes	To demonstrate the feasibility of using DTI and iUS* in addition to standard of care (neuronavigation based on preoperative MRI and intraoperative use of 5-ALA) for neurosurgery (at selected UK NHS hospitals).	1. Operation length<br>2. Successful use of DTI neuronavigation and iUS* to achieve maximal safe tumour resection without major neurological deficit<br>3. Extent of tumour resection assessed on postoperative MRI scan.<br>4. Surgical Complication and Serious Adverse Events	Hospital discharge and 6 months post-op.
**Stage 2**	**Objectives**	**Outcome Measures**	**Timepoint(s)**
**Primary** Outcomes	To assess whether additional intraoperative imaging (DTI and iUS*) to standard of care (neuronavigation based on preoperative MRI scan and intraoperative 5-ALA) improves Deterioration Free Survival (DFS) (Where deterioration relates to global health status only)	Composite of global health status domain of the QLQ-C30 questionnaire, Progression Free Survival (PFS) and Overall Survival (OS) with an event defined as either deterioration, progression or death.	To be recorded at baseline; 6wks post-op., 3mths post-op., and then 3mthly up to 24 months
**Secondary** Outcomes	To assess whether additional intraoperative imaging (DTI and iUS*) to standard of care (Neuronavigation and intraoperative 5-ALA) improves DFS where deterioration relates to physical functioning, social functioning from the QLQ-C30, and motor dysfunction and communication deficit	4 composites using the respective domain of QLQ-C30 (physical functioning and social functioning) and BN20 (motor dysfunction and communication deficit) combined with PFS and OS.	To be recorded at baseline; 6wks post-op., 3mths post-op., and then 3mthly up to 24 months
**Secondary** Outcomes	To assess whether additional intraoperative imaging (DTI and iUS*) to standard of care (Neuronavigation and intraoperative 5-ALA) improves time to deterioration	Defined similar to DFS with the exception that progression is excluded as an event (i.e. only deterioration or death are considered). There will be five time to deterioration outcomes, one for each of the domains utilised in the primary and secondary DFS outcomes, used in turn to define deterioration.	To be recorded at 6wks post-op., 3mths post-op., and then 3mthly up to 24 months
**Secondary** Outcomes	To assess whether additional intraoperative imaging (DTI and IUS*) to standard of care (Neuronavigation and intraoperative 5-ALA) improves Overall Survival (OS)	OS (time from randomisation to death or trial closure)	To be recorded at 24 months
**Secondary** Outcomes	To assess whether additional intraoperative imaging (DTI and iUS*) to standard of care (Neuronavigation and intraoperative 5-ALA) improves Progression Free Survival (PFS)	PFS (time from randomisation to radiological tumour progression on imaging, as agreed in local MDT	MRI at 6 months post-op., and then 3mthly up to 24 months or an MRI performed outside protocol if patient is symptomatic
**Secondary** Outcomes	To assess whether additional intraoperative imaging (DTI and iUS*) to standard of care (Neuronavigation and intraoperative 5-ALA) improves the extent of tumour resection	Extent of resection as volume of residual tumour postoperative contrast enhanced MRI. Extent of resection as % of pre-operative tumour volume on postoperative contrast enhanced MRI.	Post-operative review

**Secondary** Outcomes	To assess whether additional intraoperative imaging (DTI and iUS*) to standard of care (Neuronavigation and intraoperative 5-ALA) improves the incidence of surgical complications	Number and type of surgical complications	To be recorded at 5 days post-op, or discharge date (whichever is soonest); 6wks post-op., 3mths post-op., and then 3mthly up to 24 months
**Secondary** Outcomes	To assess whether additional intraoperative imaging (DTI and iUS*) to standard of care (Neuronavigation and intraoperative 5-ALA) improves the number of patients eligible for adjuvant treatment following surgery	Number of patients eligible for adjuvant treatment	3mths post-op.
**Secondary** Outcomes	To assess whether additional intraoperative imaging (DTI and iUS*) to standard of care (Neuronavigation and intraoperative 5-ALA) improves functional outcome postoperatively	WHO performance status<br>mini-MoCA (Montreal Version) Barthel Index<br>MRC grading of power in all 4 limbs	To be recorded at baseline, 5 days post-op., or discharge date (whichever is soonest); 6wks post-op., 3mths post-op., and then 3mthly up to 24 months.<br>(MRC grading to be assessed at baseline and 5 days post-op., or discharge date only)
**Secondary** Outcomes	Assess the correlation of proxy to participant classification assessment of quality of life	At a minimum, answers to questions 29 and 30 of the QLQ-C30. Ideally answers will be provided to all of the QLQ-C30 and BN20.	Baseline, 6wks post-op., 3mths post-op., and then 3mthly up to 24 months. Proxy will not complete questionnaires when participant stops completing them.
**Tertiary** Mechanistic Study Objectives – on a sub set of participants –	To assess the sensitivity and specificity of the anatomico-spatial location of DTI fibre tracts compared with intraoperative direct electrical stimulation/behavioural change without stimulation but related to adjacent white fibre tract in patients undergoing awake surgery, or motor evoked potential changes in patients undergoing surgery.	Sensitivity and specificity calculation using pre and post-surgery MRI images	Analysis will be undertaken post-surgery.
**Tertiary** Mechanistic Study Objectives – on a sub set of participants –	To assess the sensitivity and specificity of iUS* to identify the tumour boundary when compared with 5-ALA, navigated biopsies will be taken from tumour boundary tissue planned for resection.	Intra operative iUS* images and post-operative MRI scans and Intraoperative biopsy samples	Analysis will be undertaken post-surgery when biopsy results are available.

*if NiUS available, it is to be used

Table 1. Objectives and outcome measures for the trial.

End of trial

The end of the trial will be defined as the collection/receipt of the last follow-up questionnaire from the last participant and all data cleaning has been completed.

For peer review only

Statistical Methods

Full details of the statistical analysis will be detailed in a separate statistical analysis plan which will be drafted early in the trial and will be finalised after input from the TSC and DSMC. A summary of the planned statistical analysis is included here.

The analysis of the primary outcome will be a time-to-event analysis using a mixed effect Cox proportional hazard regression model. Minimisation factors (age, anticipated patient operative state and tumour location), radiotherapy, and MethylGuanine-DNA MethylTransferase (MGMT) status will be adjusted for as fixed effects. Centre will be included as a random effect.

The assumption of proportional hazard for the Cox model will be examined. If the proportional hazard assumption is not met, parametric survival analysis, such as the accelerated failure time method will be considered. A sensitivity analysis will look at the impact of adjusting for surgeon instead of centre. Secondary analysis will explore the influence of progression as an event by assessing DFS minus disease progression as an event. An unadjusted comparison using a log-rank test will also be carried out. Kaplan-Meier curves will also be generated. Secondary time-to-event outcomes (e.g. OS) will be analysed in a similar manner.

HRQoL amongst survivors will be quantified without a formal statistical comparison between treatment groups.

There are multiple factors that may influence how a patient rates their level of HRQoL, which may be related to factors other than the intervention. However, by using a randomized trial design, it is assumed that patients in both treatment arms are comparable on all aspects, both measured (e.g. age, performance status) and unmeasured (e.g. mood, coping strategy, personality). This means that the impact of the psychological state on the evaluation of HRQoL is treated as similar for the two trial arms. Thus, the trial will be able to measure whether the experimental intervention has an impact on HRQoL when compared to patients receiving standard treatment.

The trial will attempt to collect data as completely as possible. The main analysis will include participants for whom endpoint data are available, with other participants being censored after their last available relevant outcome measure. Sensitivity analyses will examine the effects of alternative assumptions about the missing data. Further details on will be provided in the the Statistical Analysis Plan, and the Data Monitoring Plan.

Sample Size Determination for IDEAL Framework IIB Trial (Stage-1)

There is no formal sample size for the IDEAL trial. Participants will be recruited at each centre, the number of cases required from each centre will vary depending upon caseload numbers and the number of neurosurgeons but is expected to be small for most sites (5), as the participating centres are already familiar with the component techniques.

Sample Size Determination for the RCT (Stage-2)

The sample size is based on a HRQoL aspect included in the primary outcome DFS, i.e. the global health status domain in the EORTC QLQ-C30 questionnaire version 3.0, and achieving

For peer review only

a statistical power of 90% for the primary analysis (see below) with 2-sided significance level of 5%. Assuming a Hazard Ratio (HR) of 0.7, median DFS survival time of 5 months in the control arm, 24 months follow-up on all participants and allowing for 5% loss to follow-up occurring by month 3, this yields an overall target of 357 participants (178/179 per arm; 335 events overall) (Stata "artsurv"; www.stata.com). In a recent trial, the mean survival time of global health status DFS was 6 months in the standard treatment arm (surgical resection with standard radiotherapy and chemotherapy)[32]. Additionally, the observed HR was 0.64, 95% CI (0.56, 0.74) for the DFS measures in this trial suggesting that a HR of 0.7 as assumed above is a plausible magnitude of effect to be observed for this population[33]. It would also be one which would be considered important to clinicians and patients given the definition of a DFS event (death, progression or a patient anchor determined clinically meaningful deterioration of 10 points). For key secondary outcomes (i.e. the other four DFS outcomes, PFS and OS) there is over 80% power for this size of trial, assuming a median OS of 6-9, 7 and 15months respectively in the control arm, a HR of 0.70 for both, and other inputs as per above.

Decision points

Stage-1 (IDEAL IIB trial)

The trial team will evaluate patient CRF and imaging data continuously on a case-by-case basis from each site and provide regular feedback and assessment. Any additional training/guidance is provided as needed. After a site has done an adequate number of cases and has objectively met the primary outcomes and workflow requirements, the completed data set will be revaluated by the trial team including the CI and Lead Investigators. A meeting between the trial team and site is then held to allow feedback from the site and discussion of lessons learned. This meeting is formally documented and if all the criteria are met, the site can then progress to Stage 2 (supplementary files).

Stage-2 (RCT)

Built into the trial is an internal pilot of recruitment to the RCT (Stage-2). There will be a formal stop/go review after 12months of recruitment to the RCT to review the number of randomisations over the pilot period – the stop-go criteria are listed in table 2. If the target of at least 80 randomisations has been met, the trial will continue to recruit for a further 15months. Data from the 80 patients will be included in the final analysis.

	**actual recruitment after 12 months of recruitment**		
**target = 80**	**>80 participants**	**65 - 80 participants**	**<65 participants**
recruitment rate (per centre per month)	0.6	0.45	0.37
stop-go criteria	recruitment feasible<br><br>proceed with trial	review recruitment strategies<br>report to TSC<br><br>Continue but modify and monitor closely	recruitment not feasible<br><br>decision not to proceed

Table 2. Proposed stop-go criteria for the TSC at 12 months.

Data Management

Data will be collected from participants and proxies via questionnaires and case report forms that will be returned to the central trial office in Oxford, via post using a pre-addressed freepost envelope, NHS email as appropriate, or directly into an online secure database (REDCap). In addition, participant images will be stored within the cloud database Quentry (BrainLab AG). As a third-party processor, BrainLab will not receive any data that could identify participants.

All trial-specific documents, except for the signed consent form and follow-up contact details, will refer to the participant with a unique trial participant number/code and not by name. The data will be stored and used in compliance with the relevant, current data protection laws (Data Protection Act 2018; General Data Protection Regulation (GDPR) 2018). The trial data (including data for SAEs) will be entered onto a validated REDCap trial database developed and maintained by OCTRU and which can only be accessed by authorised users via the application. After closure of the trial and data analyses, the data will be made publicly available at the time of publication. The Trial Master File will be archived for five years from the end of the trial.

Patient and public involvement (PPI)

The trial focuses on keeping good HRQoL for people living with a GB for as long as possible. It has been designed with the help of patient support groups at the Brain Tumour Charity and Brainstrust, the Patient Relative Advisory Group at the Oxford University Hospitals NHS Foundation Trust and the Brain Tumour PPI Group at Imperial College Healthcare NHS Trust. Dr Helen Bulbeck (Brainstrust's Director) has been part of the trial proposal and is one of the trial's investigators.

Trial oversight

The day-to-day management of the trial will be the responsibility of the clinical trial manager, based at Nuffield Department of Surgical Sciences and supported by the Oxford Clinical Trials Research Unit (OCTRU) and the Surgical Intervention Trials Unit (SITU) staff all based at the University of Oxford with the Chief Investigator. This will be overseen by the trial management group, who will meet monthly to assess progress.

A trial steering committee (TSC) and a DSMC will be set up. The DSMC will adopt a DAMOCLES based charter which defines its terms of reference and operation in relation to oversight of the trial. They will not be asked to perform any formal interim analyses of effectiveness. They will, however, see copies of data accrued to date and summaries of that data by treatment group. They will also consider emerging evidence from other related trials or research and review related SAEs that have been reported. They may advise the chair of the TSC at any time if, in their view, the trial should be stopped for ethical reasons, including concerns about participant safety. DSMC meetings will be held at least annually during the recruitment phase of the study.

Quality control

The study may be monitored, or audited in accordance with the current approved protocol, relevant regulations and standard operating procedures by the host organisation or sponsor. A monitoring plan will be developed according to OCTRU standard operating

procedures which involves a risk assessment. The monitoring activities are based on the outcome of the risk assessment and may involve central and site monitoring.

Ethics and Dissemination

The trial was registered in the Integrated Research Application System (Ref: 264482) and approved by a UK research and ethics committee (Ref: 20/LO/0840). Results will be published in a peer reviewed journal. Further dissemination to participants, patient groups and the wider medical community will utilize a range of approaches to maximize impact.

Author Contributions

PP, SC, DN were responsible for conceptualisation and design of the trial. RM is a trial clinician and drafted manuscript. JAC provided statistical input into the trial design and conduct. CW, MDJ, KA, SJP, VA are trial clinicians and provided input regarding neurosurgical expertise and trial design. MT, LD, MW provided input on neuro-oncology management. MT, LD (Linda Dirven) provided expert opinion on quality-of-life measures. AL, LD (Luke Dixon) developed section on intraoperative ultrasound techniques and analysis. PM was responsible for guidance on the IDEAL framework. NV, MGS developed section on DTI imaging and analysis. HB organised and provided PPI input. VSB provided input into the trial design. AT provided input to the trial design and is the trial manager. All authors provided critical appraisal of the protocol and manuscript.

Acknowledgments

We would like to thank our participants and other research team members (Amy Taylor, Nadjat Medehgri, Jack Morris, Lucy Eldridge, Ariel Wang and Tianshu Liu) involved in the day to day running of the trial. This trial will be conducted as part of the portfolio of trials in the UK Clinical Research Collaboration registered Clinical Trials Unit – the Oxford Clinical Trials Research Unit (OCTRU) and the Surgical Intervention Trial Unit (SITU) at the University of Oxford. It will follow their Standard Operating Procedures ensuring compliance with the principles of Good Clinical Practice and the Declaration of Helsinki and any applicable regulatory requirements.

Funding

This paper presents independent research funded by the National Institute for Health Research (NIHR) under its Efficacy and Mechanism Evaluation Programme (Grant Reference: NIHR 127930; https://fundingawards.nihr.ac.uk/award/NIHR127930). The views expressed are those of the author(s) and not necessarily those of the NIHR or the Department of Health and Social Care. The University of Oxford is the sponsor of the trial. The funders and the sponsor of the trial had no explicit role in the trial design.

Competing interests

The authors do not have any competing interests to disclose.

For peer review only

References

1 Brodbelt A, Greenberg D, Winters T, *et al.* Glioblastoma in England: 2007-2011. *Eur J Cancer* 2015;**51**:533–42. doi:10.1016/j.ejca.2014.12.014

2 Stupp R, Mason WP, van den Bent MJ, *et al.* Radiotherapy plus concomitant and adjuvant temozolomide for glioblastoma. *N Engl J Med* 2005;**352**:987–96. doi:10.1056/nejmoa043330

3 Overview | Brain tumours (primary) and brain metastases in adults | Guidance | NICE. https://www.nice.org.uk/guidance/ng99 (accessed 1 Sep 2021).

4 Creighton H, Beach B, Bamford S-M. Rethinking Cancer. 2015. www.ilcuk.org.uk (accessed 1 Sep 2021).

5 Burnet NG, Jefferies SJ, Benson RJ, *et al.* Years of life lost (YLL) from cancer is an important measure of population burden - And should be considered when allocating research funds. *Br J Cancer* 2005;**92**:241–5. doi:10.1038/sj.bjc.6602321

6 Cihoric N, Tsikkinis A, Minniti G, *et al.* Current status and perspectives of interventional clinical trials for glioblastoma - analysis of ClinicalTrials.gov. Radiat. Oncol. 2017;**12**:1. doi:10.1186/s13014-016-0740-5

7 Li YM, Suki D, Hess K, *et al.* The influence of maximum safe resection of glioblastoma on survival in 1229 patients: Can we do better than gross-total resection? *J Neurosurg* 2015;**124**:1–12. doi:10.3171/2015.5.JNS142087

8 Stummer W, Reulen HJ, Meinel T, *et al.* Extent of resection and survival in glioblastoma multiforme: identification of and adjustment for bias. *Neurosurgery* 2008;**62**:564–76. doi:00006123-200803000-00003 [pii] 10.1227/01.neu.0000317304.31579.17

9 Brown TJ, Brennan MC, Li M, *et al.* Association of the extent of resection with survival in glioblastoma a systematic review and meta-Analysis. *JAMA Oncol* 2016;**2**:1460–9. doi:10.1001/jamaoncol.2016.1373

10 Bal J, Camp SJ, Nandi D. The use of ultrasound in intracranial tumor surgery. Acta Neurochir. (Wien). 2016;**158**:1179–85. doi:10.1007/s00701-016-2803-7

11 Dixon L, Lim A, Grech-Sollars M, *et al.* Intraoperative Ultrasound in Brain Tumor Surgery: A review and implementation guide. *Neurosurg Rev* 2022.

12 Serra C, Stauffer A, Actor B, *et al.* Intraoperative high frequency ultrasound in intracerebral high-grade tumors. *Ultraschall der Medizin* 2012;**33**:E306–12. doi:10.1055/s-0032-1325369

13 Mahboob S, McPhillips R, Qiu Z, *et al.* Intraoperative Ultrasound-Guided Resection of Gliomas: A Meta-Analysis and Review of the Literature. *World Neurosurg* 2016;**92**:255–63. doi:10.1016/j.wneu.2016.05.007

14 Unsgaard G, Rygh OM, Selbekk T, *et al.* Intra-operative 3D ultrasound in neurosurgery. Acta Neurochir. (Wien). 2006;**148**:235–53. doi:10.1007/s00701-005-0688-y

15 Unsgaard G, Ommedal S, Muller T, *et al.* Neuronavigation by intraoperative three-dimensional ultrasound: Initial experience during brain tumor resection. *Neurosurgery* 2002;**50**:804–12. doi:10.1097/00006123-200204000-00022

16 Unsgaard G, Gronningsaeter A, Ommedal S, *et al.* Brain operations guided by real-time two-dimensional ultrasound: New possibilities as a result of improved image quality. *Neurosurgery* 2002;**51**:402–12. doi:10.1097/00006123-200208000-00019

17 Sæther CA, Torsteinsen M, Torp SH, *et al.* Did survival improve after the

implementation of intraoperative neuronavigation and 3d ultrasound in glioblastoma surgery? A retrospective analysis of 192 primary operations. *J Neurol Surgery, Part A Cent Eur Neurosurg* 2012;**73**:73–8. doi:10.1055/s-0031-1297247

18 Camp SJ, Apostolopoulos V, Raptopoulos V, *et al.* Objective image analysis of real-time three-dimensional intraoperative ultrasound for intrinsic brain tumour surgery. *J Ther Ultrasound* 2017;**5**:2. doi:10.1186/s40349-017-0084-0

19 Woydt M, Krone A, Becker G, *et al.* Correlation of Intra-Operative Ultrasound with Histopathologic Findings after Tumour Resection in Supratentorial Gliomas: A Method to Improve Gross Total Tumour Resection. *Acta Neurochir (Wien)* 1996;**138**:1391–8. doi:10.1007/BF01411117

20 Moiyadi A, Shetty P. Navigable Intraoperative Ultrasound and Fluorescence-Guided Resections Are Complementary in Resection Control of Malignant Gliomas: One Size Does Not Fit All. *J Neurol Surgery, Part A Cent Eur Neurosurg* 2014;**75**:434–41. doi:10.1055/s-0034-1372436

21 Voets NL, Bartsch A, Plaha P. Brain white matter fibre tracts: A review of functional neuro-oncological relevance. J. Neurol. Neurosurg. Psychiatry. 2017;**88**:1017–25. doi:10.1136/jnnp-2017-316170

22 Bello L, Riva M, Fava E, *et al.* Tailoring neurophysiological strategies with clinical context enhances resection and safety and expands indications in gliomas involving motor pathways. *Neuro Oncol* 2014;**16**:1110–28. doi:10.1093/neuonc/not327

23 Vassal F, Schneider F, Sontheimer A, *et al.* Intraoperative visualisation of language fascicles by diffusion tensor imaging-based tractography in glioma surgery. *Acta Neurochir (Wien)* 2013;**155**:437–48. doi:10.1007/s00701-012-1580-1

24 Herbet G, Maheu M, Costi E, *et al.* Mapping neuroplastic potential in brain-damaged patients. *Brain* 2016;**139**:829–44. doi:10.1093/brain/awv394

25 Winston GP, Daga P, White MJ, *et al.* Preventing visual field deficits from neurosurgery. *Neurology* 2014;**83**:604–11. doi:10.1212/WNL.0000000000000685

26 Caverzasi E, Hervey-Jumper SL, Jordan KM, *et al.* Identifying preoperative language tracts and predicting postoperative functional recovery using HARDI q-ball fiber tractography in patients with gliomas. *J Neurosurg* 2016;**125**:33–45. doi:10.3171/2015.6.JNS142203

27 Wu JS, Zhou LF, Tang WJ, *et al.* Clinical evaluation and follow-up outcome of diffusion tensor imaging-based functional neuronavigation: A prospective, controlled study in patients with gliomas involving pyramidal tracts. *Neurosurgery* 2007;**61**:935–48. doi:10.1227/01.neu.0000303189.80049.ab

28 Ma R, Livermore LJ, Plaha P. Fast Track Recovery Program After Endoscopic and Awake Intraparenchymal Brain Tumor Surgery. *World Neurosurg* 2016;**93**:246–52. doi:10.1016/j.wneu.2016.06.015

29 Jenkinson MD, Barone DG, Bryant A, *et al.* Intraoperative imaging technology to maximise extent of resection for glioma. Cochrane Database Syst. Rev. 2018;**2018**. doi:10.1002/14651858.CD012788.pub2

30 McCulloch P, Cook JA, Altman DG, *et al.* IDEAL framework for surgical innovation 1: the idea and development stages. *BMJ* 2013;**346**:f3012. doi:10.1136/bmj.f3012

31 McCulloch P, Altman DG, Campbell WB, *et al.* No surgical innovation without evaluation: the IDEAL recommendations. Lancet. 2009;**374**:1105–12. doi:10.1016/S0140-6736(09)61116-8

32 Taphoorn MJB, Henriksson R, Bottomley A, *et al.* Health-related quality of life in a

For peer review only

randomized phase III study of bevacizumab, temozolomide, and radiotherapy in newly diagnosed glioblastoma. *J Clin Oncol* 2015;**33**:2166–75. doi:10.1200/JCO.2014.60.3217
33 Cook JA, Julious SA, Sones W, *et al.* DELTA 2 guidance on choosing the target difference and undertaking and reporting the sample size calculation for a randomised controlled trial. *BMJ* 2018;**363**. doi:10.1136/bmj.k3750

For peer review only

Figure 1. Trial Flowchart

For peer review only

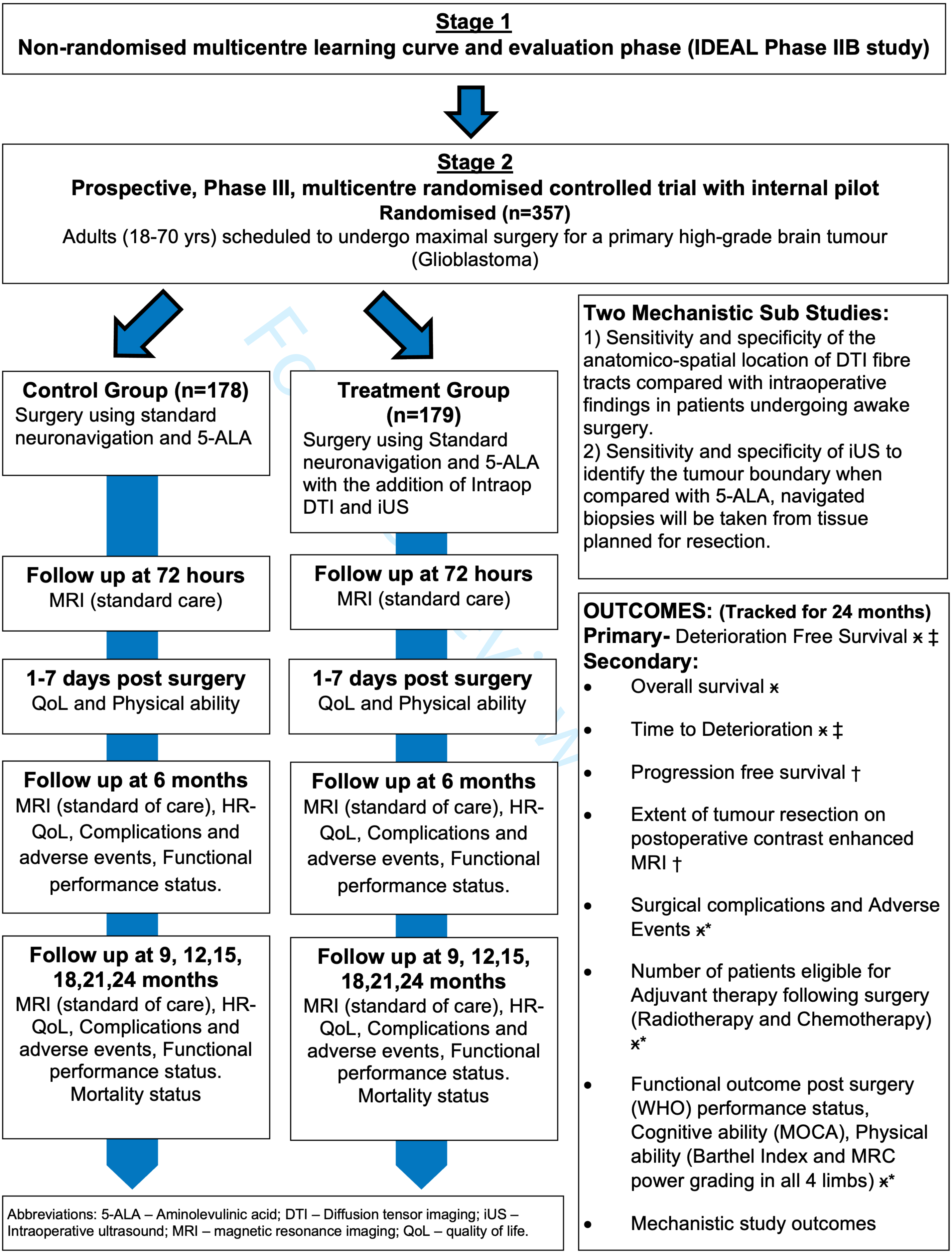
Stage 1
Non-randomised multicentre learning curve and evaluation phase (IDEAL Phase IIB study)
Stage 2
Prospective, Phase III, multicentre randomised controlled trial with internal pilot
Randomised (n=357)
Adults (18-70 yrs) scheduled to undergo maximal surgery for a primary high-grade brain tumour (Glioblastoma)
Control Group (n=178)
Surgery using standard neuronavigation and 5-ALA
Treatment Group (n=179)
Surgery using Standard neuronavigation and 5-ALA with the addition of Intraop DTI and iUS
Follow up at 72 hours
MRI (standard care)
Follow up at 72 hours
MRI (standard care)
1-7 days post surgery
QoL and Physical ability
1-7 days post surgery
QoL and Physical ability
Follow up at 6 months
MRI (standard of care), HR-QoL, Complications and adverse events, Functional performance status.
Follow up at 6 months
MRI (standard of care), HR-QoL, Complications and adverse events, Functional performance status.
Follow up at 9, 12,15, 18,21,24 months
MRI (standard of care), HR-QoL, Complications and adverse events, Functional performance status.
Mortality status
Follow up at 9, 12,15, 18,21,24 months
MRI (standard of care), HR-QoL, Complications and adverse events, Functional performance status.
Mortality status
Two Mechanistic Sub Studies:
1) Sensitivity and specificity of the anatomico-spatial location of DTI fibre tracts compared with intraoperative findings in patients undergoing awake surgery.
2) Sensitivity and specificity of iUS to identify the tumour boundary when compared with 5-ALA, navigated biopsies will be taken from tissue planned for resection.
OUTCOMES: (Tracked for 24 months)
Primary- Deterioration Free Survival ⁎ ‡
Secondary:
• Overall survival ⁎
• Time to Deterioration ⁎ ‡
• Progression free survival †
• Extent of tumour resection on postoperative contrast enhanced MRI †
• Surgical complications and Adverse Events ⁎*
• Number of patients eligible for Adjuvant therapy following surgery (Radiotherapy and Chemotherapy) ⁎*
• Functional outcome post surgery (WHO) performance status, Cognitive ability (MOCA), Physical ability (Barthel Index and MRC power grading in all 4 limbs) ⁎*
• Mechanistic study outcomes
Abbreviations: 5-ALA – Aminolevulinic acid; DTI – Diffusion tensor imaging; iUS – Intraoperative ultrasound; MRI – magnetic resonance imaging; QoL – quality of life.

FUTURE-GB STAGE 1 SITE DATA COMPLETION ASSESSMENT

**Reviewers:**	*Intra operative workflow & DTI:*	Prof Natalie Voets, Puneet Plaha, Miss Joy Roach, Amy Taylor
	Intra operative workflow & US:	Dipankar Nandi, Sophie Camp, Luke Dixon, Amy Taylor
	REDCap data entry & workflow:	Amy Taylor, Jack Morris, Puneet Plaha

**Site name:**		**Total Patients recruited:**		**Total Patients screened:**	

**Study ID**					
**Date of review**					
**Date of surgery**					
**Awake or GA surgery**					
**Pre -op tumour planning on MRI scans**					
**Pre-op tumour volume (cm^3)**					
**Post-op tumour volume (cm^3)**					
**Comments:**					
**MRI DTI and USG Acquisition**					
DTI – Site engaged with trials unit regarding DTI acquisition protocols ?	Yes/No	Yes/No	Yes/No	Yes/No	Yes/No
DTI scan acquired for surgery?	Yes/No	Yes/No	Yes/No	Yes/No	Yes/No
DTI Tracts reconstructed ?	Yes/No	Yes/No	Yes/No	Yes/No	Yes/No
USG used during surgery?	Yes/No	Yes/No	Yes/No	Yes/No	Yes/No
**Redcap data entry complete?**	Yes/No	Yes/No	Yes/No	Yes/No	Yes/No
Any difficulty entering data on REDCap ?	Yes/No	Yes/No	Yes/No	Yes/No	Yes/No
**Intraoperative workflow & Quentry imaging data transfer complete**	Yes/No	Yes/No	Yes/No	Yes/No	Yes/No
Data anonymised	Yes/No	Yes/No	Yes/No	Yes/No	Yes/No
Pre-op MRI scan performed	Yes/No	Yes/No	Yes/No	Yes/No	Yes/No
Pre -op MRI scan transferred					
Any Intra-op Screenshots acquired for awake surgery	Yes/No/ N/A	Yes/No/ N/A	Yes/No/ N/A	Yes/No/ N/A	Yes/No/ N/A
Intra-op Screenshots acquired for GA neurophysiology	Yes/No/ N/A	Yes/No/ N/A	Yes/No/ N/A	Yes/No/ N/A	Yes/No/ N/A
USG pre-resection – pictures/videos	Yes/No	Yes/No	Yes/No	Yes/No	Yes/No
USG post-resection- pictures/videos	Yes/No	Yes/No	Yes/No	Yes/No	Yes/No
Post-op MRI scan performed	Yes/No	Yes/No	Yes/No	Yes/No	Yes/No

FUTURE-GB_S1_Template_SiteDataCompletion_V3.0_18Oct2021.docx

FUTURE-GB STAGE 1 SITE DATA COMPLETION ASSESSMENT

**Post op MRI scan transferred**	Yes/No	Yes/No	Yes/No	Yes/No	Yes/No
**Difficulties in transferring Data to Quentry**	Yes/No	Yes/No	Yes/No	Yes/No	Yes/No
**Site PI - DTI comments**					
**Reviewer - DTI comments**					
**Reviewer - US comments**					
**Site PI - US comments**					
**Primary outcomes for Stage 1 complete**	Yes/No	Yes/No	Yes/No	Yes/No	Yes/No
Operation length					
Successful use of DTI neuronavigation and iUS to achieve complete tumour resection without major neurological deficit *(success defined here as appropriate and competent use of the imaging technologies to achieve the projected surgical outcome for each patient)*					
Extent of tumour resection assessed on postoperative MRI scan					
Surgical Complication and Serious Adverse Events (if applicable)	Yes/No	Yes/No	Yes/No	Yes/No	Yes/No
**Reviewer Comments:**					
**Analysis of imaging data to answer tertiary end point of the RCT study**					
Utility of imaging data to answer the DTI tertiary endpoint					
Utility of imaging data to answer the US tertiary endpoint					
**Reviewer Comments**					

**Overall reviewer comments**	

SITE FEEDBACK LOG

Date of meeting/discussion with site during Stage 1	Type of meeting	Feedback comments

FUTURE-GB_S1_Template_SiteDataCompletion_V3.0_18Oct2021.docx

FUTURE-GB STAGE 2 PROGRESSION AGREEMENT

The Co-Chief investigators of the FUTURE-GB trial agree that the trial site:

<site name and NHS Trust>

has met the following provisions in Stage 1 of the FUTURE-GB trial and recruited <number> participants

Trial team review was conducted on DDMonYYYY and it was agreed on this date (DDMonYYYY) that this site can proceed to Stage 2.

Co-Chief investigators report:

This should include:

1. *Objective endpoints of Stage 1*
2. *Quality of DT and US imaging data*
3. *Any difficulties regarding data-workflow from site*
4. *Any suggestions for improvement*

Note: Completion of this agreement by all signatories will result in the Stage 1 Registration System and screening system being closed by the Trial Manager on or after this date, and requesting that the site is opened to recruitment in the Stage 2 Screening, Randomisation and Database System. The Stage 1 Database system will not be closed to the site until all outstanding data has been entered and cleaned/queried as required by the Trial Statistician.

Document title	FUTURE-GB_Stage2Progression_V1.0_11Jan2020.docx				Page 1 of 2
Chief Investigators: Puneet Plaha, Sophie Camp, Dipankar Nandi		IRAS No.	264482	REC ref.:	

FUTURE-GB STAGE 2 PROGRESSION AGREEMENT

Name	Signature	Date
Professor Puneet Plaha		
Professor Dipankar Nandi		
Miss Sophie Camp		

Document title	FUTURE-GB_Stage2Progression_V1.0_11Jan2020.docx				Page 2 of 2
Chief Investigators: Puneet Plaha, Sophie Camp, Dipankar Nandi		IRAS No.	264482	REC ref.:	

PARTICIPANT ID: FG-XX-XXXX

PARTICIPANT QUESTIONNAIRE

Thank you for agreeing to participate in the FUTURE-GB study.

This is the baseline questionnaire.

We would be grateful if you could complete this questionnaire on how you are feeling and if you are having any issues due to your glioblastoma.

We will ask you to complete this same questionnaire again before you leave hospital, then at 6 weeks after your entered the study, 3 months after you entered the study and every 3 months thereafter up until 24 months.

If you have any questions about the study or this questionnaire, please do not hesitate to contact a member of the study team on future-gb@nds.ox.ac.uk or call 01865 xxxxxx (Monday to Friday, 9-4pm), there is an answering machine for messages outside of these times, or contact your local clinical team)

For peer review only

For peer review only - http://bmjopen.bmj.com/site/about/guidelines.xhtml

PARTICIPANT ID: FG-XX-XXXX

We are interested in some things about you and your health.

Please answer all of the questions yourself by circling the number that best applies to you. There are no “right” or “wrong” answers. The information that you provide will remain strictly confidential.

We appreciated that you may be very busy and this may be a distressing time, therefore if you are unable to complete this questionnaire either due to time or other reasons – if at all possible however we would be very grateful if could complete the date below and questions 29 and 30 on page 4.

What is today’s date: DD/MM/YYYY

For peer review only - http://bmjopen.bmj.com/site/about/guidelines.xhtml

PARTICIPANT ID: FG-XX-XXXX

		Not at All	A Little	Quite a Bit	Very Much
1.	Does you have any trouble doing strenuous activities, like carrying a heavy shopping bag or a suitcase?	1	2	3	4
2.	Do you have any trouble taking a long walk?	1	2	3	4
3.	Do you have any trouble taking a short walk outside of the house?	1	2	3	4
4.	Do you need to stay in bed or a chair during the day?	1	2	3	4
5.	Do you need help with eating, dressing, washing yourself or using the toilet?	1	2	3	4

During the past week:

		Not at All	A Little	Quite a Bit	Very Much
6.	Were you limited in doing either your work or other daily activities?	1	2	3	4
7.	Were you limited in pursuing their hobbies or other leisure time activities?	1	2	3	4
8.	Were you short of breath?	1	2	3	4
9.	Have you had pain?	1	2	3	4
10.	Did you need to rest?	1	2	3	4
11.	Have you had trouble sleeping?	1	2	3	4
12.	Have you felt weak?	1	2	3	4
13.	Have you lacked their appetite?	1	2	3	4
14.	Have you felt nauseated?	1	2	3	4
15.	Have you vomited?	1	2	3	4
16.	Have you been constipated?	1	2	3	4
17.	Have you had diarrhoea?	1	2	3	4
18.	Were you tired?	1	2	3	4
19.	Did pain interfere with your daily activities?	1	2	3	4
20.	Have you had difficulty concentrating on things, like reading a newspaper or watching television?	1	2	3	4
21.	Did you feel tense?	1	2	3	4
22.	Did you worry?	1	2	3	4
23.	Did you feel irritable?	1	2	3	4
24.	Did you feel depressed?	1	2	3	4
25.	Have you had difficulty remembering things?	1	2	3	4
26.	Has your physical condition or medical treatment interfered with your family life?	1	2	3	4
27.	Has your physical condition or medical treatment interfered with your social activities?	1	2	3	4

For peer review only - http://bmjopen.bmj.com/site/about/guidelines.xhtml

For peer review only

PARTICIPANT ID: FG-XX-XXXX

		Not at All	A Little	Quite a Bit	Very Much
28.	Has your physical condition or medical treatment caused you financial difficulties?	1	2	3	4

For the following questions please circle the number between 1 and 7 that best applies to you

29. How would you rate your overall <u>health</u> during the past week?

1 2 3 4 5 6 7

Very poor Excellent

30. How would you rate your overall <u>quality of life</u> during the past week?

1 2 3 4 5 6 7

Very poor Excellent

For peer review only

Patients sometimes report that they have the following symptoms. Please indicate the extent to which you have experienced the below symptoms or problems during the past week.

During the past week:

		**Not at All**	**A Little**	**Quite a Bit**	**Very Much**
31.	Did you feel uncertain about the future?	1	2	3	4
32.	Did you feel you had setbacks in your condition?	1	2	3	4
33.	Were you concerned about disruption of family life?	1	2	3	4
34.	Did you have headaches?	1	2	3	4
35.	Did your outlook on the future worsen?	1	2	3	4
36.	Did you have double vision?	1	2	3	4

For peer review only - http://bmjopen.bmj.com/site/about/guidelines.xhtml

PARTICIPANT ID: FG-XX-XXXX

		Not at All	A Little	Quite a Bit	Very Much
37.	Was your vision blurred?	1	2	3	4
38.	Did you have difficulty reading because of your vision?	1	2	3	4
39.	Did you have seizures?	1	2	3	4
40.	Did you have weakness on one side of your body?	1	2	3	4
41.	Did you have trouble finding the right words to express yourself?	1	2	3	4
42.	Did you have difficulty speaking?	1	2	3	4
43.	Did you trouble communicating your thoughts?	1	2	3	4
44.	Did you feel drowsy during the daytime?	1	2	3	4
45.	Did you have trouble with your coordination?	1	2	3	4
46.	Did hair loss bother you?				
47.	Did itching of their skin bother you?	1	2	3	4
48.	Did you have weakness of both legs?	1	2	3	4
49.	Did you feel unsteady on your feet?	1	2	3	4
50.	Did you have trouble controlling your bladder?	1	2	3	4

For peer review only - http://bmjopen.bmj.com/site/about/guidelines.xhtml

PARTICIPANT ID: FG-XX-XXXX

SITE ADMINISTERED QUESTIONNAIRE

Please tick which time point this questionnaire relates to:

Baseline	☐	12 months	☐
Hospital Discharge	☐	15 months	☐
6 weeks	☐	18 months	☐
3 months	☐	21 months	☐
6 months	☐	24 months	☐
9 months	☐		

Date of completion: DD/MM/YYYY

Completed by: (PRINT NAME)…………………………………………

This document contains the following validated questionnaires:

- Barthel Index
- MOCA Assessment
- MRC Muscle Power Scale
- WHO Performance Status

For peer review only - http://bmjopen.bmj.com/site/about/guidelines.xhtml

PARTICIPANT ID: FG-XX-XXXX

BARTHEL INDEX

Activity	Score
**FEEDING**<br>0 = unable<br>5 = needs help cutting, spreading butter, etc., or requires modified diet<br>10 = independent	______
**BATHING**<br>0 = dependent<br>5 = independent (or in shower)	______
**GROOMING**<br>0 = needs to help with personal care<br>5 = independent face/hair/teeth/shaving (implements provided)	______
**DRESSING**<br>0 = dependent<br>5 = needs help but can do about half unaided<br>10 = independent (including buttons, zips, laces, etc.)	______
**BOWELS**<br>0 = incontinent (or needs to be given enemas)<br>5 = occasional accident<br>10 = continent	______
**BLADDER**<br>0 = incontinent, or catheterized and unable to manage alone<br>5 = occasional accident<br>10 = continent	______
**TOILET USE**<br>0 = dependent<br>5 = needs some help, but can do something alone<br>10 = independent (on and off, dressing, wiping)	______
**TRANSFERS (BED TO CHAIR AND BACK)**<br>0 = unable, no sitting balance<br>5 = major help (one or two people, physical), can sit<br>10 = minor help (verbal or physical)<br>15 = independent	______
**MOBILITY (ON LEVEL SURFACES)**<br>0 = immobile or < 50 yards<br>5 = wheelchair independent, including corners, > 50 yards<br>10 = walks with help of one person (verbal or physical) > 50 yards<br>15 = independent (but may use any aid; for example, stick) > 50 yards	______
**STAIRS**<br>0 = unable<br>5 = needs help (verbal, physical, carrying aid)<br>10 = independent	______
**TOTAL (0–100):**	______

For peer review only - http://bmjopen.bmj.com/site/about/guidelines.xhtml

PARTICIPANT ID: FG-XX-XXXX

MOCA ASSESSMENT

VISUOSPATIAL / EXECUTIVE			POINTS
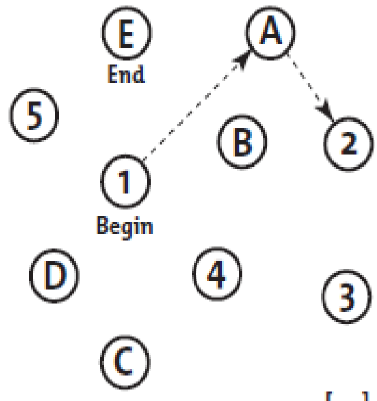 [ ]	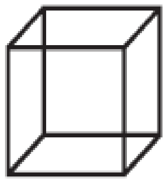 Copy cube [ ]	Draw CLOCK (Ten past eleven) (3 points) [ ] Contour [ ] Numbers [ ] Hands	___/5

NAMING	POINTS
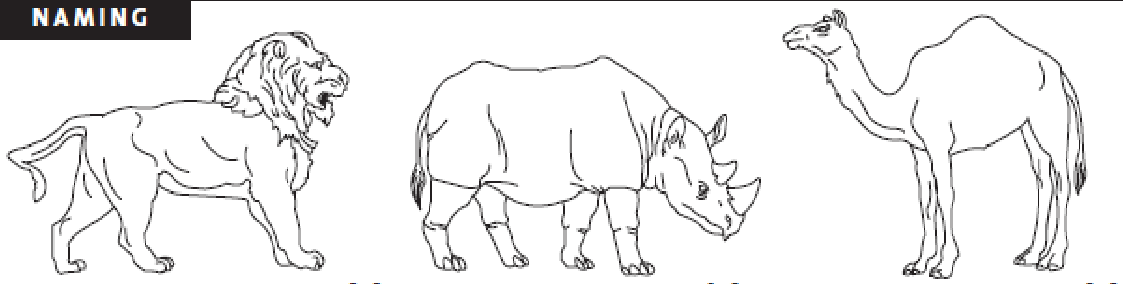 [ ] [ ] [ ]	___/3

MEMORY		FACE	VELVET	CHURCH	DAISY	RED	
Read list of words, subject must repeat them. Do 2 trials. Do a recall after 5 minutes.	1st trial						No points
	2nd trial						

ATTENTION		
Read list of digits (1 digit/ sec.).	Subject has to repeat them in the forward order [ ] 2 1 8 5 4<br>Subject has to repeat them in the backward order [ ] 7 4 2	___/2
Read list of letters. The subject must tap with his hand at each letter A. No points if ≥ 2 errors	[ ] FBACMNAAJKLBAFAKDEAAAJAMOFAAB	___/1
Serial 7 subtraction starting at 100	[ ] 93 [ ] 86 [ ] 79 [ ] 72 [ ] 65<br>4 or 5 correct subtractions: **3 pts**, 2 or 3 correct: **2 pts**, 1 correct: **1 pt**, 0 correct: **0 pt**	___/3

LANGUAGE		
Repeat :	I only know that John is the one to help today. [ ]<br>The cat always hid under the couch when dogs were in the room. [ ]	___/2
Fluency / Name maximum number of words in one minute that begin with the letter F	[ ] ____ (N ≥ 11 words)	___/1

ABSTRACTION		
Similarity between e.g. banana - orange = fruit	[ ] train – bicycle [ ] watch - ruler	___/2

DELAYED RECALL	Has to recall words	FACE	VELVET	CHURCH	DAISY	RED		
	WITH NO CUE	[ ]	[ ]	[ ]	[ ]	[ ]	Points for UNCUED recall only	___/5
Optional	Category cue							
	Multiple choice cue							

ORIENTATION		
[ ] Date [ ] Month [ ] Year [ ] Day [ ] Place [ ] City		___/6

© Z.Nasreddine MD Version November 7, 2004
www.mocatest.org

Normal ≥ 26 / 30 TOTAL ___/30
Add 1 point if ≤ 12 yr edu

For peer review only - http://bmjopen.bmj.com/site/about/guidelines.xhtml

PARTICIPANT ID: FG-XX-XXXX

MRC MUSCLE POWER SCALE

Score	Description
**0**	No contraction
**1**	Flicker or trace of contraction
**2**	Active movement, with gravity eliminated
**3**	Active movement against gravity
**4**	Active movement against gravity and resistance
**5**	Normal power

Please grade the 4 limbs of the patient

Left arm: ☐

Right arm: ☐

Left leg: ☐

Right leg: ☐

WHO PERFORMANCE STATUS

The WHO performance status classification categorises patients as:

- 0: able to carry out all normal activity without restriction
- 1: restricted in strenuous activity but ambulatory and able to carry out light work
- 2: ambulatory and capable of all self-care but unable to carry out any work activities; up and about more than 50% of waking hours
- 3: symptomatic and in a chair or in bed for greater than 50% of the day but not bedridden
- 4: completely disabled; cannot carry out any self-care; totally confined to bed or chair.

Please circle the WHO Performance status for the patient as at today:

0 1 2 3 4

For peer review only - http://bmjopen.bmj.com/site/about/guidelines.xhtml

PARTICIPANT ID: FG-XX-XXXX

PARTICIPANT QUESTIONNAIRE

Thank you for agreeing to participate in the FUTURE-GB study.

Please tick which time point this questionnaire relates to:

- Hospital Discharge ☐
- 6 weeks ☐
- 3 months ☐
- 6 months ☐
- 9 months ☐
- 12 months ☐
- 15 months ☐
- 18 months ☐
- 21 months ☐
- 24 months ☐

For peer review only

We would be grateful if you could complete this questionnaire on how you are feeling and if you are having any issues due to your glioblastoma.

If you have any questions about the study or this questionnaire, please do not hesitate to contact a member of the study team on future-gb@nds.ox.ac.uk or call 01865 xxxxxx (Monday to Friday, 9-4pm), there is an answering machine for messages outside of these times, or contact your local clinical team)

For peer review only - http://bmjopen.bmj.com/site/about/guidelines.xhtml

PARTICIPANT ID: FG-XX-XXXX

We are interested in some things about you and your health.

Please answer all of the questions yourself by circling the number that best applies to you. There are no “right” or “wrong” answers. The information that you provide will remain strictly confidential.

For peer review only

We appreciated that you may be very busy and this may be a distressing time, therefore if you are unable to complete this questionnaire either due to time or other reasons – if at all possible however we would be very grateful if could complete the date below and questions 29 and 30 on page 4.

What is today’s date: DD/MM/YYYY

For peer review only - http://bmjopen.bmj.com/site/about/guidelines.xhtml

PARTICIPANT ID: FG-XX-XXXX

		Not at All	A Little	Quite a Bit	Very Much
1.	Does you have any trouble doing strenuous activities, like carrying a heavy shopping bag or a suitcase?	1	2	3	4
2.	Do you have any trouble taking a long walk?	1	2	3	4
3.	Do you have any trouble taking a short walk outside of the house?	1	2	3	4
4.	Do you need to stay in bed or a chair during the day?	1	2	3	4
5.	Do you need help with eating, dressing, washing yourself or using the toilet?	1	2	3	4

During the past week:

		Not at All	A Little	Quite a Bit	Very Much
6.	Were you limited in doing either your work or other daily activities?	1	2	3	4
7.	Were you limited in pursuing their hobbies or other leisure time activities?	1	2	3	4
8.	Were you short of breath?	1	2	3	4
9.	Have you had pain?	1	2	3	4
10.	Did you need to rest?	1	2	3	4
11.	Have you had trouble sleeping?	1	2	3	4
12.	Have you felt weak?	1	2	3	4
13.	Have you lacked their appetite?	1	2	3	4
14.	Have you felt nauseated?	1	2	3	4
15.	Have you vomited?	1	2	3	4
16.	Have you been constipated?	1	2	3	4
17.	Have you had diarrhoea?	1	2	3	4
18.	Were you tired?	1	2	3	4
19.	Did pain interfere with your daily activities?	1	2	3	4
20.	Have you had difficulty concentrating on things, like reading a newspaper or watching television?	1	2	3	4
21.	Did you feel tense?	1	2	3	4
22.	Did you worry?	1	2	3	4
23.	Did you feel irritable?	1	2	3	4
24.	Did you feel depressed?	1	2	3	4
25.	Have you had difficulty remembering things?	1	2	3	4
26.	Has your physical condition or medical treatment interfered with your family life?	1	2	3	4
27.	Has your physical condition or medical treatment interfered with your social activities?	1	2	3	4

For peer review only

For peer review only - http://bmjopen.bmj.com/site/about/guidelines.xhtml

PARTICIPANT ID: FG-XX-XXXX

		Not at All	A Little	Quite a Bit	Very Much
28.	Has your physical condition or medical treatment caused you financial difficulties?	1	2	3	4

For the following questions please circle the number between 1 and 7 that best applies to you

29. How would you rate your overall <u>health</u> during the past week?

1 2 3 4 5 6 7

Very poor Excellent

30. How would you rate your overall <u>quality of life</u> during the past week?

1 2 3 4 5 6 7

Very poor Excellent

For peer review only

Patients sometimes report that they have the following symptoms. Please indicate the extent to which you have experienced the below symptoms or problems during the past week.

During the past week:

		**Not at All**	**A Little**	**Quite a Bit**	**Very Much**
31.	Did you feel uncertain about the future?	1	2	3	4
32.	Did you feel you had setbacks in your condition?	1	2	3	4
33.	Were you concerned about disruption of family life?	1	2	3	4
34.	Did you have headaches?	1	2	3	4
35.	Did your outlook on the future worsen?	1	2	3	4
36.	Did you have double vision?	1	2	3	4

For peer review only - http://bmjopen.bmj.com/site/about/guidelines.xhtml

PARTICIPANT ID: FG-XX-XXXX

		Not at All	A Little	Quite a Bit	Very Much
37.	Was your vision blurred?	1	2	3	4
38.	Did you have difficulty reading because of your vision?	1	2	3	4
39.	Did you have seizures?	1	2	3	4
40.	Did you have weakness on one side of your body?	1	2	3	4
41.	Did you have trouble finding the right words to express yourself?	1	2	3	4
42.	Did you have difficulty speaking?	1	2	3	4
43.	Did you trouble communicating your thoughts?	1	2	3	4
44.	Did you feel drowsy during the daytime?	1	2	3	4
45.	Did you have trouble with your coordination?	1	2	3	4
46.	Did hair loss bother you?				
47.	Did itching of their skin bother you?	1	2	3	4
48.	Did you have weakness of both legs?	1	2	3	4
49.	Did you feel unsteady on your feet?	1	2	3	4
50.	Did you have trouble controlling your bladder?	1	2	3	4

For peer review only

For peer review only - http://bmjopen.bmj.com/site/about/guidelines.xhtml

PARTICIPANT ID: FG-XX-XXXX

PARTICIPANT QUESTIONNAIRE

Thank you for agreeing to participate in the FUTURE-GB study.

This is the hospital discharge questionnaire.

We would be grateful if you could complete this questionnaire on how you are feeling and if you are having any issues due to your glioblastoma.

This is the same questionnaire as the one you have completed previously.

If you have any questions about the study or this questionnaire, please do not hesitate to contact a member of the study team on future-gb@nds.ox.ac.uk or call 01865 xxxxxx (Monday to Friday, 9-4pm), there is an answering machine for messages outside of these times, or contact your local clinical team)

For peer review only

For peer review only - http://bmjopen.bmj.com/site/about/guidelines.xhtml

PARTICIPANT ID: FG-XX-XXXX

We are interested in some things about you and your health.

Please answer all of the questions yourself by circling the number that best applies to you. There are no “right” or “wrong” answers. The information that you provide will remain strictly confidential.

We appreciated that you may be very busy and this may be a distressing time, therefore if you are unable to complete this questionnaire either due to time or other reasons – if at all possible however we would be very grateful if could complete the date below and questions 29 and 30 on page 4.

What is today’s date: DD/MM/YYYY

For peer review only - http://bmjopen.bmj.com/site/about/guidelines.xhtml

PARTICIPANT ID: FG-XX-XXXX

		Not at All	A Little	Quite a Bit	Very Much
1.	Does you have any trouble doing strenuous activities, like carrying a heavy shopping bag or a suitcase?	1	2	3	4
2.	Do you have any trouble taking a long walk?	1	2	3	4
3.	Do you have any trouble taking a short walk outside of the house?	1	2	3	4
4.	Do you need to stay in bed or a chair during the day?	1	2	3	4
5.	Do you need help with eating, dressing, washing yourself or using the toilet?	1	2	3	4

During the past week:

		Not at All	A Little	Quite a Bit	Very Much
6.	Were you limited in doing either your work or other daily activities?	1	2	3	4
7.	Were you limited in pursuing their hobbies or other leisure time activities?	1	2	3	4
8.	Were you short of breath?	1	2	3	4
9.	Have you had pain?	1	2	3	4
10.	Did you need to rest?	1	2	3	4
11.	Have you had trouble sleeping?	1	2	3	4
12.	Have you felt weak?	1	2	3	4
13.	Have you lacked their appetite?	1	2	3	4
14.	Have you felt nauseated?	1	2	3	4
15.	Have you vomited?	1	2	3	4
16.	Have you been constipated?	1	2	3	4
17.	Have you had diarrhoea?	1	2	3	4
18.	Were you tired?	1	2	3	4
19.	Did pain interfere with your daily activities?	1	2	3	4
20.	Have you had difficulty concentrating on things, like reading a newspaper or watching television?	1	2	3	4
21.	Did you feel tense?	1	2	3	4
22.	Did you worry?	1	2	3	4
23.	Did you feel irritable?	1	2	3	4
24.	Did you feel depressed?	1	2	3	4
25.	Have you had difficulty remembering things?	1	2	3	4
26.	Has your physical condition or medical treatment interfered with your family life?	1	2	3	4
27.	Has your physical condition or medical treatment interfered with your social activities?	1	2	3	4

For peer review only - http://bmjopen.bmj.com/site/about/guidelines.xhtml

For peer review only

PARTICIPANT ID: FG-XX-XXXX

		Not at All	A Little	Quite a Bit	Very Much
28.	Has your physical condition or medical treatment caused you financial difficulties?	1	2	3	4

For the following questions please circle the number between 1 and 7 that best applies to you

29. How would you rate your overall <u>health</u> during the past week?

1 2 3 4 5 6 7

Very poor Excellent

30. How would you rate your overall <u>quality of life</u> during the past week?

1 2 3 4 5 6 7

Very poor Excellent

Patients sometimes report that they have the following symptoms. Please indicate the extent to which you have experienced the below symptoms or problems during the past week.

During the past week:

		**Not at All**	**A Little**	**Quite a Bit**	**Very Much**
31.	Did you feel uncertain about the future?	1	2	3	4
32.	Did you feel you had setbacks in your condition?	1	2	3	4
33.	Were you concerned about disruption of family life?	1	2	3	4
34.	Did you have headaches?	1	2	3	4
35.	Did your outlook on the future worsen?	1	2	3	4
36.	Did you have double vision?	1	2	3	4

For peer review only - http://bmjopen.bmj.com/site/about/guidelines.xhtml
For peer review only

PARTICIPANT ID: FG-XX-XXXX

		Not at All	A Little	Quite a Bit	Very Much
37.	Was your vision blurred?	1	2	3	4
38.	Did you have difficulty reading because of your vision?	1	2	3	4
39.	Did you have seizures?	1	2	3	4
40.	Did you have weakness on one side of your body?	1	2	3	4
41.	Did you have trouble finding the right words to express yourself?	1	2	3	4
42.	Did you have difficulty speaking?	1	2	3	4
43.	Did you trouble communicating your thoughts?	1	2	3	4
44.	Did you feel drowsy during the daytime?	1	2	3	4
45.	Did you have trouble with your coordination?	1	2	3	4
46.	Did hair loss bother you?				
47.	Did itching of their skin bother you?	1	2	3	4
48.	Did you have weakness of both legs?	1	2	3	4
49.	Did you feel unsteady on your feet?	1	2	3	4
50.	Did you have trouble controlling your bladder?	1	2	3	4

For peer review only

For peer review only - http://bmjopen.bmj.com/site/about/guidelines.xhtml

PROXY ID: FG-XX-XXXX

PROXY QUESTIONNAIRE

Thank you for agreeing to participate in the FUTURE-GB study as a proxy.

This is the 3 months post operation questionnaire.

We would be grateful if you could complete the questionnaire on your opinion of how you friend/spouse/relative is coping in their glioblastoma journey. This is the same questionnaire as all the others you have completed to date.

If you have any questions about the study or this questionnaire, please do not hesitate to contact a member of the study team on future-gb@nds.ox.ac.uk or call 01865 xxxxxx (Monday to Friday, 9-4pm, there is an answering machine for messages outside of these times)

For peer review only

PROXY ID: FG-XX-XXXX

We are interested in some things about the person and their health of the person you have agreed to act as a proxy for. The below questionnaire will use the term friend – we appreciate though that you may be answering about a relative/friend or spouse. We hope you will allow the use of this term for the purposes of this questionnaire.

Please answer all of the questions yourself by circling the number that best applies to them. There are no “right” or “wrong” answers. The information that you provide will remain strictly confidential.

We appreciated that you may be very busy and this may be a distressing time, therefore if you are unable to complete this questionnaire either due to time or other reasons – if at all possible however we would be very grateful if could complete the date below and questions 29 and 30 on page 4.

What is today’s date: DD/MM/YYYY

This document contains the following validated questionnaires:

- EORTC QLQ-C30
- EORTC QLQ - BN20

For peer review only - http://bmjopen.bmj.com/site/about/guidelines.xhtml

PROXY ID: FG-XX-XXXX

		Not at All	A Little	Quite a Bit	Very Much
1.	Does your friend have any trouble doing strenuous activities, like carrying a heavy shopping bag or a suitcase?	1	2	3	4
2.	Does your friend have any trouble taking a long walk?	1	2	3	4
3.	Does your friend have any trouble taking a short walk outside of the house?	1	2	3	4
4.	Does your friend need to stay in bed or a chair during the day?	1	2	3	4
5.	Does your friend need help with eating, dressing, washing themselves or using the toilet?	1	2	3	4

During the past week:

		Not at All	A Little	Quite a Bit	Very Much
6.	Was your friend limited in doing either work or other daily activities?	1	2	3	4
7.	Was your friend limited in pursuing their hobbies or other leisure time activities?	1	2	3	4
8.	Were they short of breath?	1	2	3	4
9.	Have they had pain?	1	2	3	4
10.	Have they needed to rest?	1	2	3	4
11.	Have they had trouble sleeping?	1	2	3	4
12.	Have they felt weak?	1	2	3	4
13.	Have they lacked their appetite?	1	2	3	4
14.	Have they felt nauseated?	1	2	3	4
15.	Have they vomited?	1	2	3	4
16.	Have they been constipated?	1	2	3	4
17.	Have they had diarrhoea?	1	2	3	4
18.	Have they been tired?	1	2	3	4
19.	Has pain interfered with their daily activities?	1	2	3	4
20.	Have they have difficulty concentrating on things, like reading a newspaper or watching television?	1	2	3	4
21.	Have they felt tense?	1	2	3	4
22.	Have they worried?	1	2	3	4
23.	Have they felt irritable?	1	2	3	4
24.	Have they felt depressed?	1	2	3	4
25.	Have they had difficulty remembering things?	1	2	3	4
26.	Has their physical condition or medical treatment interfered with their family life?	1	2	3	4

For peer review only

For peer review only - http://bmjopen.bmj.com/site/about/guidelines.xhtml

PROXY ID: FG-XX-XXXX

		Not at All	A Little	Quite a Bit	Very Much
27.	Has their physical condition or medical treatment interfered with their social activities?	1	2	3	4
28.	Has their physical condition or medical treatment caused them financial difficulties?	1	2	3	4

For the following questions please circle the number between 1 and 7 that best applies to the person you are a proxy for in your opinion

29. How would you rate their overall health during the past week?

1 2 3 4 5 6 7

Very poor Excellent

30. How would you rate their overall quality of life during the past week?

1 2 3 4 5 6 7

Very poor Excellent

For peer review only

Patients sometimes report that they have the following symptoms. Please indicate the extent to which your relative/proxy/friend have experienced the below symptoms or problems during the past week.

During the past week:

		**Not at All**	**A Little**	**Quite a Bit**	**Very Much**
31.	Did they feel uncertain about the future?	1	2	3	4
32.	Did they feel that have had setbacks in their condition?	1	2	3	4
33.	Were they concerned about disruption of family life?	1	2	3	4
34.	Did they have headaches?	1	2	3	4

For peer review only - http://bmjopen.bmj.com/site/about/guidelines.xhtml

PROXY ID: FG-XX-XXXX

		Not at All	A Little	Quite a Bit	Very Much
35.	Did their outlook on the future worsen?	1	2	3	4
36.	Did they have double vision?	1	2	3	4
37.	Has their vision blurred?	1	2	3	4
38.	Did they have difficulty reading because of their vision?	1	2	3	4
39.	Did they have seizures?	1	2	3	4
40.	Did they have weakness on one side of their body?	1	2	3	4
41.	Did they have trouble finding the right words to express themselves?	1	2	3	4
42.	Did they have difficulty speaking?	1	2	3	4
43.	Did they have trouble communicating their thoughts?	1	2	3	4
44.	Did they feel drowsy during the daytime?	1	2	3	4
45.	Did they trouble with their coordination?	1	2	3	4
46.	Did hair loss bother them?				
47.	Did itching of their skin bother them?	1	2	3	4
48.	Did they have weakness of both of their legs?	1	2	3	4
49.	Did they feel unsteady on their feet?	1	2	3	4
50.	Did they have trouble controlling their bladder?	1	2	3	4

For peer review only

For peer review only - http://bmjopen.bmj.com/site/about/guidelines.xhtml

Oxford University Hospitals
NHS Foundation Trust

Functional and Ultrasound guided Resection of Glioblastoma – the FUTURE-GB study –

Stage 1- IDEAL 2b Phase

Patient Information Leaflet

Invitation to join the FUTURE-GB study

We would like to invite you to take part in a research study (also called a clinical trial).

Before you decide whether to take part or not, it is important that you understand why we are doing this study and what it will involve.

Please take time to read the following information and talk to others about the study. If anything is unclear, or if you would like more information, please ask a member of the study team who will be happy to answer any questions.

What is the purpose of this study?

There are many different types of brain tumours. These can vary in how quickly they grow and what symptoms they cause. For a brain tumour that grows quickly it is important to remove as much tumour tissue as possible. To do this without causing damage to important functional parts of the brain involved in speaking, moving etc., we need accurate imaging during surgery. Several different types of imaging are used during operations, but at present we don't know how effective they are and whether they are actually better than standard care.

We have been funded by the National Institute of Health Research (NIHR) which receives its funding from the UK Government, to find out whether some of these available newer technologies improve quality of life, and life expectancy for people with brain tumours who undergo surgery. We also want to see if using these techniques during an operation means it takes more time for a tumour to come back, and if people have fewer complications from the surgery.

Stage 1

In the first part of the study (Stage 1) we are studying how to use these technologies in combination to achieve the best effect. This means that surgeons will be mentored in the best use of the imaging methods. The surgical teams too will be familiarised with a standard way of using them together. We will carefully monitor how the new methods are used during this phase and discuss them with the surgeons. The aim of this stage of the study is to standardise their use, as we want to have them used in the same way in all participating hospitals by the start of Stage 2. Everyone who agrees to take part in this stage may be helping the doctors treating you familiarise themselves with the new technologies. It is highly likely that your surgeon is already familiar with the use of these technologies.

Note: <u>You are only being asked to take part in Stage 1 and if you agree to take part you will only be part of Stage 1 of this study</u>

FUNDED BY NIHR | National Institute for Health Research OCTRU SITU UNIVERSITY OF OXFORD Imperial College London

For peer review only - http://bmjopen.bmj.com/site/about/guidelines.xhtml

Oxford University Hospitals
NHS Foundation Trust

For information – once approximately 5 people have agreed to take part in Stage 1 and their operations have taken place at this hospital, Stage 2 of the study will start at this hospital– the information below tells you more about Stage 2.

Stage 2

Stage 2 of the study will compare the operation using the new imaging techniques with the standard operation - so half of those that agree will have the additional, newer, technologies and half will have the standard operation. We hope this will allow us to find a definite answer about which technologies should be used during an operation. The aim of the surgery is to remove safely as much tumour as possible, whilst minimising the risks of damaging brain function and hence affecting quality of life.

The technologies that will be used in this study are all available and in use across NHS practice, and have been shown to be safe. No one knows whether using all of them together will have a definite positive effect on outcome, but it is logical to expect that it should.

The design of this study (FUTURE-GB) has involved patients, their families and healthcare professionals, including brain surgeons, using their knowledge and experience at every stage of project development.

Who is taking part and why have I been invited to take part?

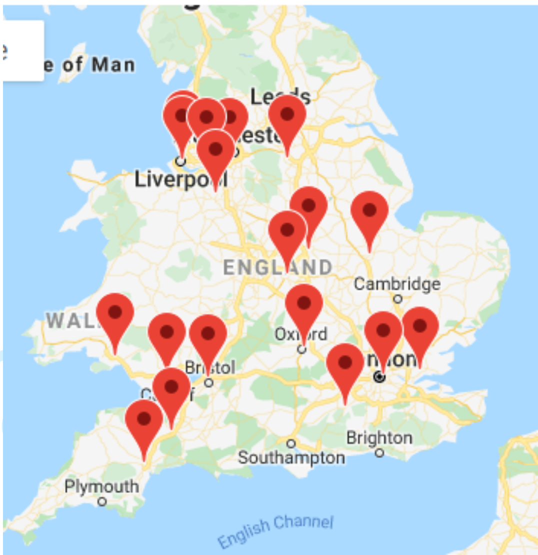

We are hoping to enrol 75 people aged 18 or over, from approximately15 neurosurgical centres in the UK. You will have surgery in your local hospital, which is participating in this trial.

You have been invited to take part because your brain scan suggests you have a brain tumour which comes from the brain itself, rather than from a cancer elsewhere in the body which has spread to the brain. Your scan also suggests the tumour is likely to be aggressive, called a glioblastoma or high-grade tumour.

Do I have to take part in this study?

No, you are under no obligation to take part in the study. Deciding not to will not affect the treatment/care you receive from your team. It is up to you to decide whether to take part or not.

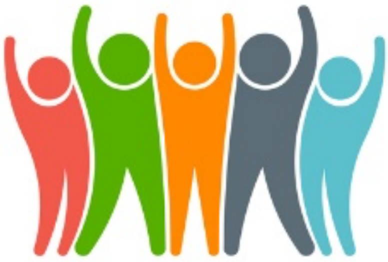

Please keep this leaflet and use it as it may to help you make your decision. If you decide to take part, you will be asked to sign another consent form, as well as that used for your NHS operation.

If you choose not to join the study, you will receive the routine NHS treatment, as agreed by your local treating team of healthcare professionals, in accordance with standard NHS practice as deemed appropriate by your treating team. A note will be made of your age and gender, so that we can find out who decides not to take part. You cannot be identified from this data. A researcher may ask you if you would be happy to give a reason for not wanting to take part in the study. Giving this information is entirely voluntary.

Should you decide to take part, you are still free to withdraw at any time and without giving a reason. This will not affect the standard of care you receive as either an inpatient or an outpatient.

For peer review only - http://bmjopen.bmj.com/site/about/guidelines.xhtml

Oxford University Hospitals
NHS Foundation Trust

What will happen if I take part?

If you are happy to take part in this study, a researcher will ask you some simple questions and check your medical history to confirm that you are eligible.

Initial assessment: If you are eligible and wish to participate, you will be asked to sign and date a consent form for the study. Researchers will then take from your medical notes, information about your brain tumour history including the symptoms you have had and where your tumour is.

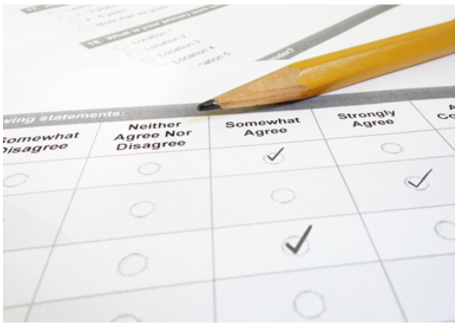

The researchers would then record details about your operation and details of when you leave hospital after your operation. No questions will need to be asked of you as this will either be standard things already being recorded in your notes or when the technologies are being used, the settings being used in your operation.

Most significantly for those that agree to take part in FUTURE-GB the time taken for your preoperative scans (which you will have by being in the study or not) will perhaps take another 5 minutes. Your operation may also be slightly longer due to the technologies being used – this might perhaps extend it by 15 minutes. (Your doctors will talk to you about what happens in your preoperative scans – but we want you to know that some people find them quite claustrophobic – but the scans are needed for your surgery regardless of taking part in FUTURE-GB). Also, those who have metal in their body may potentially not be able to have type of scan called an MRI scan – talk to the doctors if you think you have metal in your body.

Please note: The design of this study has involved patients, their families and healthcare professionals, including brain surgeons, using their knowledge and experience at every stage of its development.

What technologies will be used in my operation?

Surgery will involve standard care and newer technologies

Standard care:

You will have an MRI scan before your operation. This can be used during the surgery to help your surgeon identify where your brain tumour is located, and what brain structures are close by. MRI stands for Magnetic Resonance Imaging. It is a type of scan that uses strong magnetic fields to produce detailed images. It is used for brain tumour surgery to obtain detailed images of your brain and specifically your brain tumour. There is no risk of radiation exposure. All MRI scans that you will receive are received by all those with a brain tumour, anything seen on these scans will be acted upon as per local NHS Trust and national guidelines.

This is combined with use of a chemical called 5-aminolevulinic acid (5-ALA), which is a drink taken a few hours before surgery. This allows the tumour cells to light up pink, when a blue light is shone on them during surgery. This has been shown to help surgeons remove more of the brain tumour, as they are able to see better the edges of the tumour and differentiate it from the surrounding normal brain. This makes sure as much of the tumour is removed as is possible, but it can never usually be totally removed.

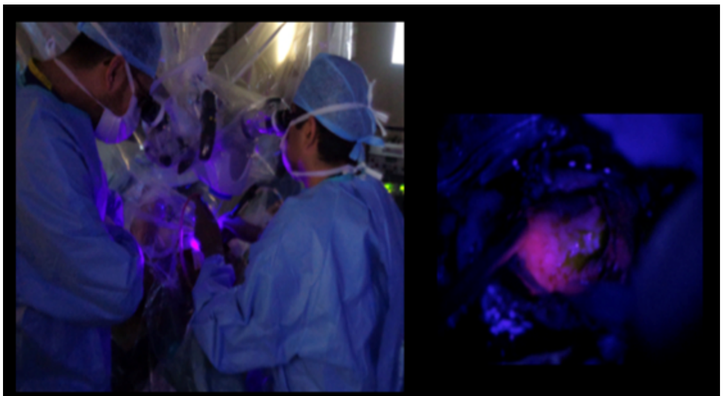

Surgeons using microscope equipped with blue light — Appearance of tumour with 5ALA under blue light

For peer review only - http://bmjopen.bmj.com/site/about/guidelines.xhtml

Oxford University Hospitals
NHS Foundation Trust

Newer technologies:

1) Diffusion Tensor Imaging (DTI) is an MRI technology which allows the surgeons to have a scan of all the nerve fibres which are involved in movement, speech etc. around a tumour. This means that when removing the tumour, the surgeon knows more easily where these are based on your DTI scan and can avoid them.

2) Intraoperative Ultrasound is a technology that uses high frequency sound waves to create an image of the brain tumour during the operation. The ultrasound provides "live" pictures of your tumour as surgery progresses and tumour is removed. The surgeon can use this as many times as necessary during your surgery. The ultrasound is the same as that used to provide a picture of a baby inside a pregnant woman.

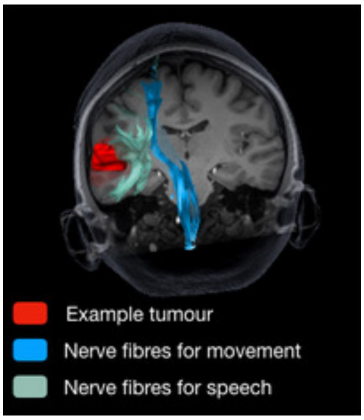

Both these technologies are safe and have been used in brain tumour surgery for a number of years. Your surgeon is familiar with their use and has used them during surgery. However, the benefit of using these 2 new technologies together, in addition to the present "standard of care" surgery has not been scientifically tested, or formally assessed. There are no extra drugs or chemicals used.

Further possible contacts: A researcher from the trial coordinating team may visit while you are having your operation so that we can check how the surgery is being undertaken. We will always check that you are happy for this to happen. If not, the researcher will not come into your operation. At the end of the study, we will report how well the treatments were delivered as it is important we fully understand this process.

Please note, no-one can ever be identified in any public report about the study.

There are 2 companies supporting this study by providing machinery and software to sites if they do not already have the equipment needed for this study. The companies are called BrainLab and Medtronic they are supporting doctors in the UK in using the new technologies. Neither company will be able to influence the results of the study.

What happens after my operation in FUTURE-GB?

Researchers will record information about your operation from your medical notes and directly into the study database, researchers will also collect information from your medical notes and scans when you are discharged home after your operation. The care, any tests, further outpatient appointments and any other surgery or treatments will not be changed by you agreeing to take part in FUTURE-GB. Researchers will check your medical notes for up to 6 months after your operation so find out how you are getting on – but there will be no further contact with you from the study team.

What are the benefits and risks of taking part in the study?

For those that take part in the study, your operation will be conducted by the same surgeon/surgical team whom you have already seen.

We hope the information from this study will answer the question:

Which imaging tools should be used by surgeons when removing a glioblastoma, to offer the highest chance of removing as much of the tumour as possible without causing functional problems, and therefore keeping a good quality of life?

For peer review only

For peer review only - http://bmjopen.bmj.com/site/about/guidelines.xhtml

Oxford University Hospitals
NHS Foundation Trust

We cannot promise the study will help you directly, but the information we get has the potential to be of benefit, potentially allowing more of your tumour to be removed safely.

The risks relating to the brain tumour surgery itself will be discussed with you in detail as part of the standard, routine consent for an operation. We do not think that being part of this study will change any of the risks of the operation but this is one of the things we be will be studying. The technologies will however add some time to the scan before your operation and during your operation.

We are undertaking this study because the extra imaging tools add significant costs to NHS treatment, and therefore we have also been funded to identify whether they provide a real benefit for people with brain tumours.

People sometimes feel uncomfortable answering certain questions about their health, or may be unable to answer. If you, or the person you nominate to answer for you, feel uncomfortable at any point, then you do not have to answer the questions.

Who will know that I am taking part?

The only people who will know that you are taking part in this study are the members of the research team and the healthcare professionals involved in your care. You can tell anyone you would like to that you are taking part.

The only people in the University of Oxford who will have access to information that identifies you will be people who need to contact you to about the study, or review the data. The people who analyse the information will not be able to identify you and will not be able to find out your name or contact details. Also, your de-identified scans will be reviewed by members of the research team and the companies providing the extra imaging technologies. The images will be transferred using cloud servers, however, nothing that could identify you will be included.

Representatives from the Sponsor, relevant regulatory organisations and [insert local Trust] may also need access to monitor or audit the study to ensure that the research is complying with applicable regulations.

Paperwork that is completed by you, the research team, or the treating clinical team, will be sent securely to the study team managing the FUTURE-GB study that are based at the University of Oxford.

Will my details be kept confidential?

Yes. All information collected about you and from you during the course of the research, including from your medical records, will be kept strictly confidential. Everyone who takes part in the study will be assigned a code number and all of the data relating to each person will be held on a computer database and will only be linked to that code number, and not to people's names or addresses. The study team will record into the study database your name, date of birth, NHS or CHI number, Hospital number and your email address. These details will allow the central study team and the local teams to ensure they are collecting data on the correct person. Your email address will only be used to allow you to complete the consent form at home, although this can also be done at the hospital, and to send you a copy of your consent form for your records. Your NHS or CHI number will be used to look up your status 6 months after agreeing to take part.

We will ask you for your permission for individuals from the University of Oxford and Imperial College London, and the regulatory authorities, to have access to your medical notes and data. This is in order that they may conduct checks on the study data that has been collected and to ensure all the study data has been completed correctly. We will also ask you for your permission to allow appropriate individuals from the NHS Trust that you are being approached at to also undertake this review.

At the end of the study, all of the data will be de-identified so that no-one can be identified. This de-identified data will be shared so that more researchers can gain a deeper understanding about patients

For peer review only - http://bmjopen.bmj.com/site/about/guidelines.xhtml

Oxford University Hospitals
NHS Foundation Trust

who have had surgery for glioblastoma. It may be shared with other researchers around the world and with commercial organisations but this information will not identify you, and will not be combined with other information in a way that could identify you. The information will only be used for the purpose of healthcare research, and cannot be used to contact you, nor will it affect your care.

In line with what happens in the NHS, the only situation that confidentiality would need to be broken would be if you told a health professional or research team member of something that could result in harm to yourself or others.

What will happen to my data?

Research is carried out in the public interest. The University of Oxford, as Sponsor, is the data controller. This means that we, as University of Oxford researchers, are responsible for looking after your information as part of FUTURE-GB, and using it properly. We will use the minimum possible personally-identifiable information, and this will be kept for 12 months after the study has finished. Non-identifiable research data and any research documents with personal information, will be stored securely at the University of Oxford for a maximum of 5 years after the end of the study, as part of the research record.

We will be using information from you and your medical records in order to carry out this study. The Oxford University Hospitals NHS Foundation Trust will use your NHS number and contact details to get in touch with you, and to make sure that relevant study information is recorded from your care records. They will keep your identifiable information safely for 12 months after the study has finished. Consent forms and study documents held at [local NHS Trust name] will be archived securely, in accordance with their local procedures.

Data protection regulation provides you with control over your personal data and how it is used. When you agree to your information being used in research, however, some of those rights may be limited, as we need to manage your information in specific ways in order for the research to be reliable and accurate. Further information about your rights with respect to your personal data, is available at https://compliance.web.ox.ac.uk/individual-rights

You can find out more about how we use your information by contacting the FUTURE-GB study team on: futuregb@nds.ox.ac.uk.

What will happen if I don't want to carry on with the study?

You are free to withdraw from taking part in the study at any time without giving a reason. Please remember, it is your decision to take part. If you agree to take part now, but you change your mind during the study, this will not change the standard of care you receive from the NHS. If you were to decide to stop taking part in the study at any time, any data collected on you would be kept. You would not be contacted about the study again or have any further data collected.

What happens at the end of the study?

We will share the results with healthcare researchers and professionals to improve future patient care. Also, we will present them in research reports, at scientific conferences, and publish them in scientific journals, and publish them on the study website futuregb.octru.ox.ac.uk.

We will not include any data that could identify you in the results. If the funders of this research ask us to make the study data available for other researchers, we will first de-identify your information (i.e. we will take your name and other identifying details out) so that you cannot be identified.

For peer review only - http://bmjopen.bmj.com/site/about/guidelines.xhtml

Oxford University Hospitals
NHS Foundation Trust

Who is organising and funding the research?

The University of Oxford is the Sponsor and is organising this study. It is being conducted by a research team led by Prof Puneet Plaha, Consultant Neurosurgeon at the Oxford University Hospitals NHS Foundation Trust and the University of Oxford, and Miss Sophie Camp and Prof. Dipankar Nandi (both Consultant Neurosurgeons at Imperial College Healthcare NHS Trust).

The National Institute of Health Research – Health Technology Assessment programme is funding the study. The funding for the NIHR comes from the UK Government.

Who has approved this study?

A panel of independent researchers and patient representatives, as well as a Research Ethics Committee (REC Reference 20/LO/0840) have reviewed and approved this study.

What if I have concerns?

The University of Oxford, as the study sponsor, has appropriate insurance in place in the unlikely event that you suffer any harm as a direct consequence of your participation in this study.

If you have any concerns or complaints about any aspect of the study, please contact the FUTURE-GB research team using the details below. You can also contact the University of Oxford Research Governance, Ethics & Assurance office on 01865 616480 or by email on ctrg@admin.ox.ac.uk.

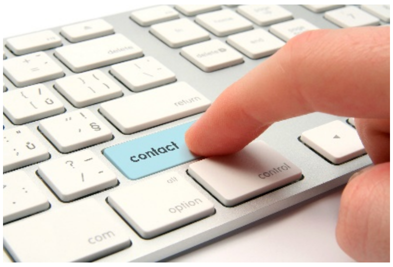

If you would prefer to speak with someone who is not involved in the study, then please contact the Patient Advice and Liaison Service (PALS). PALS is a confidential NHS service that can provide you with support for any complaints or queries you have regarding the care you receive as an NHS patient. However, PALS cannot provide information about this research study.

PALS phone number: [local PALS phone number]

PALS email: [local PALS email]

You can also contact your local clinical team directly:

<local PI/research team name and contact details>

If you have any questions about the study, please contact the FUTURE-GB team on:

Email: futuregb@nds.ox.ac.uk Telephone: 07917 101 649

Postal address: FUTURE-GB study, Botnar Research Centre, Nuffield Orthopaedic Centre, Windmill Road, Oxford, OX3 7LD.

Further information can be found on our study website – futuregb.octru.ox.ac.uk

Thank you for reading this information leaflet and considering taking part.

For peer review only - http://bmjopen.bmj.com/site/about/guidelines.xhtml

LOCAL TRUST LOGO

Functional and Ultrasound guided Resection of Glioblastoma – the FUTURE-GB study – Stage 2 - Randomised Controlled Trial

Patient Information Leaflet

Invitation to join the FUTURE-GB study

We would like to invite you to take part in a research study (also called a clinical trial).

Before you decide whether to take part or not, it is important that you understand why we are doing this study and what it will involve.

Please take time to read the following information and talk to others about the study. If anything is unclear, or if you would like more information, please ask a member of the study team who will be happy to answer any questions.

What is the purpose of this study?

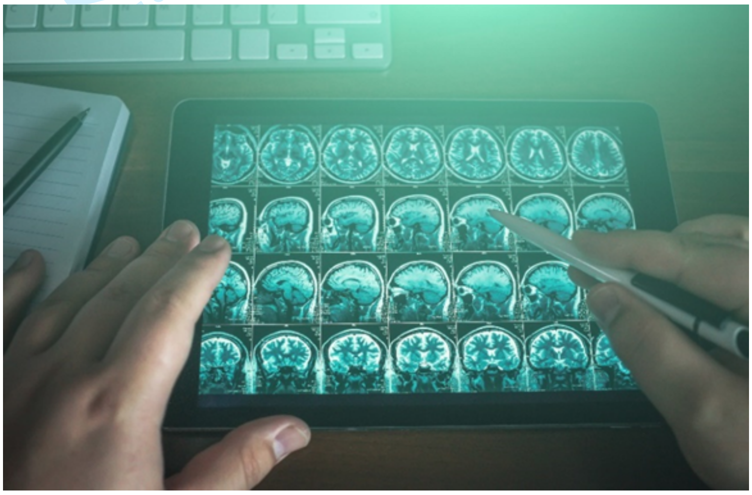

There are many different types of brain tumours. These can vary in how quickly they grow and what symptoms they cause. Studies have shown that when a brain tumour is growing quickly it is better to remove as much tumour as possible. Being able to do this without causing damage to the parts of the brain that are involved in things such as speaking and moving, surgeons need to be able to see clearly parts of the brain during surgery, using accurate imaging. This has led to an increase in the use of imaging (such as ultrasound and MRI scans) during operations. However, we don't know if all the extra imaging tools do definitely make a difference.

We have been funded by the National Institute of Health Research (NIHR) which receives its funding from the UK Government to find out whether some of these additional imaging tools available make a positive difference to quality of life for people with fast growing brain tumours who have surgery. We will also be looking to see if these imaging tools used during an operation mean people with a brain tumour:

- have a better quality of life?
- if it takes more/less time for their tumour to come back
- if they have more/fewer complications from the surgery.

For peer review only - http://bmjopen.bmj.com/site/about/guidelines.xhtml

LOCAL TRUST LOGO

This study will attempt to give a definite answer, so that surgeons know which imaging tools they should use during an operation to enable as much tumour as possible to be removed safely, whilst minimising the risks of damaging brain function and hence affecting quality of life.

The imaging tools that will be used in this study are available and in use across the NHS, and have been shown to be safe. However, no one knows if using them together will have a definite positive effect on outcome for those with a brain tumour.

Who is taking part and why have I been invited to take part?

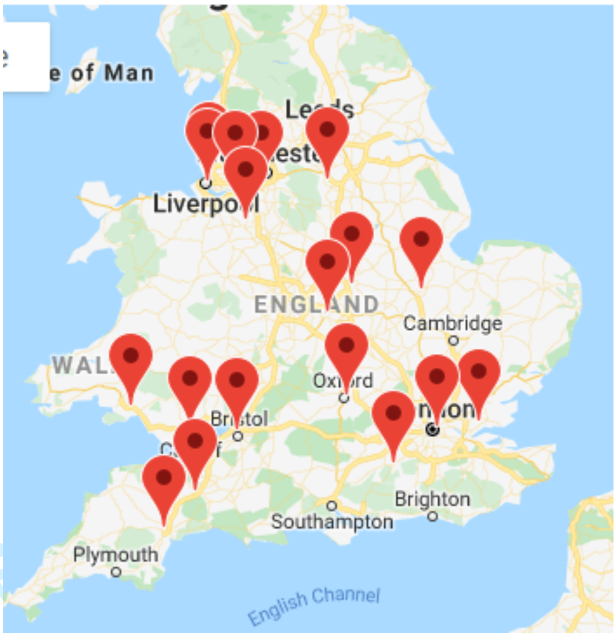

Half of the people taking part in the study will have standard NHS imaging (scans), and the other half will have standard NHS imaging (scans) and some additional imaging (scans).

We want to enrol 357 people aged 18 - 70 from approximately 15 neurosurgical centres in the UK. You will have surgery in your local neurosurgical unit, which is participating in this study.

You have been invited to take part because your brain scan suggests you have a brain tumour, which comes from the brain itself, rather than from a cancer elsewhere in the body which has spread to the brain. Your scan also suggests the tumour is likely to be aggressive, called a glioblastoma, or high grade tumour.

Do I have to take part in this study?

No, you are under no obligation to take part in the study. Deciding not to will not affect the treatment/care you receive from your team. It is up to you to decide whether to take part or not. Please keep this leaflet and use it as it may to help you make your decision. If you decide to take part, you will be asked to sign another consent form, as well as that used for your NHS operation.

If you choose not to join the study, you will receive the routine NHS treatment, agreed by your local treating team of healthcare professionals, in accordance with standard NHS practice using the imaging your treating team deems appropriate. A note will be made of your age and gender, so that we can find out who decides not to take part. You cannot be identified from this data. A researcher may ask you if you would be happy to give a reason for not wanting to take part in the study. Giving this information is entirely voluntary.

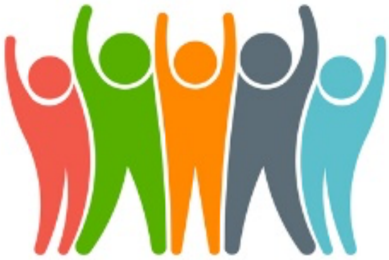

Should you decide to take part, you are still free to withdraw at any time and without giving a reason. This will not affect the standard of care you receive, as either an inpatient or an outpatient.

What will happen if I take part?

If you are happy to take part in this study, a researcher will ask you some simple questions and check your medical history to confirm that you are eligible.

Initial assessment: If you are eligible, you will be asked to sign and date a consent form for the study. We will also ask you to complete some short questionnaires about your health, the activities you are able to

FUNDED BY NIHR | National Institute for Health Research OCTRU SITU UNIVERSITY OF OXFORD Imperial College London

For peer review only - http://bmjopen.bmj.com/site/about/guidelines.xhtml

LOCAL TRUST LOGO

carry out, and about your quality of life. These questionnaires should take you no more than 10-15 minutes to complete. (The questionnaires are electronic – but sometimes you may be given a paper questionnaire to complete, if the electronic system is not available). A researcher will also ask you to complete a brain activity and a recall test, check the strength in your arms and legs and talk to you about how you care for yourself.

Most significantly for those that agree to take part in FUTURE-GB the time taken for your preoperative scans (which you will have by being in the study or not) will perhaps take another 5 minutes. Your operation may also be slightly longer due to the technologies being used – this might perhaps extend it by 15 minutes. (Your doctors will talk to you about what happens in your preoperative scans – but we want you to know that some people find them quite claustrophobic –the scans are needed for your surgery regardless of taking part in FUTURE-GB). Also, those who have metal in their body may potentially not be able to have type of scan called an MRI scan – talk to the doctors if you think you have metal in your body.

Imaging (scans) allocation: You will then be randomly allocated to an imaging group by a computer, which has no information about you as an individual, i.e. allocation is by chance. You will have an equal chance of being allocated to either group, like the toss of a coin. There is a 50% chance that you will be put into the group in which the additional imaging tools will be used during your operation, in addition to the standard techniques, and a 50% chance that you will be in the group where the surgeon uses the present, standard imaging tools.

The random allocation is important because this way, we can test the different imaging tools fairly and nobody can influence into which group you are placed. If you enrol in the study, your healthcare and research teams will not be able to affect which imaging tools will get used in your operation and you will not be able to choose. You will not be aware into which arm of the study you have been allocated, just in case you answer the questionnaires differently based on the imaging used in your operation.

Please note: The design of this study has involved patients, their families and healthcare professionals, including brain surgeons, using their knowledge and experience at every stage of its development.

The FUTURE-GB study aims to find out if the new technologies do, or do not, improve the quality of life of those treated for a brain tumour. We need to know how you are, and your abilities during the study, before and after your operation. We are therefore asking everyone who agrees to take part to nominate a good friend/relative/partner to complete the same questionnaires at the same time as you. They will be asked the same questions as you are asked but they will be asked to answer what their opinion is of your health and abilities.

If you become unable to answer the questions at some point during the study, we would like to know the answers from your good friend/relative/partner to help us identify any changes in your quality of life up to that point in the study. It is helpful to have both assessments to be sure we know about any change in your health status during the study. This is why we would ask both you and your friend/relative/partner to complete the questionnaires throughout the study. The responses that you and your friend/relative/partner give will be used by the trial team to understand the impact of the new technologies on quality of life.
Note: All the data that you and your friend/relative/partner gives will be used by the trial team.

What technologies will be used in my operation?

If you are allocated to the standard techniques arm of the study, your surgeon will use the standard NHS imaging tools.

You will have a Magnetic Resonance Imaging (MRI) scan before your operation. This can be used during the surgery to help your surgeon identify where your brain tumour is located, and what brain structures are close by. This is a type of scan that uses strong magnetic fields to obtain detailed images

For peer review only - http://bmjopen.bmj.com/site/about/guidelines.xhtml

LOCAL TRUST LOGO

of your brain and specifically your brain tumour. There is no risk of radiation exposure. All MRI scans that you will receive are received by all those with a brain tumour, anything seen on these scans will be acted upon as per local NHS Trust and national guidelines.

This is combined with use of a chemical called 5-aminolevulinic acid (5-ALA), which is a drink taken a few hours before surgery. This allows the tumour cells to light up pink, when a blue light is shone on them during surgery. This is known to help surgeons remove more of the brain tumour, as they are able to see better the edges of the tumour compared to the rest of the brain, making sure as much of the tumour is removed as is possible in each individual case.

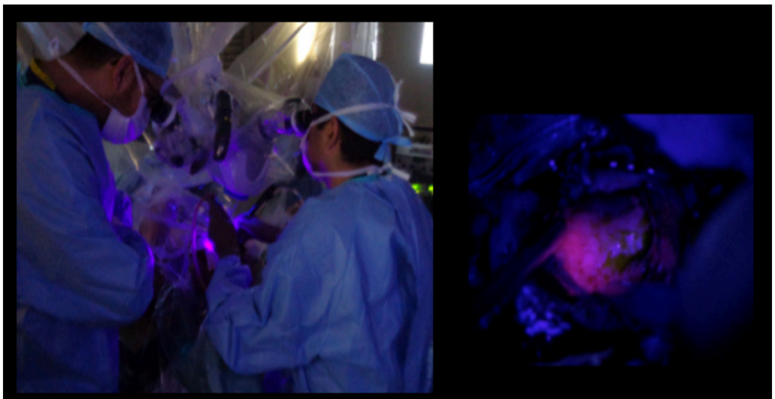
Surgeons using microscope equipped with blue light

Appearance of tumour with 5ALA under blue light

If you are allocated to the group that will use the additional imaging tools, your surgeon will undertake all of what is listed above, together with the additional imaging tools. You will have a slightly longer MRI scan (additional 5 minutes) and have the imaging outlined below also undertaken as part of your operation.

Diffusion Tensor Imaging (DTI) – this is an imaging tool that allows the surgeons to have a scan of all the nerve fibres which are involved in movement, speech etc. around a tumour. This means that when removing the tumour the surgeons potentially know, more accurately, where these are located specifically in your brain from this scan and so can avoid them. This scan is taken using an MRI machine – this is why your MRI scan would be 5 minutes longer.

Intraoperative Ultrasound – this is a technology whereby high frequency sound waves are used to create an image of the brain tumour during the operation. The ultrasound provides "live" pictures of your tumour as surgery progresses and tumour is removed. The surgeon can use this as many times as necessary during your surgery. The ultrasound is the same as that used to provide a picture of a baby inside a pregnant woman.

Both these imaging tools are safe and are used in brain tumour surgery already, but their benefit has not been formally assessed. There are no extra drugs or chemicals used for the additional imaging tools.

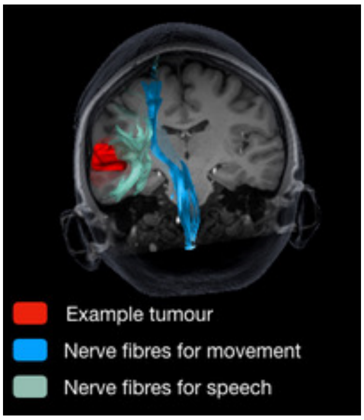

There are 2 companies supporting this study by providing machinery and software to sites if they do not already have the equipment needed for this study. The companies are called BrainLab and Medtronic they are supporting doctors in the UK in using the new technologies. Neither company will be able to influence the results of the study.

Further possible contacts: A researcher from the trial coordinating team may visit while you are having your operation so that we can check how the surgery is being undertaken. We will always check that you are happy for this to happen. If not, the researcher will not come into your operation. At the end of the study, we will report how well the treatments were delivered as it is important we fully understand this process.

For peer review only - http://bmjopen.bmj.com/site/about/guidelines.xhtml

LOCAL TRUST LOGO

What happens after my operation in FUTURE-GB?

If you take part in the study, we will not know whether the additional imaging tools are helpful or not until many months after your operation.

As part of the study you will be asked to complete some further questionnaires. The questionnaires ask about you, your health and activity, and your quality of life. We ask that you complete the questionnaires before you have surgery, when you leave hospital, at 6 and 12 weeks after surgery, and then every 3 months, for a maximum of 2 years. These time points are when you would usually be coming back to the hospital for routine NHS care, and it may be possible to complete them at these appointments. However, they will also be emailed to you for online completion. In addition, either when you are at the hospital for your outpatient appointments, or via telephone at those timepoints, a researcher will also ask you to complete a brain activity and a recall test, and talk to you about how you care for yourself, these will be the same tests and questions as before your operation.

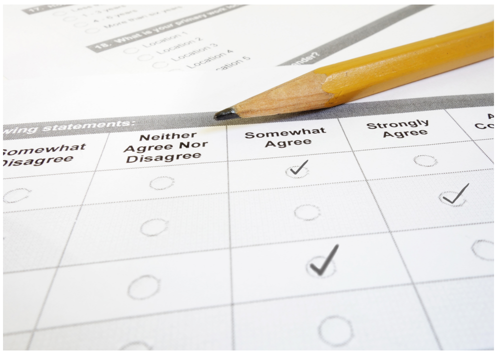

The questionnaires should take no more than 10 minutes to complete on paper/online, or over the telephone. The questionnaires are all completed online, but speak to your researcher if you want to take part but do not have access to the internet.

Please note, the care, any tests, further outpatient appointments and any other surgery or treatments will not be changed by you agreeing to take part in FUTURE-GB. Researchers will check your medical notes for up to 24 months after your operation to find out how you are getting on, and the trial team will use your details to send out the follow-up questionnaires.

Please note if you are deemed to lose capacity at any point during the study, you will not be asked to complete any further questionnaires.

What are the benefits and risks of taking part in the study?

For those that take part in the study, your operation will be conducted by the same surgeon/surgical team whom you have already met.

The information from this study we hope will answer the question:

Which imaging tools should be used by surgeons when removing a glioblastoma, to offer the highest chance of removing as much of the tumour as possible without causing functional problems, and therefore keeping a good quality of life?

We cannot promise the study will help you directly, but the information we get has the potential to be of benefit, potentially allowing more of your tumour to be removed safely.

The risks relating to the brain tumour surgery itself will be discussed with you in detail as part of the standard, routine consent for an operation. We don't think that being part of this study will change any of the risks of the operation but this is one of the things we be will be studying. The technologies will however add some time to the scan you have before your operation and your operation if you are put into the group where the new technologies are used.

We are undertaking this study because we want to find out whether the extra imaging tools provide a real benefit for people with brain tumours. These add significant costs to NHS treatment, and so we need to know if they are worth the extra cost.

For peer review only - http://bmjopen.bmj.com/site/about/guidelines.xhtml

LOCAL TRUST LOGO

People sometimes feel uncomfortable answering certain questions about their health, or may be unable to answer. If you, or the person you nominate to answer for you, feel uncomfortable at any point, then you do not have to answer the questions.

We are not able to pay travel expenses for you to attend your follow-up sessions, however any research questions asked will be as part of your routine out-patient follow-up appointments, via email, or over the telephone.

Who will know that I am taking part?

The only people who will know that you are taking part in this study are the members of the research team and the healthcare professionals involved in your care. You can tell anyone you would like to that you are taking part.

The only people in the University of Oxford who will have access to information that identifies you will be people who need to contact you to about the study, or review the data. The people who analyse the information will not be able to identify you and will not be able to find out your name or contact details. Also, your de-identified scans will be reviewed by members of the research team and the companies providing the extra imaging technologies. The images will be transferred using secure cloud servers, however, nothing that could identify you will be included.

Representatives from the sponsor, relevant regulatory organisations and [Insert local Trust] may also need access to monitor or audit the study to ensure that the research is complying with applicable regulations.

Paperwork that is completed by you, your nominee (friend/relative/partner), the research team, or the treating clinical team, will be sent securely to the study team managing the FUTURE-GB study that are based at the University of Oxford.

We will contact your GP (doctor) to tell them that you have agreed to take part in the FUTURE-GB study. However, we do not share with them anything you answer in your study questionnaires.

Will my details be kept confidential?

Yes. All information collected about you and from you and your nominee (friend/relative/partner) during the course of the research, including from your medical records, will be kept strictly confidential. Everyone who takes part in the study will be assigned a code number and all of the data relating to each person will be held on a computer database and will only be linked to that code number, and not to people's names or addresses. The study team will record into the study database your name, date of birth, NHS or CHI number, Hospital number, address, phone number, GP name and address, and your email address. These details will allow the central study team and the local teams to ensure they are collecting data on the correct person. Your email address will only be used to send you a copy of your consent form for your records and any follow-up questionnaires. This is also the reason your address will be kept on file – in case your questionnaires need to be posted to you to complete. Your phone number will be used by the researchers to call and ask you the questions about brain activity and recall if these cannot be completed at your outpatient appointment. Your NHS or CHI number will be used to check your status 24 months after agreeing to take part. Your GPs details will be used to send a letter to your GP informing them of you taking part in this study.

We will ask you for your permission for individuals from the University of Oxford and Imperial College London, and the regulatory authorities, to have access to your medical notes and data. This is in order that they may conduct checks on the study data that has been collected and to ensure all the study data has been completed correctly. We will also ask you for your permission to allow appropriate individuals from the NHS Trust that you are being approached at to also undertake this review.

For peer review only - http://bmjopen.bmj.com/site/about/guidelines.xhtml

LOCAL TRUST LOGO

At the end of the study, all of the data will be de-identified so that no-one can be identified. This de-identified data will be shared so that more researchers can gain a deeper understanding about patients who have had surgery for glioblastoma. It may be shared with other researchers around the world and with commercial organisations but this information will not identify you, and will not be combined with other information in a way that could identify you. The information will only be used for the purpose of healthcare research, and cannot be used to contact you, nor will it affect your care.

In line with what happens in the NHS, the only situation that confidentiality would need to be broken would be if you told a health professional or research team member of something that could result in harm to yourself or others.

What will happen to my data?

For peer review only

Research is carried out in the public interest. The University of Oxford, as Sponsor, is the data controller. This means that we, as University of Oxford researchers, are responsible for looking after your information as part of FUTURE-GB, and using it properly. We will use the minimum possible personally-identifiable information, and this will be kept for 12 months after the study has finished. Non-identifiable research data and any research documents with personal information, will be stored securely at the University of Oxford for a maximum of 5 years after the end of the study, as part of the research record.

We will be using information from you and your medical records in order to carry out this study. The [local NHS Trust name] will use your NHS number and contact details to get in touch with you, and to make sure that relevant study information is recorded from your care records. They will keep your identifiable information safely for 12 months after the study has finished. Consent forms and study documents held at [local NHS Trust name] will be archived securely, in accordance with their local procedures.

Data protection regulation provides you with control over your personal data and how it is used. When you agree to your information being used in research, however, some of those rights may be limited, as we need to manage your information in specific ways in order for the research to be reliable and accurate. Further information about your rights with respect to your personal data, is available at https://compliance.web.ox.ac.uk/individual-rights

You can find out more about how we use your information by contacting the FUTURE-GB study team on: futuregb@nds.ox.ac.uk.

What will happen if I don't want to carry on with the study?

You are free to withdraw from taking part in the study at any time without giving a reason. Please remember, it is your decision to take part. If you agree to take part now, but you change your mind during the study, this will not change the standard of care you receive from the NHS. If you were to decide to stop taking part in the study at any time, any data collected on you would be kept. You would not be contacted about the study again or have any further data collected about you from your medical records. If you withdraw or lose capacity please note we will not continue to contact your nominee (proxy).

What happens at the end of the study?

We will share the results with healthcare researchers and professionals to improve future patient care. Also, we will present them in research reports, at scientific conferences, and publish them in scientific journals, and publish them on the study website: futuregb.octru.ox.ac.uk.

For peer review only - http://bmjopen.bmj.com/site/about/guidelines.xhtml

LOCAL TRUST LOGO

We will not include any data that could identify you in the results. If the funders of this research ask us to make the study data available for other researchers, we will first de-identify your information (i.e. we will take your name and other identifying details out) so that you cannot be identified.

Who is organising and funding the research?

The University of Oxford is the Sponsor and is organising this study. It is being conducted by a research team led by Prof. Puneet Plaha, Consultant Neurosurgeon at the Oxford University Hospitals NHS Foundation Trust and the University of Oxford, and Ms Sophie Camp and Prof. Dipankar Nandi (both Consultant Neurosurgeons at Imperial College Healthcare NHS Trust).

The National Institute of Health Research – Health Technology Assessment programme is funding the study. The funding for the NIHR comes from the UK Government.

Who has approved this study?

A panel of independent researchers and patient representatives, as well as a Research Ethics Committee (REC Reference 20/LO/0840) have reviewed and approved this study.

What if I have concerns?

The University of Oxford, as the study sponsor, has appropriate insurance in place in the unlikely event that you suffer any harm as a direct consequence of your participation in this study.

If you have any concerns or complaints about any aspect of the study, please contact the FUTURE-GB research team using the details below. You can also contact the University of Oxford Research Governance, Ethics & Assurance office on 01865 616480 or by email on ctrg@admin.ox.ac.uk.

If you would prefer to speak with someone who is not involved in the study, then please contact the Patient Advice and Liaison Service (PALS). PALS is a confidential NHS service that can provide you with support for any complaints or queries you have regarding the care you receive as an NHS patient. However, PALS cannot provide information about this research study.

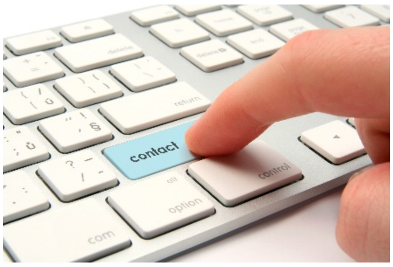

PALS phone number: <Insert local PALS number>

PALS email: <insert local PALS email address>

You can also contact your local clinical team directly:

<local PI/research team name and contact details>

If you have any questions about the study, please contact the FUTURE-GB team on:

Email: futuregb@nds.ox.ac.uk Telephone: 07917 101 649

Postal address: FUTURE-GB study, Botnar Research Centre, Nuffield Orthopaedic Centre, Windmill Road, Oxford, OX3 7LD.

Further information can be found on our study website – futuregb.octru.ox.ac.uk

Thank you for reading this information leaflet and considering taking part.

FUTURE GB

FUNDED BY NIHR National Institute for Health Research | OCTRU | SITU | UNIVERSITY OF OXFORD | Imperial College London

For peer review only - http://bmjopen.bmj.com/site/about/guidelines.xhtml

For peer review only

LOCAL TRUST LOGO

Functional and Ultrasound guided Resection of Glioblastoma – the FUTURE-GB study – This is for those that are asked to consider supporting a potential participant in the Future-GB study

Partner/Relative/Friend (Proxy) Information Leaflet

Invitation to join the FUTURE-GB study

You are receiving this information leaflet as a friend/relative/partner of yours has been approached to take part in this study. As part of the study they are asked to nominate a friend or relative who would be willing to answer some questionnaires about them (a 'proxy'). As the nominated person, we would like to invite you to take agree to part in a research study (also called a clinical trial), which for you will only involve answering questionnaires.

Before you decide whether to take part or not, it is important that you understand why we are doing this study and what it will involve.

Please take time to read the following information and talk to others about the study. If anything is unclear, or if you would like more information, please ask a member of the study team who will be happy to answer any questions.

What is the purpose of this study?

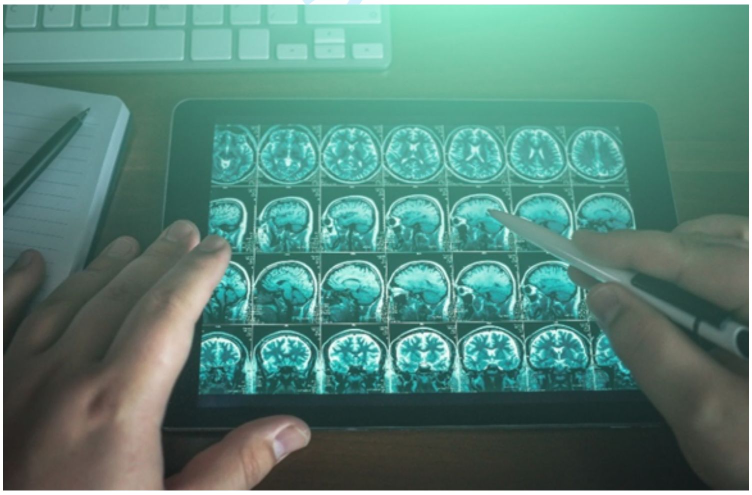

There are many different types of brain tumours. They can vary in how quickly they grow and what symptoms they cause. Studies have shown that when a brain tumour is growing quickly it is better to remove as much tumour as possible. Being able to do this without causing damage to the parts of the brain that are involved in things such as speaking and moving, surgeons need to be able to see clearly parts of the brain during surgery using accurate imaging. This has led to an increase in the use of imaging (such as ultrasound

FUNDED BY NIHR | National Institute for Health Research · OCTRU · SITU · UNIVERSITY OF OXFORD · Imperial College London

For peer review only - http://bmjopen.bmj.com/site/about/guidelines.xhtml

LOCAL TRUST LOGO

and MRI scans) during operations. However, we don't know if all the extra imaging tools do definitely make a difference.

We have been funded by the National Institute of Health Research (NIHR) which receives its funding from the UK Government, to find out whether some of these additional imaging tools available make a positive difference to quality of life for people with brain tumours who have surgery. We will also be looking to see if these imaging tools used during an operation mean people with a brain tumour:

- have a better quality of life?
- if it takes more/less time for their tumour to come back
- if they have more/fewer complications from the surgery

This study will attempt to find a definite answer, so that surgeons know which imaging tools they should use during an operation to enable as much tumour as possible to be removed safely, whilst minimising the risks of damaging brain function and hence affecting quality of life.

The imaging tools that will be used in this study are available across the NHS, and have been shown to be safe. However, no one knows if using them together will have a definite positive effect on outcome for those with a brain tumour. We would encourage you to read the Participant Information Sheet to find out more about the study.

Who is taking part and why have I been invited to take part?

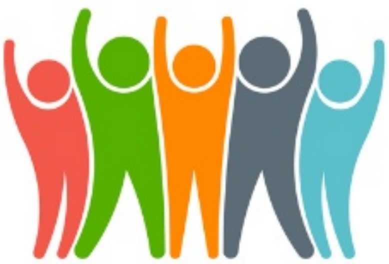

We are hoping to enrol a nominated relative/friend/partner (proxy) from each of the 357 people aged 18 -70 we plan to recruit to the trial, from approximately 15 neurosurgical centres in the UK who have agreed to take part in the FUTURE-GB study.

Specifically, the FUTURE-GB study aims to find out if the new technologies do or do not improve the quality of life of those treated for a brain tumour. We need to know how those taking part are and their abilities during the study, before and after their operation. If your friend/partner/relative becomes unable to answer the questionnaires at some point during the study, we would like to also have answers from you to help us identify any changes in their quality of life over the course of the study. However, if we need to use your answers instead of theirs – we can only do this if we know your answers at the start of the study, so that we can work out what changes have occurred. This is why we would ask both you and your friend/relative/partner to complete the questionnaires throughout the study. The responses that you and your friend/relative/partner give will be used by the trial team to understand the impact of the new technologies on quality of life

Do I have to take part in this study?

No, you are under no obligation to take part in the study. Deciding not to will not affect the treatment/care your friend/relative/partner receives. It is up to you to decide whether to take part or not. Please keep this leaflet and use it as it may to help you make your decision. If you decide to take part, you will be asked to sign a consent form.

FUNDED BY NIHR National Institute for Health Research | OCTRU | SITU | UNIVERSITY OF OXFORD | Imperial College London

For peer review only - http://bmjopen.bmj.com/site/about/guidelines.xhtml

LOCAL TRUST LOGO

What will happen if I take part?

If you are happy to take part in this study, a researcher will contact you to complete questionnaires before your friend/relative/partner's operation, 5 days after their operation or when they leave hospital, 6 weeks after their operation and then every 3 months after your friend/relative/partner's operation for a maximum of 2 years.

Questionnaires take no more than 10 minutes to complete on paper/online, or over the telephone.

Please note if the person who has nominated you withdraws from the study or loses their capacity to consent this will complete your involvement with the study.

What are the benefits and risks of taking part in the study?

For those that take part in the study, your friend/relative/partner's operation will be conducted by the same surgeon/surgical team whom they have already seen.

The information from this study we hope will answer the question:

Which imaging tools should be used by surgeons when removing a glioblastoma, to offer the highest chance of removing as much of the tumour as possible without causing functional problems, and therefore keeping a good quality of life?

We cannot promise the study will help your friend/relative/partner directly, but the more information we collect, the greater the potential to be of benefit, as more of the tumour may be removed.

We are undertaking this study because we want to find out whether the extra imaging tools provide a real benefit for people with brain tumours. These add significant costs to NHS treatment, and so we need to know if they are worth the extra cost.

People sometimes feel uncomfortable answering certain questions about a person's health, or may be unable to answer. If you feel uncomfortable at any point, then you do not have to answer the questions.

Who will know that I am taking part?

The only people who will know that you are taking part in this study are the members of the research team and the person who nominated you. You can tell anyone you would like to that you are taking part.

The only people in the University of Oxford who will have access to information that identifies you will be people who need to contact you to about the study, or review the data. The people who analyse the information will not be able to identify you and will not be able to find out your name or contact details. Representatives from the sponsor, relevant regulatory organisations and [local NHS Trust name] may also need access to monitor or audit the study to ensure that the research is complying with applicable regulations.

Paperwork that is completed by you, your friend/relative/partner, the research team, or the treating clinical team, will be sent securely to the study team managing the FUTURE-GB study, who are based at the University of Oxford.

For peer review only - http://bmjopen.bmj.com/site/about/guidelines.xhtml

LOCAL TRUST LOGO

Will the details be kept confidential?

Yes. All information collected from you during the course of the research, will be kept strictly confidential. Everyone who takes part in the study will be assigned a code number and all of the data relating to each person will be held on a computer database and will only be linked to that code number, and not to people's names or addresses. The study team will record into the study database your name, age, relationship to the study participant, address, and your email address. These details will allow the central study team and the local teams to ensure they are collecting data on the correct person. Your email address will only be used to send you a copy of your consent form for your records and any follow-up questionnaires. This is also the reason your address will be kept on file – in case your questionnaires need to be posted to you to complete.

At the end of the study, all of the data will be de-identified so that no-one can be identified. This de-identified data will be shared so that more researchers can gain a deeper understanding about patients who have had surgery for glioblastoma. It may be shared with other researchers around the world and with commercial organisations but this information will not identify you, and will not be combined with other information in a way that could identify you. The information will only be used for the purpose of healthcare research, and cannot be used to contact you, nor will it affect your care.

In line with what happens in the NHS, the only situation that confidentiality would need to be broken would be if you told a health professional or research team member of something that could result in harm to yourself or others.

What will happen to my data?

Research is carried out in the public interest. The University of Oxford, as Sponsor, is the data controller. This means that we, as University of Oxford researchers, are responsible for looking after your information as part of FUTURE-GB, and using it properly. We will use the minimum possible personally-identifiable information, and this will be kept for 12 months after the study has finished. Non-identifiable research data and any research documents with personal information, will be stored securely at the University of Oxford for a maximum of 5 years after the end of the study, as part of the research record.

The local and central FUTURE-GB study team might use your contact details to get in touch with you. They will keep your identifiable information safely for 12 months after the study has finished. Consent forms and study documents held at [local NHS Trust name] will be archived securely, in accordance with their local procedures.

Data protection regulation provides you with control over your personal data and how it is used. When you agree to your information being used in research, however, some of those rights may be limited, as we need to manage your information in specific ways in order for the research to be reliable and accurate. Further information about your rights with respect to your personal data, is available at https://compliance.web.ox.ac.uk/individual-rights

You can find out more about how we use your information by contacting the FUTURE-GB study team on: futuregb@nds.ox.ac.uk.

For peer review only - http://bmjopen.bmj.com/site/about/guidelines.xhtml

LOCAL TRUST LOGO

Who is organising and funding the research?

The University of Oxford is the Sponsor and is organising this study. It is being conducted by a research team led by Professor Puneet Plaha, Consultant Neurosurgeon at the Oxford University Hospitals NHS Foundation Trust and the University of Oxford, and Miss Sophie Camp and Prof. Dipankar Nandi (both Consultant Neurosurgeons at Imperial College Healthcare NHS Trust, London).

The National Institute of Health Research – Health Technology Assessment programme is funding the study. The funding for the NIHR comes from the UK Government.

Who has approved this study?

A panel of independent researchers and patient representatives, as well as a Research Ethics Committee (REC Reference 20/LO/0840) have reviewed and approved this study.

What if I have concerns?

The University of Oxford, as the study sponsor, has appropriate insurance in place in the unlikely event that you suffer any harm as a direct consequence of your participation in this study.

If you have any concerns or complaints about any aspect of the study, please contact the FUTURE-GB research team using the details below. You can also contact the University of Oxford Research Governance, Ethics & Assurance office on 01865 616480 or by email on ctrg@admin.ox.ac.uk.

If you would prefer to speak with someone who is not involved in the study, then please contact the Patient Advice and Liaison Service (PALS). PALS is a confidential NHS service that can provide you with support for any complaints or queries you have regarding the care you receive as an NHS patient. However, PALS cannot provide information about this research study.

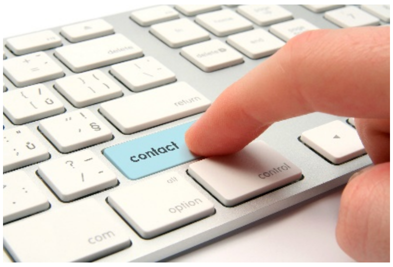

PALS phone number: <Insert local PALS number>

PALS email: <insert local PALS email address>

You can also contact your local clinical team directly:

<local PI/research team name and contact details>

If you have any questions about the study, please contact the FUTURE-GB team on:

Email: futuregb@nds.ox.ac.uk Telephone: 07917 101 649

Postal address: FUTURE-GB study, Botnar Research Centre, Nuffield Orthopaedic Centre, Windmill Road, Oxford, OX3 7LD.

Further information can be found on our study website – futuregb.octru.ox.ac.uk

Thank you for reading this information leaflet and considering taking part.

FUNDED BY NIHR | National Institute for Health Research

For peer review only

For peer review only - http://bmjopen.bmj.com/site/about/guidelines.xhtml

University of Oxford Logo

CONSENT FORM (Stage 1-IDEAL Phase)

FUTURE GB

Imperial Logo

If you agree, **please check box**

1. I confirm that I have read and understood the Information Leaflet dated DDMon20YY version XX. I have had the opportunity to consider the information, ask questions and have had these answered satisfactorily.	
2. I understand that my participation is voluntary and that I am free to withdraw at any time without giving any reason, and without my medical care or legal rights being affected. After withdrawal from the study any data collection from databases will stop.	
3. I understand that relevant sections of my medical notes and data collected during the study may be looked at by individuals from the University of Oxford and Imperial College, London, from regulatory authorities and from the NHS Trusts, where it is relevant to me taking part in this research. I give permission for these individuals to have access to my records.	
4. I consent to the research team holding my contact details so that they can contact me about the study if required. I understand these details will be held securely and destroyed 12 months after the end of the study.	
5. I understand that the information held and maintained by NHS Digital / NHS Central Register may be used to help contact me or provide information about my health status over the next 12 months.<br>I understand and give permission for my NHS/CHI number to be used for this purpose.	
6. I agree to take part in the FUTURE-GB study.	
7. I agree that my operation may be observed for quality assurance purposes by a member of the FUTURE-GB study team. Yes / No	

**Name of Participant**	**Date**	**Signature**
**Name of Person Taking Consent**	**Date**	**Signature**

For peer review only - http://bmjopen.bmj.com/site/about/guidelines.xhtml

University of Oxford Logo

Imperial Logo

CONSENT FORM

If you agree, **please check box**

1. I confirm that I have read and understood the Information Leaflet dated DDMon20YY version XX. I have had the opportunity to consider the information, ask questions and have had these answered satisfactorily.	
2. I understand that my participation is voluntary and that I am free to withdraw at any time without giving any reason, and without my medical care or legal rights being affected. After withdrawal from the study any data collection from databases will stop.	
3. I understand that relevant sections of my medical notes and data collected during the study may be looked at by individuals from the University of Oxford and Imperial College, London, from regulatory authorities and from the NHS Trusts, where it is relevant to me taking part in this research. I give permission for these individuals to have access to my records.	
4. I consent to the research team holding my contact details so that they can contact me about the study. I understand these details will be held securely and destroyed 12 months after the end of the study.	
5. I agree to my General Practitioner (GP) being informed of my participation in the study.	
6. I understand that the information held and maintained by NHS Digital / NHS Central Register may be used to help contact me or provide information about my health status for the next 24 months. I understand and give permission for my NHS/CHI number to be used for this purpose.	
7. If I complete any questionnaires online or via the telephone regarding the FUTURE-GB study, I agree for these to be passed to the hospital I was recruited at for this study.	
8. I agree to take part in the FUTURE-GB study.	
9. I nominate the following person to be my proxy and to answer questionnaires about me during my time in the FUTURE-GB study. They will no longer be asked to provide information about me if I withdraw from the study or lose capacity. Proxy name: (INSERT NAME HERE)	
10. I agree that my operation may be observed for quality assurance purposes by a member of the FUTURE-GB study team. Yes / No	

Name of Participant **Date** **Signature**

Name of Person Taking Consent **Date** **Signature**

For peer review only - http://bmjopen.bmj.com/site/about/guidelines.xhtml

University of Oxford Logo

FUTURE GB **PROXY CONSENT FORM**

Imperial Logo

	If you agree, **please check box**
1. I confirm that I have read and understood the Proxy Information Leaflet dated DDMon20YY version XX. I have had the opportunity to consider the information, ask questions and have had these answered satisfactorily.	
2. I understand that my participation is voluntary and that I am free to withdraw at any time without giving any reason, and without my relative/friend's medical care or legal rights being affected.	
3. I consent to the research team holding my contact details so that they can contact me about the study. I understand these details will be held securely and destroyed at the 12 months after the end of the study.	
4. I understand that as long as I feel able to I will complete regular questionnaires about the abilities and quality of life of the person I have been nominated to be the Proxy of. I understand if the person who nominated me withdraws or loses capacity – this will also end my participation.	
5. I agree to take part in the FUTURE-GB study as a Proxy Representative.	

Name of Participant: ____________________

Relationship to the participant: Partner ☐ Family member ☐ Carer ☐ Friend ☐
Other (please specify) ____________________

Approximately how much time do you spend with the FUTURE-GB participant per week?
- I am in contact with them daily ☐
- I am in contact with them every few days ☐
- I am in contact with them weekly ☐
- I am in contact with them every 2 weeks ☐
- I am in contact with them monthly ☐

Name of Proxy Representative Signature: ____________ Date: ____________

Name of Person Taking Consent Signature: ____________ Date: ____________

Deleted: FUTUREGB_ProxyICF_V3.0_10Jun2021_clean.docx

FUTUREGB_ProxyICF_V3.0_10Jun2021.docx IRAS ID: 264482
Co- Investigator: Prof Puneet Plaha, Ms Sophie Camp and Prof Dipankar Nandi.

For peer review only

Reporting checklist for protocol of a clinical trial.

Based on the SPIRIT guidelines.

Instructions to authors

Complete this checklist by entering the page numbers from your manuscript where readers will find each of the items listed below.

Your article may not currently address all the items on the checklist. Please modify your text to include the missing information. If you are certain that an item does not apply, please write "n/a" and provide a short explanation.

Upload your completed checklist as an extra file when you submit to a journal.

In your methods section, say that you used the SPIRITreporting guidelines, and cite them as:

Chan A-W, Tetzlaff JM, Gøtzsche PC, Altman DG, Mann H, Berlin J, Dickersin K, Hróbjartsson A, Schulz KF, Parulekar WR, Krleža-Jerić K, Laupacis A, Moher D. SPIRIT 2013 Explanation and Elaboration: Guidance for protocols of clinical trials. BMJ. 2013;346:e7586

		Reporting Item	Page Number
**Administrative information**			
Title	#1	Descriptive title identifying the study design, population, interventions, and, if applicable, trial acronym	1
Trial registration	#2a	Trial identifier and registry name. If not yet registered, name of intended registry	2
Trial registration: data set	#2b	All items from the World Health Organization Trial Registration Data Set	
Protocol version	#3	Date and version identifier	1
Funding	#4	Sources and types of financial, material, and other support	14
Roles and responsibilities: contributorship	#5a	Names, affiliations, and roles of protocol contributors	1
Roles and	#5b	Name and contact information for the trial sponsor	

For peer review only

responsibilities: sponsor contact information			
Roles and responsibilities: sponsor and funder	#5c	Role of study sponsor and funders, if any, in study design; collection, management, analysis, and interpretation of data; writing of the report; and the decision to submit the report for publication, including whether they will have ultimate authority over any of these activities	
Roles and responsibilities: committees	#5d	Composition, roles, and responsibilities of the coordinating centre, steering committee, endpoint adjudication committee, data management team, and other individuals or groups overseeing the trial, if applicable (see Item 21a for data monitoring committee)	
**Introduction**			
Background and rationale	#6a	Description of research question and justification for undertaking the trial, including summary of relevant studies (published and unpublished) examining benefits and harms for each intervention	4-5
Background and rationale: choice of comparators	#6b	Explanation for choice of comparators	4-5
Objectives	#7	Specific objectives or hypotheses	6
Trial design	#8	Description of trial design including type of trial (eg, parallel group, crossover, factorial, single group), allocation ratio, and framework (eg, superiority, equivalence, non-inferiority, exploratory)	6
**Methods: Participants, interventions, and outcomes**			
Study setting	#9	Description of study settings (eg, community clinic, academic hospital) and list of countries where data will be collected. Reference to where list of study sites can be obtained	6

For peer review only

Eligibility criteria	#10	Inclusion and exclusion criteria for participants. If applicable, eligibility criteria for study centres and individuals who will perform the interventions (eg, surgeons, psychotherapists)	7
Interventions: description	#11a	Interventions for each group with sufficient detail to allow replication, including how and when they will be administered	6-7
Interventions: modifications	#11b	Criteria for discontinuing or modifying allocated interventions for a given trial participant (eg, drug dose change in response to harms, participant request, or improving / worsening disease)	6
Interventions: adherance	#11c	Strategies to improve adherence to intervention protocols, and any procedures for monitoring adherence (eg, drug tablet return; laboratory tests)	
Interventions: concomitant care	#11d	Relevant concomitant care and interventions that are permitted or prohibited during the trial	
Outcomes	#12	Primary, secondary, and other outcomes, including the specific measurement variable (eg, systolic blood pressure), analysis metric (eg, change from baseline, final value, time to event), method of aggregation (eg, median, proportion), and time point for each outcome. Explanation of the clinical relevance of chosen efficacy and harm outcomes is strongly recommended	5, table 1
Participant timeline	#13	Time schedule of enrolment, interventions (including any run-ins and washouts), assessments, and visits for participants. A schematic diagram is highly recommended (see Figure)	Fig 1
Sample size	#14	Estimated number of participants needed to achieve study objectives and how it was determined, including clinical and statistical assumptions supporting any sample size calculations	9
Recruitment	#15	Strategies for achieving adequate participant enrolment to reach target sample size	8
**Methods: Assignment of**			

For peer review only

**interventions (for controlled trials)**			
Allocation: sequence generation	#16a	Method of generating the allocation sequence (eg, computer-generated random numbers), and list of any factors for stratification. To reduce predictability of a random sequence, details of any planned restriction (eg, blocking) should be provided in a separate document that is unavailable to those who enrol participants or assign interventions	8
Allocation concealment mechanism	#16b	Mechanism of implementing the allocation sequence (eg, central telephone; sequentially numbered, opaque, sealed envelopes), describing any steps to conceal the sequence until interventions are assigned	8
Allocation: implementation	#16c	Who will generate the allocation sequence, who will enrol participants, and who will assign participants to interventions	8
Blinding (masking)	#17a	Who will be blinded after assignment to interventions (eg, trial participants, care providers, outcome assessors, data analysts), and how	8
Blinding (masking): emergency unblinding	#17b	If blinded, circumstances under which unblinding is permissible, and procedure for revealing a participant's allocated intervention during the trial	8
**Methods: Data collection, management, and analysis**			
Data collection plan	#18a	Plans for assessment and collection of outcome, baseline, and other trial data, including any related processes to promote data quality (eg, duplicate measurements, training of assessors) and a description of study instruments (eg, questionnaires, laboratory tests) along with their reliability and validity, if known. Reference to where data collection forms can be found, if not in the protocol	10
Data collection plan: retention	#18b	Plans to promote participant retention and complete follow-up, including list of any outcome data to be collected for participants who discontinue or deviate from intervention protocols	10

For peer review only

Data management	#19	Plans for data entry, coding, security, and storage, including any related processes to promote data quality (eg, double data entry; range checks for data values). Reference to where details of data management procedures can be found, if not in the protocol	10
Statistics: outcomes	#20a	Statistical methods for analysing primary and secondary outcomes. Reference to where other details of the statistical analysis plan can be found, if not in the protocol	9
Statistics: additional analyses	#20b	Methods for any additional analyses (eg, subgroup and adjusted analyses)	9
Statistics: analysis population and missing data	#20c	Definition of analysis population relating to protocol non-adherence (eg, as randomised analysis), and any statistical methods to handle missing data (eg, multiple imputation)	9
**Methods: Monitoring**			
Data monitoring: formal committee	#21a	Composition of data monitoring committee (DMC); summary of its role and reporting structure; statement of whether it is independent from the sponsor and competing interests; and reference to where further details about its charter can be found, if not in the protocol. Alternatively, an explanation of why a DMC is not needed	10-11
Data monitoring: interim analysis	#21b	Description of any interim analyses and stopping guidelines, including who will have access to these interim results and make the final decision to terminate the trial	
Harms	#22	Plans for collecting, assessing, reporting, and managing solicited and spontaneously reported adverse events and other unintended effects of trial interventions or trial conduct	
Auditing	#23	Frequency and procedures for auditing trial conduct, if any, and whether the process will be independent from investigators and the sponsor	10
**Ethics and dissemination**			
Research ethics approval	#24	Plans for seeking research ethics committee / institutional review board (REC / IRB) approval	11

For peer review only

Protocol amendments	#25	Plans for communicating important protocol modifications (eg, changes to eligibility criteria, outcomes, analyses) to relevant parties (eg, investigators, REC / IRBs, trial participants, trial registries, journals, regulators)	
Consent or assent	#26a	Who will obtain informed consent or assent from potential trial participants or authorised surrogates, and how (see Item 32)	8
Consent or assent: ancillary studies	#26b	Additional consent provisions for collection and use of participant data and biological specimens in ancillary studies, if applicable	
Confidentiality	#27	How personal information about potential and enrolled participants will be collected, shared, and maintained in order to protect confidentiality before, during, and after the trial	10
Declaration of interests	#28	Financial and other competing interests for principal investigators for the overall trial and each study site	14
Data access	#29	Statement of who will have access to the final trial dataset, and disclosure of contractual agreements that limit such access for investigators	10
Ancillary and post trial care	#30	Provisions, if any, for ancillary and post-trial care, and for compensation to those who suffer harm from trial participation	
Dissemination policy: trial results	#31a	Plans for investigators and sponsor to communicate trial results to participants, healthcare professionals, the public, and other relevant groups (eg, via publication, reporting in results databases, or other data sharing arrangements), including any publication restrictions	
Dissemination policy: authorship	#31b	Authorship eligibility guidelines and any intended use of professional writers	
Dissemination policy: reproducible research	#31c	Plans, if any, for granting public access to the full protocol, participant-level dataset, and statistical code	
**Appendices**			
Informed consent materials	#32	Model consent form and other related documentation given to participants and authorised surrogates	Supplementary files

For peer review only

Biological specimens	#33	Plans for collection, laboratory evaluation, and storage of biological specimens for genetic or molecular analysis in the current trial and for future use in ancillary studies, if applicable

None The SPIRIT Explanation and Elaboration paper is distributed under the terms of the Creative Commons Attribution License CC-BY-NC. This checklist can be completed online using https://www.goodreports.org/, a tool made by the EQUATOR Network in collaboration with Penelope.ai

For peer review only
